# Molecular cloning of doublesex genes of four cladocera (water flea) species

**DOI:** 10.1186/1471-2164-14-239

**Published:** 2013-04-10

**Authors:** Kenji Toyota, Yasuhiko Kato, Masaru Sato, Naomi Sugiura, Shinichi Miyagawa, Hitoshi Miyakawa, Hajime Watanabe, Shigeto Oda, Yukiko Ogino, Chizue Hiruta, Takeshi Mizutani, Norihisa Tatarazako, Susanne Paland, Craig Jackson, John K Colbourne, Taisen Iguchi

**Affiliations:** 1 Okazaki Institute for Integrative Bioscience, National Institute for Basic Biology, National Institutes of Natural Sciences, and Department of Basic Biology, Faculty of Life Science, Graduate University for Advanced Studies (SOKENDAI), 5-1 Higashiyama, Myodaiji, Okazaki, Aichi, 444-8787, Japan; 2 Department of Biotechnology, Graduate School of Engineering, Osaka University, 2-1 Yamadaoka, Suita, Osaka, 565-0871, Japan; 3 Toyota Nishi High School, 14-65 Kosaka, Toyota, Aichi, 471-0035, Japan; 4 National Institute for Environmental Studies, 16-2 Onogawa, Tsukuba, Ibaraki, 305-8506, Japan; 5 The Center for Genomics and Bioinformatics, Indiana University, 915 East Third Street, Bloomington, IN, 47405, USA; 6 Present address: School of Biosciences, University of Birmingham, Birmingham, B15 2TT, UK

**Keywords:** Cladocera, Doublesex genes, Environmental sex determination, Gene duplication, Annotated regulatory regions

## Abstract

**Background:**

The gene *doublesex* (*dsx*) is known as a key factor regulating genetic sex determination in many organisms. We previously identified two *dsx* genes (*DapmaDsx1* and *DapmaDsx2*) from a freshwater branchiopod crustacean, *Daphnia magna,* which are expressed in males but not in females*. D. magna* produces males by parthenogenesis in response to environmental cues (environmental sex determination) and we showed that *DapmaDsx1* expression during embryonic stages is responsible for the male trait development. The *D. magna dsx* genes are thought to have arisen by a cladoceran-specific duplication; therefore, to investigate evolutionary conservation of sex specific expression of *dsx* genes and to further assess their functions in the environmental sex determination, we searched for *dsx* homologs in four closely related cladoceran species.

**Results:**

We identified homologs of both *dsx* genes from, *D. pulex*, *D. galeata*, and *Ceriodaphnia dubia,* yet only a single *dsx* gene was found from *Moina macrocopa*. The deduced amino acid sequences of all 9 *dsx* homologs contained the DM and oligomerization domains, which are characteristic for all arthropod DSX family members. Molecular phylogenetic analysis suggested that the dsx gene duplication likely occurred prior to the divergence of these cladoceran species, because that of the giant tiger prawn *Penaeus monodon* is rooted ancestrally to both DSX1 and DSX2 of cladocerans. Therefore, this result also suggested that *M. macrocopa* lost *dsx2* gene secondarily. Furthermore, all dsx genes identified in this study showed male-biased expression levels, yet only half of the putative 5’ upstream regulatory elements are preserved in *D*. *magna* and *D*. *pulex*.

**Conclusions:**

The all dsx genes of five cladoceran species examined had similar amino acid structure containing highly conserved DM and oligomerization domains, and exhibited sexually dimorphic expression patterns, suggesting that these genes may have similar functions for environmental sex determination in cladocerans.

## Background

Sex determination is a fundamental developmental process, affecting the sexual differentiation of gonads, and leads to sex-specific differences in behavior, physiology and morphology. Sex-determining systems can be divided into two categories: genotypic sex determination (GSD) and environmental sex determination (ESD) [1-3]. GSD is attributed to the genetic segregation of genes, often residing on sex chromosomes that initiate alternate sex-determining developmental pathways. In contrast, ESD has repeatedly arisen during animal evolution [4] and is initiated by diverse environmental cues, such as temperature, photoperiod, nutrition and population density, that trigger alternative genetic signals, resulting in the regulation of male or female sex-determining genes [5,6].

Natural selection of rare mutational variants has been suggested to mediate the transitions between GSD and ESD [7,8]. A previous phylogenetic analysis revealed that there have been at least three independent switches from GSD to ESD in lizards, and six transitions from ESD to GSD in turtles [9]. Moreover, previous experiments using temperature-sensitive mutations created artificially in *Caenorhabditis elegans* demonstrated how GSD could rapidly evolve into ESD as a consequence of a mutation in key sex determining genes [9]. Orthologs of GSD genes such as *dmrt1*, *sox9* and *cyp19a* (aromatase) are expressed in the gonads during the temperature-sensitive period in ESD of reptiles [10]. Thus, according to the current interpretation of these data, ESD mechanisms are likely to share many sex-determining components with GSD [5].

Sex determination systems in insects vary considerably in key factors and regulatory mechanisms to develop sex-specific traits. The sex determination mechanism in *Drosophila melanogaster* is best understood. The ratio of X chromosomes to autosomes (X:A ratio) is thought to provide the initial signal for the activation of *sex-lethal* (*sxl*), a master gene of the sex determination cascade. Then, *sxl* is produced as the sex-specific splicing isoforms. *Sxl* in female acts on the pre-mRNA of *transformer* (*tra*) resulting in reproduction of functional Tra. The functional Tra in the female, in concert with Tra-2, regulates the production of female-specific *doublesex* (*dsx*) mRNA. The male-specific splice form of *dsx* mRNA is the default splice-variant in *D. melanogaster*. *Dsx* regulates the various sex-specific traits such as gonads. Recently, sex determination mechanisms have also been demonstrated in various insect lineages such as Diptera (*Musca domestica* and *Ceratitis capitata*), Hymenoptera (*Apis mellifera* and *Nasonia vitripennnis*) and Coleoptera (*Tribolium castaneum*). These studies revealed that *tra* and *dsx* are highly conserved among insects [11-14]. However, in case of Lepidoptera, *Bombyx mori*, *tra* and *tra-2* are assumed not to be required for the sex-specific splicing of *Bmdsx* pre-mRNA, because *Bmdsx* has no Tra/Tra-2 binding motif. Recently, it has been revealed that binding of the *BmPSI*, a *Bombyx* homolog of *P-element* somatic inhibitor, to the exonic splicing suppressor sequence on expected region is involved in sex-specific splicing of *Bmdsx*[12]. These data suggest that upstream genetic cascades of dsx might be diverse among insects.

The Cladocera (commonly called water fleas) is an ancient clade of branchiopod crustaceans comprising 16 or 18 family lineages [15,16] that all reproduce by cyclical parthenogenesis involving ESD [17]. The most well studied species are from the family Daphniidae, particularly of the genus *Daphnia*. *Daphnia* inhabit freshwater ponds and lakes on all continents and are known to switch between parthenogenetic and sexual reproduction when environmental conditions for growth and reproduction deteriorate. During normal growing conditions, populations are most often entirely composed of females. However, shortened photoperiod, a lack of food and/or increased population density all lead to the clonal production of males that are genetically identical to their sisters and mothers [18]. First instar male juveniles are easily distinguished from the females [19]. During maturation, daphniids undergo morphological sexual differentiation of various somatic tissues, including the armament of a first thoracic leg with the copulatory hook in males, which becomes larger during the fifth instar [20]. Gonads develop and finally settle at both sides of the gut during embryogenesis in both sexes [21]. The appearance of males allows sexual reproduction to occur [22,23], when females begin producing haploid eggs requiring fertilization.

Recently, we and others found that male production occurred independently of environmental cues by treatment with exogenous juvenile hormone (JH) or its analogs [24,25]. Exposure of *D. magna* to JH analogs at the stage corresponding to the environmentally-sensitive period for sex determination of a cladoceran species of the family Moinidae [26], produced exclusively male broods, suggesting that JH could be a key molecule for understanding mechanisms of ESD [24,27,28].

A *doublesex* (*dsx*) gene was originally identified in *D. melanogaster* as a critical and terminal transcription factor in the fly sex determining cascade. *Dsx* is spatially and temporally transcribed into two sex-specific splice forms conferring sexually dimorphic traits during development [29,30]. The *dsx* gene contains two conserved domains: the Dsx/Mab-3 (DM) domain at the N-terminus and the oligomerization domain at the C-terminus [31]. Genes encoding DM-domain (DM-domain genes) were discovered to play a related role in *C. elegans*[32,33] and also in vertebrates [34-36]. In contrast, results from numerous studies have shown that other genes in the genetic sex-determination cascade widely diversified among species [1,2,37].

To understand the molecular and evolutionary relationships between GSD and ESD, we previously identified and analyzed three DM-domain genes (DMRT11E, DMRT93B and DMRT99B) from *D. magna*, displaying sexual dimorphic gene expression patterns in adult gonads [38]. However, none of these DM-domain genes exhibited sexually dimorphic expression patterns during embryonic development, suggesting that they are not involved in sex determination [38]. Two additional DM-domain genes were later found in the *D. magna* expressed sequence tags (ESTs) database [39]. Therefore, we analyzed the function of these two genes from *D. magna* using gene manipulations that we developed [40]. These experiments revealed that two *dsx* genes in *D. magna* were obtained by lineage-specific duplication, and then one of the paralogs, *Daphnia magna dsx1* (*DapmaDsx1*), plays an important role in directing the major sexually dimorphic development of *D. magna*[41]. In contrast, specific function of *Daphnia magna dsx2* (*DapmaDsx2*) remains unknown. These newly identified *dsx* genes showed greater sequence similarity at the amino acid sequence level to known insect *dsx* genes than to the previously identified DM-domain containing genes in *D. magna*. A genome-wide study of gene functions in *D. pulex* suggested that lineage-specific duplicated genes are most responsive to varying environmental conditions [42]. In the present study, we investigated the sequence and functional conservation of the two *dsx* genes in a broader taxonomic sampling of cladocerans by cloning *dsx* homologs, and determining their sex specific expression in four species representing two families and three genera. We also analyzed the structures of cloned *dsx* genes of *D. magna* and *D. pulex* including their putative regulatory motifs and putative transcription factor binding sites in the 5’ upstream regions of these duplicated *dsx* genes.

## Results and discussion

### Molecular cloning of *doublesex* genes from cladocerans

**Figure 1:**
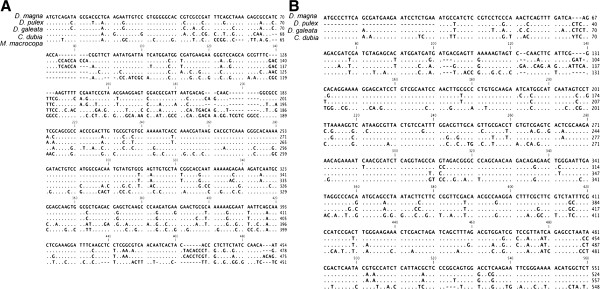
**Nucleotide sequence comparison of *dsx1 *and *dsx2 *genes from five cladocerans.** (**A**) Alignment of nucleotide sequence of *dsx1* genes from *Daphnia magna*, *D. pulex*, *D. galeata*, *Ceriodaphnia dubia* and *Moina macrocopa*. (**B**) Alignment of nucleotide sequence of *dsx2* genes from *D. magna*, *D. pulex*, *D. galeata* and *C. dubia*.

**Figure 2:**
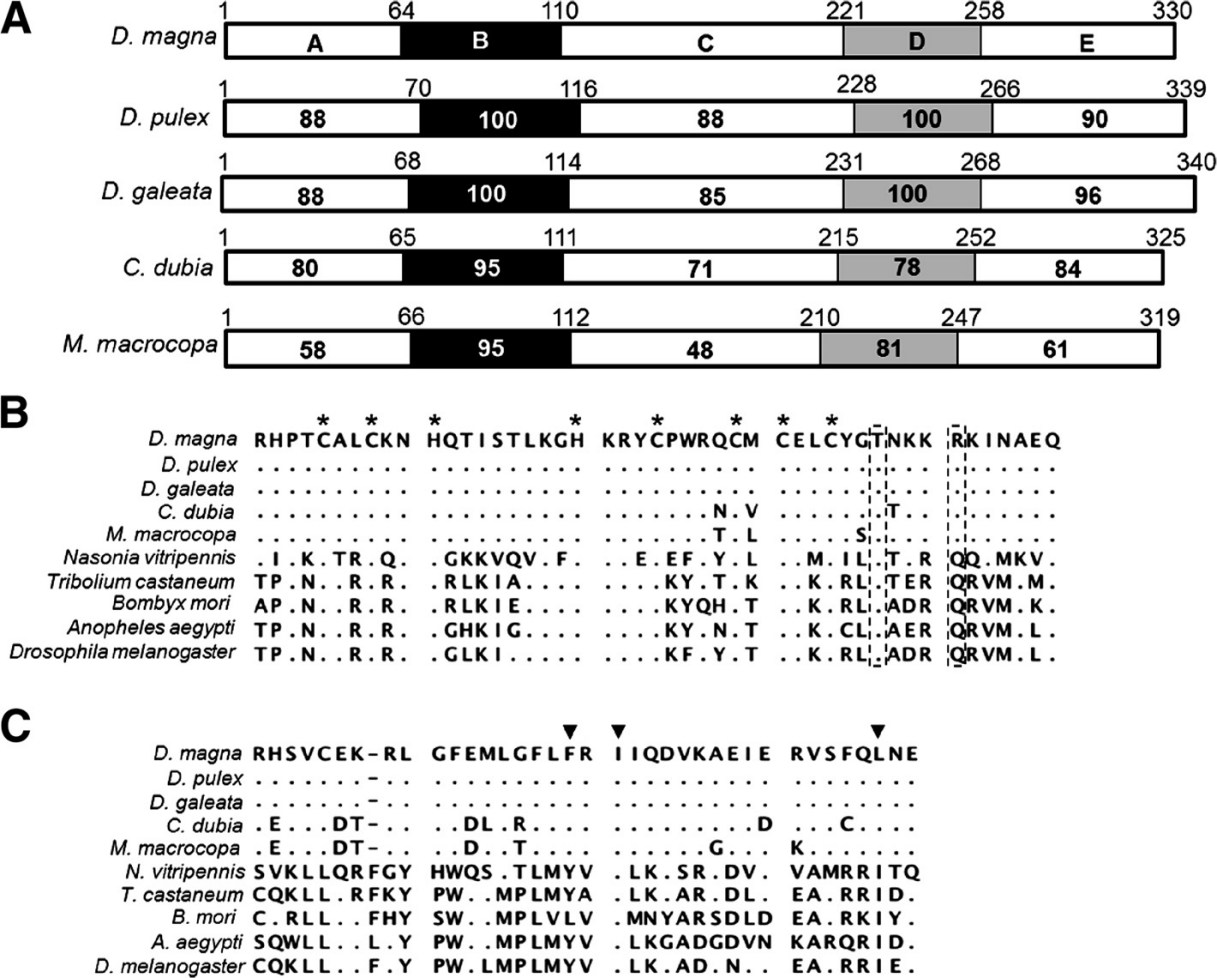
**Schematic diagrams of the DSX1 structures and its sequence comparison of DM- and oligomerization-domains.** (**A**) Domain structures of DSX1 in *Daphnia magna*, and identity with *D. pulex*, *D. galeata*, *C. dubia* and *M. macrocopa*. DM- and oligomerization-domains are indicated by black and gray boxes, respectively. (**B, C**) Alignment of predicted amino acid sequences of DM- and oligomerization-domains of DSX1 from five cladocerans, respectively. Amino acid sequences were aligned using CLUSTAL-X. Dotted boxes highlight the conserved threonine (T) residue in the DM-domain, and arginine (R) residue substituted for glutamine (Q), which is conserved amino acid residues of DSX. Asterisks indicate the zinc chelating residues [43]. Position of non-polar amino acids important in formation of the hydrophobic interface between oligomerization domains in *Drosophila* DSX are indicated with solid triangles [31,41].

**Figure 3:**
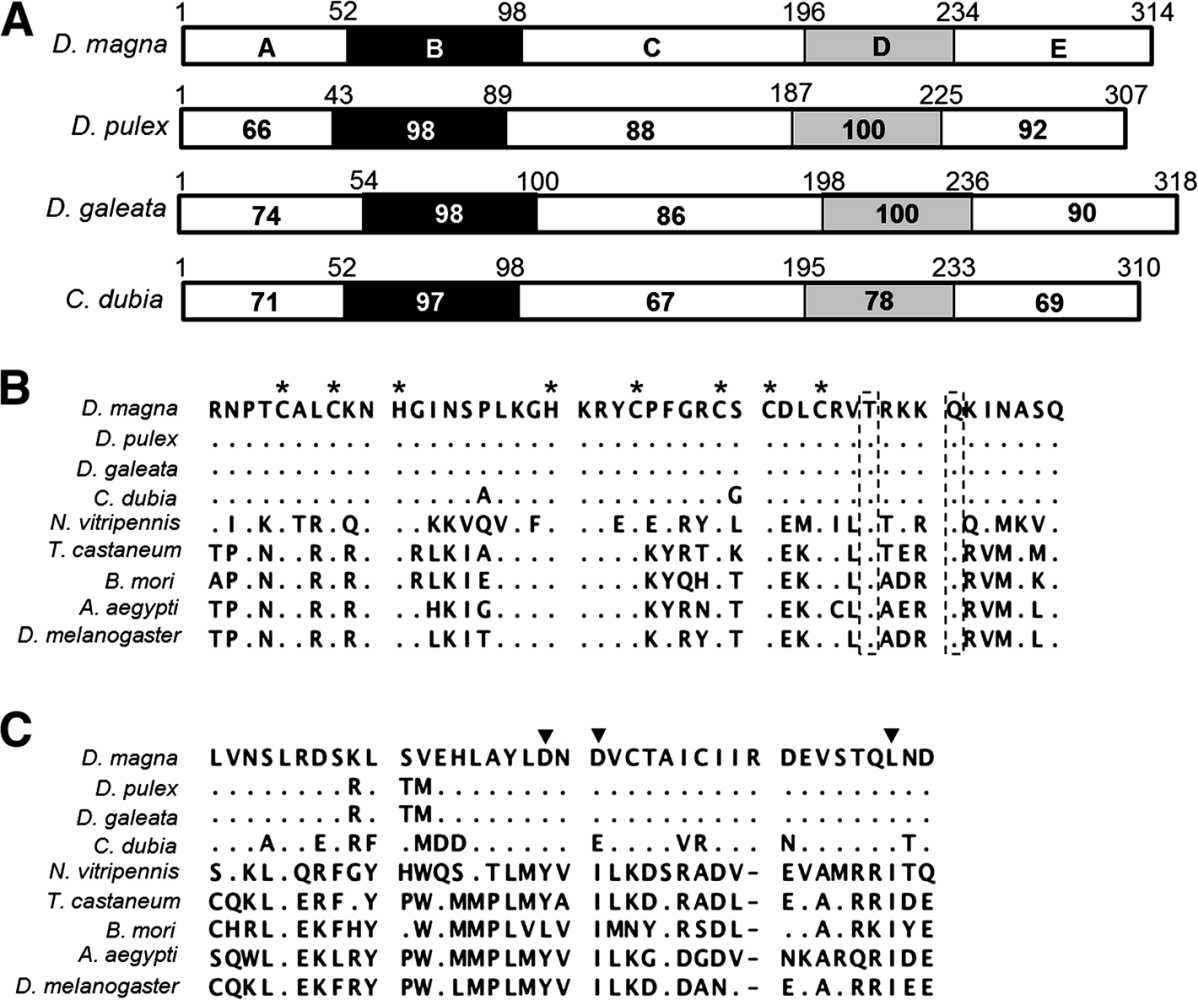
**Schematic diagrams of the DSX2 structures and its sequence comparison of DM- and oligomerization-domains.** (**A**) Domain structures of the DSX2 of *Daphnia magna*, and identity with *D. pulex*, *D. galeata* and *C. dubia*. DM- and oligomerization-domains are indicated by black and gray boxes, respectively. (**B, C**) Alignment of predicted amino acid sequences of DM- and oligomerization-domains of DSX2 from four daphniids, respectively. Amino acid sequences were aligned using CLUSTAL-X. Dotted boxes highlight the conserved threonine (T) and glutamine (Q) residues in DSX2. Asterisks indicate the zinc chelating residues. Position of non-polar amino acids important in formation of the hydrophobic interface between oligomerization-domains in *Drosophila* DSX are indicated with solid triangles [31,41].

**Figure 4:**
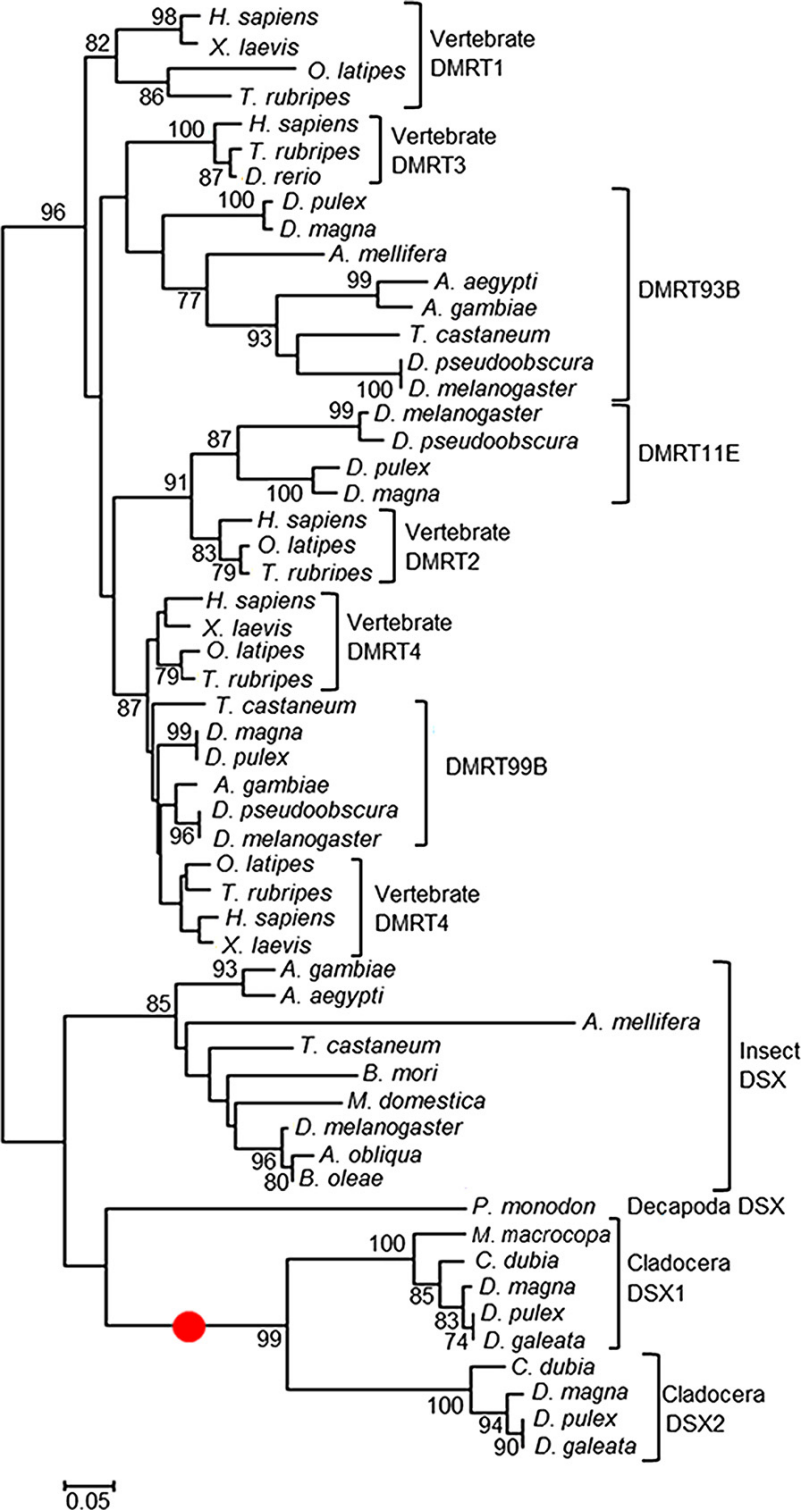
**Phylogeny of DM-domain containing genes based on amino-acid sequence conservation.** The evolutionary history of DM-domain containing genes was inferred by using the Neighbor-Joining method. The percentage of replicate trees in which the associated genes clustered together in the bootstrap test (1,000 replicates) is shown next to the branches (Bootstrap values below 70 percent are not shown). The tree is drawn to scale, with branch lengths in the same units as those of the evolutionary distances used to infer the phylogenetic tree. The evolutionary distances were computed using the Poisson correction method and are in the units of the number of amino acid substitutions per site. The analysis involved 55 amino acid sequences. All positions containing gaps and missing data were eliminated. There were a total of 62 positions in the final dataset. Evolutionary analyses were conducted in MEGA5 [45]. Red spot indicates duplication period of dsx gene duplication in cladocerans.

To verify whether homologs of *dsx* genes among daphniids are conserved, we first cloned *dsx* genes from four cladocerans (*D. pulex*, *D. galeata*, *C. dubia* and *M. macrocopa*), then characterized them by comparison with *dsx* genes of *D. magna* and several insect species [41,43] (Figure 1). As a result, two *dsx* homologs were identified from *D. pulex*, *D. galeata* and *C. dubia*, while only one *dsx* homolog was isolated from *M. macrocopa* (Figure 1B and Additional file 1). The deduced amino acid sequences of all 9 homologs contained the expected DM- and oligomerization-domains, which are characteristic for all arthropod DSX family members [31,44] (Figures 2, 3). Phylogenetic analysis with other known DSX of various species revealed that DSX of cladocerans grouped into two distinct monophyletic groups: DSX1 and DSX2 (Figure 4). Because DSX of the giant tiger prawn *Penaeus monodon* is rooted ancestrally to both DSX1 and DSX2, the gene duplication event likely occurred after the divergence of Branchiopoda and Malacostraca (Figure 4). In the present study, only *dsx1*, but not *dsx2*, was identified from *M. macrocopa*. To test whether another copy might exist, we performed reverse transcription PCR assays using primers corresponding to highly conserved region of *dsx1* and *dsx2* among Daphniidae. Only a single amplified DNA could be detected from both sexes in *M. macrocopa* (Additional file 1). These results suggest that the *dsx* gene duplication occurred prior to the divergence of these cladoceran species, therefore we infer that the *M. macrocopa dsx2* gene was secondarily lost.

By comparing the DapmaDsx1 sequence to orthologs from the four studied species (*D. pulex*, *D. galeata*, *C. dubia* and *M. macrocopa*), we found that DapmaDsx1 shows 88-58%, 100-95%, 88-48%, 100-78% and 96-61% sequence identities to the A, B (DM-domain), C, D (oligomerization-domain) and E domains, respectively (Figure 2A). Similarly, by comparing the DapmaDsx2 sequence with identified orthologs, we observed that DapmaDsx2 shows comparable relative ratios among each of the domains: 74-66%, 98-97%, 88-67%, 100-78% and 92-69% sequence identities to the A, B, C, D and E domains, respectively (Figure 3A). These results suggest that putative amino acids of both the DM- and oligomerization-domains are highly conserved among the Cladocera. On the other hand, amino acid similarities outside of these domains are lower, and are proportional to the evolutionary distance between each genus; *Daphnia*, *Ceriodaphnia* and *Moina*[46] (Additional files 2 with 3).

The DM-domain contains zinc chelating residues, and among the insects studied to date, two highly conserved amino acid residues, threonine and glutamine (Boxed in Figures 2B, 3B), distinguish the DM-domain of DSX from DM-domains of other insect proteins [43]. Therefore, we searched for similar highly conserved amino acid residues within the DSX DM-domains of cladocerans. Indeed, all zinc chelating residues are found to be conserved in the DM domains of DSX1 and DSX2 among the five cladoceran species (Figures 2B, 3B). Yet, although the threonine and glutamine residues were conserved in DSX2, the glutamine residue in DSX1 was substituted by arginine in all cladoceran species examined (Figures 2B, 3B). These results suggest that DSX1 in cladocerans might have gained a novel function affecting sex determination by amino acid replacement after duplication of *dsx* in branchiopoda lineage.

Compared to the DM-domains, more amino acid variation is observed in alignments of the oligomerization-domains (Figures 2C, 3C). Dimerization, which enhances specific DNA binding, is mediated by several residues in a non-polar interface that is conserved within oligomerization-domains. Previous study has revealed that, in DSX2 of *D. magna*, two of three nonpolar amino acids indispensable for formation of the nonpolar interface are substituted with the polar acidic amino acid, aspartic acid [41]. This suggests that the daphniid DSX2 proteins are unable to dimerize and may not be functional or may have a different, unknown functions.

### Sex specific expression of *dsx* genes in five cladoceran species

We previously reported that both *dsx* genes in *D. magna* were transcriptionally up-regulated in males and showed no sex-specific splice isoforms. We and others also reported that exposure to JH analogs reliably produces male daphniids [24,25]. Moreover, by using gene knock-down (RNAi) and overexpression methods in *D. magna*[40,41], we discovered that *DapmaDsx1* is necessary and sufficient for sex determination in *D. magna*, whereas the tandemly duplicated *DapmaDsx2* gene does not determine sex, even though its transcription is equally sex-biased [41].

**Figure 5:**
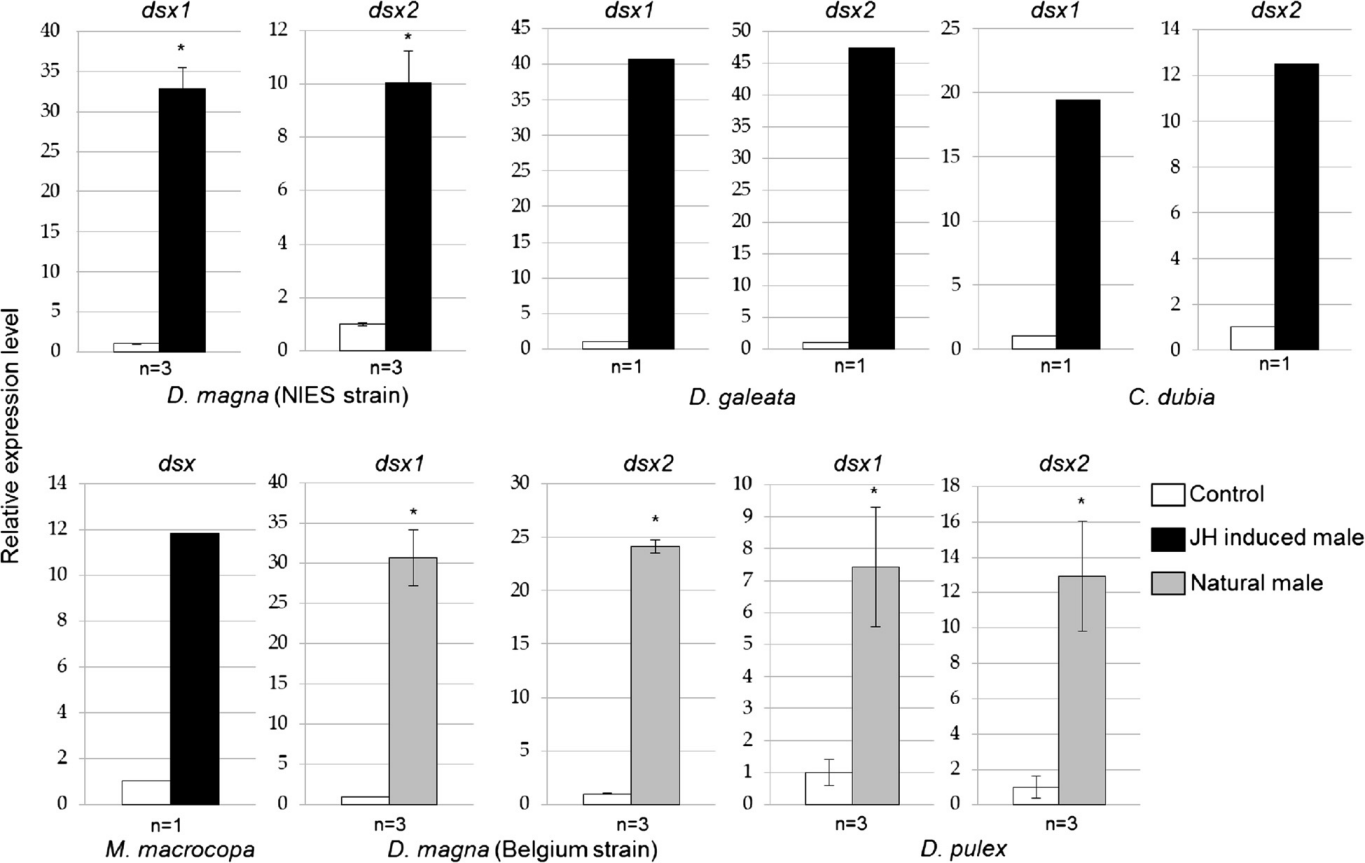
**Relative transcriptional expression levels of *dsx1 *and *dsx2 *genes in adult males compared with females.** The males of *D. magna* (NIES strain), *D. galeata*, *C. dubia* and *M. macrocopa* were induced by JH analog exposure. *D. magna* (Belgium strain) and *D. pulex* males were induced by environmental cues (see Materials and methods). These genes showed higher expression levels in males using both JHs (black) and natural cues (grey) than in females (white). Y-axes indicate relative expression levels normalized by female expression levels. Bars indicate standard errors. Numbers indicate the biological replicates. Asterisks indicate significant differences (P < 0.05, based on Student’s *t*-test).

In this study, we confirmed that expression patterns of *DapmaDsx1* and *DapmaDsx2* homologous genes are conserved in other cladocerans, by studying steady state mRNA levels for *dsx* transcripts in adult females and males by quantitative PCR. We found that the mRNA levels of these *dsx* genes range from seven to forty fold greater in males than in females (Figure 5). Our results indicate that the sexual dimorphic mRNA expression patterns of *dsx* are conserved among daphniids and *Moina*.

### Annotation of *dsx* gene structures in *D. Magna* and *D. Pulex*

We previously cloned and described the mRNA transcripts of the *DapmaDsx1* and *DapmaDsx2* genes [41]. *DapmaDsx1* produces two mRNA, *DapmaDsx1-α* and *DapmaDsx1-β*, which are expressed in both sexes and differ only in their 5’ UTR, while *DapmaDsx2* produces only one mRNA transcript. The *D. pulex* draft genome sequence was recently published [42], and the *D. magna* genome sequencing project is currently in progress by the *Daphnia* Genomics Consortium. The *DapmaDsx1-α*, *DapmaDsx1-β*, and *DapmaDsx2* mRNA transcripts were aligned to the genome assemblies and used to annotate the *dsx1* and *dsx2* gene models in the *D. magna* and *D. pulex* genomes (Additional file 4).

The *D. magna dsx* gene cluster is located on scaffold 2190 of the *D. magna* genome assembly v2.4 with a ~10 Kbp intergenic region between the *DapmaDsx1* (~16.1 Kbp length) and *DapmaDsx2* (~1.6 Kbp length) genes (Additional file 4A). The second exon of the *DapmaDsx1-β* mRNA transcript fell within an assembly gap in scaffold 2190, but was located on scaffold 521. We conclude that scaffold 521 (~5.8 Kbp length) characterizes a ~3.1 Kbp gap in scaffold 2190, located in the intragenic region of *DapmaDsx1*.

The *D. pulex dsx* gene cluster is located on scaffold 32 of the *D. pulex* genome assembly v1.1 with a ~9.6 Kbp intergenic region between the *DappuDsx1* (~21.5 Kbp length) and *DappuDsx2* (~1.6 Kbp length) genes (Additional file 4B). The *D. pulex* v1.1 gene model predictions did not correctly identify the *dsx1* and *dsx2* duplicate gene cluster. Our annotations improved the *dsx* gene models in both *D. magna* and *D. pulex* genomes*.*

### Potential transcriptional regulatory elements in the 5’ upstream promoter regions of the *dsx* genes

To further annotate putative functional and conserved elements of the *dsx* genes, we searched and compared transcriptional promoter regions of the genes in *D. magna* and *D. pulex*. 1.0 Kbp upstream of the transcription start site (TSS) of *dsx1-α*, *dsx1-β*, and *dsx2* were extracted as transcriptional regulatory regions. This interval spans the predicted intergenic region between adjacent loci. Promoter sequences are challenging for multiple alignment algorithms, because upstream regulatory regions are not well conserved compared to protein coding regions of genes [47,48]. We aligned orthologous promoter sequences from *D. magna* and *D. pulex* using Pro-Coffee [47], an alignment algorithm specifically designed for homologous promoter regions (Additional files 5, 6, 7). The *DapmaDsx1-α* and *DappuDsx1-α* promoter alignment showed 46% sequence identity (Additional file 5), the *DapmaDsx1-β* and *DappuDsx1-β* promoter alignment showed 60% sequence identity (Additional file 6), and the *DapmaDsx2* and *DappuDsx2* promoter alignment showed 62% sequence identity (Additional file 7). The promoter regions of *dsx1* and *dsx2* have much less conservation than their respective protein coding regions, which have 84% and 83% sequence identity, respectively (92% and 84% sequence identity at synonymous sites on protein coding region, respectively).

**Figure 6:**
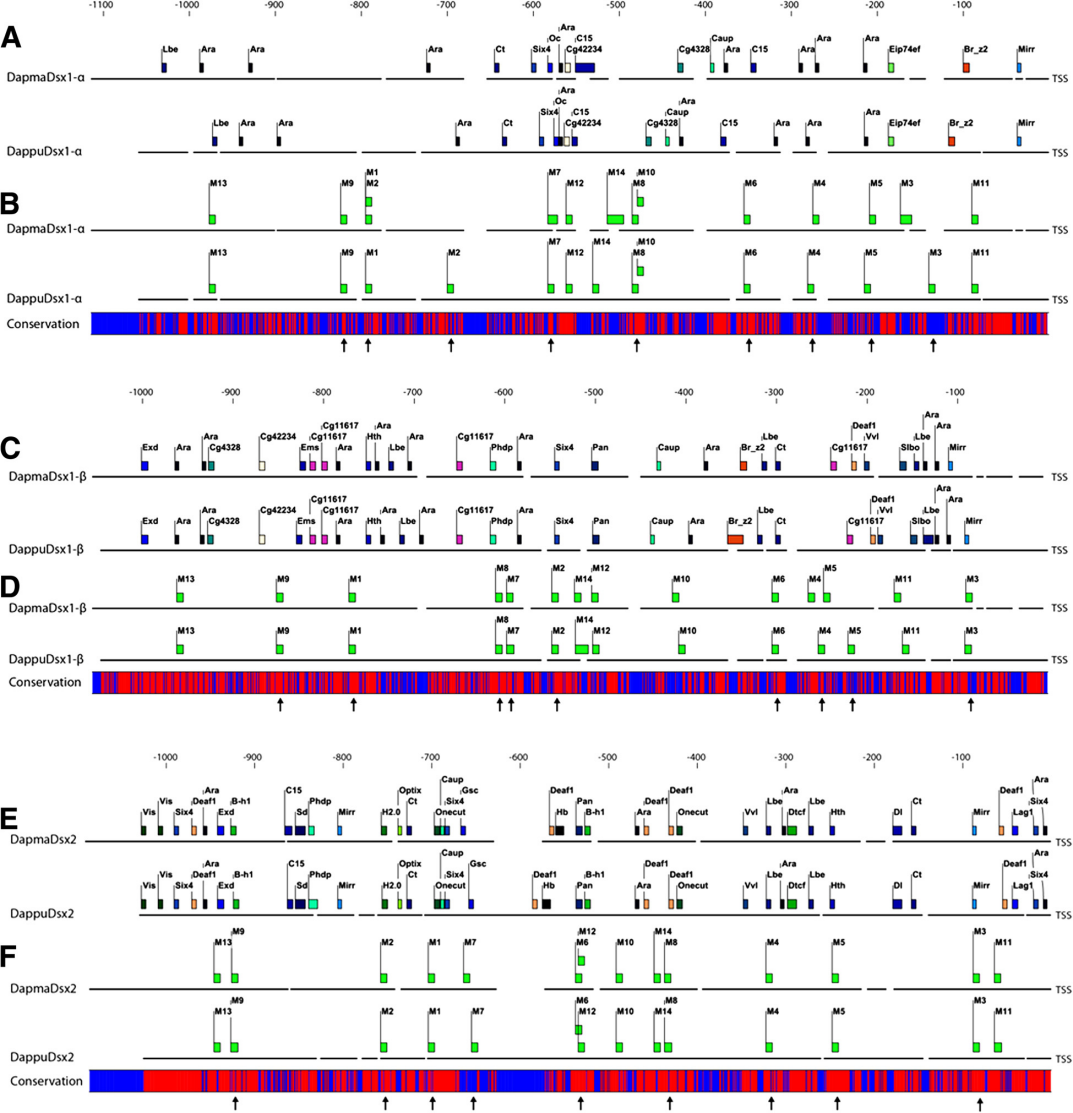
**Annotations mapped onto Pro-Coffee alignment of *dsx1-α *, *dsx1-β *and *dsx2 *5’ upstream promoter regions.** (**A**) Putative known transcription factor binding sites in *D. magna* and *D. pulex dsx1-α* promoter regions predicted by TF-map alignment algorithm. (**B**) *dsx1-α* promoter locations of conserved regulatory motifs predicted in both *D. magna* and *D. pulex dsx* promoter regions. (**C**) Putative known transcription factor binding sites in *D. magna* and *D. pulex dsx1-β* promoter regions predicted by TF-map alignment algorithm. (**D**) *dsx1-β* promoter locations of conserved regulatory motifs predicted in all *D. magna* and *D. pulex dsx* promoter regions. (**E**) Putative known transcription factor binding sites in *D. magna* and *D. pulex dsx2* promoter regions predicted by TF-map alignment algorithm. (**F**) *dsx2* promoter locations of conserved regulatory motifs predicted in all *D. magna* and *D. pulex dsx* promoter regionArrows underneath denote the conserved regulatory motifs also found *D. melanogaster dsx* promoter region.

We characterized putative known transcription factor binding sites (TFBS) in the *dsx* upstream promoter regions using transcription factor map (TF-map) alignments [48] between orthologous *dsx* promoter regions in *D. magna* and *D. pulex*, based on matches to position frequency matrices (PFMs) from JASPAR [49] and TRANSFAC [50] TFBS databases. The optimal *dsx1-α* promoter TF-map alignment contains 20 putative known TFBSs (Figure 6A, Additional file 8). The optimal *dsx1-β* promoter TF-map alignment contains 31 putative known TFBSs (Figure 6C, Additional file 9). The optimal *dsx2* promoter TF-map alignment contains 39 putative known TFBSs (Figure 6E, Additional file 10). The positions of the putative TFBS pairs (between orthologous promoters) are well aligned when annotated onto the promoter sequence Pro-Coffee alignments (Figure 6), suggesting these predicted putative TFBSs are conserved between *D. magna* and *D. pulex*.

**Figure 7:**
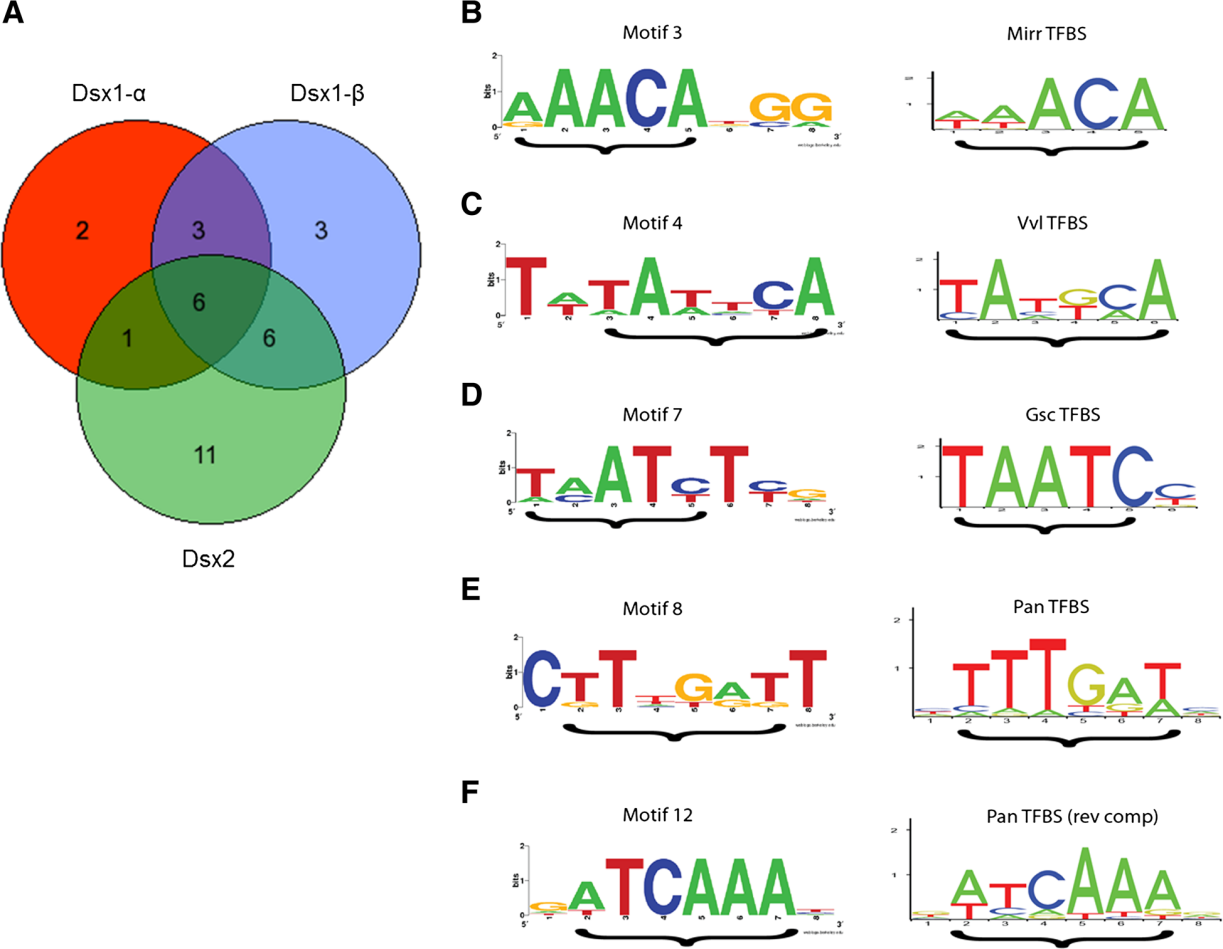
**Venn diagram of putative transcriptional factors and sequence logos of *de novo dsx *promoter motifs.** (**A**) Venn diagram showing the number of unique putative transcription factors shared amongst the *D. magna* and *D. pulex dsx1-α, dsx1-β,* and *dsx2* TF-map alignments. (**B-F**) Braces under the sequence logos denote the similar regions between the *de novo* motif and known TFBS. Sequence logos were created with WebLogo [51]. (**B**) Motif 3 and Mirr TFBS (**C**) Motif 4 and Vvl TFBS (**D**) Motif 7 and Gsc TFBS (**E**) Motif 8 and Pan TFBS (**F**) Motif 12 and Pan reverse complement TFBS.

We compared the number of unique predicted transcription factors (TFs) shared amongst the *dsx* promoter regions (Figure 7A). In total, 32 unique TFs were predicted in the *dsx* promoters, half (16) are present in at least two of the promoters (Additional file 11). Six unique TFs were predicted in all three *dsx* promoters; an additional six unique TFs were also shared between *dsx1-β* and *dsx2* promoter regions. Interestingly, 11 unique TFs were predicted in the *dsx2* promoter but not in either *dsx1* promoters. We previously showed that *dsx1-β* mRNA expression levels are three times greater than expression levels of *dsx1-α* during male *D. magna* development, and that transcription of *dsx2* is even greater than both *dsx1* mRNAs combined [41]. The shared TF motifs suggest a duplication history involving at least part of the 5’ region upstream of *dsx1-β,* while numeric differences observed among *dsx* promoter regions are reflective of these expression level differences. Based on the promoter sequence conservation between *D. magna* and *D. pulex*, and the greater number of predicted TFs, the *dsx1-β* promoter seems to be the more widely used and evolutionarily conserved, while the *dsx1-α* promoter has experienced more sequence divergence and loss of TFBSs.

Since we previously reported that *DapmaDsx1* and *DapmaDsx2* are paralogs, and that both *DapmaDsx1* and *DapmaDsx2* mRNAs are transcriptionally up-regulated in male *D. magna*[41], we searched for *de novo* conserved motifs present in all *dsx* promoter regions in *D. magna* and *D. pulex* (*DapmaDsx1-α*, *DappuDsx1-α, DapmaDsx1-β, DappuDsx1-β, DapmaDsx2,* and *DappuDsx2*), without reference to TFBS sequence databases. We identified 14 conserved motifs in the *Daphnia dsx* promoters (Additional file 12), which can later be functionally investigated as potential TFBSs and/or potential transcriptional promoters of *Daphnia dsx*. The motifs were labeled M1 through M14 and annotated onto the *D. magna/D. pulex dsx* promoter alignments (Figures 6B, 6D and 6F). Motifs 1 through 9 were also found in the *D. melanogaster dsx* upstream promoter region, supporting the conservation and potential regulatory functions of these motifs.

In order to assess whether these *de novo* conserved motifs are similar to any known TFBSs, we scanned the individual motif sequences for matches to TFBS PFMs and compared each motif consensus sequence to known TFBS consensus sequences. Several of the conserved motifs showed similarity to known TFBSs (Figures 7B-F). Motif 3 matches the TFBS of Mirr, a homeobox transcription factor in the Iroquois complex of transcription factors, which is predicted in all three *dsx* promoter TF-map alignments (Figure 7B). Motif 4 matches the TFBS of Vvl, a homeobox transcription factor which is predicted in both *dsx1-β* and *dsx2* TF-map alignments (Figure 7C). Motif 7 matches the TFBS of Gsc/Bcd/Oc, three homeobox transcription factors with nearly identical binding sites (Figure 7D), with four of the Motif 7 sequences matching the Gsc/Bcd/Oc consensus TAATC exactly. Gsc was also predicted in the *dsx2* TF-map alignment, and Oc was predicted in the *dsx1-α* TF-map alignment. Motif 8 matches the TFBS of Pan, a high mobility group transcription factor, which is predicted in both *dsx1-β* and *dsx2* TF-map alignments (Figure 7E). Motif 12 also matches the TFBS of Pan, but with the reverse complement of the binding site (Figure 7F). The similarity of these *de novo* conserved motifs to known TFBSs that were also predicted by the TF-map alignment further supports our results describing the regulatory elements of the *Daphnia dsx* genes.

## Conclusions

In summary, we identified the orthologs of *DapmaDsx1* and *DapmaDsx2* genes from closely related species belonging to two cladoceran families and three genera: *D. pulex*, *D. galeata*, *C. dubia* (Daphniidae) and *M. macrocopa* (Moinidae), with highly conserved DM- and oligomerization-domains. All five species examined exhibited sexually dimorphic expression pattern of *dsx* genes, suggesting that these genes may have similar functions for sex determination in cladocerans. Daphniids are unique animals that exhibit ESD and are; therefore, attractive for understanding the evolution of ESD. We also identified potential regulatory motifs and transcription factor binding sites in the putative promoter regions of these genes in *D. magna* and *D. pulex.* This information will facilitate future study of molecular mechanisms underlying sex-determination in cladocerans.

## Methods

### *Daphnia* strain and culture conditions

Isoclonal strains of *D. magna* (NIES and Belgium strains), *D. galeata*, *C. dubia* and *M. macrocopa* were obtained from the National Institute for Environmental Studies (NIES; Tsukuba, Japan) [25,52]. *D. pulex* was obtained from Hokkaido University, Sapporo, Japan [53], and maintained as described previously [27]. Briefly, culture medium was prepared using charcoal-filtered tap water and cultures of 20 individuals per liter were incubated at 21 ± 1°C under a 14-h light/10-h dark photoperiod. A 0.01-ml suspension of 4.3 × 10^8^ cells ml^−1^ Chlorella (*Chlorella vulgaris*) was added daily to each culture. The water hardness was between 72 and 83 mg L^−1^, the pH between 7.0 and 7.5, and the dissolved oxygen concentration between 80 and 99%. To obtain natural male embryos, adult *D. magna* (Belgium clone) was reared in crowded conditions, and *D. pulex* was incubated at 18°C under a 10-h light/14-h dark photoperiod, and a 0.01-ml suspension of 4.3 × 10^8^ cells ml^−1^ Chlorella was added every two days. To obtain male embryos of *D. magna* (NIES clone)*, D. galeata, C. dubia and M. macrocopa*, (in which natural males are rarely seen) adult individuals (about 2 weeks of age) were chemically induced to produce males by treating them with a synthetic JH analog, fenoxycarb (1 μg/L) (technical grade 96.6% pure, Wako Pure Chemical Industries, Ltd., Osaka, Japan) [25]. We confirmed the offspring sexes by the length of the first antenna [19] observed and photographed using a Leica MZ APO dissecting microscope (Leica, Mannheim, Germany).

### Cloning of *dsx* genes

The nucleotide sequences of the *D. magna dsx* genes were used for designing primers that amplify *dsx* genes in four different species. The harvested animals were homogenized using the Micro Smash MS-100R (Tomy, Tokyo, Japan). Total RNA was extracted with ISOGEN reagent according to the manufacturer’s protocol (NIPPON GENE, Tokyo, Japan). Poly (A) + RNA was isolated from purified total RNA using Fast Track (Life Technologies, Carlsbad, CA USA) and converted to cDNA using Superscript III and random primers (Life Technologies) according to the manufacturer’s protocol. cDNAs corresponding to the EST sequences were obtained by PCR amplification, and full-length cDNAs were obtained by RACE (Cap Fishing; SeeGene, Seoul, South Korea) using the oligonucleotide sequences as shown in Additional file 13.

### Phylogenetic analysis of the DM-domain genes

A phylogenetic tree of DM-domain genes including newly cloned *D. pulex*, *D. galeata*, *C. dubia and M. macrocopa dsx* genes were constructed using amino acid sequences of DM-domain genes used in the previous study [38] (Additional file 14). A multiple alignment was constructed using Clustal W [45,54] with the following settings (pairwise alignment parameters: gap opening penalty 15, gap extension penalty 6.66, identity protein weight matrix; multiple alignment parameters: delay divergent cutoff 30%, gap separation distance 4). Phylogenetic reconstruction was performed using the maximum likelihood and the neighbor-joining methods implemented in *MEGA* version 5 [45].

### Quantitative PCR

Two to three weeks old male and female animals of the five cladoceran species were used in quantitative-PCR (q-PCR) assays of gene expression levels. mRNAs were quantified as described previously [38]. Animals were washed briefly and soaked in RNAlater (Life Technologies) for 10 min. Total RNA was purified and cDNA was synthesized as described above except that a random oligonucleotide was used as the primer. PCR was performed in an ABI Prism 7000 (Life Technologies) using the SYBR-Green PCR core reagents kit (Life Technologies), in the presence of appropriate primers. PCR amplifications were performed using the following conditions: 2 min at 50°C and 10 min at 95°C, followed by a total of 40 two-temperature cycles (15 s at 95°C and 1 min at 60°C).

The primers were chosen to amplify short PCR products of 150 bp; the primer sequences are listed in Additional file 15. Ribosomal protein L32 gene was used for normalization purposes [21,55]. Data acquisition and analysis were performed by ABI Prism 7000 SDS software ver. 1.1 (Life Technologies). The baseline and threshold for the Ct (cycle threshold) were set automatically. Each gene was tested in technical triplicate samples by the relative standard curve method. In the case of *D. magna* and *D. pulex*, each experiment was performed in biological triplicate and statistical analyses were applied.

### *dsx* gene annotations

The genomic locations of *DapmaDsx1-α*, *DapmaDsx1-β*, and *DapmaDsx2* mRNA transcripts were identified using BLASTN sequence similarity searches against a reference blast database of the *D. magna* genome assembly v2.4 scaffolds, and against a reference blast database of the *D. pulex* genome assembly v1.1 scaffolds. The best BLAST matches were analyzed and used to map the gene exons onto the *D. magna* and *D. pulex* scaffolds. ESTs mapped onto the genome assembly with PASA [56], microarray tiling path expression data, and RNA-Seq data from wFleaBase [57] were used as supporting evidence for exon annotations. The *dsx* gene annotation figures were created with AnnotationSketch [58] (Figure 6).

### Transcription factor map alignments

Matscan [48] was used to search for matches to 125 JASPAR core insect TFBS matrices and 44 TRANSFAC insect TFBS matrices in each *dsx* promoter region in *D. magna* and *D. pulex*. A threshold of 0.85 matrix similarities was used to find TFBS matrix matches in the promoter sequences. For each promoter sequence, the collection of TFBS matrix matches is referred to as its TF-map. The TF-maps for each promoter sequence can be found in the following Additional files: *DapmaDsx1-α* (Additional file 16), *DappuDsx1-α* (Additional file 17), *DapmaDsx1-β* (Additional file 18), *DappuDsx1-β* (Additional file 19), *DapmaDsx2* (Additional file 20), *DappuDsx2* (Additional file 21). Meta [48] was then used to find the best meta-alignment of the orthologous promoters TF-maps (with parameters: a = 0.5, l = 0.1, m = 0.1).

### *de novo* conserved promoter motifs

The 5^′^ 1.0 Kbp upstream region was extracted from all *D. pulex* gene models and used to create a background frequency model for 8 bp length motifs in *Daphnia* promoter regions. WeederH [59] was used to search for conserved regulatory motifs of length 8 present in all six *dsx* promoters in *D. magna* and *D. pulex*. The WeederH algorithm measures conservation based on the sequence conservation as well as the motif occurrence’s positions relative to the TSS. WeederH produces a χ^2^ score assessing how conserved the motif is compared to the rest of the homologous sequences. We used a χ^2^ score threshold of 3, discarding motifs with a χ^2^ score less than 3.

## Competing interests

The authors declare that they have no competing interests.

## Authors’ contributions

KT, YK, HM, HW, NT and TI designed the experiments; KT, YK, MS, NS, SM, HM, SO, YO, CH and TM performed the experiments; KT, HM, JKC, TI, SP and CJ analyzed the data; KT, HM, CJ, JKC and TI wrote the paper. All authors have read and approved the final manuscript.

## Supplementary Material

Additional file 1 **RT-PCR of oligonucleotides corresponding to highly conserved region of *dsx1* (A) and *dsx2* (B).** The amplified cDNAs were analyzed by agarose gel electrophoresis. Lane M: molecular weight marker. Lane 1 to 10: *D. magna* (female), *D. magna* (male), *D. pulex* (female), *D. pulex* (male), *D. galeata* (female), *D. galeata* (male), *C. dubia* (female), *C. dubia* (male), *M. macrocopa* (female), *M. macrocopa* (male). Click here for file

Additional file 2 **Estimation of evolutionary divergence between the DSX1 except for DM- and oligomerization-domain and COI sequences.** The number of amino acid differences per site between sequences are shown. The analysis involved 5 amino acid sequences. All positions containing gaps and missing data were eliminated. There were total of 225 and 208 positions in the final dataset of DSX1 and COI, respectively. Evolutionary analyses were conducted in MEGA5 [45]. Click here for file

Additional file 3 **Estimation of evolutionary divergence between the DSX2 except for DM- and oligomerization-domain and COI sequences.** The number of amino acid differences per site between sequences are shown. The analysis involved 5 amino acid sequences. All positions containing gaps and missing data were eliminated. There were total of 210 and 208 positions in the final dataset of DSX2 and COI, respectively. Evolutionary analyses were conducted in MEGA5 [45]. Click here for file

Additional file 4 **Gene model annotations on the *D. magna* and *D. pulex* genome assembly.** (A) *D. magna dsx1* and *dsx2* gene model annotations on the *D. magna* genome assembly. (B) *D. pulex dsx1* and *dsx2* gene model annotations on the *D. pulex* genome assembly. Figures were created with AnnotationSketch [58]. Click here for file

Additional file 5 **Nucleotide sequence comparison of *dsx1-α *promoter regions in *D. magna *and *D. pulex.*** Pro-Coffee alignment of *Dsx1-α* 1.0 Kbp upstream promoter region from *D. magna* and *D. pulex*. Click here for file

Additional file 6 **Nucleotide sequence comparison of *dsx1-β *promoter regions in *D. magna *and *D. pulex. ***Pro-Coffee alignment of *dsx1-β* 1.0 Kbp upstream promoter region from *D. magna* and *D. pulex*. Click here for file

Additional file 7 **Nucleotide sequence comparison of *dsx2 *promoter regions in *D. magna *and *D. pulex.*** Pro-Coffee alignment of *dsx2* 1.0 Kbp upstream promoter region from *D. magna* and *D. pulex*. Click here for file

Additional file 8 ***dsx1-α *TF-map alignment.** Click here for file

Additional file 9 ***dsx1-β *TF-map alignments.** Click here for file

Additional file 10 ***dsx2 *TF-map alignments.** Click here for file

Additional file 11 TF-map alignments unique TFs comparison. Click here for file

Additional file 12 ***de novo *conserved regulatory motifs.** Click here for file

Additional file 13 **Primer sequences for 5’ and 3’ RACE in *D. galeata, C. dubia *and *M. macrocopa.*** Click here for file

Additional file 14 Accession numbers of the sequences for phylogenetic analysis. Click here for file

Additional file 15 Primer sequences for quantitative PCR. Click here for file

Additional file 16 ***DapmaDsx1-α *TF-map.** Click here for file

Additional file 17 ***DappuDsx1-α *TF-map.** Click here for file

Additional file 18 ***DapmaDsx1-β *TF-map.** Click here for file

Additional file 19 ***DappuDsx1-β *TF-map.** Click here for file

Additional file 20 ***DapmaDsx2 *TF-map.** Click here for file

Additional file 21 ***DappuDsx2 *TF-map.** Click here for file
