# Supplementary material for: Molecular cloning of doublesex genes of four cladocera (water flea) species
Source: BMC Genomics. 2013 Apr 10;14:239. doi: 10.1186/1471-2164-14-239 (PMC3637828; doi:10.1186/1471-2164-14-239)
Supplement: Additional file 1 — RT-PCR of oligonucleotides corresponding to highly conserved region of dsx1 (A) and dsx2 (B). The amplified cDNAs were analyzed by agarose gel electrophoresis. Lane M: molecular weight marker. Lane 1 to 10: D. magna (female), D. magna (male), D. pulex (female), D. pulex (male), D. galeata (female), D. galeata (male), C. dubia (female), C. dubia (male), M. macrocopa (female), M. macrocopa (male). [file 1471-2164-14-239-S1.doc]

Supplemental Material 1.
