# Supplementary material for: Molecular cloning of doublesex genes of four cladocera (water flea) species
Source: BMC Genomics. 2013 Apr 10;14:239. doi: 10.1186/1471-2164-14-239 (PMC3637828; doi:10.1186/1471-2164-14-239)
Supplement: Additional file 2 — Estimation of evolutionary divergence between the DSX1 except for DM- and oligomerization-domain and COI sequences. The number of amino acid differences per site between sequences are shown. The analysis involved 5 amino acid sequences. All positions containing gaps and missing data were eliminated. There were total of 225 and 208 positions in the final dataset of DSX1 and COI, respectively. Evolutionary analyses were conducted in MEGA5 [45]. [file 1471-2164-14-239-S2.doc]

Supplemental Material 2. Estimates of evolutionary divergence between the DSX1 except for DM- and oligomerization-domain and COI

|  | *D. magna* | *D. pulex* | *D. galeata* | *C. dubia* | *M. macrocopa* |
| --- | --- | --- | --- | --- | --- |
| *D. magna* | - | 0.0288 | 0.0288 | 0.0240 | 0.0721 |
| *D. pulex* | 0.0622 | - | 0.0192 | 0.0288 | 0.0721 |
| *D. galeata* | 0.0533 | 0.0267 | - | 0.0240 | 0.0673 |
| *C. dubia* | 0.1822 | 0.1956 | 0.1733 | - | 0.0625 |
| *M. macrocopa* | 0.3778 | 0.3911 | 0.3778 | 0.3911 | - |
