# Supplementary figures and images for: Molecular cloning of doublesex genes of four cladocera (water flea) species
Source: BMC Genomics. 2013 Apr 10;14:239. doi: 10.1186/1471-2164-14-239 (PMC3637828; doi:10.1186/1471-2164-14-239)

Supplemental Material 4.


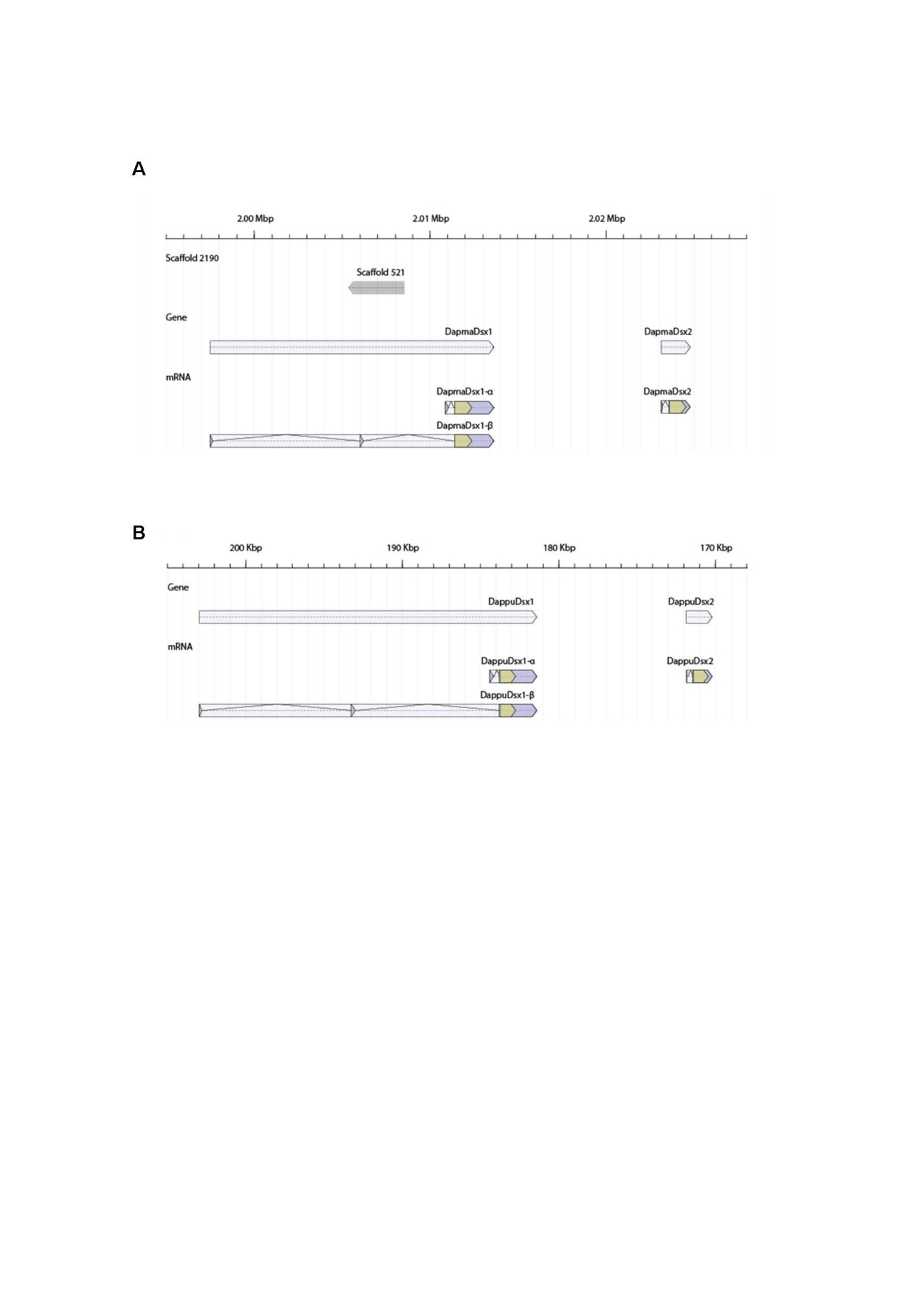

Supplement: Additional file 4 — Gene model annotations on the D. magna and D. pulex genome assembly. (A) D. magna dsx1 and dsx2 gene model annotations on the D. magna genome assembly. (B) D. pulex dsx1 and dsx2 gene model annotations on the D. pulex genome assembly. Figures were created with AnnotationSketch [58]. [file 1471-2164-14-239-S4.doc]

Supplemental Material 5.


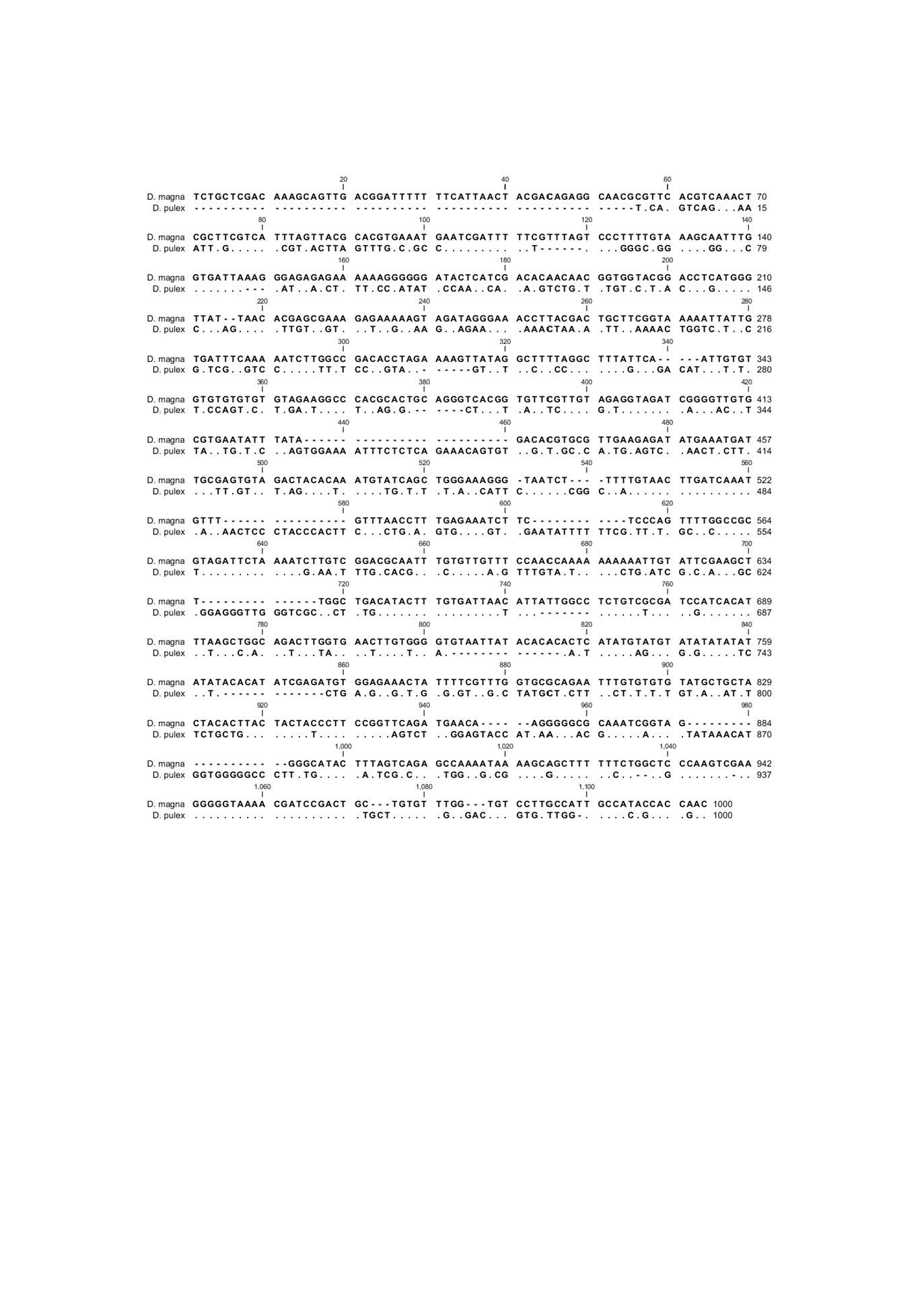

Supplement: Additional file 5 — Nucleotide sequence comparison of dsx1-α promoter regions in D. magna and D. pulex. Pro-Coffee alignment of Dsx1-α 1.0 Kbp upstream promoter region from D. magna and D. pulex. [file 1471-2164-14-239-S5.doc]

Supplemental Material 6.


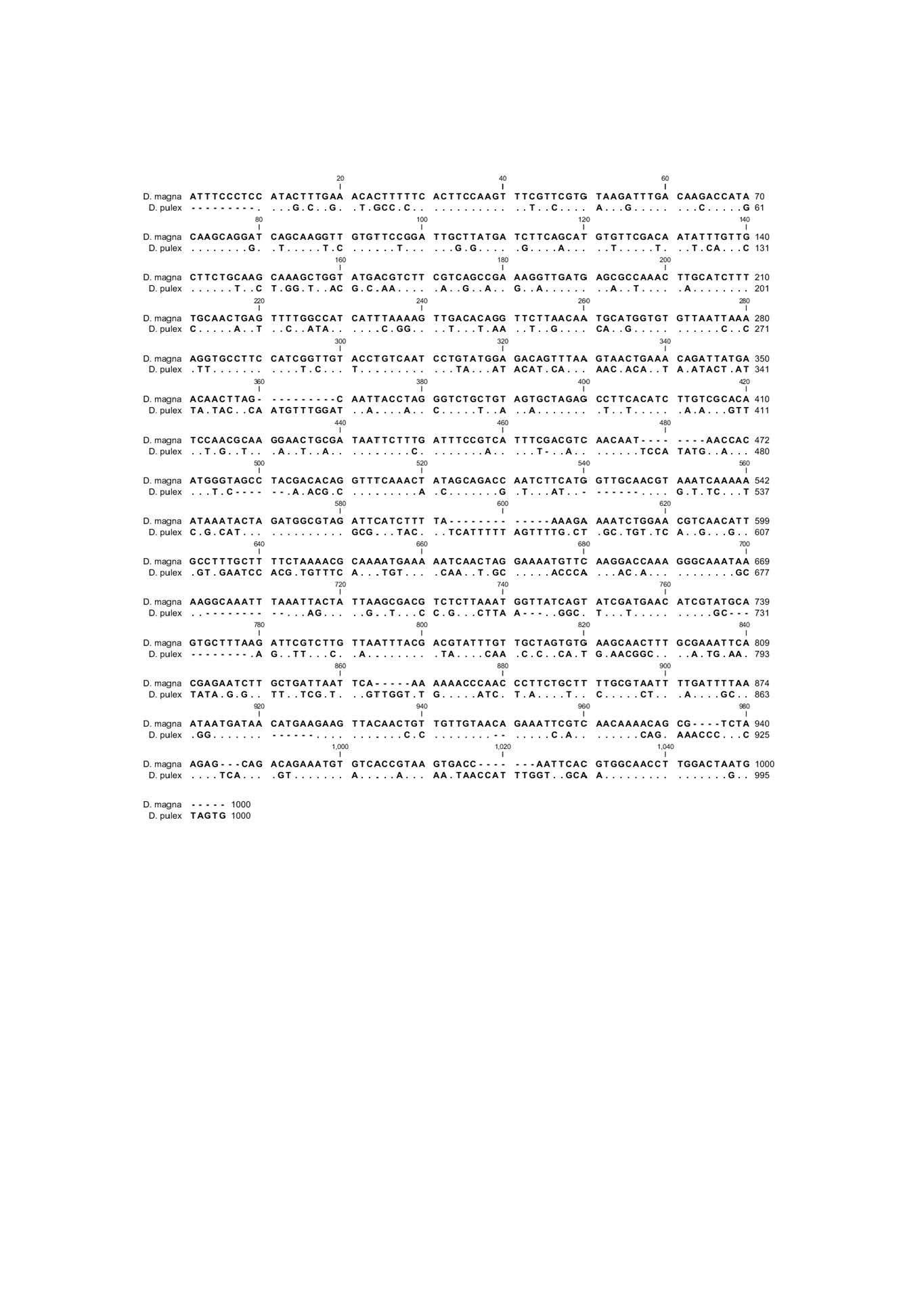

Supplement: Additional file 6 — Nucleotide sequence comparison of dsx1-β promoter regions in D. magna and D. pulex. Pro-Coffee alignment of dsx1-β 1.0 Kbp upstream promoter region from D. magna and D. pulex. [file 1471-2164-14-239-S6.doc]

Supplemental Material 7.


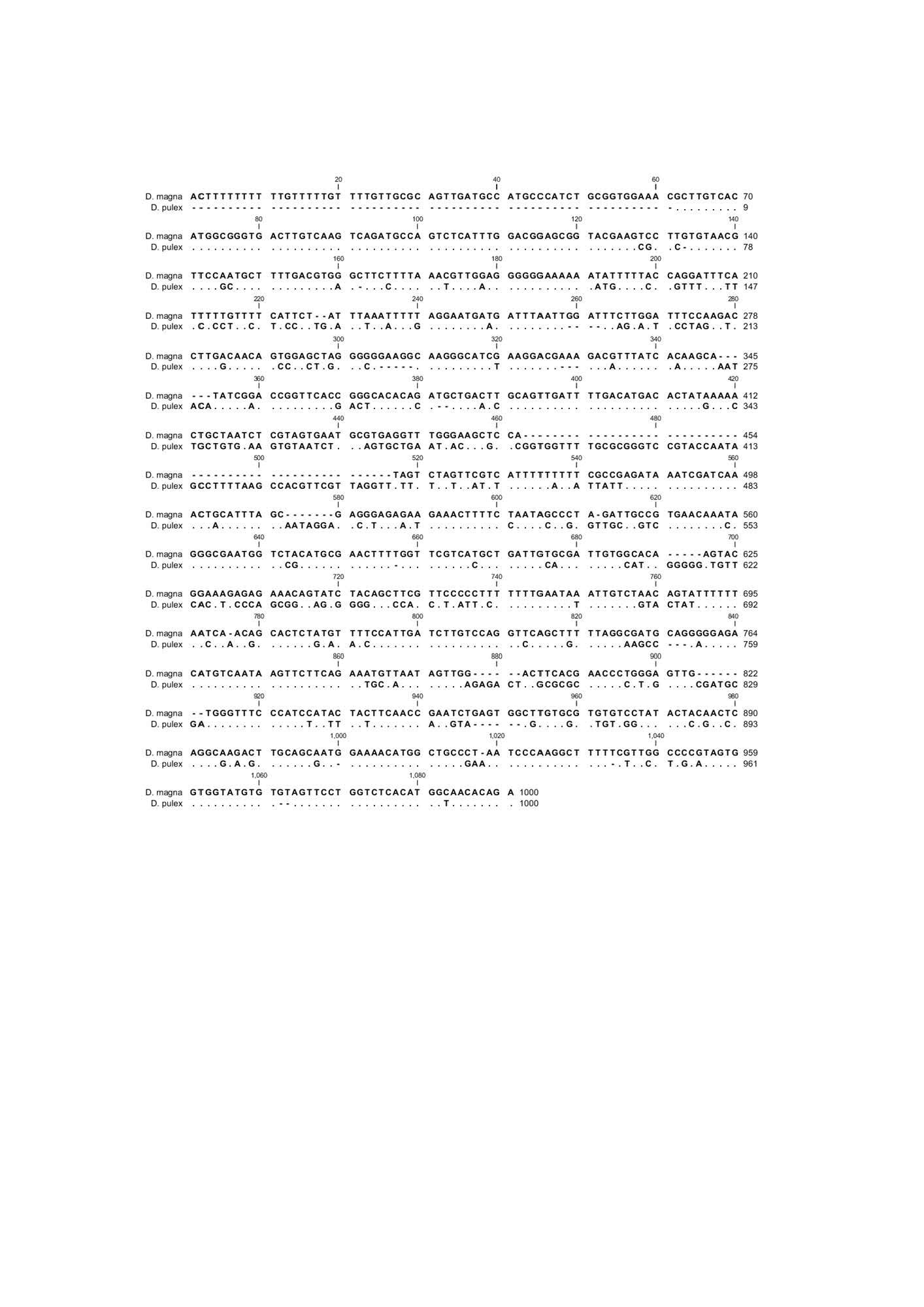

Supplement: Additional file 7 — Nucleotide sequence comparison of dsx2 promoter regions in D. magna and D. pulex. Pro-Coffee alignment of dsx2 1.0 Kbp upstream promoter region from D. magna and D. pulex. [file 1471-2164-14-239-S7.doc]
