# Supplementary material for: Molecular cloning of doublesex genes of four cladocera (water flea) species
Source: BMC Genomics. 2013 Apr 10;14:239. doi: 10.1186/1471-2164-14-239 (PMC3637828; doi:10.1186/1471-2164-14-239)
Supplement: Additional file 8 — dsx1-α TF-map alignment. [file 1471-2164-14-239-S8.doc]

Supplemental Material 8. *dsx1-α* TF-map alignment

| # meta_v1.1 parameters | |  |  |  |  |
| --- | --- | --- | --- | --- | --- |
| # date Fri Apr 6 05:12:38 2012 | |  |  |  |  |
| # MAP1 Dpulex_dsx1-a - Length = 396 elems | | |  |  |  |
| # MAP2 Dmagna_dsx1-a - Length = 498 elems | | |  |  |  |
| # ALPHA = 0.50, LAMBDA = 0.10, MU = 0.10 | | |  |  |  |
| # Maximum similarity: -76.00 | |  |  |  |  |
| # SimMatrix: 2903 matches / 197208 positions (1.47 %) | | | |  |  |
| ### Best meta-alignment contains 20 elements | | |  |  |  |
|  |  |  |  |  |  |
| Column Descriptions |  |  |  |  |  |
| Sequence ID | Promoter region ID - Species, dsx paralog number, and dsx transcript identifier | | | | |
| Source | Name of program that generated results | | | |  |
| Type (TF) | Name of transcription factor identified | | | |  |
| Start | Start of transcription factor binding site (TFBS) | | | | |
| End | End of transcription factor binding site (TFBS) | | | | |
| Score | Match score between known TFBS (from TFBS database) and identified Daphnia dsx promoter sequence motif | | | | |
|  |  |  |  |  |  |
| **Sequence ID** | **Source** | **Type (TF)** | **Start** | **End** | **Score** |
| Dpulex_dsx1-a | meta_v1.1 | lbe | 81 | 86 | 0.94 |
| Dmagna_dsx1-a | meta_v1.1 | lbe | 83 | 88 | 1 |
| Dpulex_dsx1-a | meta_v1.1 | ara | 109 | 113 | 0.89 |
| Dmagna_dsx1-a | meta_v1.1 | ara | 127 | 131 | 0.91 |
| Dpulex_dsx1-a | meta_v1.1 | ara | 153 | 157 | 1 |
| Dmagna_dsx1-a | meta_v1.1 | ara | 184 | 188 | 0.89 |
| Dpulex_dsx1-a | meta_v1.1 | ara | 349 | 353 | 0.99 |
| Dmagna_dsx1-a | meta_v1.1 | ara | 384 | 388 | 0.93 |
| Dpulex_dsx1-a | meta_v1.1 | ct | 403 | 408 | 0.9 |
| Dmagna_dsx1-a | meta_v1.1 | ct | 437 | 442 | 0.89 |
| Dpulex_dsx1-a | meta_v1.1 | Six4 | 446 | 451 | 0.92 |
| Dmagna_dsx1-a | meta_v1.1 | Six4 | 480 | 485 | 1 |
| Dpulex_dsx1-a | meta_v1.1 | oc | 463 | 468 | 0.86 |
| Dmagna_dsx1-a | meta_v1.1 | oc | 498 | 503 | 0.85 |
| Dpulex_dsx1-a | meta_v1.1 | ara | 469 | 473 | 0.91 |
| Dmagna_dsx1-a | meta_v1.1 | ara | 507 | 511 | 0.91 |
| Dpulex_dsx1-a | meta_v1.1 | CG42234 | 475 | 481 | 0.87 |
| Dmagna_dsx1-a | meta_v1.1 | CG42234 | 514 | 520 | 0.87 |
| Dpulex_dsx1-a | meta_v1.1 | C15 | 484 | 490 | 0.93 |
| Dmagna_dsx1-a | meta_v1.1 | C15 | 526 | 532 | 0.88 |
| Dpulex_dsx1-a | meta_v1.1 | CG4328 | 570 | 576 | 0.87 |
| Dmagna_dsx1-a | meta_v1.1 | CG4328 | 617 | 623 | 0.87 |
| Dpulex_dsx1-a | meta_v1.1 | caup | 593 | 597 | 0.9 |
| Dmagna_dsx1-a | meta_v1.1 | caup | 640 | 644 | 0.85 |
| Dpulex_dsx1-a | meta_v1.1 | ara | 609 | 613 | 0.91 |
| Dmagna_dsx1-a | meta_v1.1 | ara | 656 | 660 | 1 |
| Dpulex_dsx1-a | meta_v1.1 | C15 | 657 | 663 | 0.93 |
| Dmagna_dsx1-a | meta_v1.1 | C15 | 687 | 693 | 0.94 |
| Dpulex_dsx1-a | meta_v1.1 | ara | 712 | 716 | 0.89 |
| Dmagna_dsx1-a | meta_v1.1 | ara | 743 | 747 | 0.91 |
| Dpulex_dsx1-a | meta_v1.1 | ara | 735 | 739 | 0.91 |
| Dmagna_dsx1-a | meta_v1.1 | ara | 762 | 766 | 0.91 |
| Dpulex_dsx1-a | meta_v1.1 | ara | 790 | 794 | 1 |
| Dmagna_dsx1-a | meta_v1.1 | ara | 818 | 822 | 0.91 |
| Dpulex_dsx1-a | meta_v1.1 | Eip74EF | 818 | 824 | 1 |
| Dmagna_dsx1-a | meta_v1.1 | Eip74EF | 847 | 853 | 1 |
| Dpulex_dsx1-a | meta_v1.1 | br_Z2 | 888 | 895 | 0.91 |
| Dmagna_dsx1-a | meta_v1.1 | br_Z2 | 907 | 914 | 0.86 |
| Dpulex_dsx1-a | meta_v1.1 | mirr | 965 | 969 | 0.89 |
| Dmagna_dsx1-a | meta_v1.1 | mirr | 967 | 971 | 1 |
