# Supplementary material for: Molecular cloning of doublesex genes of four cladocera (water flea) species
Source: BMC Genomics. 2013 Apr 10;14:239. doi: 10.1186/1471-2164-14-239 (PMC3637828; doi:10.1186/1471-2164-14-239)
Supplement: Additional file 9 — dsx1-β TF-map alignments. [file 1471-2164-14-239-S9.doc]

Supplemental Material 9. *dsx1-β* TF-map alignments

| # meta_v1.1 paramters |  |  |  |  |  |
| --- | --- | --- | --- | --- | --- |
| # date Fri Apr 6 05:12:38 2012 | |  |  |  |  |
| # MAP1 Dpulex_dsx1-b - Length = 441 elems | | |  |  |  |
| # MAP2 Dmagna_dsx1-b - Length = 623 elems | | |  |  |  |
| # ALPHA = 0.50, LAMBDA = 0.10, MU = 0.10 | | |  |  |  |
| # Maximum similarity: -79.73 | |  |  |  |  |
| # SimMatrix: 4962 matches / 274743 positions (1.81 %) | | | |  |  |
| ### Best meta-alignment contains 31 elements | | |  |  |  |
|  |  |  |  |  |  |
| Column Descriptions |  |  |  |  |  |
| Sequence ID | Promoter region ID - Species, dsx paralog number, and dsx transcript identifier | | | | |
| Source | Name of program that generated results | | | |  |
| Type (TF) | Name of transcription factor identified | | | |  |
| Start | Start of transcription factor binding site (TFBS) | | | | |
| End | End of transcription factor binding site (TFBS) | | | | |
| Score | Match score between known TFBS (from TFBS database) and identified Daphnia dsx promoter sequence motif | | | | |
|  |  |  |  |  |  |
| **Sequence ID** | **Source** | **Type (TF)** | **Start** | **End** | **Score** |
| Dpulex_dsx1-b | meta_v1.1 | exd | 46 | 53 | 0.99 |
| Dmagna_dsx1-b | meta_v1.1 | exd | 55 | 62 | 0.99 |
| Dpulex_dsx1-b | meta_v1.1 | ara | 83 | 87 | 0.93 |
| Dmagna_dsx1-b | meta_v1.1 | ara | 92 | 96 | 0.93 |
| Dpulex_dsx1-b | meta_v1.1 | ara | 111 | 115 | 0.99 |
| Dmagna_dsx1-b | meta_v1.1 | ara | 122 | 126 | 0.93 |
| Dpulex_dsx1-b | meta_v1.1 | CG4328 | 119 | 125 | 0.87 |
| Dmagna_dsx1-b | meta_v1.1 | CG4328 | 129 | 135 | 0.89 |
| Dpulex_dsx1-b | meta_v1.1 | CG42234 | 176 | 182 | 0.91 |
| Dmagna_dsx1-b | meta_v1.1 | CG42234 | 185 | 191 | 0.91 |
| Dpulex_dsx1-b | meta_v1.1 | ems | 217 | 223 | 0.86 |
| Dmagna_dsx1-b | meta_v1.1 | ems | 230 | 236 | 0.87 |
| Dpulex_dsx1-b | meta_v1.1 | CG11617 | 232 | 238 | 0.94 |
| Dmagna_dsx1-b | meta_v1.1 | CG11617 | 241 | 247 | 0.92 |
| Dpulex_dsx1-b | meta_v1.1 | CG11617 | 245 | 251 | 0.92 |
| Dmagna_dsx1-b | meta_v1.1 | CG11617 | 254 | 260 | 0.99 |
| Dpulex_dsx1-b | meta_v1.1 | ara | 261 | 265 | 1 |
| Dmagna_dsx1-b | meta_v1.1 | ara | 270 | 274 | 1 |
| Dpulex_dsx1-b | meta_v1.1 | hth | 294 | 299 | 1 |
| Dmagna_dsx1-b | meta_v1.1 | hth | 303 | 308 | 1 |
| Dpulex_dsx1-b | meta_v1.1 | ara | 310 | 314 | 0.91 |
| Dmagna_dsx1-b | meta_v1.1 | ara | 313 | 317 | 0.91 |
| Dpulex_dsx1-b | meta_v1.1 | lbe | 331 | 336 | 0.91 |
| Dmagna_dsx1-b | meta_v1.1 | lbe | 328 | 333 | 0.92 |
| Dpulex_dsx1-b | meta_v1.1 | ara | 353 | 357 | 0.99 |
| Dmagna_dsx1-b | meta_v1.1 | ara | 349 | 353 | 0.93 |
| Dpulex_dsx1-b | meta_v1.1 | CG11617 | 394 | 400 | 0.94 |
| Dmagna_dsx1-b | meta_v1.1 | CG11617 | 393 | 399 | 0.89 |
| Dpulex_dsx1-b | meta_v1.1 | PHDP | 431 | 437 | 0.9 |
| Dmagna_dsx1-b | meta_v1.1 | PHDP | 430 | 436 | 0.9 |
| Dpulex_dsx1-b | meta_v1.1 | ara | 460 | 464 | 0.89 |
| Dmagna_dsx1-b | meta_v1.1 | ara | 460 | 464 | 0.89 |
| Dpulex_dsx1-b | meta_v1.1 | Six4 | 495 | 500 | 0.92 |
| Dmagna_dsx1-b | meta_v1.1 | Six4 | 493 | 498 | 0.92 |
| Dpulex_dsx1-b | meta_v1.1 | pan | 530 | 537 | 0.86 |
| Dmagna_dsx1-b | meta_v1.1 | pan | 534 | 541 | 0.93 |
| Dpulex_dsx1-b | meta_v1.1 | caup | 594 | 598 | 0.85 |
| Dmagna_dsx1-b | meta_v1.1 | caup | 593 | 597 | 0.87 |
| Dpulex_dsx1-b | meta_v1.1 | ara | 636 | 640 | 0.99 |
| Dmagna_dsx1-b | meta_v1.1 | ara | 645 | 649 | 0.93 |
| Dpulex_dsx1-b | meta_v1.1 | br_Z2 | 679 | 686 | 0.88 |
| Dmagna_dsx1-b | meta_v1.1 | br_Z2 | 685 | 692 | 0.89 |
| Dpulex_dsx1-b | meta_v1.1 | lbe | 702 | 707 | 0.86 |
| Dmagna_dsx1-b | meta_v1.1 | lbe | 709 | 714 | 0.95 |
| Dpulex_dsx1-b | meta_v1.1 | ct | 719 | 724 | 1 |
| Dmagna_dsx1-b | meta_v1.1 | ct | 724 | 729 | 0.86 |
| Dpulex_dsx1-b | meta_v1.1 | CG11617 | 787 | 793 | 0.94 |
| Dmagna_dsx1-b | meta_v1.1 | CG11617 | 785 | 791 | 0.88 |
| Dpulex_dsx1-b | meta_v1.1 | Deaf1 | 813 | 818 | 0.96 |
| Dmagna_dsx1-b | meta_v1.1 | Deaf1 | 808 | 813 | 0.91 |
| Dpulex_dsx1-b | meta_v1.1 | vvl | 821 | 826 | 0.9 |
| Dmagna_dsx1-b | meta_v1.1 | vvl | 822 | 827 | 0.88 |
| Dpulex_dsx1-b | meta_v1.1 | slbo | 857 | 864 | 1 |
| Dmagna_dsx1-b | meta_v1.1 | slbo | 856 | 863 | 0.85 |
| Dpulex_dsx1-b | meta_v1.1 | lbe | 871 | 876 | 0.86 |
| Dmagna_dsx1-b | meta_v1.1 | lbe | 872 | 877 | 0.91 |
| Dpulex_dsx1-b | meta_v1.1 | ara | 878 | 882 | 0.91 |
| Dmagna_dsx1-b | meta_v1.1 | ara | 882 | 886 | 1 |
| Dpulex_dsx1-b | meta_v1.1 | ara | 891 | 895 | 0.91 |
| Dmagna_dsx1-b | meta_v1.1 | ara | 895 | 899 | 0.91 |
| Dpulex_dsx1-b | meta_v1.1 | mirr | 909 | 913 | 1 |
| Dmagna_dsx1-b | meta_v1.1 | mirr | 910 | 914 | 0.99 |
