# Supplementary material for: Molecular cloning of doublesex genes of four cladocera (water flea) species
Source: BMC Genomics. 2013 Apr 10;14:239. doi: 10.1186/1471-2164-14-239 (PMC3637828; doi:10.1186/1471-2164-14-239)
Supplement: Additional file 10 — dsx2 TF-map alignments. [file 1471-2164-14-239-S10.doc]

Supplemental Material 10. *dsx2* TF-map alignments

| # meta_v1.1 parameters | |  |  |  |  |
| --- | --- | --- | --- | --- | --- |
| # date Fri Apr 6 05:12:38 2012 | |  |  |  |  |
| # MAP1 Dpulex_dsx2 - Length = 324 elems | | |  |  |  |
| # MAP2 Dmagna_dsx2 - Length = 316 elems | | |  |  |  |
| # ALPHA = 0.50, LAMBDA = 0.10, MU = 0.10 | | |  |  |  |
| # Maximum similarity: -37.76 | |  |  |  |  |
| # SimMatrix: 1942 matches / 102384 positions (1.90 %) | | | |  |  |
| ### Best meta-alignment contains 39 elements | | |  |  |  |
|  |  |  |  |  |  |
| Column Descriptions |  |  |  |  |  |
| Sequence ID | Promoter region ID - Species, dsx paralog number, and dsx transcript identifier | | | | |
| Source | Name of program that generated results | | | |  |
| Type (TF) | Name of transcription factor identified | | | |  |
| Start | Start of transcription factor binding site (TFBS) | | | | |
| End | End of transcription factor binding site (TFBS) | | | | |
| Score | Match score between known TFBS (from TFBS database) and identified Daphnia dsx promoter sequence motif | | | | |
|  |  |  |  |  |  |
| **Sequence ID** | **Source** | **Type (TF)** | **Start** | **End** | **Score** |
| Dpulex_dsx2 | meta_v1.1 | vis | 3 | 8 | 0.95 |
| Dmagna_dsx2 | meta_v1.1 | vis | 64 | 69 | 0.95 |
| Dpulex_dsx2 | meta_v1.1 | vis | 22 | 27 | 0.95 |
| Dmagna_dsx2 | meta_v1.1 | vis | 83 | 88 | 0.95 |
| Dpulex_dsx2 | meta_v1.1 | Six4 | 40 | 45 | 0.95 |
| Dmagna_dsx2 | meta_v1.1 | Six4 | 101 | 106 | 0.95 |
| Dpulex_dsx2 | meta_v1.1 | Deaf1 | 60 | 65 | 0.93 |
| Dmagna_dsx2 | meta_v1.1 | Deaf1 | 121 | 126 | 0.93 |
| Dpulex_dsx2 | meta_v1.1 | ara | 72 | 76 | 0.91 |
| Dmagna_dsx2 | meta_v1.1 | ara | 134 | 138 | 0.91 |
| Dpulex_dsx2 | meta_v1.1 | exd | 88 | 95 | 0.95 |
| Dmagna_dsx2 | meta_v1.1 | exd | 150 | 157 | 0.95 |
| Dpulex_dsx2 | meta_v1.1 | B-H1 | 105 | 111 | 0.96 |
| Dmagna_dsx2 | meta_v1.1 | B-H1 | 165 | 171 | 0.86 |
| Dpulex_dsx2 | meta_v1.1 | C15 | 166 | 172 | 0.85 |
| Dmagna_dsx2 | meta_v1.1 | C15 | 226 | 232 | 0.95 |
| Dpulex_dsx2 | meta_v1.1 | sd | 175 | 186 | 0.87 |
| Dmagna_dsx2 | meta_v1.1 | sd | 236 | 247 | 0.86 |
| Dpulex_dsx2 | meta_v1.1 | PHDP | 190 | 196 | 0.96 |
| Dmagna_dsx2 | meta_v1.1 | PHDP | 251 | 257 | 0.96 |
| Dpulex_dsx2 | meta_v1.1 | mirr | 219 | 223 | 0.89 |
| Dmagna_dsx2 | meta_v1.1 | mirr | 284 | 288 | 0.89 |
| Dpulex_dsx2 | meta_v1.1 | H2.0 | 261 | 267 | 0.86 |
| Dmagna_dsx2 | meta_v1.1 | H2.0 | 333 | 339 | 0.9 |
| Dpulex_dsx2 | meta_v1.1 | Optix | 279 | 283 | 0.87 |
| Dmagna_dsx2 | meta_v1.1 | Optix | 346 | 350 | 0.87 |
| Dpulex_dsx2 | meta_v1.1 | ct | 289 | 294 | 0.86 |
| Dmagna_dsx2 | meta_v1.1 | ct | 356 | 361 | 0.86 |
| Dpulex_dsx2 | meta_v1.1 | onecut | 318 | 324 | 1 |
| Dmagna_dsx2 | meta_v1.1 | onecut | 387 | 393 | 1 |
| Dpulex_dsx2 | meta_v1.1 | caup | 325 | 329 | 0.85 |
| Dmagna_dsx2 | meta_v1.1 | caup | 394 | 398 | 0.85 |
| Dpulex_dsx2 | meta_v1.1 | Six4 | 330 | 335 | 0.98 |
| Dmagna_dsx2 | meta_v1.1 | Six4 | 399 | 404 | 0.98 |
| Dpulex_dsx2 | meta_v1.1 | Gsc | 357 | 362 | 0.94 |
| Dmagna_dsx2 | meta_v1.1 | Gsc | 417 | 422 | 0.94 |
| Dpulex_dsx2 | meta_v1.1 | Deaf1 | 429 | 434 | 0.96 |
| Dmagna_dsx2 | meta_v1.1 | Deaf1 | 463 | 468 | 0.98 |
| Dpulex_dsx2 | meta_v1.1 | hb | 440 | 449 | 0.92 |
| Dmagna_dsx2 | meta_v1.1 | hb | 470 | 479 | 0.95 |
| Dpulex_dsx2 | meta_v1.1 | pan | 478 | 485 | 0.95 |
| Dmagna_dsx2 | meta_v1.1 | pan | 493 | 500 | 0.95 |
| Dpulex_dsx2 | meta_v1.1 | B-H1 | 488 | 494 | 0.89 |
| Dmagna_dsx2 | meta_v1.1 | B-H1 | 503 | 509 | 0.89 |
| Dpulex_dsx2 | meta_v1.1 | ara | 545 | 549 | 0.93 |
| Dmagna_dsx2 | meta_v1.1 | ara | 552 | 556 | 0.93 |
| Dpulex_dsx2 | meta_v1.1 | Deaf1 | 555 | 560 | 0.87 |
| Dmagna_dsx2 | meta_v1.1 | Deaf1 | 562 | 567 | 0.87 |
| Dpulex_dsx2 | meta_v1.1 | Deaf1 | 582 | 587 | 0.98 |
| Dmagna_dsx2 | meta_v1.1 | Deaf1 | 590 | 595 | 0.98 |
| Dpulex_dsx2 | meta_v1.1 | onecut | 591 | 597 | 0.87 |
| Dmagna_dsx2 | meta_v1.1 | onecut | 599 | 605 | 0.87 |
| Dpulex_dsx2 | meta_v1.1 | vvl | 666 | 671 | 0.98 |
| Dmagna_dsx2 | meta_v1.1 | vvl | 669 | 674 | 0.98 |
| Dpulex_dsx2 | meta_v1.1 | lbe | 692 | 697 | 0.95 |
| Dmagna_dsx2 | meta_v1.1 | lbe | 695 | 700 | 0.94 |
| Dpulex_dsx2 | meta_v1.1 | ara | 708 | 712 | 0.91 |
| Dmagna_dsx2 | meta_v1.1 | ara | 712 | 716 | 0.99 |
| Dpulex_dsx2 | meta_v1.1 | dTCF | 716 | 726 | 0.91 |
| Dmagna_dsx2 | meta_v1.1 | dTCF | 718 | 728 | 0.91 |
| Dpulex_dsx2 | meta_v1.1 | lbe | 740 | 745 | 0.86 |
| Dmagna_dsx2 | meta_v1.1 | lbe | 742 | 747 | 0.85 |
| Dpulex_dsx2 | meta_v1.1 | hth | 761 | 766 | 0.96 |
| Dmagna_dsx2 | meta_v1.1 | hth | 766 | 771 | 0.96 |
| Dpulex_dsx2 | meta_v1.1 | dl | 832 | 842 | 0.87 |
| Dmagna_dsx2 | meta_v1.1 | dl | 823 | 833 | 0.87 |
| Dpulex_dsx2 | meta_v1.1 | ct | 853 | 858 | 0.89 |
| Dmagna_dsx2 | meta_v1.1 | ct | 844 | 849 | 0.89 |
| Dpulex_dsx2 | meta_v1.1 | mirr | 915 | 919 | 1 |
| Dmagna_dsx2 | meta_v1.1 | mirr | 913 | 917 | 1 |
| Dpulex_dsx2 | meta_v1.1 | Deaf1 | 948 | 953 | 0.98 |
| Dmagna_dsx2 | meta_v1.1 | Deaf1 | 942 | 947 | 0.96 |
| Dpulex_dsx2 | meta_v1.1 | Lag1 | 959 | 965 | 0.88 |
| Dmagna_dsx2 | meta_v1.1 | Lag1 | 957 | 963 | 0.88 |
| Dpulex_dsx2 | meta_v1.1 | Six4 | 981 | 986 | 0.95 |
| Dmagna_dsx2 | meta_v1.1 | Six4 | 981 | 986 | 0.95 |
| Dpulex_dsx2 | meta_v1.1 | ara | 992 | 996 | 1 |
| Dmagna_dsx2 | meta_v1.1 | ara | 992 | 996 | 0.89 |
