# Supplementary material for: Molecular cloning of doublesex genes of four cladocera (water flea) species
Source: BMC Genomics. 2013 Apr 10;14:239. doi: 10.1186/1471-2164-14-239 (PMC3637828; doi:10.1186/1471-2164-14-239)
Supplement: Additional file 11 — TF-map alignments unique TFs comparison. [file 1471-2164-14-239-S11.doc]

Supplemental Material 11. TF-map alignments unique TFs comparison

| **Dsx1-a** | **Dsx1-b** | **Dsx2** | **Total** | **All** | **Dsx1-a & Dsx1-b** | **Dsx1-a & Dsx2** | **Dsx1-b & Dsx2** |
| --- | --- | --- | --- | --- | --- | --- | --- |
| ara | ara | ara | ara | ara | br_Z2 | C15 | Deaf1 |
| br_Z2 | br_Z2 | B-H1 | B-H1 | caup | CG42234 |  | exd |
| C15 | caup | C15 | br_Z2 | ct | CG4328 |  | hth |
| caup | CG11617 | caup | C15 | mirr |  |  | pan |
| CG42234 | CG42234 | ct | caup | lbe |  |  | PHDP |
| CG4328 | CG4328 | Deaf1 | CG11617 | Six4 |  |  | vvl |
| ct | ct | dl | CG42234 |  |  |  |  |
| Eip74EF | Deaf1 | dTCF | CG4328 |  |  |  |  |
| lbe | ems | exd | ct |  |  |  |  |
| mirr | exd | Gsc | Deaf1 |  |  |  |  |
| oc | hth | H2.0 | dl |  |  |  |  |
| Six4 | lbe | hb | dTCF |  |  |  |  |
|  | mirr | hth | Eip74EF |  |  |  |  |
|  | pan | Lag1 | ems |  |  |  |  |
|  | PHDP | lbe | exd |  |  |  |  |
|  | Six4 | mirr | Gsc |  |  |  |  |
|  | slbo | onecut | H2.0 |  |  |  |  |
|  | vvl | Optix | hb |  |  |  |  |
|  |  | pan | hth |  |  |  |  |
|  |  | PHDP | Lag1 |  |  |  |  |
|  |  | sd | lbe |  |  |  |  |
|  |  | Six4 | mirr |  |  |  |  |
|  |  | vis | oc |  |  |  |  |
|  |  | vvl | onecut |  |  |  |  |
|  |  |  | Optix |  |  |  |  |
|  |  |  | pan |  |  |  |  |
|  |  |  | PHDP |  |  |  |  |
|  |  |  | sd |  |  |  |  |
|  |  |  | Six4 |  |  |  |  |
|  |  |  | slbo |  |  |  |  |
|  |  |  | vis |  |  |  |  |
|  |  |  | vvl |  |  |  |  |
