# Supplementary material for: Molecular cloning of doublesex genes of four cladocera (water flea) species
Source: BMC Genomics. 2013 Apr 10;14:239. doi: 10.1186/1471-2164-14-239 (PMC3637828; doi:10.1186/1471-2164-14-239)
Supplement: Additional file 12 — de novo conserved regulatory motifs. [file 1471-2164-14-239-S12.doc]

Supplemental Material 12. *de novo* conserved regulatory motifs

| Motif # |  |  |  |
| --- | --- | --- | --- |
| Seq 1 = DappuDsx1-a |  |  |  |
| Seq 2 = DapmaDsx1-a |  |  |  |
| Seq 3 = DappuDsx1-b |  |  |  |
| Seq 4 = DapmaDsx1-b |  |  |  |
| Seq 5 = DappuDsx2 |  |  |  |
| Seq 6 = DapmaDsx2 |  |  |  |
| Seq 7 = D. mel (only present if motif found in Dmel) | | |  |
|  |  | From TSS | |
| **Motif Sequences** | **X^2^ Score** | **Start** | **Stop** |
| 1 |  |  |  |
| TGCCTTCC | 10.192 | -751 | -744 |
| GGCTTTTA |  | -683 |  |
| TGCCTTCC |  | -726 |  |
| TGCCTTCC |  | -717 |  |
| TGTTTTCC |  | -693 |  |
| TGACTTGC |  | -624 |  |
| TGCTTTCG |  | -417 |  |
| 2 |  |  |  |
| GACGTTTA | 5.1378 | -662 | -655 |
| GGCTTTTA |  | -683 |  |
| GACGTTTC |  | -509 |  |
| CAGGTTTC |  | -511 |  |
| GACATTTA |  | -745 |  |
| GACGTTTA |  | -672 |  |
| GACGTCAA |  | -613 |  |
| 3 |  |  |  |
| AAACATGG | 5.1375 | -136 | -129 |
| GAACAAGG |  | -140 |  |
| AAACAGGA |  | -92 |  |
| AAACAGCG |  | -72 |  |
| AAACATGG |  | -86 |  |
| AAACATGG |  | -88 |  |
| AAACATTT |  | -218 |  |
| 4 |  |  |  |
| TATATTCA | 4.5179 | -264 | -257 |
| TATATACA |  | -242 |  |
| TATATTCA |  | -246 |  |
| TTAATTTA |  | -241 |  |
| TTTAACCA |  | -311 |  |
| TTTAATCA |  | -308 |  |
| CATATTAA |  | -255 |  |
| 5 |  |  |  |
| TGTTACTA | 4.3927 | -211 | -204 |
| TGCTACTA |  | -176 |  |
| TGTAAATA |  | -213 |  |
| TGTTGCTA |  | -224 |  |
| TGTCAATA |  | -239 |  |
| TGTCAATA |  | -234 |  |
| TGATATTA |  | -168 |  |
| 6 |  |  |  |
| ATCGATCA | 4.3504 | -324 | -317 |
| ATCCATCA |  | -322 |  |
| TTCGTTGA |  | -286 |  |
| ATCGATGA |  | -281 |  |
| ATCGATCA |  | -526 |  |
| ATCGATCA |  | -511 |  |
| ATCGCTCA |  | -531 |  |
| 7 |  |  |  |
| TAATCTCG | 4.1444 | -545 | -538 |
| TAATCTTT |  | -503 |  |
| ACATTTTA |  | -552 |  |
| TCATTTCG |  | -553 |  |
| TAATCTTG |  | -644 |  |
| TAATCTCG |  | -584 |  |
| CACTCTCG |  | -463 |  |
| 8 |  |  |  |
| CTTAGATT | 4.0943 | -447 | -440 |
| CGTAGATT |  | -437 |  |
| CTTCGATT |  | -564 |  |
| CTTTGATT |  | -565 |  |
| CTTTGGTT |  | -425 |  |
| CTTTTGGT |  | -418 |  |
| CTTCGATG |  | -507 |  |
| 9 |  |  |  |
| CATCTTTT | 3.8198 | -774 | -767 |
| AATCTTGG |  | -712 |  |
| CATCTTTC |  | -806 |  |
| CATCTTTT |  | -797 |  |
| GTTCCTTT |  | -902 |  |
| CTTCTTTT |  | -839 |  |
| TATCTTTT |  | -949 |  |
| 10 |  |  |  |
| TTCTAAAA | 3.3795 | -441 | -434 |
| TTCTAAAA |  | -431 |  |
| TTTCAAAA |  | -376 |  |
| TTCTAAAA |  | -391 |  |
| TTCCAATA |  | -480 |  |
| TTCTAATA |  | -472 |  |
| 11 |  |  |  |
| GGCTTTTT | 3.3698 | -86 | -79 |
| AGCTTTTT |  | -84 |  |
| GTCTTTTA |  | -153 |  |
| TGCTTTTG |  | -151 |  |
| GGCTTTTC |  | -62 |  |
| GGCTTTTT |  | -65 |  |
| 12 |  |  |  |
| GATCAAAT | 3.2212 | -524 | -517 |
| GATCAAAT |  | -486 |  |
| TTTCAAAT |  | -471 |  |
| AATCAAAA |  | -467 |  |
| GATCAAAC |  | -523 |  |
| GATCAAAC |  | -508 |  |
| 13 |  |  |  |
| TTCGTGAT | 3.1028 | -924 | -917 |
| TTGGTGAT |  | -863 |  |
| TTCTGGAT |  | -916 |  |
| TTCCGGAT |  | -907 |  |
| TTCCGCTG |  | -921 |  |
| TTCCAATG |  | -859 |  |
| 14 |  |  |  |
| TCTGCATG | 3.0511 | -493 | -486 |
| TCTCCCAG |  | -453 |  |
| TCTATATA |  | -483 |  |
| TCTTCATG |  | -486 |  |
| TCCGCATG |  | -437 |  |
| TCTACATG |  | -430 |  |
