# Supplementary material for: Molecular cloning of doublesex genes of four cladocera (water flea) species
Source: BMC Genomics. 2013 Apr 10;14:239. doi: 10.1186/1471-2164-14-239 (PMC3637828; doi:10.1186/1471-2164-14-239)
Supplement: Additional file 13 — Primer sequences for 5’ and 3’ RACE in D. galeata, C. dubia and M. macrocopa. [file 1471-2164-14-239-S13.doc]

Supplemental Material 13. Primer sequences for 5’ and 3’ RACE in *D. galeata*, *C. dubia* and *M. macrocopa*.

|  | First primer (5’ to 3’) | Nested primer (5’ to 3’) |
| --- | --- | --- |
| *D. galeata*-Dsx1 (5'RACE) | GGGCCACTTGCTCCGCATTTATCTTTCG | GTGCCCTTTGAGCGTGCTTATCGTTTG |
| *D. galeata*-Dsx1 (3'RACE) | GCACGCTCAAAGGGCACAAAAGATACTG | GATAAATGCGGAGCAAGTGGCCCTGAG |
| *D. galeata*-Dsx2 (5'RACE) | GACACAAGTCACAACTGCACCGCCCAAA | CTGCACCGCCCAAATGGACAGTAACG |
| *D. galeata*-Dsx2 (3'RACE) | CCATTTGGGCGGTGCAGTTGTGACTTG | GGGCGGTGCAGTTGTGACTTGTGTCGAG |
| *C. dubia*-Dsx1 (5'RACE) | AGACACAGTTTCGCCAGGGACAGT | CCGTAGCACAGTTCACAGACACAGTT |
| *C. dubia*-Dsx1 (3'RACE) | GGTGGCAATGTTTGCTGAATTGGGGA | TGGAAACGAGACGAAGGAACGATGAG |
| *C. dubia*-Dsx2 (5'RACE) | TTTTTCTGCTTCTTGCGAGTGACCCTG | AGCGGCCAAATGGACAGTATCGCTT |
| *C. dubia*-Dsx2 (3'RACE) | GCGCCCTAGCATCCAGAACGTTATT | CCACTGTCTACATTCATTTTCCCTCC |
| *M. macrocopa*-Dsx (5'RACE) | CCTGCTCGGCATTGATCTTGCGCTTC |  |
| *M. macrocopa*-Dsx (3'RACE) | ACCATCAGCACGCTCAAAGGCCACAA |  |
