# Supplementary material for: Molecular cloning of doublesex genes of four cladocera (water flea) species
Source: BMC Genomics. 2013 Apr 10;14:239. doi: 10.1186/1471-2164-14-239 (PMC3637828; doi:10.1186/1471-2164-14-239)
Supplement: Additional file 14 — Accession numbers of the sequences for phylogenetic analysis. [file 1471-2164-14-239-S14.doc]

Supplemental Material 14. Accession numbers of the sequences for phylogenetic analysis

| Organism | Gene name in phylogenetic tree (definition in NCBI) | Accession No. |
| --- | --- | --- |
| Human (Homo sapiens) | DMRT1 (doublesex and mab-3-related transcription factor 1b) | AAR89619 |
|  | DMRT2 (doublesex and mab-3-related transcription factor 2 isoform 1) | NP_006548 |
|  | DMRT3 (doublesex and mab-3-related transcription factor 3) | NP_067063 |
|  | DMRT4 (DMRT-like family A1) | NP_071443 |
|  | DMRT5 (predicted: similar to doublesex and mab-3-related transcription factor-like family A2) | XP_946699 |
| African clawed frog (Xenopus laevis) | DMRT1 (doublesex- and mab-3-related transcription factor 1) | Q3LH63 |
|  | DMRT4 (DMRT4) | AAV66322 |
|  | DMRT5 (doublesex and mab-3-related transcription factor 5) | ABC55871 |
| Japanese medaka (Oryzias latipes) | DMY (DMY protein) | BAB92012 |
|  | DMRT2 (DMRT2) | AAL02163 |
|  | DMRT4 (OlaDMRT4) | BAB63259 |
|  | DMRT5 (doublesex and mab-3-related transcription factor 5) | BAD00703 |
| Pufferfish (Takifugu rubripes) | DMRT1 (doublesex and mab-3-related transcription factor 1) | NP_001033038 |
|  | DMRT2 (doublesex and mab-3-related transcription factor 2) | NP_001033035 |
|  | DMRT3 (doublesex and mab-3-related transcription factor 3) | NP_001033034 |
|  | DMRT4 (doublesex and mab-3-related transcription factor 4) | NP_001033037 |
|  | DMRT5 (doublesex and mab-3-related transcription factor 5) | NP_001033039 |
| Zebrafish (Danio rerio) | DMRT3 (doublesex and mab-3-related transcription factor 3) | AAU89440 |
| Honeybee (Apis mellifera) | DMRT93B (predicted: similar to CG5737-PA) | XP_392966 |
|  | doublesex | NP_001104725 |
| Beetle (Tribolium castaneum) | DMRT93B (predicted: similar to CG5737-PA) | XP_971604 |
|  | DMRT99B (predicted: similar to CG15504-PA) | XP_975675 |
|  | PREDICTED: similar to CG11094-PB, isoform B | XP_971776 |
| Silkworm (Bombyx mori) | doublesex isoform M | NP_001104815 |
| African malaria mosquito (Anopheles gambiae) | DMRT93B (ENSANGP00000016774) | XP_321748 |
|  | DMRT99B (ENSANGP00000020063) | XP_310668 |
|  | AGAP004050-PC | XP_560052 |
| Yellow fever mosquito (Aedes aegypti) | DMRT93B (conserved hypothetical protein) | EAT43900 |
|  | male specific 1 protein | ABD96573 |
| Fruit fly (Drosophila melanogaster) | DMRT11E (doublesex-Mab-related 11E CG15749-PA) | NP_511146 |
|  | DMRT93B (doublesex-Mab-related 93B CG5737-PA) | NP_524428 |
|  | DMRT99B (doublesex-Mab-related 99B CG15504-PA) | NP_524549 |
|  | doublesex CG11094-PA, isoform A | NP_731197 |
| Fruit fly (Drosophila pseudoobscura) | DMRT11E (GA13932-PA) | XP_001355530 |
|  | DMRT93B(GA19095-PA) | XP_001360059 |
|  | DMRT99B(GA13771-PA) | XP_001357766 |
| House fly (*Musca domestica*) | DSXM | AAR23813 |
| Fruit fly (*Anastrepha obliqua*) | doublesexM | AAY25167 |
| Fruit fly (*Bactrocera oleae*) | male-specific doublesex protein | CAD67987 |
| Eriocheir sinensis | Dmrt-like protein | ADH15934 |
| Penaeus monodon | testis-specific DMRT1 | ACC94178 |
| Daphnia magna | doublesex-Mab related 99B | BAG12873 |
|  | doublesex-Mab related 11E | BAG12871 |
|  | doublesex-Mab related 93B | BAG12872 |
|  | doublesex1-a | AB569296 |
|  | doublesex1-b | AB569297 |
|  | doublesex2 | AB569298 |
| Daphnia pulex | DMRT-99B-like protein | EFX84867 |
|  | Doublesex and mab-3 related transcription factor 3 | EFX89054 |
|  | DM DNA-binding protein | EFX74782 |
|  | doublesex1 | AB693158 |
|  | doublesex2 | AB693159 |
| Daphnia galeata | doublesex1 | AB693160 |
|  | doublesex2 | AB693161 |
| Ceriodaphnia dubia | doublesex1 | AB693162 |
|  | doublesex2 | AB693163 |
| Moina macrocopa | doublesex | AB693164 |
