# Supplementary material for: Molecular cloning of doublesex genes of four cladocera (water flea) species
Source: BMC Genomics. 2013 Apr 10;14:239. doi: 10.1186/1471-2164-14-239 (PMC3637828; doi:10.1186/1471-2164-14-239)
Supplement: Additional file 15 — Primer sequences for quantitative PCR. [file 1471-2164-14-239-S15.doc]

Supplemental Material 15. Primer sequences for quantitative PCR

| Organism | Gene name | Forward primer (5’ to 3’) | Reverse primer (5’ to 3’) |
| --- | --- | --- | --- |
| *D. magna* | *Dsx1* | CCATTCATCATTACCAAATCCCTTC | AAGTTTGGTGTAGGGGAGGATGAG |
| *D. magna* | *Dsx2* | TCTAATGCCAGTGCGAAATCC | CTAAACGCATCTTCCGTCGTC |
| *D. magna* | *RpL32* | GACCAAAGGGTATTGACAACAGA | CCAACTTTTGGCATAAGGTACTG |
| *D. pulex* | *Dsx1* | GCGATGCAGAAGAGTTGTCC | AACGCTGCTCGATCCTTCTT |
| *D. pulex* | *Dsx2* | TCGGAGGAGGCTTTTAGTGTC | CGGATAAACAGGAAGCGAAA |
| *D. pulex* | *RpL32* | TTTCGCGGAGTTGGATTTAC | TTGGGGTCTCTCTTGGGAAT |
| *D. galeata* | *Dsx1* | AGACAGCGACGCAGAAGAGT | TTGAAGTCGAAACGCTGCTC |
| *D. galeata* | *Dsx2* | AGATCAGCTTTGGACGTAGAGC | GAGCCATGTTTTTCCCGAAT |
| *D. galeata* | *RpL32* | TGGCAGAACCATAACCAACC | GCTGAAGAGGAACTGGCGTA |
| *C. dubia* | *Dsx1* | CGTCGTCTTCGGTCAACAAT | CCGGAAAAGAAATCTCAGCA |
| *C. dubia* | *Dsx2* | ATCTCCGTCCTCGCTAAACC | TCGCTCCACCGAAAGAAGTT |
| *C. dubia* | *RpL32* | GACAGTACCTGATGCCCAAGA | GCGGTTCTGCATCATAAGGA |
| *M. macrocopa* | *Dsx* | GCCTCTCGGAGGTTGTCAAA | GTTTAGCGTTAGGCCCGTCT |
| *M. macrocopa* | *RpL32* | AGAGAAACTGGCGCAAACCT | GCTTGGTAGCTTTGGCAGAA |
