# Supplementary material for: Molecular cloning of doublesex genes of four cladocera (water flea) species
Source: BMC Genomics. 2013 Apr 10;14:239. doi: 10.1186/1471-2164-14-239 (PMC3637828; doi:10.1186/1471-2164-14-239)
Supplement: Additional file 16 — DapmaDsx1-α TF-map. [file 1471-2164-14-239-S16.doc]

Supplemental Material 16. *DapmaDsx1-α* TF-map

| Column Descriptions |  |  |  |  |  |  |  |  | |
| --- | --- | --- | --- | --- | --- | --- | --- | --- | --- |
| Sequence ID | Promoter region ID - Species, dsx paralog number, and dsx transcript identifier  Name of program that generated results  Name of transcription factor identified  Start of transcription factor binding site (TFBS)  End of transcription factor binding site (TFBS)  Match score between known TFBS (from TFBS database) and identified Daphnia dsx promoter sequence motif  Strand on which TFBS was identified in sequence  Reading frame for CDS feature types (not used)  Sequence of transcription factor binding motif (from TFBS database) | | | | | | | | |
| Source |
| Type (TF) |
| Start |
| End |
| Score |
| Strand |
| Phase |
| TF Binding Motif |
|  |  |  |  |  |  |  |  |  | |
| **Sequence ID** | **Source** | **Type (TF)** | **Start** | **End** | **Score** | **Strand** | **TF Binding Motif** | |  |
| Dmagna_dsx1-a | MatScan | hth | 19 | 24 | 0.88 | + | # TGACGG | |  |
| Dmagna_dsx1-a | MatScan | CG7056 | 30 | 37 | 0.86 | + | # TTTCATTA | |  |
| Dmagna_dsx1-a | MatScan | Ubx | 30 | 37 | 0.87 | + | # TTTCATTA | |  |
| Dmagna_dsx1-a | MatScan | abd-A | 31 | 37 | 0.85 | + | # TTCATTA | |  |
| Dmagna_dsx1-a | MatScan | al | 31 | 37 | 0.85 | - | # TAATGAA | |  |
| Dmagna_dsx1-a | MatScan | Awh | 31 | 37 | 0.86 | + | # TTCATTA | |  |
| Dmagna_dsx1-a | MatScan | cad | 31 | 37 | 0.86 | + | # TTCATTA | |  |
| Dmagna_dsx1-a | MatScan | CG15696 | 31 | 37 | 0.88 | + | # TTCATTA | |  |
| Dmagna_dsx1-a | MatScan | CG32105 | 31 | 37 | 0.85 | + | # TTCATTA | |  |
| Dmagna_dsx1-a | MatScan | CG42234 | 31 | 37 | 0.91 | + | # TTCATTA | |  |
| Dmagna_dsx1-a | MatScan | CG4328 | 31 | 37 | 0.89 | + | # TTCATTA | |  |
| Dmagna_dsx1-a | MatScan | H2.0 | 31 | 37 | 0.91 | + | # TTCATTA | |  |
| Dmagna_dsx1-a | MatScan | Lim1 | 31 | 37 | 0.85 | + | # TTCATTA | |  |
| Dmagna_dsx1-a | MatScan | Lim3 | 31 | 37 | 0.85 | + | # TTCATTA | |  |
| Dmagna_dsx1-a | MatScan | abd-A | 32 | 38 | 0.95 | - | # TTAATGA | |  |
| Dmagna_dsx1-a | MatScan | Abd-B | 32 | 38 | 0.91 | - | # TTAATGA | |  |
| Dmagna_dsx1-a | MatScan | al | 32 | 38 | 0.85 | + | # TCATTAA | |  |
| Dmagna_dsx1-a | MatScan | Antp | 32 | 38 | 1 | - | # TTAATGA | |  |
| Dmagna_dsx1-a | MatScan | ap | 32 | 38 | 0.91 | - | # TTAATGA | |  |
| Dmagna_dsx1-a | MatScan | Awh | 32 | 38 | 0.9 | - | # TTAATGA | |  |
| Dmagna_dsx1-a | MatScan | bsh | 32 | 38 | 0.97 | - | # TTAATGA | |  |
| Dmagna_dsx1-a | MatScan | btn | 32 | 38 | 1 | - | # TTAATGA | |  |
| Dmagna_dsx1-a | MatScan | C15 | 32 | 38 | 0.96 | - | # TTAATGA | |  |
| Dmagna_dsx1-a | MatScan | CG13424 | 32 | 38 | 0.88 | - | # TTAATGA | |  |
| Dmagna_dsx1-a | MatScan | CG18599 | 32 | 38 | 0.95 | - | # TTAATGA | |  |
| Dmagna_dsx1-a | MatScan | CG32105 | 32 | 38 | 0.85 | - | # TTAATGA | |  |
| Dmagna_dsx1-a | MatScan | CG32532 | 32 | 38 | 0.86 | - | # TTAATGA | |  |
| Dmagna_dsx1-a | MatScan | CG42234 | 32 | 38 | 0.96 | - | # TTAATGA | |  |
| Dmagna_dsx1-a | MatScan | CG4328 | 32 | 38 | 0.85 | - | # TTAATGA | |  |
| Dmagna_dsx1-a | MatScan | Dfd | 32 | 38 | 1 | - | # TTAATGA | |  |
| Dmagna_dsx1-a | MatScan | E5 | 32 | 38 | 0.97 | - | # TTAATGA | |  |
| Dmagna_dsx1-a | MatScan | ems | 32 | 38 | 1 | - | # TTAATGA | |  |
| Dmagna_dsx1-a | MatScan | en | 32 | 38 | 0.86 | - | # TTAATGA | |  |
| Dmagna_dsx1-a | MatScan | eve | 32 | 38 | 0.99 | - | # TTAATGA | |  |
| Dmagna_dsx1-a | MatScan | ftz | 32 | 38 | 1 | - | # TTAATGA | |  |
| Dmagna_dsx1-a | MatScan | H2.0 | 32 | 38 | 0.96 | - | # TTAATGA | |  |
| Dmagna_dsx1-a | MatScan | HGTX | 32 | 38 | 0.94 | - | # TTAATGA | |  |
| Dmagna_dsx1-a | MatScan | ind | 32 | 38 | 0.94 | - | # TTAATGA | |  |
| Dmagna_dsx1-a | MatScan | lab | 32 | 38 | 0.96 | - | # TTAATGA | |  |
| Dmagna_dsx1-a | MatScan | lbe | 32 | 37 | 0.92 | - | # TAATGA | |  |
| Dmagna_dsx1-a | MatScan | lbl | 32 | 37 | 0.96 | - | # TAATGA | |  |
| Dmagna_dsx1-a | MatScan | Lim1 | 32 | 38 | 0.85 | - | # TTAATGA | |  |
| Dmagna_dsx1-a | MatScan | Lim3 | 32 | 38 | 0.91 | - | # TTAATGA | |  |
| Dmagna_dsx1-a | MatScan | NK7.1 | 32 | 38 | 0.88 | - | # TTAATGA | |  |
| Dmagna_dsx1-a | MatScan | Oct | 32 | 39 | 0.95 | - | # GTTAATGA | |  |
| Dmagna_dsx1-a | MatScan | otp | 32 | 38 | 0.91 | - | # TTAATGA | |  |
| Dmagna_dsx1-a | MatScan | pb | 32 | 38 | 0.99 | - | # TTAATGA | |  |
| Dmagna_dsx1-a | MatScan | PHDP | 32 | 38 | 0.85 | - | # TTAATGA | |  |
| Dmagna_dsx1-a | MatScan | Scr | 32 | 38 | 1 | - | # TTAATGA | |  |
| Dmagna_dsx1-a | MatScan | slou | 32 | 38 | 0.93 | - | # TTAATGA | |  |
| Dmagna_dsx1-a | MatScan | tup | 32 | 38 | 0.87 | - | # TTAATGA | |  |
| Dmagna_dsx1-a | MatScan | Ubx | 32 | 39 | 0.89 | - | # GTTAATGA | |  |
| Dmagna_dsx1-a | MatScan | unpg | 32 | 38 | 0.86 | - | # TTAATGA | |  |
| Dmagna_dsx1-a | MatScan | Vsx1 | 32 | 38 | 0.89 | - | # TTAATGA | |  |
| Dmagna_dsx1-a | MatScan | zen | 32 | 38 | 0.96 | - | # TTAATGA | |  |
| Dmagna_dsx1-a | MatScan | zen2 | 32 | 38 | 0.96 | - | # TTAATGA | |  |
| Dmagna_dsx1-a | MatScan | al | 35 | 41 | 0.85 | - | # TAGTTAA | |  |
| Dmagna_dsx1-a | MatScan | C15 | 35 | 41 | 0.91 | + | # TTAACTA | |  |
| Dmagna_dsx1-a | MatScan | lab | 35 | 41 | 0.85 | + | # TTAACTA | |  |
| Dmagna_dsx1-a | MatScan | Lim1 | 35 | 41 | 0.85 | + | # TTAACTA | |  |
| Dmagna_dsx1-a | MatScan | lbe | 36 | 41 | 1 | + | # TAACTA | |  |
| Dmagna_dsx1-a | MatScan | lbl | 36 | 41 | 0.89 | + | # TAACTA | |  |
| Dmagna_dsx1-a | MatScan | ct | 57 | 62 | 0.86 | - | # GTGAAC | |  |
| Dmagna_dsx1-a | MatScan | exd | 62 | 69 | 0.94 | - | # GTTTGACG | |  |
| Dmagna_dsx1-a | MatScan | Deaf1 | 74 | 79 | 0.98 | + | # TTCGTC | |  |
| Dmagna_dsx1-a | MatScan | ems | 78 | 84 | 0.87 | - | # TAAATGA | |  |
| Dmagna_dsx1-a | MatScan | ftz | 78 | 84 | 0.85 | - | # TAAATGA | |  |
| Dmagna_dsx1-a | MatScan | B-H1 | 79 | 85 | 0.89 | - | # CTAAATG | |  |
| Dmagna_dsx1-a | MatScan | al | 82 | 88 | 0.85 | - | # TAACTAA | |  |
| Dmagna_dsx1-a | MatScan | Lim1 | 82 | 88 | 0.85 | + | # TTAGTTA | |  |
| Dmagna_dsx1-a | MatScan | lbe | 83 | 88 | 1 | - | # TAACTA | |  |
| Dmagna_dsx1-a | MatScan | lbl | 83 | 88 | 0.89 | - | # TAACTA | |  |
| Dmagna_dsx1-a | MatScan | vvl | 87 | 92 | 0.9 | + | # TACGCA | |  |
| Dmagna_dsx1-a | MatScan | vnd | 91 | 99 | 0.86 | - | # TTTCACGTG | |  |
| Dmagna_dsx1-a | MatScan | br_Z3 | 108 | 118 | 0.86 | - | # TAAACGAAAAA | |  |
| Dmagna_dsx1-a | MatScan | Deaf1 | 111 | 116 | 0.96 | + | # TTCGTT | |  |
| Dmagna_dsx1-a | MatScan | ct | 114 | 119 | 0.9 | - | # CTAAAC | |  |
| Dmagna_dsx1-a | MatScan | CG11617 | 126 | 132 | 0.93 | - | # TTTACAA | |  |
| Dmagna_dsx1-a | MatScan | ara | 127 | 131 | 0.91 | - | # TTACA | |  |
| Dmagna_dsx1-a | MatScan | caup | 127 | 131 | 0.87 | - | # TTACA | |  |
| Dmagna_dsx1-a | MatScan | mirr | 127 | 131 | 0.88 | - | # TTACA | |  |
| Dmagna_dsx1-a | MatScan | Dfd | 138 | 153 | 0.87 | + | # TTGGTGATTAAAGGGA | |  |
| Dmagna_dsx1-a | MatScan | Bcd | 141 | 148 | 0.91 | + | # GTGATTAA | |  |
| Dmagna_dsx1-a | MatScan | Dll | 141 | 147 | 0.87 | - | # TAATCAC | |  |
| Dmagna_dsx1-a | MatScan | dri | 141 | 150 | 0.87 | + | # GTGATTAAAG | |  |
| Dmagna_dsx1-a | MatScan | onecut | 141 | 147 | 0.86 | + | # GTGATTA | |  |
| Dmagna_dsx1-a | MatScan | abd-A | 142 | 148 | 0.89 | - | # TTAATCA | |  |
| Dmagna_dsx1-a | MatScan | al | 142 | 148 | 0.85 | + | # TGATTAA | |  |
| Dmagna_dsx1-a | MatScan | Antp | 142 | 148 | 0.91 | - | # TTAATCA | |  |
| Dmagna_dsx1-a | MatScan | ap | 142 | 148 | 0.86 | - | # TTAATCA | |  |
| Dmagna_dsx1-a | MatScan | Awh | 142 | 148 | 0.87 | - | # TTAATCA | |  |
| Dmagna_dsx1-a | MatScan | bsh | 142 | 148 | 0.93 | - | # TTAATCA | |  |
| Dmagna_dsx1-a | MatScan | btn | 142 | 148 | 0.89 | - | # TTAATCA | |  |
| Dmagna_dsx1-a | MatScan | C15 | 142 | 148 | 0.93 | - | # TTAATCA | |  |
| Dmagna_dsx1-a | MatScan | CG18599 | 142 | 148 | 0.9 | - | # TTAATCA | |  |
| Dmagna_dsx1-a | MatScan | CG42234 | 142 | 148 | 0.91 | - | # TTAATCA | |  |
| Dmagna_dsx1-a | MatScan | CG7056 | 142 | 149 | 0.88 | - | # TTTAATCA | |  |
| Dmagna_dsx1-a | MatScan | Dfd | 142 | 148 | 0.87 | - | # TTAATCA | |  |
| Dmagna_dsx1-a | MatScan | E5 | 142 | 148 | 0.91 | - | # TTAATCA | |  |
| Dmagna_dsx1-a | MatScan | ems | 142 | 148 | 0.92 | - | # TTAATCA | |  |
| Dmagna_dsx1-a | MatScan | eve | 142 | 148 | 0.92 | - | # TTAATCA | |  |
| Dmagna_dsx1-a | MatScan | ftz | 142 | 148 | 0.92 | - | # TTAATCA | |  |
| Dmagna_dsx1-a | MatScan | Gsc | 142 | 147 | 0.89 | - | # TAATCA | |  |
| Dmagna_dsx1-a | MatScan | H2.0 | 142 | 148 | 0.91 | - | # TTAATCA | |  |
| Dmagna_dsx1-a | MatScan | HGTX | 142 | 148 | 0.89 | - | # TTAATCA | |  |
| Dmagna_dsx1-a | MatScan | ind | 142 | 148 | 0.87 | - | # TTAATCA | |  |
| Dmagna_dsx1-a | MatScan | lab | 142 | 148 | 0.9 | - | # TTAATCA | |  |
| Dmagna_dsx1-a | MatScan | lbe | 142 | 147 | 0.94 | - | # TAATCA | |  |
| Dmagna_dsx1-a | MatScan | lbl | 142 | 147 | 0.92 | - | # TAATCA | |  |
| Dmagna_dsx1-a | MatScan | Lim1 | 142 | 148 | 0.85 | - | # TTAATCA | |  |
| Dmagna_dsx1-a | MatScan | Lim3 | 142 | 148 | 0.89 | - | # TTAATCA | |  |
| Dmagna_dsx1-a | MatScan | Oct | 142 | 149 | 0.94 | - | # TTTAATCA | |  |
| Dmagna_dsx1-a | MatScan | otp | 142 | 148 | 0.88 | - | # TTAATCA | |  |
| Dmagna_dsx1-a | MatScan | pb | 142 | 148 | 0.91 | - | # TTAATCA | |  |
| Dmagna_dsx1-a | MatScan | Ptx1 | 142 | 148 | 0.86 | - | # TTAATCA | |  |
| Dmagna_dsx1-a | MatScan | Scr | 142 | 148 | 0.88 | - | # TTAATCA | |  |
| Dmagna_dsx1-a | MatScan | slou | 142 | 148 | 0.89 | - | # TTAATCA | |  |
| Dmagna_dsx1-a | MatScan | Ubx | 142 | 149 | 0.89 | - | # TTTAATCA | |  |
| Dmagna_dsx1-a | MatScan | Vsx1 | 142 | 148 | 0.87 | - | # TTAATCA | |  |
| Dmagna_dsx1-a | MatScan | vvl | 142 | 147 | 0.88 | - | # TAATCA | |  |
| Dmagna_dsx1-a | MatScan | zen2 | 142 | 148 | 0.89 | - | # TTAATCA | |  |
| Dmagna_dsx1-a | MatScan | pan | 143 | 150 | 0.85 | - | # CTTTAATC | |  |
| Dmagna_dsx1-a | MatScan | B-H1 | 145 | 151 | 0.85 | + | # TTAAAGG | |  |
| Dmagna_dsx1-a | MatScan | C15 | 145 | 151 | 0.9 | + | # TTAAAGG | |  |
| Dmagna_dsx1-a | MatScan | Trl | 150 | 159 | 0.9 | - | # TCTCTCTCCC | |  |
| Dmagna_dsx1-a | MatScan | Trl | 152 | 161 | 0.98 | - | # TTTCTCTCTC | |  |
| Dmagna_dsx1-a | MatScan | dl | 155 | 165 | 0.86 | + | # AGAGAAAAAAG | |  |
| Dmagna_dsx1-a | MatScan | vvl | 172 | 177 | 0.88 | + | # TACTCA | |  |
| Dmagna_dsx1-a | MatScan | ara | 184 | 188 | 0.89 | + | # CAACA | |  |
| Dmagna_dsx1-a | MatScan | caup | 184 | 188 | 0.87 | + | # CAACA | |  |
| Dmagna_dsx1-a | MatScan | mirr | 184 | 188 | 0.89 | + | # CAACA | |  |
| Dmagna_dsx1-a | MatScan | Dfd | 207 | 222 | 0.85 | + | # TGGGTTATTAACACGA | |  |
| Dmagna_dsx1-a | MatScan | cad | 210 | 216 | 0.87 | + | # GTTATTA | |  |
| Dmagna_dsx1-a | MatScan | CG4328 | 210 | 216 | 0.93 | + | # GTTATTA | |  |
| Dmagna_dsx1-a | MatScan | Dll | 210 | 216 | 0.89 | - | # TAATAAC | |  |
| Dmagna_dsx1-a | MatScan | H2.0 | 210 | 216 | 0.9 | + | # GTTATTA | |  |
| Dmagna_dsx1-a | MatScan | abd-A | 211 | 217 | 0.89 | - | # TTAATAA | |  |
| Dmagna_dsx1-a | MatScan | al | 211 | 217 | 0.85 | + | # TTATTAA | |  |
| Dmagna_dsx1-a | MatScan | Antp | 211 | 217 | 0.91 | - | # TTAATAA | |  |
| Dmagna_dsx1-a | MatScan | ap | 211 | 217 | 0.86 | - | # TTAATAA | |  |
| Dmagna_dsx1-a | MatScan | Awh | 211 | 217 | 0.87 | - | # TTAATAA | |  |
| Dmagna_dsx1-a | MatScan | bsh | 211 | 217 | 0.9 | - | # TTAATAA | |  |
| Dmagna_dsx1-a | MatScan | btn | 211 | 217 | 0.89 | - | # TTAATAA | |  |
| Dmagna_dsx1-a | MatScan | C15 | 211 | 217 | 0.9 | - | # TTAATAA | |  |
| Dmagna_dsx1-a | MatScan | CG13424 | 211 | 217 | 0.87 | - | # TTAATAA | |  |
| Dmagna_dsx1-a | MatScan | CG18599 | 211 | 217 | 0.89 | - | # TTAATAA | |  |
| Dmagna_dsx1-a | MatScan | CG42234 | 211 | 217 | 0.89 | - | # TTAATAA | |  |
| Dmagna_dsx1-a | MatScan | Dfd | 211 | 217 | 0.88 | - | # TTAATAA | |  |
| Dmagna_dsx1-a | MatScan | E5 | 211 | 217 | 0.93 | - | # TTAATAA | |  |
| Dmagna_dsx1-a | MatScan | ems | 211 | 217 | 0.91 | - | # TTAATAA | |  |
| Dmagna_dsx1-a | MatScan | eve | 211 | 217 | 0.9 | - | # TTAATAA | |  |
| Dmagna_dsx1-a | MatScan | ftz | 211 | 217 | 0.92 | - | # TTAATAA | |  |
| Dmagna_dsx1-a | MatScan | H2.0 | 211 | 217 | 0.96 | - | # TTAATAA | |  |
| Dmagna_dsx1-a | MatScan | HGTX | 211 | 217 | 0.9 | - | # TTAATAA | |  |
| Dmagna_dsx1-a | MatScan | ind | 211 | 217 | 0.87 | - | # TTAATAA | |  |
| Dmagna_dsx1-a | MatScan | lab | 211 | 217 | 0.9 | - | # TTAATAA | |  |
| Dmagna_dsx1-a | MatScan | lbe | 211 | 216 | 0.93 | - | # TAATAA | |  |
| Dmagna_dsx1-a | MatScan | lbl | 211 | 216 | 0.9 | - | # TAATAA | |  |
| Dmagna_dsx1-a | MatScan | Lim1 | 211 | 217 | 0.85 | - | # TTAATAA | |  |
| Dmagna_dsx1-a | MatScan | Lim3 | 211 | 217 | 0.87 | - | # TTAATAA | |  |
| Dmagna_dsx1-a | MatScan | NK7.1 | 211 | 217 | 0.87 | - | # TTAATAA | |  |
| Dmagna_dsx1-a | MatScan | Oct | 211 | 218 | 0.91 | - | # GTTAATAA | |  |
| Dmagna_dsx1-a | MatScan | otp | 211 | 217 | 0.87 | - | # TTAATAA | |  |
| Dmagna_dsx1-a | MatScan | pb | 211 | 217 | 0.91 | - | # TTAATAA | |  |
| Dmagna_dsx1-a | MatScan | PHDP | 211 | 217 | 0.85 | - | # TTAATAA | |  |
| Dmagna_dsx1-a | MatScan | Scr | 211 | 217 | 0.88 | - | # TTAATAA | |  |
| Dmagna_dsx1-a | MatScan | slou | 211 | 217 | 0.9 | - | # TTAATAA | |  |
| Dmagna_dsx1-a | MatScan | Vsx1 | 211 | 217 | 0.89 | - | # TTAATAA | |  |
| Dmagna_dsx1-a | MatScan | zen2 | 211 | 217 | 0.89 | - | # TTAATAA | |  |
| Dmagna_dsx1-a | MatScan | vvl | 212 | 217 | 0.88 | + | # TATTAA | |  |
| Dmagna_dsx1-a | MatScan | CG11617 | 214 | 220 | 0.99 | + | # TTAACAC | |  |
| Dmagna_dsx1-a | MatScan | ara | 215 | 219 | 1 | + | # TAACA | |  |
| Dmagna_dsx1-a | MatScan | caup | 215 | 219 | 1 | + | # TAACA | |  |
| Dmagna_dsx1-a | MatScan | mirr | 215 | 219 | 0.99 | + | # TAACA | |  |
| Dmagna_dsx1-a | MatScan | Deaf1 | 218 | 223 | 0.91 | - | # CTCGTG | |  |
| Dmagna_dsx1-a | MatScan | Trl | 225 | 234 | 0.87 | - | # TTTCTCTTTC | |  |
| Dmagna_dsx1-a | MatScan | Deaf1 | 262 | 267 | 0.96 | + | # TTCGGT | |  |
| Dmagna_dsx1-a | MatScan | Dfd | 266 | 281 | 0.88 | + | # GTAAAAATTATTGTGA | |  |
| Dmagna_dsx1-a | MatScan | lbe | 267 | 272 | 0.85 | + | # TAAAAA | |  |
| Dmagna_dsx1-a | MatScan | CG4328 | 269 | 275 | 0.87 | + | # AAAATTA | |  |
| Dmagna_dsx1-a | MatScan | Dll | 269 | 275 | 0.91 | - | # TAATTTT | |  |
| Dmagna_dsx1-a | MatScan | CG4328 | 270 | 276 | 0.87 | - | # ATAATTT | |  |
| Dmagna_dsx1-a | MatScan | lbl | 270 | 275 | 0.85 | - | # TAATTT | |  |
| Dmagna_dsx1-a | MatScan | PHDP | 270 | 276 | 0.93 | - | # ATAATTT | |  |
| Dmagna_dsx1-a | MatScan | Pph13 | 270 | 276 | 0.88 | - | # ATAATTT | |  |
| Dmagna_dsx1-a | MatScan | cad | 272 | 278 | 0.87 | + | # ATTATTG | |  |
| Dmagna_dsx1-a | MatScan | CG4328 | 272 | 278 | 0.99 | + | # ATTATTG | |  |
| Dmagna_dsx1-a | MatScan | onecut | 278 | 284 | 0.87 | + | # GTGATTT | |  |
| Dmagna_dsx1-a | MatScan | slbo | 281 | 288 | 0.9 | + | # ATTTCAAA | |  |
| Dmagna_dsx1-a | MatScan | exd | 282 | 289 | 0.89 | - | # TTTTGAAA | |  |
| Dmagna_dsx1-a | MatScan | pan | 282 | 289 | 0.89 | - | # TTTTGAAA | |  |
| Dmagna_dsx1-a | MatScan | Abd-B | 329 | 335 | 0.86 | + | # TTTATTC | |  |
| Dmagna_dsx1-a | MatScan | cad | 329 | 335 | 0.91 | + | # TTTATTC | |  |
| Dmagna_dsx1-a | MatScan | CG42234 | 329 | 335 | 0.85 | + | # TTTATTC | |  |
| Dmagna_dsx1-a | MatScan | CG4328 | 329 | 335 | 0.9 | + | # TTTATTC | |  |
| Dmagna_dsx1-a | MatScan | CG7056 | 330 | 337 | 0.89 | - | # TTGAATAA | |  |
| Dmagna_dsx1-a | MatScan | vvl | 331 | 336 | 0.98 | + | # TATTCA | |  |
| Dmagna_dsx1-a | MatScan | CG15696 | 334 | 340 | 0.87 | + | # TCAATTG | |  |
| Dmagna_dsx1-a | MatScan | CF1 | 374 | 382 | 0.88 | + | # AGGGTCACG | |  |
| Dmagna_dsx1-a | MatScan | CF1 | 374 | 382 | 0.9 | + | # AGGGTCACG | |  |
| Dmagna_dsx1-a | MatScan | usp | 374 | 383 | 0.88 | + | # AGGGTCACGG | |  |
| Dmagna_dsx1-a | MatScan | ara | 384 | 388 | 0.93 | - | # GAACA | |  |
| Dmagna_dsx1-a | MatScan | caup | 384 | 388 | 0.88 | - | # GAACA | |  |
| Dmagna_dsx1-a | MatScan | mirr | 384 | 388 | 0.89 | - | # GAACA | |  |
| Dmagna_dsx1-a | MatScan | Deaf1 | 386 | 391 | 0.96 | + | # TTCGTT | |  |
| Dmagna_dsx1-a | MatScan | TATA | 414 | 428 | 0.86 | - | # CTATAAATATTCACG | |  |
| Dmagna_dsx1-a | MatScan | vvl | 416 | 421 | 0.98 | - | # TATTCA | |  |
| Dmagna_dsx1-a | MatScan | lbe | 420 | 425 | 0.91 | - | # TAAATA | |  |
| Dmagna_dsx1-a | MatScan | Abd-B | 422 | 428 | 0.87 | + | # TTTATAG | |  |
| Dmagna_dsx1-a | MatScan | cad | 422 | 428 | 0.89 | + | # TTTATAG | |  |
| Dmagna_dsx1-a | MatScan | ct | 437 | 442 | 0.89 | - | # TTCAAC | |  |
| Dmagna_dsx1-a | MatScan | vvl | 447 | 452 | 0.9 | + | # TATGAA | |  |
| Dmagna_dsx1-a | MatScan | CG4328 | 453 | 459 | 0.88 | + | # ATGATTG | |  |
| Dmagna_dsx1-a | MatScan | onecut | 453 | 459 | 0.86 | + | # ATGATTG | |  |
| Dmagna_dsx1-a | MatScan | ara | 479 | 483 | 0.91 | - | # ATACA | |  |
| Dmagna_dsx1-a | MatScan | mirr | 479 | 483 | 0.89 | - | # ATACA | |  |
| Dmagna_dsx1-a | MatScan | Six4 | 480 | 485 | 1 | - | # TGATAC | |  |
| Dmagna_dsx1-a | MatScan | so | 480 | 485 | 1 | - | # TGATAC | |  |
| Dmagna_dsx1-a | MatScan | Optix | 481 | 485 | 1 | - | # TGATA | |  |
| Dmagna_dsx1-a | MatScan | Kr | 490 | 500 | 0.87 | + | # GGAAAGGGTAA | |  |
| Dmagna_dsx1-a | MatScan | Dfd | 492 | 507 | 0.89 | - | # AAAAAGATTACCCTTT | |  |
| Dmagna_dsx1-a | MatScan | ttk | 493 | 501 | 0.87 | + | # AAGGGTAAT | |  |
| Dmagna_dsx1-a | MatScan | Gsc | 498 | 503 | 0.94 | + | # TAATCT | |  |
| Dmagna_dsx1-a | MatScan | oc | 498 | 503 | 0.85 | + | # TAATCT | |  |
| Dmagna_dsx1-a | MatScan | ara | 507 | 511 | 0.91 | - | # TTACA | |  |
| Dmagna_dsx1-a | MatScan | caup | 507 | 511 | 0.87 | - | # TTACA | |  |
| Dmagna_dsx1-a | MatScan | mirr | 507 | 511 | 0.88 | - | # TTACA | |  |
| Dmagna_dsx1-a | MatScan | CG42234 | 513 | 519 | 0.87 | + | # TTGATCA | |  |
| Dmagna_dsx1-a | MatScan | CG42234 | 514 | 520 | 0.87 | - | # TTGATCA | |  |
| Dmagna_dsx1-a | MatScan | pan | 515 | 522 | 0.96 | - | # ATTTGATC | |  |
| Dmagna_dsx1-a | MatScan | br_Z3 | 521 | 531 | 0.85 | - | # TAAACAAACAT | |  |
| Dmagna_dsx1-a | MatScan | ara | 522 | 526 | 0.99 | - | # AAACA | |  |
| Dmagna_dsx1-a | MatScan | caup | 522 | 526 | 0.9 | - | # AAACA | |  |
| Dmagna_dsx1-a | MatScan | fkh | 522 | 532 | 0.98 | + | # TGTTTGTTTAA | |  |
| Dmagna_dsx1-a | MatScan | mirr | 522 | 526 | 1 | - | # AAACA | |  |
| Dmagna_dsx1-a | MatScan | slp1 | 525 | 535 | 0.88 | + | # TTGTTTAACCT | |  |
| Dmagna_dsx1-a | MatScan | ara | 526 | 530 | 0.99 | - | # AAACA | |  |
| Dmagna_dsx1-a | MatScan | C15 | 526 | 532 | 0.88 | - | # TTAAACA | |  |
| Dmagna_dsx1-a | MatScan | caup | 526 | 530 | 0.9 | - | # AAACA | |  |
| Dmagna_dsx1-a | MatScan | lbe | 526 | 531 | 0.86 | - | # TAAACA | |  |
| Dmagna_dsx1-a | MatScan | mirr | 526 | 530 | 1 | - | # AAACA | |  |
| Dmagna_dsx1-a | MatScan | ct | 527 | 532 | 0.98 | - | # TTAAAC | |  |
| Dmagna_dsx1-a | MatScan | ct | 528 | 533 | 0.89 | + | # TTTAAC | |  |
| Dmagna_dsx1-a | MatScan | exd | 534 | 541 | 0.88 | + | # CTTTGAGA | |  |
| Dmagna_dsx1-a | MatScan | ara | 597 | 601 | 0.89 | - | # CAACA | |  |
| Dmagna_dsx1-a | MatScan | caup | 597 | 601 | 0.87 | - | # CAACA | |  |
| Dmagna_dsx1-a | MatScan | mirr | 597 | 601 | 0.89 | - | # CAACA | |  |
| Dmagna_dsx1-a | MatScan | ara | 600 | 604 | 0.99 | - | # AAACA | |  |
| Dmagna_dsx1-a | MatScan | caup | 600 | 604 | 0.9 | - | # AAACA | |  |
| Dmagna_dsx1-a | MatScan | mirr | 600 | 604 | 1 | - | # AAACA | |  |
| Dmagna_dsx1-a | MatScan | Lag1 | 606 | 612 | 0.91 | + | # CAACCAA | |  |
| Dmagna_dsx1-a | MatScan | hb | 609 | 618 | 0.94 | + | # CCAAAAAAAA | |  |
| Dmagna_dsx1-a | MatScan | hb | 610 | 619 | 0.92 | + | # CAAAAAAAAA | |  |
| Dmagna_dsx1-a | MatScan | hb | 611 | 620 | 0.88 | + | # AAAAAAAAAA | |  |
| Dmagna_dsx1-a | MatScan | CG4328 | 617 | 623 | 0.87 | + | # AAAATTG | |  |
| Dmagna_dsx1-a | MatScan | ara | 622 | 626 | 0.91 | - | # ATACA | |  |
| Dmagna_dsx1-a | MatScan | mirr | 622 | 626 | 0.89 | - | # ATACA | |  |
| Dmagna_dsx1-a | MatScan | achi | 640 | 645 | 0.92 | + | # TGACAT | |  |
| Dmagna_dsx1-a | MatScan | caup | 640 | 644 | 0.85 | + | # TGACA | |  |
| Dmagna_dsx1-a | MatScan | hth | 640 | 645 | 0.96 | + | # TGACAT | |  |
| Dmagna_dsx1-a | MatScan | vis | 640 | 645 | 0.95 | + | # TGACAT | |  |
| Dmagna_dsx1-a | MatScan | Dfd | 648 | 663 | 0.88 | + | # TTTGTGATTAACATTA | |  |
| Dmagna_dsx1-a | MatScan | Bcd | 651 | 658 | 0.91 | + | # GTGATTAA | |  |
| Dmagna_dsx1-a | MatScan | Dll | 651 | 657 | 0.87 | - | # TAATCAC | |  |
| Dmagna_dsx1-a | MatScan | dri | 651 | 660 | 0.87 | + | # GTGATTAACA | |  |
| Dmagna_dsx1-a | MatScan | onecut | 651 | 657 | 0.86 | + | # GTGATTA | |  |
| Dmagna_dsx1-a | MatScan | abd-A | 652 | 658 | 0.89 | - | # TTAATCA | |  |
| Dmagna_dsx1-a | MatScan | al | 652 | 658 | 0.85 | + | # TGATTAA | |  |
| Dmagna_dsx1-a | MatScan | Antp | 652 | 658 | 0.91 | - | # TTAATCA | |  |
| Dmagna_dsx1-a | MatScan | ap | 652 | 658 | 0.86 | - | # TTAATCA | |  |
| Dmagna_dsx1-a | MatScan | Awh | 652 | 658 | 0.87 | - | # TTAATCA | |  |
| Dmagna_dsx1-a | MatScan | bsh | 652 | 658 | 0.93 | - | # TTAATCA | |  |
| Dmagna_dsx1-a | MatScan | btn | 652 | 658 | 0.89 | - | # TTAATCA | |  |
| Dmagna_dsx1-a | MatScan | C15 | 652 | 658 | 0.93 | - | # TTAATCA | |  |
| Dmagna_dsx1-a | MatScan | CG18599 | 652 | 658 | 0.9 | - | # TTAATCA | |  |
| Dmagna_dsx1-a | MatScan | CG42234 | 652 | 658 | 0.91 | - | # TTAATCA | |  |
| Dmagna_dsx1-a | MatScan | Dfd | 652 | 658 | 0.87 | - | # TTAATCA | |  |
| Dmagna_dsx1-a | MatScan | E5 | 652 | 658 | 0.91 | - | # TTAATCA | |  |
| Dmagna_dsx1-a | MatScan | ems | 652 | 658 | 0.92 | - | # TTAATCA | |  |
| Dmagna_dsx1-a | MatScan | eve | 652 | 658 | 0.92 | - | # TTAATCA | |  |
| Dmagna_dsx1-a | MatScan | ftz | 652 | 658 | 0.92 | - | # TTAATCA | |  |
| Dmagna_dsx1-a | MatScan | Gsc | 652 | 657 | 0.89 | - | # TAATCA | |  |
| Dmagna_dsx1-a | MatScan | H2.0 | 652 | 658 | 0.91 | - | # TTAATCA | |  |
| Dmagna_dsx1-a | MatScan | HGTX | 652 | 658 | 0.89 | - | # TTAATCA | |  |
| Dmagna_dsx1-a | MatScan | ind | 652 | 658 | 0.87 | - | # TTAATCA | |  |
| Dmagna_dsx1-a | MatScan | lab | 652 | 658 | 0.9 | - | # TTAATCA | |  |
| Dmagna_dsx1-a | MatScan | lbe | 652 | 657 | 0.94 | - | # TAATCA | |  |
| Dmagna_dsx1-a | MatScan | lbl | 652 | 657 | 0.92 | - | # TAATCA | |  |
| Dmagna_dsx1-a | MatScan | Lim1 | 652 | 658 | 0.85 | - | # TTAATCA | |  |
| Dmagna_dsx1-a | MatScan | Lim3 | 652 | 658 | 0.89 | - | # TTAATCA | |  |
| Dmagna_dsx1-a | MatScan | Oct | 652 | 659 | 0.92 | - | # GTTAATCA | |  |
| Dmagna_dsx1-a | MatScan | otp | 652 | 658 | 0.88 | - | # TTAATCA | |  |
| Dmagna_dsx1-a | MatScan | pb | 652 | 658 | 0.91 | - | # TTAATCA | |  |
| Dmagna_dsx1-a | MatScan | Ptx1 | 652 | 658 | 0.86 | - | # TTAATCA | |  |
| Dmagna_dsx1-a | MatScan | Scr | 652 | 658 | 0.88 | - | # TTAATCA | |  |
| Dmagna_dsx1-a | MatScan | slou | 652 | 658 | 0.89 | - | # TTAATCA | |  |
| Dmagna_dsx1-a | MatScan | Vsx1 | 652 | 658 | 0.87 | - | # TTAATCA | |  |
| Dmagna_dsx1-a | MatScan | vvl | 652 | 657 | 0.88 | - | # TAATCA | |  |
| Dmagna_dsx1-a | MatScan | zen2 | 652 | 658 | 0.89 | - | # TTAATCA | |  |
| Dmagna_dsx1-a | MatScan | Dfd | 654 | 669 | 0.86 | + | # ATTAACATTATTGGCC | |  |
| Dmagna_dsx1-a | MatScan | CG11617 | 655 | 661 | 1 | + | # TTAACAT | |  |
| Dmagna_dsx1-a | MatScan | ara | 656 | 660 | 1 | + | # TAACA | |  |
| Dmagna_dsx1-a | MatScan | caup | 656 | 660 | 1 | + | # TAACA | |  |
| Dmagna_dsx1-a | MatScan | mirr | 656 | 660 | 0.99 | + | # TAACA | |  |
| Dmagna_dsx1-a | MatScan | cad | 660 | 666 | 0.87 | + | # ATTATTG | |  |
| Dmagna_dsx1-a | MatScan | CG4328 | 660 | 666 | 0.99 | + | # ATTATTG | |  |
| Dmagna_dsx1-a | MatScan | B-H1 | 687 | 693 | 0.96 | - | # TTAAATG | |  |
| Dmagna_dsx1-a | MatScan | B-H2 | 687 | 693 | 0.91 | - | # TTAAATG | |  |
| Dmagna_dsx1-a | MatScan | C15 | 687 | 693 | 0.94 | - | # TTAAATG | |  |
| Dmagna_dsx1-a | MatScan | CG11085 | 687 | 693 | 0.86 | - | # TTAAATG | |  |
| Dmagna_dsx1-a | MatScan | CG34031 | 687 | 693 | 0.87 | - | # TTAAATG | |  |
| Dmagna_dsx1-a | MatScan | Hmx | 687 | 693 | 0.86 | - | # TTAAATG | |  |
| Dmagna_dsx1-a | MatScan | NK7.1 | 687 | 693 | 0.89 | - | # TTAAATG | |  |
| Dmagna_dsx1-a | MatScan | ct | 707 | 712 | 0.86 | + | # GTGAAC | |  |
| Dmagna_dsx1-a | MatScan | ara | 721 | 725 | 0.91 | - | # TTACA | |  |
| Dmagna_dsx1-a | MatScan | caup | 721 | 725 | 0.87 | - | # TTACA | |  |
| Dmagna_dsx1-a | MatScan | CG7056 | 721 | 728 | 0.87 | + | # TGTAATTA | |  |
| Dmagna_dsx1-a | MatScan | inv | 721 | 728 | 0.92 | + | # TGTAATTA | |  |
| Dmagna_dsx1-a | MatScan | mirr | 721 | 725 | 0.88 | - | # TTACA | |  |
| Dmagna_dsx1-a | MatScan | Oct | 721 | 728 | 0.91 | + | # TGTAATTA | |  |
| Dmagna_dsx1-a | MatScan | abd-A | 722 | 728 | 0.87 | + | # GTAATTA | |  |
| Dmagna_dsx1-a | MatScan | al | 722 | 728 | 0.87 | - | # TAATTAC | |  |
| Dmagna_dsx1-a | MatScan | ap | 722 | 728 | 0.91 | + | # GTAATTA | |  |
| Dmagna_dsx1-a | MatScan | Awh | 722 | 728 | 0.91 | + | # GTAATTA | |  |
| Dmagna_dsx1-a | MatScan | bsh | 722 | 728 | 0.89 | + | # GTAATTA | |  |
| Dmagna_dsx1-a | MatScan | btn | 722 | 728 | 0.88 | + | # GTAATTA | |  |
| Dmagna_dsx1-a | MatScan | CG11294 | 722 | 728 | 0.88 | + | # GTAATTA | |  |
| Dmagna_dsx1-a | MatScan | CG13424 | 722 | 728 | 0.88 | + | # GTAATTA | |  |
| Dmagna_dsx1-a | MatScan | CG15696 | 722 | 728 | 0.88 | + | # GTAATTA | |  |
| Dmagna_dsx1-a | MatScan | CG18599 | 722 | 728 | 0.94 | + | # GTAATTA | |  |
| Dmagna_dsx1-a | MatScan | CG32105 | 722 | 728 | 0.89 | + | # GTAATTA | |  |
| Dmagna_dsx1-a | MatScan | CG32532 | 722 | 728 | 0.92 | + | # GTAATTA | |  |
| Dmagna_dsx1-a | MatScan | CG4328 | 722 | 728 | 0.91 | + | # GTAATTA | |  |
| Dmagna_dsx1-a | MatScan | CG9876 | 722 | 728 | 0.95 | + | # GTAATTA | |  |
| Dmagna_dsx1-a | MatScan | Dll | 722 | 728 | 1 | - | # TAATTAC | |  |
| Dmagna_dsx1-a | MatScan | Dr | 722 | 728 | 0.86 | + | # GTAATTA | |  |
| Dmagna_dsx1-a | MatScan | E5 | 722 | 728 | 0.95 | + | # GTAATTA | |  |
| Dmagna_dsx1-a | MatScan | ems | 722 | 728 | 0.92 | + | # GTAATTA | |  |
| Dmagna_dsx1-a | MatScan | en | 722 | 728 | 0.93 | + | # GTAATTA | |  |
| Dmagna_dsx1-a | MatScan | eve | 722 | 728 | 0.9 | + | # GTAATTA | |  |
| Dmagna_dsx1-a | MatScan | exex | 722 | 728 | 1 | + | # GTAATTA | |  |
| Dmagna_dsx1-a | MatScan | exex | 722 | 728 | 1 | + | # GTAATTA | |  |
| Dmagna_dsx1-a | MatScan | ftz | 722 | 728 | 0.86 | + | # GTAATTA | |  |
| Dmagna_dsx1-a | MatScan | H2.0 | 722 | 728 | 0.91 | + | # GTAATTA | |  |
| Dmagna_dsx1-a | MatScan | hbn | 722 | 728 | 0.91 | + | # GTAATTA | |  |
| Dmagna_dsx1-a | MatScan | HGTX | 722 | 728 | 0.93 | + | # GTAATTA | |  |
| Dmagna_dsx1-a | MatScan | ind | 722 | 728 | 0.91 | + | # GTAATTA | |  |
| Dmagna_dsx1-a | MatScan | lab | 722 | 728 | 0.88 | + | # GTAATTA | |  |
| Dmagna_dsx1-a | MatScan | Lim1 | 722 | 728 | 0.87 | + | # GTAATTA | |  |
| Dmagna_dsx1-a | MatScan | Lim3 | 722 | 728 | 0.94 | + | # GTAATTA | |  |
| Dmagna_dsx1-a | MatScan | OdsH | 722 | 728 | 0.95 | + | # GTAATTA | |  |
| Dmagna_dsx1-a | MatScan | OdsH | 722 | 728 | 0.95 | + | # GTAATTA | |  |
| Dmagna_dsx1-a | MatScan | otp | 722 | 728 | 0.92 | + | # GTAATTA | |  |
| Dmagna_dsx1-a | MatScan | pb | 722 | 728 | 0.92 | + | # GTAATTA | |  |
| Dmagna_dsx1-a | MatScan | PHDP | 722 | 728 | 0.93 | + | # GTAATTA | |  |
| Dmagna_dsx1-a | MatScan | Pph13 | 722 | 728 | 0.96 | + | # GTAATTA | |  |
| Dmagna_dsx1-a | MatScan | repo | 722 | 728 | 0.92 | + | # GTAATTA | |  |
| Dmagna_dsx1-a | MatScan | ro | 722 | 728 | 0.94 | + | # GTAATTA | |  |
| Dmagna_dsx1-a | MatScan | Rx | 722 | 728 | 0.92 | + | # GTAATTA | |  |
| Dmagna_dsx1-a | MatScan | slou | 722 | 728 | 0.9 | + | # GTAATTA | |  |
| Dmagna_dsx1-a | MatScan | unc-4 | 722 | 728 | 0.85 | + | # GTAATTA | |  |
| Dmagna_dsx1-a | MatScan | unpg | 722 | 728 | 0.95 | + | # GTAATTA | |  |
| Dmagna_dsx1-a | MatScan | Vsx1 | 722 | 728 | 0.92 | + | # GTAATTA | |  |
| Dmagna_dsx1-a | MatScan | zen2 | 722 | 728 | 0.96 | + | # GTAATTA | |  |
| Dmagna_dsx1-a | MatScan | abd-A | 723 | 729 | 0.88 | - | # ATAATTA | |  |
| Dmagna_dsx1-a | MatScan | al | 723 | 729 | 0.87 | + | # TAATTAT | |  |
| Dmagna_dsx1-a | MatScan | Antp | 723 | 729 | 0.85 | - | # ATAATTA | |  |
| Dmagna_dsx1-a | MatScan | ap | 723 | 729 | 0.93 | - | # ATAATTA | |  |
| Dmagna_dsx1-a | MatScan | Awh | 723 | 729 | 0.92 | - | # ATAATTA | |  |
| Dmagna_dsx1-a | MatScan | bsh | 723 | 729 | 0.88 | - | # ATAATTA | |  |
| Dmagna_dsx1-a | MatScan | btn | 723 | 729 | 0.89 | - | # ATAATTA | |  |
| Dmagna_dsx1-a | MatScan | CG11294 | 723 | 729 | 0.88 | - | # ATAATTA | |  |
| Dmagna_dsx1-a | MatScan | CG13424 | 723 | 729 | 0.88 | - | # ATAATTA | |  |
| Dmagna_dsx1-a | MatScan | CG15696 | 723 | 729 | 0.88 | - | # ATAATTA | |  |
| Dmagna_dsx1-a | MatScan | CG18599 | 723 | 729 | 0.96 | - | # ATAATTA | |  |
| Dmagna_dsx1-a | MatScan | CG32105 | 723 | 729 | 0.91 | - | # ATAATTA | |  |
| Dmagna_dsx1-a | MatScan | CG32532 | 723 | 729 | 0.92 | - | # ATAATTA | |  |
| Dmagna_dsx1-a | MatScan | CG4328 | 723 | 729 | 0.97 | - | # ATAATTA | |  |
| Dmagna_dsx1-a | MatScan | CG7056 | 723 | 730 | 0.87 | - | # TATAATTA | |  |
| Dmagna_dsx1-a | MatScan | CG9876 | 723 | 729 | 0.94 | - | # ATAATTA | |  |
| Dmagna_dsx1-a | MatScan | Dll | 723 | 729 | 0.95 | + | # TAATTAT | |  |
| Dmagna_dsx1-a | MatScan | E5 | 723 | 729 | 0.95 | - | # ATAATTA | |  |
| Dmagna_dsx1-a | MatScan | ems | 723 | 729 | 0.93 | - | # ATAATTA | |  |
| Dmagna_dsx1-a | MatScan | en | 723 | 729 | 0.93 | - | # ATAATTA | |  |
| Dmagna_dsx1-a | MatScan | eve | 723 | 729 | 0.93 | - | # ATAATTA | |  |
| Dmagna_dsx1-a | MatScan | exex | 723 | 729 | 0.93 | - | # ATAATTA | |  |
| Dmagna_dsx1-a | MatScan | exex | 723 | 729 | 0.93 | - | # ATAATTA | |  |
| Dmagna_dsx1-a | MatScan | ftz | 723 | 729 | 0.87 | - | # ATAATTA | |  |
| Dmagna_dsx1-a | MatScan | H2.0 | 723 | 729 | 0.92 | - | # ATAATTA | |  |
| Dmagna_dsx1-a | MatScan | hbn | 723 | 729 | 0.91 | - | # ATAATTA | |  |
| Dmagna_dsx1-a | MatScan | HGTX | 723 | 729 | 0.94 | - | # ATAATTA | |  |
| Dmagna_dsx1-a | MatScan | ind | 723 | 729 | 0.91 | - | # ATAATTA | |  |
| Dmagna_dsx1-a | MatScan | inv | 723 | 730 | 0.93 | - | # TATAATTA | |  |
| Dmagna_dsx1-a | MatScan | lab | 723 | 729 | 0.89 | - | # ATAATTA | |  |
| Dmagna_dsx1-a | MatScan | lbe | 723 | 728 | 0.99 | - | # TAATTA | |  |
| Dmagna_dsx1-a | MatScan | lbe | 723 | 728 | 0.99 | + | # TAATTA | |  |
| Dmagna_dsx1-a | MatScan | lbl | 723 | 728 | 1 | - | # TAATTA | |  |
| Dmagna_dsx1-a | MatScan | lbl | 723 | 728 | 1 | + | # TAATTA | |  |
| Dmagna_dsx1-a | MatScan | Lim1 | 723 | 729 | 0.87 | - | # ATAATTA | |  |
| Dmagna_dsx1-a | MatScan | Lim3 | 723 | 729 | 0.95 | - | # ATAATTA | |  |
| Dmagna_dsx1-a | MatScan | NK7.1 | 723 | 729 | 0.86 | - | # ATAATTA | |  |
| Dmagna_dsx1-a | MatScan | Oct | 723 | 730 | 0.98 | - | # TATAATTA | |  |
| Dmagna_dsx1-a | MatScan | OdsH | 723 | 729 | 0.92 | - | # ATAATTA | |  |
| Dmagna_dsx1-a | MatScan | OdsH | 723 | 729 | 0.92 | - | # ATAATTA | |  |
| Dmagna_dsx1-a | MatScan | otp | 723 | 729 | 0.92 | - | # ATAATTA | |  |
| Dmagna_dsx1-a | MatScan | pb | 723 | 729 | 0.95 | - | # ATAATTA | |  |
| Dmagna_dsx1-a | MatScan | PHDP | 723 | 729 | 0.97 | - | # ATAATTA | |  |
| Dmagna_dsx1-a | MatScan | Pph13 | 723 | 729 | 0.98 | - | # ATAATTA | |  |
| Dmagna_dsx1-a | MatScan | repo | 723 | 729 | 0.9 | - | # ATAATTA | |  |
| Dmagna_dsx1-a | MatScan | ro | 723 | 729 | 0.92 | - | # ATAATTA | |  |
| Dmagna_dsx1-a | MatScan | Rx | 723 | 729 | 0.94 | - | # ATAATTA | |  |
| Dmagna_dsx1-a | MatScan | Scr | 723 | 729 | 0.85 | - | # ATAATTA | |  |
| Dmagna_dsx1-a | MatScan | slou | 723 | 729 | 0.92 | - | # ATAATTA | |  |
| Dmagna_dsx1-a | MatScan | unpg | 723 | 729 | 0.93 | - | # ATAATTA | |  |
| Dmagna_dsx1-a | MatScan | Vsx1 | 723 | 729 | 0.92 | - | # ATAATTA | |  |
| Dmagna_dsx1-a | MatScan | zen | 723 | 729 | 0.87 | - | # ATAATTA | |  |
| Dmagna_dsx1-a | MatScan | zen2 | 723 | 729 | 0.95 | - | # ATAATTA | |  |
| Dmagna_dsx1-a | MatScan | vvl | 727 | 732 | 0.88 | + | # TATACA | |  |
| Dmagna_dsx1-a | MatScan | ara | 728 | 732 | 0.91 | + | # ATACA | |  |
| Dmagna_dsx1-a | MatScan | mirr | 728 | 732 | 0.89 | + | # ATACA | |  |
| Dmagna_dsx1-a | MatScan | z | 732 | 741 | 0.9 | - | # ATGAGTGTGT | |  |
| Dmagna_dsx1-a | MatScan | ara | 743 | 747 | 0.91 | - | # ATACA | |  |
| Dmagna_dsx1-a | MatScan | mirr | 743 | 747 | 0.89 | - | # ATACA | |  |
| Dmagna_dsx1-a | MatScan | Cf2_II | 744 | 753 | 0.85 | - | # ATATACATAC | |  |
| Dmagna_dsx1-a | MatScan | Cf2_II | 744 | 753 | 0.86 | + | # GTATGTATAT | |  |
| Dmagna_dsx1-a | MatScan | CF2-II | 744 | 752 | 0.87 | + | # GTATGTATA | |  |
| Dmagna_dsx1-a | MatScan | CF2-II | 744 | 752 | 0.88 | + | # GTATGTATA | |  |
| Dmagna_dsx1-a | MatScan | Cf2_II | 746 | 755 | 0.86 | - | # ATATATACAT | |  |
| Dmagna_dsx1-a | MatScan | ara | 747 | 751 | 0.91 | - | # ATACA | |  |
| Dmagna_dsx1-a | MatScan | CF2-II | 747 | 755 | 0.87 | - | # ATATATACA | |  |
| Dmagna_dsx1-a | MatScan | mirr | 747 | 751 | 0.89 | - | # ATACA | |  |
| Dmagna_dsx1-a | MatScan | vvl | 747 | 752 | 0.88 | - | # TATACA | |  |
| Dmagna_dsx1-a | MatScan | Cf2_II | 748 | 757 | 0.97 | - | # ATATATATAC | |  |
| Dmagna_dsx1-a | MatScan | Cf2_II | 748 | 757 | 0.99 | + | # GTATATATAT | |  |
| Dmagna_dsx1-a | MatScan | CF2-II | 748 | 756 | 1 | + | # GTATATATA | |  |
| Dmagna_dsx1-a | MatScan | CF2-II | 748 | 756 | 1 | + | # GTATATATA | |  |
| Dmagna_dsx1-a | MatScan | CF2-II | 749 | 757 | 0.95 | - | # ATATATATA | |  |
| Dmagna_dsx1-a | MatScan | CF2-II | 749 | 757 | 0.97 | - | # ATATATATA | |  |
| Dmagna_dsx1-a | MatScan | Cf2_II | 750 | 759 | 0.97 | - | # ATATATATAT | |  |
| Dmagna_dsx1-a | MatScan | Cf2_II | 750 | 759 | 0.97 | + | # ATATATATAT | |  |
| Dmagna_dsx1-a | MatScan | CF2-II | 750 | 758 | 0.95 | + | # ATATATATA | |  |
| Dmagna_dsx1-a | MatScan | CF2-II | 750 | 758 | 0.97 | + | # ATATATATA | |  |
| Dmagna_dsx1-a | MatScan | CF2-II | 751 | 759 | 0.95 | - | # ATATATATA | |  |
| Dmagna_dsx1-a | MatScan | CF2-II | 751 | 759 | 0.97 | - | # ATATATATA | |  |
| Dmagna_dsx1-a | MatScan | Cf2_II | 752 | 761 | 0.97 | - | # ATATATATAT | |  |
| Dmagna_dsx1-a | MatScan | Cf2_II | 752 | 761 | 0.97 | + | # ATATATATAT | |  |
| Dmagna_dsx1-a | MatScan | CF2-II | 752 | 760 | 0.95 | + | # ATATATATA | |  |
| Dmagna_dsx1-a | MatScan | CF2-II | 752 | 760 | 0.97 | + | # ATATATATA | |  |
| Dmagna_dsx1-a | MatScan | CF2-II | 753 | 761 | 0.95 | - | # ATATATATA | |  |
| Dmagna_dsx1-a | MatScan | CF2-II | 753 | 761 | 0.97 | - | # ATATATATA | |  |
| Dmagna_dsx1-a | MatScan | Cf2_II | 754 | 763 | 0.97 | - | # ATATATATAT | |  |
| Dmagna_dsx1-a | MatScan | Cf2_II | 754 | 763 | 0.97 | + | # ATATATATAT | |  |
| Dmagna_dsx1-a | MatScan | CF2-II | 754 | 762 | 0.95 | + | # ATATATATA | |  |
| Dmagna_dsx1-a | MatScan | CF2-II | 754 | 762 | 0.97 | + | # ATATATATA | |  |
| Dmagna_dsx1-a | MatScan | CF2-II | 755 | 763 | 0.95 | - | # ATATATATA | |  |
| Dmagna_dsx1-a | MatScan | CF2-II | 755 | 763 | 0.97 | - | # ATATATATA | |  |
| Dmagna_dsx1-a | MatScan | Cf2_II | 756 | 765 | 0.97 | + | # ATATATATAC | |  |
| Dmagna_dsx1-a | MatScan | Cf2_II | 756 | 765 | 0.99 | - | # GTATATATAT | |  |
| Dmagna_dsx1-a | MatScan | CF2-II | 756 | 764 | 0.95 | + | # ATATATATA | |  |
| Dmagna_dsx1-a | MatScan | CF2-II | 756 | 764 | 0.97 | + | # ATATATATA | |  |
| Dmagna_dsx1-a | MatScan | CF2-II | 757 | 765 | 1 | - | # GTATATATA | |  |
| Dmagna_dsx1-a | MatScan | CF2-II | 757 | 765 | 1 | - | # GTATATATA | |  |
| Dmagna_dsx1-a | MatScan | Cf2_II | 758 | 767 | 0.87 | - | # GTGTATATAT | |  |
| Dmagna_dsx1-a | MatScan | Cf2_II | 758 | 767 | 0.87 | + | # ATATATACAC | |  |
| Dmagna_dsx1-a | MatScan | CF2-II | 758 | 766 | 0.87 | + | # ATATATACA | |  |
| Dmagna_dsx1-a | MatScan | CF2-II | 759 | 767 | 0.87 | - | # GTGTATATA | |  |
| Dmagna_dsx1-a | MatScan | CF2-II | 759 | 767 | 0.89 | - | # GTGTATATA | |  |
| Dmagna_dsx1-a | MatScan | vvl | 761 | 766 | 0.88 | + | # TATACA | |  |
| Dmagna_dsx1-a | MatScan | ara | 762 | 766 | 0.91 | + | # ATACA | |  |
| Dmagna_dsx1-a | MatScan | mirr | 762 | 766 | 0.89 | + | # ATACA | |  |
| Dmagna_dsx1-a | MatScan | Optix | 769 | 773 | 0.87 | - | # CGATA | |  |
| Dmagna_dsx1-a | MatScan | br_Z2 | 785 | 792 | 0.94 | + | # AACTATTT | |  |
| Dmagna_dsx1-a | MatScan | Deaf1 | 792 | 797 | 0.96 | + | # TTCGTT | |  |
| Dmagna_dsx1-a | MatScan | ara | 818 | 822 | 0.91 | - | # ATACA | |  |
| Dmagna_dsx1-a | MatScan | mirr | 818 | 822 | 0.89 | - | # ATACA | |  |
| Dmagna_dsx1-a | MatScan | bap | 833 | 839 | 0.86 | - | # GTAAGTG | |  |
| Dmagna_dsx1-a | MatScan | Eip74EF | 847 | 853 | 1 | - | # CCGGAAG | |  |
| Dmagna_dsx1-a | MatScan | ct | 853 | 858 | 0.92 | - | # CTGAAC | |  |
| Dmagna_dsx1-a | MatScan | ct | 859 | 864 | 0.86 | + | # ATGAAC | |  |
| Dmagna_dsx1-a | MatScan | ara | 861 | 865 | 0.93 | + | # GAACA | |  |
| Dmagna_dsx1-a | MatScan | caup | 861 | 865 | 0.88 | + | # GAACA | |  |
| Dmagna_dsx1-a | MatScan | mirr | 861 | 865 | 0.89 | + | # GAACA | |  |
| Dmagna_dsx1-a | MatScan | btd | 866 | 875 | 0.91 | + | # AGGGGGCGCA | |  |
| Dmagna_dsx1-a | MatScan | AP-1 | 893 | 901 | 0.87 | + | # TTTAGTCAG | |  |
| Dmagna_dsx1-a | MatScan | Abd-B | 907 | 913 | 0.88 | - | # TTTATTT | |  |
| Dmagna_dsx1-a | MatScan | br_Z2 | 907 | 914 | 0.86 | - | # TTTTATTT | |  |
| Dmagna_dsx1-a | MatScan | cad | 907 | 913 | 0.91 | - | # TTTATTT | |  |
| Dmagna_dsx1-a | MatScan | CG42234 | 907 | 913 | 0.85 | - | # TTTATTT | |  |
| Dmagna_dsx1-a | MatScan | CG4328 | 907 | 913 | 0.9 | - | # TTTATTT | |  |
| Dmagna_dsx1-a | MatScan | hb | 920 | 929 | 0.91 | - | # CCAGAAAAAA | |  |
| Dmagna_dsx1-a | MatScan | brk | 926 | 933 | 0.86 | + | # CTGGCTCC | |  |
| Dmagna_dsx1-a | MatScan | Lag1 | 930 | 936 | 0.86 | + | # CTCCCAA | |  |
| Dmagna_dsx1-a | MatScan | Deaf1 | 937 | 942 | 0.87 | - | # TTCGAC | |  |
| Dmagna_dsx1-a | MatScan | Kr | 939 | 949 | 0.89 | + | # CGAAGGGGGTA | |  |
| Dmagna_dsx1-a | MatScan | ara | 967 | 971 | 0.99 | - | # AAACA | |  |
| Dmagna_dsx1-a | MatScan | caup | 967 | 971 | 0.9 | - | # AAACA | |  |
| Dmagna_dsx1-a | MatScan | mirr | 967 | 971 | 1 | - | # AAACA | |  |
| Dmagna_dsx1-a | MatScan | Lag1 | 993 | 999 | 0.99 | + | # CCACCAA | |  |
