# Supplementary material for: Molecular cloning of doublesex genes of four cladocera (water flea) species
Source: BMC Genomics. 2013 Apr 10;14:239. doi: 10.1186/1471-2164-14-239 (PMC3637828; doi:10.1186/1471-2164-14-239)
Supplement: Additional file 17 — DappuDsx1-α TF-map. [file 1471-2164-14-239-S17.doc]

Supplemental Material 17. *DappuDsx1-α* TF-map

| Column Descriptions | | | | Promoter region ID - Species, dsx paralog number, and dsx transcript identifier  Name of program that generated results  Name of transcription factor identified  Start of transcription factor binding site (TFBS)  End of transcription factor binding site (TFBS)  Match score between known TFBS (from TFBS database) and identified Daphnia dsx promoter sequence motif  Strand on which TFBS was identified in sequence  Reading frame for CDS feature types (not used)  Sequence of transcription factor binding motif (from TFBS database) | | | | | | | | | | | | | | | | | | | | | |  |
| --- | --- | --- | --- | --- | --- | --- | --- | --- | --- | --- | --- | --- | --- | --- | --- | --- | --- | --- | --- | --- | --- | --- | --- | --- | --- | --- |
| Sequence ID | | | |  |
| Source | | | |  |
| Type (TF) | | | |  |
| Start | | | |  |
| End | | | |  |
| Score | | | |  |
| Strand | | | |  |
| Phase | | | |  |
| TF Binding Motif | | | |  |
|  |  |  |  | |  |  | |  | |  |  | |  | |  | |  | |  |  |  |  | |  |  | |
| **Sequence ID** | | | | **Source** | | | **Type (TF)** | | **Start** | | | **End** | | **Score** | | **Strand** | | **TF Binding Motif** | | | | |  | | | |
| Dpulex_dsx1-a | | | | MatScan | | | hb | | 7 | | | 16 | | 0.88 | | + | | # TCAGAAAAAA | | | | |  | | | |
| Dpulex_dsx1-a | | | | MatScan | | | hb | | 52 | | | 61 | | 0.86 | | - | | # GGAAAAAAAT | | | | |  | | | |
| Dpulex_dsx1-a | | | | MatScan | | | dl | | 53 | | | 63 | | 0.88 | | - | | # CGGGAAAAAAA | | | | |  | | | |
| Dpulex_dsx1-a | | | | MatScan | | | hb | | 53 | | | 62 | | 0.87 | | - | | # GGGAAAAAAA | | | | |  | | | |
| Dpulex_dsx1-a | | | | MatScan | | | Eip74EF | | 66 | | | 72 | | 0.88 | | + | | # CGGGAAG | | | | |  | | | |
| Dpulex_dsx1-a | | | | MatScan | | | Deaf1 | | 77 | | | 82 | | 1 | | + | | # TTCGTG | | | | |  | | | |
| Dpulex_dsx1-a | | | | MatScan | | | Bcd | | 80 | | | 87 | | 0.86 | | + | | # GTGATTAG | | | | |  | | | |
| Dpulex_dsx1-a | | | | MatScan | | | Dll | | 80 | | | 86 | | 0.87 | | - | | # TAATCAC | | | | |  | | | |
| Dpulex_dsx1-a | | | | MatScan | | | onecut | | 80 | | | 86 | | 0.86 | | + | | # GTGATTA | | | | |  | | | |
| Dpulex_dsx1-a | | | | MatScan | | | ap | | 81 | | | 87 | | 0.89 | | - | | # CTAATCA | | | | |  | | | |
| Dpulex_dsx1-a | | | | MatScan | | | bsh | | 81 | | | 87 | | 0.85 | | - | | # CTAATCA | | | | |  | | | |
| Dpulex_dsx1-a | | | | MatScan | | | CG18599 | | 81 | | | 87 | | 0.9 | | - | | # CTAATCA | | | | |  | | | |
| Dpulex_dsx1-a | | | | MatScan | | | E5 | | 81 | | | 87 | | 0.9 | | - | | # CTAATCA | | | | |  | | | |
| Dpulex_dsx1-a | | | | MatScan | | | ems | | 81 | | | 87 | | 0.9 | | - | | # CTAATCA | | | | |  | | | |
| Dpulex_dsx1-a | | | | MatScan | | | eve | | 81 | | | 87 | | 0.93 | | - | | # CTAATCA | | | | |  | | | |
| Dpulex_dsx1-a | | | | MatScan | | | Gsc | | 81 | | | 86 | | 0.89 | | - | | # TAATCA | | | | |  | | | |
| Dpulex_dsx1-a | | | | MatScan | | | ind | | 81 | | | 87 | | 0.9 | | - | | # CTAATCA | | | | |  | | | |
| Dpulex_dsx1-a | | | | MatScan | | | inv | | 81 | | | 88 | | 0.85 | | - | | # TCTAATCA | | | | |  | | | |
| Dpulex_dsx1-a | | | | MatScan | | | lbe | | 81 | | | 86 | | 0.94 | | - | | # TAATCA | | | | |  | | | |
| Dpulex_dsx1-a | | | | MatScan | | | lbl | | 81 | | | 86 | | 0.92 | | - | | # TAATCA | | | | |  | | | |
| Dpulex_dsx1-a | | | | MatScan | | | Lim3 | | 81 | | | 87 | | 0.88 | | - | | # CTAATCA | | | | |  | | | |
| Dpulex_dsx1-a | | | | MatScan | | | Oct | | 81 | | | 88 | | 0.88 | | - | | # TCTAATCA | | | | |  | | | |
| Dpulex_dsx1-a | | | | MatScan | | | pb | | 81 | | | 87 | | 0.9 | | - | | # CTAATCA | | | | |  | | | |
| Dpulex_dsx1-a | | | | MatScan | | | ro | | 81 | | | 87 | | 0.86 | | - | | # CTAATCA | | | | |  | | | |
| Dpulex_dsx1-a | | | | MatScan | | | vvl | | 81 | | | 86 | | 0.88 | | - | | # TAATCA | | | | |  | | | |
| Dpulex_dsx1-a | | | | MatScan | | | zen | | 81 | | | 87 | | 0.89 | | - | | # CTAATCA | | | | |  | | | |
| Dpulex_dsx1-a | | | | MatScan | | | zen2 | | 81 | | | 87 | | 0.86 | | - | | # CTAATCA | | | | |  | | | |
| Dpulex_dsx1-a | | | | MatScan | | | Six4 | | 89 | | | 94 | | 0.92 | | + | | # TGAAAC | | | | |  | | | |
| Dpulex_dsx1-a | | | | MatScan | | | Dfd | | 90 | | | 105 | | 0.88 | | + | | # GAAACTATTACCGATA | | | | |  | | | |
| Dpulex_dsx1-a | | | | MatScan | | | CG4328 | | 93 | | | 99 | | 0.85 | | + | | # ACTATTA | | | | |  | | | |
| Dpulex_dsx1-a | | | | MatScan | | | Optix | | 101 | | | 105 | | 0.87 | | + | | # CGATA | | | | |  | | | |
| Dpulex_dsx1-a | | | | MatScan | | | ara | | 109 | | | 113 | | 0.89 | | + | | # CAACA | | | | |  | | | |
| Dpulex_dsx1-a | | | | MatScan | | | caup | | 109 | | | 113 | | 0.87 | | + | | # CAACA | | | | |  | | | |
| Dpulex_dsx1-a | | | | MatScan | | | mirr | | 109 | | | 113 | | 0.89 | | + | | # CAACA | | | | |  | | | |
| Dpulex_dsx1-a | | | | MatScan | | | vvl | | 130 | | | 135 | | 0.87 | | - | | # CATGCA | | | | |  | | | |
| Dpulex_dsx1-a | | | | MatScan | | | Ttk | | 131 | | | 138 | | 0.87 | | - | | # GGTCATGC | | | | |  | | | |
| Dpulex_dsx1-a | | | | MatScan | | | CF1 | | 132 | | | 140 | | 0.88 | | - | | # AGGGTCATG | | | | |  | | | |
| Dpulex_dsx1-a | | | | MatScan | | | ara | | 153 | | | 157 | | 1 | | + | | # TAACA | | | | |  | | | |
| Dpulex_dsx1-a | | | | MatScan | | | caup | | 153 | | | 157 | | 1 | | + | | # TAACA | | | | |  | | | |
| Dpulex_dsx1-a | | | | MatScan | | | mirr | | 153 | | | 157 | | 0.99 | | + | | # TAACA | | | | |  | | | |
| Dpulex_dsx1-a | | | | MatScan | | | mtTFA | | 164 | | | 174 | | 0.9 | | - | | # TTCTTATCTAC | | | | |  | | | |
| Dpulex_dsx1-a | | | | MatScan | | | lbe | | 169 | | | 174 | | 0.86 | | + | | # TAAGAA | | | | |  | | | |
| Dpulex_dsx1-a | | | | MatScan | | | hb | | 181 | | | 190 | | 0.92 | | + | | # GAAGAAAAAA | | | | |  | | | |
| Dpulex_dsx1-a | | | | MatScan | | | BR-C | | 184 | | | 198 | | 0.89 | | + | | # GAAAAAACTAAAATT | | | | |  | | | |
| Dpulex_dsx1-a | | | | MatScan | | | br_Z3 | | 187 | | | 197 | | 0.91 | | + | | # AAAACTAAAAT | | | | |  | | | |
| Dpulex_dsx1-a | | | | MatScan | | | C15 | | 197 | | | 203 | | 0.85 | | - | | # TTAAAAA | | | | |  | | | |
| Dpulex_dsx1-a | | | | MatScan | | | lbe | | 197 | | | 202 | | 0.85 | | - | | # TAAAAA | | | | |  | | | |
| Dpulex_dsx1-a | | | | MatScan | | | Deaf1 | | 214 | | | 219 | | 0.96 | | + | | # TTCGGT | | | | |  | | | |
| Dpulex_dsx1-a | | | | MatScan | | | Eip74EF | | 253 | | | 259 | | 1 | | - | | # CCGGAAG | | | | |  | | | |
| Dpulex_dsx1-a | | | | MatScan | | | AP-1 | | 261 | | | 269 | | 0.87 | | + | | # TTTAGTCAG | | | | |  | | | |
| Dpulex_dsx1-a | | | | MatScan | | | hb | | 274 | | | 283 | | 0.89 | | - | | # GAAAAAAAAT | | | | |  | | | |
| Dpulex_dsx1-a | | | | MatScan | | | hb | | 275 | | | 284 | | 0.91 | | - | | # GGAAAAAAAA | | | | |  | | | |
| Dpulex_dsx1-a | | | | MatScan | | | sd | | 288 | | | 299 | | 0.85 | | - | | # GCCATTCAAAGA | | | | |  | | | |
| Dpulex_dsx1-a | | | | MatScan | | | pan | | 289 | | | 296 | | 0.88 | | + | | # CTTTGAAT | | | | |  | | | |
| Dpulex_dsx1-a | | | | MatScan | | | Lag1 | | 323 | | | 329 | | 0.88 | | - | | # CCACCAC | | | | |  | | | |
| Dpulex_dsx1-a | | | | MatScan | | | Lag1 | | 326 | | | 332 | | 0.89 | | - | | # CTACCAC | | | | |  | | | |
| Dpulex_dsx1-a | | | | MatScan | | | Abd-B | | 343 | | | 349 | | 0.92 | | + | | # TTTATGT | | | | |  | | | |
| Dpulex_dsx1-a | | | | MatScan | | | ara | | 349 | | | 353 | | 0.99 | | - | | # AAACA | | | | |  | | | |
| Dpulex_dsx1-a | | | | MatScan | | | caup | | 349 | | | 353 | | 0.9 | | - | | # AAACA | | | | |  | | | |
| Dpulex_dsx1-a | | | | MatScan | | | mirr | | 349 | | | 353 | | 1 | | - | | # AAACA | | | | |  | | | |
| Dpulex_dsx1-a | | | | MatScan | | | bap | | 354 | | | 360 | | 0.86 | | + | | # CTAAGTG | | | | |  | | | |
| Dpulex_dsx1-a | | | | MatScan | | | Trl | | 366 | | | 375 | | 0.85 | | + | | # TTTCTCTCAG | | | | |  | | | |
| Dpulex_dsx1-a | | | | MatScan | | | ovo | | 374 | | | 382 | | 0.93 | | + | | # AGAAACAGT | | | | |  | | | |
| Dpulex_dsx1-a | | | | MatScan | | | prd | | 374 | | | 382 | | 0.93 | | + | | # AGAAACAGT | | | | |  | | | |
| Dpulex_dsx1-a | | | | MatScan | | | ara | | 376 | | | 380 | | 0.99 | | + | | # AAACA | | | | |  | | | |
| Dpulex_dsx1-a | | | | MatScan | | | caup | | 376 | | | 380 | | 0.9 | | + | | # AAACA | | | | |  | | | |
| Dpulex_dsx1-a | | | | MatScan | | | mirr | | 376 | | | 380 | | 1 | | + | | # AAACA | | | | |  | | | |
| Dpulex_dsx1-a | | | | MatScan | | | ct | | 403 | | | 408 | | 0.9 | | + | | # CTAAAC | | | | |  | | | |
| Dpulex_dsx1-a | | | | MatScan | | | lbe | | 419 | | | 424 | | 0.95 | | - | | # TAACCA | | | | |  | | | |
| Dpulex_dsx1-a | | | | MatScan | | | H2.0 | | 421 | | | 427 | | 0.85 | | + | | # GTTATAA | | | | |  | | | |
| Dpulex_dsx1-a | | | | MatScan | | | ems | | 432 | | | 438 | | 0.85 | | + | | # CTAATGT | | | | |  | | | |
| Dpulex_dsx1-a | | | | MatScan | | | eve | | 432 | | | 438 | | 0.87 | | + | | # CTAATGT | | | | |  | | | |
| Dpulex_dsx1-a | | | | MatScan | | | ara | | 436 | | | 440 | | 0.89 | | - | | # CAACA | | | | |  | | | |
| Dpulex_dsx1-a | | | | MatScan | | | caup | | 436 | | | 440 | | 0.87 | | - | | # CAACA | | | | |  | | | |
| Dpulex_dsx1-a | | | | MatScan | | | mirr | | 436 | | | 440 | | 0.89 | | - | | # CAACA | | | | |  | | | |
| Dpulex_dsx1-a | | | | MatScan | | | run::Bgb | | 438 | | | 446 | | 0.86 | | - | | # AAACAGCAA | | | | |  | | | |
| Dpulex_dsx1-a | | | | MatScan | | | ara | | 442 | | | 446 | | 0.99 | | - | | # AAACA | | | | |  | | | |
| Dpulex_dsx1-a | | | | MatScan | | | caup | | 442 | | | 446 | | 0.9 | | - | | # AAACA | | | | |  | | | |
| Dpulex_dsx1-a | | | | MatScan | | | mirr | | 442 | | | 446 | | 1 | | - | | # AAACA | | | | |  | | | |
| Dpulex_dsx1-a | | | | MatScan | | | exd | | 443 | | | 450 | | 0.88 | | + | | # GTTTGAAA | | | | |  | | | |
| Dpulex_dsx1-a | | | | MatScan | | | pan | | 443 | | | 450 | | 0.85 | | + | | # GTTTGAAA | | | | |  | | | |
| Dpulex_dsx1-a | | | | MatScan | | | Six4 | | 446 | | | 451 | | 0.92 | | + | | # TGAAAC | | | | |  | | | |
| Dpulex_dsx1-a | | | | MatScan | | | ara | | 448 | | | 452 | | 0.99 | | + | | # AAACA | | | | |  | | | |
| Dpulex_dsx1-a | | | | MatScan | | | caup | | 448 | | | 452 | | 0.9 | | + | | # AAACA | | | | |  | | | |
| Dpulex_dsx1-a | | | | MatScan | | | mirr | | 448 | | | 452 | | 1 | | + | | # AAACA | | | | |  | | | |
| Dpulex_dsx1-a | | | | MatScan | | | Bcd | | 455 | | | 462 | | 0.87 | | - | | # GAGATTAG | | | | |  | | | |
| Dpulex_dsx1-a | | | | MatScan | | | Gsc | | 456 | | | 461 | | 0.94 | | + | | # TAATCT | | | | |  | | | |
| Dpulex_dsx1-a | | | | MatScan | | | oc | | 456 | | | 461 | | 0.85 | | + | | # TAATCT | | | | |  | | | |
| Dpulex_dsx1-a | | | | MatScan | | | Deaf1 | | 460 | | | 465 | | 0.89 | | + | | # CTCGGC | | | | |  | | | |
| Dpulex_dsx1-a | | | | MatScan | | | oc | | 463 | | | 468 | | 0.86 | | - | | # TAAGCC | | | | |  | | | |
| Dpulex_dsx1-a | | | | MatScan | | | gt | | 465 | | | 474 | | 0.85 | | - | | # GTTACATAAG | | | | |  | | | |
| Dpulex_dsx1-a | | | | MatScan | | | gt | | 465 | | | 474 | | 0.85 | | + | | # CTTATGTAAC | | | | |  | | | |
| Dpulex_dsx1-a | | | | MatScan | | | ara | | 469 | | | 473 | | 0.91 | | - | | # TTACA | | | | |  | | | |
| Dpulex_dsx1-a | | | | MatScan | | | caup | | 469 | | | 473 | | 0.87 | | - | | # TTACA | | | | |  | | | |
| Dpulex_dsx1-a | | | | MatScan | | | mirr | | 469 | | | 473 | | 0.88 | | - | | # TTACA | | | | |  | | | |
| Dpulex_dsx1-a | | | | MatScan | | | CG42234 | | 475 | | | 481 | | 0.87 | | + | | # TTGATCA | | | | |  | | | |
| Dpulex_dsx1-a | | | | MatScan | | | CG42234 | | 476 | | | 482 | | 0.87 | | - | | # TTGATCA | | | | |  | | | |
| Dpulex_dsx1-a | | | | MatScan | | | pan | | 477 | | | 484 | | 0.96 | | - | | # ATTTGATC | | | | |  | | | |
| Dpulex_dsx1-a | | | | MatScan | | | Dfd | | 480 | | | 495 | | 0.89 | | + | | # CAAATGATTAACTCCC | | | | |  | | | |
| Dpulex_dsx1-a | | | | MatScan | | | ems | | 480 | | | 486 | | 0.85 | | + | | # CAAATGA | | | | |  | | | |
| Dpulex_dsx1-a | | | | MatScan | | | CG4328 | | 483 | | | 489 | | 0.88 | | + | | # ATGATTA | | | | |  | | | |
| Dpulex_dsx1-a | | | | MatScan | | | dri | | 483 | | | 492 | | 0.86 | | + | | # ATGATTAACT | | | | |  | | | |
| Dpulex_dsx1-a | | | | MatScan | | | onecut | | 483 | | | 489 | | 0.87 | | + | | # ATGATTA | | | | |  | | | |
| Dpulex_dsx1-a | | | | MatScan | | | abd-A | | 484 | | | 490 | | 0.89 | | - | | # TTAATCA | | | | |  | | | |
| Dpulex_dsx1-a | | | | MatScan | | | al | | 484 | | | 490 | | 0.85 | | + | | # TGATTAA | | | | |  | | | |
| Dpulex_dsx1-a | | | | MatScan | | | Antp | | 484 | | | 490 | | 0.91 | | - | | # TTAATCA | | | | |  | | | |
| Dpulex_dsx1-a | | | | MatScan | | | ap | | 484 | | | 490 | | 0.86 | | - | | # TTAATCA | | | | |  | | | |
| Dpulex_dsx1-a | | | | MatScan | | | Awh | | 484 | | | 490 | | 0.87 | | - | | # TTAATCA | | | | |  | | | |
| Dpulex_dsx1-a | | | | MatScan | | | bsh | | 484 | | | 490 | | 0.93 | | - | | # TTAATCA | | | | |  | | | |
| Dpulex_dsx1-a | | | | MatScan | | | btn | | 484 | | | 490 | | 0.89 | | - | | # TTAATCA | | | | |  | | | |
| Dpulex_dsx1-a | | | | MatScan | | | C15 | | 484 | | | 490 | | 0.93 | | - | | # TTAATCA | | | | |  | | | |
| Dpulex_dsx1-a | | | | MatScan | | | CG18599 | | 484 | | | 490 | | 0.9 | | - | | # TTAATCA | | | | |  | | | |
| Dpulex_dsx1-a | | | | MatScan | | | CG42234 | | 484 | | | 490 | | 0.91 | | - | | # TTAATCA | | | | |  | | | |
| Dpulex_dsx1-a | | | | MatScan | | | Dfd | | 484 | | | 490 | | 0.87 | | - | | # TTAATCA | | | | |  | | | |
| Dpulex_dsx1-a | | | | MatScan | | | E5 | | 484 | | | 490 | | 0.91 | | - | | # TTAATCA | | | | |  | | | |
| Dpulex_dsx1-a | | | | MatScan | | | ems | | 484 | | | 490 | | 0.92 | | - | | # TTAATCA | | | | |  | | | |
| Dpulex_dsx1-a | | | | MatScan | | | eve | | 484 | | | 490 | | 0.92 | | - | | # TTAATCA | | | | |  | | | |
| Dpulex_dsx1-a | | | | MatScan | | | ftz | | 484 | | | 490 | | 0.92 | | - | | # TTAATCA | | | | |  | | | |
| Dpulex_dsx1-a | | | | MatScan | | | Gsc | | 484 | | | 489 | | 0.89 | | - | | # TAATCA | | | | |  | | | |
| Dpulex_dsx1-a | | | | MatScan | | | H2.0 | | 484 | | | 490 | | 0.91 | | - | | # TTAATCA | | | | |  | | | |
| Dpulex_dsx1-a | | | | MatScan | | | HGTX | | 484 | | | 490 | | 0.89 | | - | | # TTAATCA | | | | |  | | | |
| Dpulex_dsx1-a | | | | MatScan | | | ind | | 484 | | | 490 | | 0.87 | | - | | # TTAATCA | | | | |  | | | |
| Dpulex_dsx1-a | | | | MatScan | | | lab | | 484 | | | 490 | | 0.9 | | - | | # TTAATCA | | | | |  | | | |
| Dpulex_dsx1-a | | | | MatScan | | | lbe | | 484 | | | 489 | | 0.94 | | - | | # TAATCA | | | | |  | | | |
| Dpulex_dsx1-a | | | | MatScan | | | lbl | | 484 | | | 489 | | 0.92 | | - | | # TAATCA | | | | |  | | | |
| Dpulex_dsx1-a | | | | MatScan | | | Lim1 | | 484 | | | 490 | | 0.85 | | - | | # TTAATCA | | | | |  | | | |
| Dpulex_dsx1-a | | | | MatScan | | | Lim3 | | 484 | | | 490 | | 0.89 | | - | | # TTAATCA | | | | |  | | | |
| Dpulex_dsx1-a | | | | MatScan | | | Oct | | 484 | | | 491 | | 0.92 | | - | | # GTTAATCA | | | | |  | | | |
| Dpulex_dsx1-a | | | | MatScan | | | otp | | 484 | | | 490 | | 0.88 | | - | | # TTAATCA | | | | |  | | | |
| Dpulex_dsx1-a | | | | MatScan | | | pb | | 484 | | | 490 | | 0.91 | | - | | # TTAATCA | | | | |  | | | |
| Dpulex_dsx1-a | | | | MatScan | | | Ptx1 | | 484 | | | 490 | | 0.86 | | - | | # TTAATCA | | | | |  | | | |
| Dpulex_dsx1-a | | | | MatScan | | | Scr | | 484 | | | 490 | | 0.88 | | - | | # TTAATCA | | | | |  | | | |
| Dpulex_dsx1-a | | | | MatScan | | | slou | | 484 | | | 490 | | 0.89 | | - | | # TTAATCA | | | | |  | | | |
| Dpulex_dsx1-a | | | | MatScan | | | Vsx1 | | 484 | | | 490 | | 0.87 | | - | | # TTAATCA | | | | |  | | | |
| Dpulex_dsx1-a | | | | MatScan | | | vvl | | 484 | | | 489 | | 0.88 | | - | | # TAATCA | | | | |  | | | |
| Dpulex_dsx1-a | | | | MatScan | | | zen2 | | 484 | | | 490 | | 0.89 | | - | | # TTAATCA | | | | |  | | | |
| Dpulex_dsx1-a | | | | MatScan | | | vvl | | 510 | | | 515 | | 0.87 | | - | | # CATGCA | | | | |  | | | |
| Dpulex_dsx1-a | | | | MatScan | | | vvl | | 525 | | | 530 | | 0.98 | | - | | # TATTCA | | | | |  | | | |
| Dpulex_dsx1-a | | | | MatScan | | | Deaf1 | | 535 | | | 540 | | 0.96 | | + | | # TTCGTT | | | | |  | | | |
| Dpulex_dsx1-a | | | | MatScan | | | Deaf1 | | 547 | | | 552 | | 0.87 | | + | | # TTCGCC | | | | |  | | | |
| Dpulex_dsx1-a | | | | MatScan | | | Dfd | | 564 | | | 579 | | 0.86 | | - | | # GGCAAAATTACATTTT | | | | |  | | | |
| Dpulex_dsx1-a | | | | MatScan | | | ara | | 568 | | | 572 | | 0.91 | | - | | # TTACA | | | | |  | | | |
| Dpulex_dsx1-a | | | | MatScan | | | caup | | 568 | | | 572 | | 0.87 | | - | | # TTACA | | | | |  | | | |
| Dpulex_dsx1-a | | | | MatScan | | | mirr | | 568 | | | 572 | | 0.88 | | - | | # TTACA | | | | |  | | | |
| Dpulex_dsx1-a | | | | MatScan | | | exex | | 569 | | | 575 | | 0.86 | | + | | # GTAATTT | | | | |  | | | |
| Dpulex_dsx1-a | | | | MatScan | | | exex | | 569 | | | 575 | | 0.86 | | + | | # GTAATTT | | | | |  | | | |
| Dpulex_dsx1-a | | | | MatScan | | | OdsH | | 569 | | | 575 | | 0.86 | | + | | # GTAATTT | | | | |  | | | |
| Dpulex_dsx1-a | | | | MatScan | | | OdsH | | 569 | | | 575 | | 0.86 | | + | | # GTAATTT | | | | |  | | | |
| Dpulex_dsx1-a | | | | MatScan | | | PHDP | | 569 | | | 575 | | 0.89 | | + | | # GTAATTT | | | | |  | | | |
| Dpulex_dsx1-a | | | | MatScan | | | Pph13 | | 569 | | | 575 | | 0.86 | | + | | # GTAATTT | | | | |  | | | |
| Dpulex_dsx1-a | | | | MatScan | | | CG4328 | | 570 | | | 576 | | 0.87 | | - | | # AAAATTA | | | | |  | | | |
| Dpulex_dsx1-a | | | | MatScan | | | Dll | | 570 | | | 576 | | 0.91 | | + | | # TAATTTT | | | | |  | | | |
| Dpulex_dsx1-a | | | | MatScan | | | lbl | | 570 | | | 575 | | 0.85 | | + | | # TAATTT | | | | |  | | | |
| Dpulex_dsx1-a | | | | MatScan | | | ara | | 587 | | | 591 | | 0.89 | | - | | # CAACA | | | | |  | | | |
| Dpulex_dsx1-a | | | | MatScan | | | caup | | 587 | | | 591 | | 0.87 | | - | | # CAACA | | | | |  | | | |
| Dpulex_dsx1-a | | | | MatScan | | | mirr | | 587 | | | 591 | | 0.89 | | - | | # CAACA | | | | |  | | | |
| Dpulex_dsx1-a | | | | MatScan | | | ara | | 593 | | | 597 | | 0.99 | | - | | # AAACA | | | | |  | | | |
| Dpulex_dsx1-a | | | | MatScan | | | caup | | 593 | | | 597 | | 0.9 | | - | | # AAACA | | | | |  | | | |
| Dpulex_dsx1-a | | | | MatScan | | | mirr | | 593 | | | 597 | | 1 | | - | | # AAACA | | | | |  | | | |
| Dpulex_dsx1-a | | | | MatScan | | | slbo | | 595 | | | 602 | | 0.94 | | - | | # ATTACAAA | | | | |  | | | |
| Dpulex_dsx1-a | | | | MatScan | | | ara | | 597 | | | 601 | | 0.91 | | - | | # TTACA | | | | |  | | | |
| Dpulex_dsx1-a | | | | MatScan | | | caup | | 597 | | | 601 | | 0.87 | | - | | # TTACA | | | | |  | | | |
| Dpulex_dsx1-a | | | | MatScan | | | mirr | | 597 | | | 601 | | 0.88 | | - | | # TTACA | | | | |  | | | |
| Dpulex_dsx1-a | | | | MatScan | | | E5 | | 598 | | | 604 | | 0.88 | | + | | # GTAATAA | | | | |  | | | |
| Dpulex_dsx1-a | | | | MatScan | | | exex | | 598 | | | 604 | | 0.86 | | + | | # GTAATAA | | | | |  | | | |
| Dpulex_dsx1-a | | | | MatScan | | | exex | | 598 | | | 604 | | 0.86 | | + | | # GTAATAA | | | | |  | | | |
| Dpulex_dsx1-a | | | | MatScan | | | H2.0 | | 598 | | | 604 | | 0.86 | | + | | # GTAATAA | | | | |  | | | |
| Dpulex_dsx1-a | | | | MatScan | | | zen2 | | 598 | | | 604 | | 0.85 | | + | | # GTAATAA | | | | |  | | | |
| Dpulex_dsx1-a | | | | MatScan | | | abd-A | | 599 | | | 605 | | 0.87 | | - | | # TTTATTA | | | | |  | | | |
| Dpulex_dsx1-a | | | | MatScan | | | Abd-B | | 599 | | | 605 | | 0.97 | | - | | # TTTATTA | | | | |  | | | |
| Dpulex_dsx1-a | | | | MatScan | | | al | | 599 | | | 605 | | 0.85 | | + | | # TAATAAA | | | | |  | | | |
| Dpulex_dsx1-a | | | | MatScan | | | Awh | | 599 | | | 605 | | 0.86 | | - | | # TTTATTA | | | | |  | | | |
| Dpulex_dsx1-a | | | | MatScan | | | C15 | | 599 | | | 605 | | 0.85 | | - | | # TTTATTA | | | | |  | | | |
| Dpulex_dsx1-a | | | | MatScan | | | cad | | 599 | | | 605 | | 1 | | - | | # TTTATTA | | | | |  | | | |
| Dpulex_dsx1-a | | | | MatScan | | | CG15696 | | 599 | | | 605 | | 0.91 | | - | | # TTTATTA | | | | |  | | | |
| Dpulex_dsx1-a | | | | MatScan | | | CG32105 | | 599 | | | 605 | | 0.87 | | - | | # TTTATTA | | | | |  | | | |
| Dpulex_dsx1-a | | | | MatScan | | | CG42234 | | 599 | | | 605 | | 1 | | - | | # TTTATTA | | | | |  | | | |
| Dpulex_dsx1-a | | | | MatScan | | | CG4328 | | 599 | | | 605 | | 1 | | - | | # TTTATTA | | | | |  | | | |
| Dpulex_dsx1-a | | | | MatScan | | | CG7056 | | 599 | | | 606 | | 0.88 | | - | | # TTTTATTA | | | | |  | | | |
| Dpulex_dsx1-a | | | | MatScan | | | H2.0 | | 599 | | | 605 | | 0.99 | | - | | # TTTATTA | | | | |  | | | |
| Dpulex_dsx1-a | | | | MatScan | | | hb | | 599 | | | 608 | | 0.87 | | + | | # TAATAAAAAC | | | | |  | | | |
| Dpulex_dsx1-a | | | | MatScan | | | lbe | | 599 | | | 604 | | 0.93 | | + | | # TAATAA | | | | |  | | | |
| Dpulex_dsx1-a | | | | MatScan | | | lbl | | 599 | | | 604 | | 0.9 | | + | | # TAATAA | | | | |  | | | |
| Dpulex_dsx1-a | | | | MatScan | | | Lim1 | | 599 | | | 605 | | 0.85 | | - | | # TTTATTA | | | | |  | | | |
| Dpulex_dsx1-a | | | | MatScan | | | Lim3 | | 599 | | | 605 | | 0.86 | | - | | # TTTATTA | | | | |  | | | |
| Dpulex_dsx1-a | | | | MatScan | | | repo | | 599 | | | 605 | | 0.85 | | - | | # TTTATTA | | | | |  | | | |
| Dpulex_dsx1-a | | | | MatScan | | | Ubx | | 599 | | | 606 | | 0.89 | | - | | # TTTTATTA | | | | |  | | | |
| Dpulex_dsx1-a | | | | MatScan | | | lbe | | 602 | | | 607 | | 0.85 | | + | | # TAAAAA | | | | |  | | | |
| Dpulex_dsx1-a | | | | MatScan | | | ara | | 609 | | | 613 | | 0.91 | | - | | # ATACA | | | | |  | | | |
| Dpulex_dsx1-a | | | | MatScan | | | mirr | | 609 | | | 613 | | 0.89 | | - | | # ATACA | | | | |  | | | |
| Dpulex_dsx1-a | | | | MatScan | | | Optix | | 611 | | | 615 | | 0.87 | | - | | # CGATA | | | | |  | | | |
| Dpulex_dsx1-a | | | | MatScan | | | nub | | 640 | | | 651 | | 0.89 | | - | | # TATGCAAAGCAG | | | | |  | | | |
| Dpulex_dsx1-a | | | | MatScan | | | vvl | | 646 | | | 651 | | 1 | | - | | # TATGCA | | | | |  | | | |
| Dpulex_dsx1-a | | | | MatScan | | | Dfd | | 653 | | | 668 | | 0.92 | | + | | # TTTGTGATTAATATTT | | | | |  | | | |
| Dpulex_dsx1-a | | | | MatScan | | | Dfd | | 655 | | | 670 | | 0.87 | | - | | # AGAAATATTAATCACA | | | | |  | | | |
| Dpulex_dsx1-a | | | | MatScan | | | Bcd | | 656 | | | 663 | | 0.91 | | + | | # GTGATTAA | | | | |  | | | |
| Dpulex_dsx1-a | | | | MatScan | | | Dll | | 656 | | | 662 | | 0.87 | | - | | # TAATCAC | | | | |  | | | |
| Dpulex_dsx1-a | | | | MatScan | | | dri | | 656 | | | 665 | | 0.94 | | + | | # GTGATTAATA | | | | |  | | | |
| Dpulex_dsx1-a | | | | MatScan | | | onecut | | 656 | | | 662 | | 0.86 | | + | | # GTGATTA | | | | |  | | | |
| Dpulex_dsx1-a | | | | MatScan | | | abd-A | | 657 | | | 663 | | 0.89 | | - | | # TTAATCA | | | | |  | | | |
| Dpulex_dsx1-a | | | | MatScan | | | al | | 657 | | | 663 | | 0.85 | | + | | # TGATTAA | | | | |  | | | |
| Dpulex_dsx1-a | | | | MatScan | | | Antp | | 657 | | | 663 | | 0.91 | | - | | # TTAATCA | | | | |  | | | |
| Dpulex_dsx1-a | | | | MatScan | | | ap | | 657 | | | 663 | | 0.86 | | - | | # TTAATCA | | | | |  | | | |
| Dpulex_dsx1-a | | | | MatScan | | | Awh | | 657 | | | 663 | | 0.87 | | - | | # TTAATCA | | | | |  | | | |
| Dpulex_dsx1-a | | | | MatScan | | | bsh | | 657 | | | 663 | | 0.93 | | - | | # TTAATCA | | | | |  | | | |
| Dpulex_dsx1-a | | | | MatScan | | | btn | | 657 | | | 663 | | 0.89 | | - | | # TTAATCA | | | | |  | | | |
| Dpulex_dsx1-a | | | | MatScan | | | C15 | | 657 | | | 663 | | 0.93 | | - | | # TTAATCA | | | | |  | | | |
| Dpulex_dsx1-a | | | | MatScan | | | CG18599 | | 657 | | | 663 | | 0.9 | | - | | # TTAATCA | | | | |  | | | |
| Dpulex_dsx1-a | | | | MatScan | | | CG42234 | | 657 | | | 663 | | 0.91 | | - | | # TTAATCA | | | | |  | | | |
| Dpulex_dsx1-a | | | | MatScan | | | Dfd | | 657 | | | 663 | | 0.87 | | - | | # TTAATCA | | | | |  | | | |
| Dpulex_dsx1-a | | | | MatScan | | | E5 | | 657 | | | 663 | | 0.91 | | - | | # TTAATCA | | | | |  | | | |
| Dpulex_dsx1-a | | | | MatScan | | | ems | | 657 | | | 663 | | 0.92 | | - | | # TTAATCA | | | | |  | | | |
| Dpulex_dsx1-a | | | | MatScan | | | eve | | 657 | | | 663 | | 0.92 | | - | | # TTAATCA | | | | |  | | | |
| Dpulex_dsx1-a | | | | MatScan | | | ftz | | 657 | | | 663 | | 0.92 | | - | | # TTAATCA | | | | |  | | | |
| Dpulex_dsx1-a | | | | MatScan | | | Gsc | | 657 | | | 662 | | 0.89 | | - | | # TAATCA | | | | |  | | | |
| Dpulex_dsx1-a | | | | MatScan | | | H2.0 | | 657 | | | 663 | | 0.91 | | - | | # TTAATCA | | | | |  | | | |
| Dpulex_dsx1-a | | | | MatScan | | | HGTX | | 657 | | | 663 | | 0.89 | | - | | # TTAATCA | | | | |  | | | |
| Dpulex_dsx1-a | | | | MatScan | | | ind | | 657 | | | 663 | | 0.87 | | - | | # TTAATCA | | | | |  | | | |
| Dpulex_dsx1-a | | | | MatScan | | | lab | | 657 | | | 663 | | 0.9 | | - | | # TTAATCA | | | | |  | | | |
| Dpulex_dsx1-a | | | | MatScan | | | lbe | | 657 | | | 662 | | 0.94 | | - | | # TAATCA | | | | |  | | | |
| Dpulex_dsx1-a | | | | MatScan | | | lbl | | 657 | | | 662 | | 0.92 | | - | | # TAATCA | | | | |  | | | |
| Dpulex_dsx1-a | | | | MatScan | | | Lim1 | | 657 | | | 663 | | 0.85 | | - | | # TTAATCA | | | | |  | | | |
| Dpulex_dsx1-a | | | | MatScan | | | Lim3 | | 657 | | | 663 | | 0.89 | | - | | # TTAATCA | | | | |  | | | |
| Dpulex_dsx1-a | | | | MatScan | | | Oct | | 657 | | | 664 | | 0.88 | | - | | # ATTAATCA | | | | |  | | | |
| Dpulex_dsx1-a | | | | MatScan | | | otp | | 657 | | | 663 | | 0.88 | | - | | # TTAATCA | | | | |  | | | |
| Dpulex_dsx1-a | | | | MatScan | | | pb | | 657 | | | 663 | | 0.91 | | - | | # TTAATCA | | | | |  | | | |
| Dpulex_dsx1-a | | | | MatScan | | | Ptx1 | | 657 | | | 663 | | 0.86 | | - | | # TTAATCA | | | | |  | | | |
| Dpulex_dsx1-a | | | | MatScan | | | Scr | | 657 | | | 663 | | 0.88 | | - | | # TTAATCA | | | | |  | | | |
| Dpulex_dsx1-a | | | | MatScan | | | slou | | 657 | | | 663 | | 0.89 | | - | | # TTAATCA | | | | |  | | | |
| Dpulex_dsx1-a | | | | MatScan | | | Vsx1 | | 657 | | | 663 | | 0.87 | | - | | # TTAATCA | | | | |  | | | |
| Dpulex_dsx1-a | | | | MatScan | | | vvl | | 657 | | | 662 | | 0.88 | | - | | # TAATCA | | | | |  | | | |
| Dpulex_dsx1-a | | | | MatScan | | | zen2 | | 657 | | | 663 | | 0.89 | | - | | # TTAATCA | | | | |  | | | |
| Dpulex_dsx1-a | | | | MatScan | | | vvl | | 660 | | | 665 | | 0.88 | | - | | # TATTAA | | | | |  | | | |
| Dpulex_dsx1-a | | | | MatScan | | | CG4328 | | 661 | | | 667 | | 0.89 | | - | | # AATATTA | | | | |  | | | |
| Dpulex_dsx1-a | | | | MatScan | | | Abd-B | | 702 | | | 708 | | 1 | | + | | # TTTATGA | | | | |  | | | |
| Dpulex_dsx1-a | | | | MatScan | | | cad | | 702 | | | 708 | | 0.91 | | + | | # TTTATGA | | | | |  | | | |
| Dpulex_dsx1-a | | | | MatScan | | | CG42234 | | 702 | | | 708 | | 0.98 | | + | | # TTTATGA | | | | |  | | | |
| Dpulex_dsx1-a | | | | MatScan | | | CG4328 | | 702 | | | 708 | | 0.88 | | + | | # TTTATGA | | | | |  | | | |
| Dpulex_dsx1-a | | | | MatScan | | | H2.0 | | 702 | | | 708 | | 0.95 | | + | | # TTTATGA | | | | |  | | | |
| Dpulex_dsx1-a | | | | MatScan | | | H2.0 | | 703 | | | 709 | | 0.86 | | - | | # TTCATAA | | | | |  | | | |
| Dpulex_dsx1-a | | | | MatScan | | | vvl | | 704 | | | 709 | | 0.9 | | + | | # TATGAA | | | | |  | | | |
| Dpulex_dsx1-a | | | | MatScan | | | ara | | 712 | | | 716 | | 0.89 | | - | | # CAACA | | | | |  | | | |
| Dpulex_dsx1-a | | | | MatScan | | | caup | | 712 | | | 716 | | 0.87 | | - | | # CAACA | | | | |  | | | |
| Dpulex_dsx1-a | | | | MatScan | | | mirr | | 712 | | | 716 | | 0.89 | | - | | # CAACA | | | | |  | | | |
| Dpulex_dsx1-a | | | | MatScan | | | mtTFA | | 714 | | | 724 | | 0.88 | | - | | # TAATTATCCAA | | | | |  | | | |
| Dpulex_dsx1-a | | | | MatScan | | | vvl | | 715 | | | 720 | | 0.88 | | - | | # TATCCA | | | | |  | | | |
| Dpulex_dsx1-a | | | | MatScan | | | bcd | | 716 | | | 721 | | 0.86 | | - | | # TTATCC | | | | |  | | | |
| Dpulex_dsx1-a | | | | MatScan | | | inv | | 717 | | | 724 | | 0.87 | | + | | # GATAATTA | | | | |  | | | |
| Dpulex_dsx1-a | | | | MatScan | | | Oct | | 717 | | | 724 | | 0.96 | | + | | # GATAATTA | | | | |  | | | |
| Dpulex_dsx1-a | | | | MatScan | | | abd-A | | 718 | | | 724 | | 0.88 | | + | | # ATAATTA | | | | |  | | | |
| Dpulex_dsx1-a | | | | MatScan | | | al | | 718 | | | 724 | | 0.87 | | - | | # TAATTAT | | | | |  | | | |
| Dpulex_dsx1-a | | | | MatScan | | | Antp | | 718 | | | 724 | | 0.85 | | + | | # ATAATTA | | | | |  | | | |
| Dpulex_dsx1-a | | | | MatScan | | | ap | | 718 | | | 724 | | 0.93 | | + | | # ATAATTA | | | | |  | | | |
| Dpulex_dsx1-a | | | | MatScan | | | Awh | | 718 | | | 724 | | 0.92 | | + | | # ATAATTA | | | | |  | | | |
| Dpulex_dsx1-a | | | | MatScan | | | bsh | | 718 | | | 724 | | 0.88 | | + | | # ATAATTA | | | | |  | | | |
| Dpulex_dsx1-a | | | | MatScan | | | btn | | 718 | | | 724 | | 0.89 | | + | | # ATAATTA | | | | |  | | | |
| Dpulex_dsx1-a | | | | MatScan | | | CG11294 | | 718 | | | 724 | | 0.88 | | + | | # ATAATTA | | | | |  | | | |
| Dpulex_dsx1-a | | | | MatScan | | | CG13424 | | 718 | | | 724 | | 0.88 | | + | | # ATAATTA | | | | |  | | | |
| Dpulex_dsx1-a | | | | MatScan | | | CG15696 | | 718 | | | 724 | | 0.88 | | + | | # ATAATTA | | | | |  | | | |
| Dpulex_dsx1-a | | | | MatScan | | | CG18599 | | 718 | | | 724 | | 0.96 | | + | | # ATAATTA | | | | |  | | | |
| Dpulex_dsx1-a | | | | MatScan | | | CG32105 | | 718 | | | 724 | | 0.91 | | + | | # ATAATTA | | | | |  | | | |
| Dpulex_dsx1-a | | | | MatScan | | | CG32532 | | 718 | | | 724 | | 0.92 | | + | | # ATAATTA | | | | |  | | | |
| Dpulex_dsx1-a | | | | MatScan | | | CG4328 | | 718 | | | 724 | | 0.97 | | + | | # ATAATTA | | | | |  | | | |
| Dpulex_dsx1-a | | | | MatScan | | | CG9876 | | 718 | | | 724 | | 0.94 | | + | | # ATAATTA | | | | |  | | | |
| Dpulex_dsx1-a | | | | MatScan | | | Dll | | 718 | | | 724 | | 0.95 | | - | | # TAATTAT | | | | |  | | | |
| Dpulex_dsx1-a | | | | MatScan | | | E5 | | 718 | | | 724 | | 0.95 | | + | | # ATAATTA | | | | |  | | | |
| Dpulex_dsx1-a | | | | MatScan | | | ems | | 718 | | | 724 | | 0.93 | | + | | # ATAATTA | | | | |  | | | |
| Dpulex_dsx1-a | | | | MatScan | | | en | | 718 | | | 724 | | 0.93 | | + | | # ATAATTA | | | | |  | | | |
| Dpulex_dsx1-a | | | | MatScan | | | eve | | 718 | | | 724 | | 0.93 | | + | | # ATAATTA | | | | |  | | | |
| Dpulex_dsx1-a | | | | MatScan | | | exex | | 718 | | | 724 | | 0.93 | | + | | # ATAATTA | | | | |  | | | |
| Dpulex_dsx1-a | | | | MatScan | | | exex | | 718 | | | 724 | | 0.93 | | + | | # ATAATTA | | | | |  | | | |
| Dpulex_dsx1-a | | | | MatScan | | | ftz | | 718 | | | 724 | | 0.87 | | + | | # ATAATTA | | | | |  | | | |
| Dpulex_dsx1-a | | | | MatScan | | | H2.0 | | 718 | | | 724 | | 0.92 | | + | | # ATAATTA | | | | |  | | | |
| Dpulex_dsx1-a | | | | MatScan | | | hbn | | 718 | | | 724 | | 0.91 | | + | | # ATAATTA | | | | |  | | | |
| Dpulex_dsx1-a | | | | MatScan | | | HGTX | | 718 | | | 724 | | 0.94 | | + | | # ATAATTA | | | | |  | | | |
| Dpulex_dsx1-a | | | | MatScan | | | ind | | 718 | | | 724 | | 0.91 | | + | | # ATAATTA | | | | |  | | | |
| Dpulex_dsx1-a | | | | MatScan | | | lab | | 718 | | | 724 | | 0.89 | | + | | # ATAATTA | | | | |  | | | |
| Dpulex_dsx1-a | | | | MatScan | | | Lim1 | | 718 | | | 724 | | 0.87 | | + | | # ATAATTA | | | | |  | | | |
| Dpulex_dsx1-a | | | | MatScan | | | Lim3 | | 718 | | | 724 | | 0.95 | | + | | # ATAATTA | | | | |  | | | |
| Dpulex_dsx1-a | | | | MatScan | | | NK7.1 | | 718 | | | 724 | | 0.86 | | + | | # ATAATTA | | | | |  | | | |
| Dpulex_dsx1-a | | | | MatScan | | | OdsH | | 718 | | | 724 | | 0.92 | | + | | # ATAATTA | | | | |  | | | |
| Dpulex_dsx1-a | | | | MatScan | | | OdsH | | 718 | | | 724 | | 0.92 | | + | | # ATAATTA | | | | |  | | | |
| Dpulex_dsx1-a | | | | MatScan | | | otp | | 718 | | | 724 | | 0.92 | | + | | # ATAATTA | | | | |  | | | |
| Dpulex_dsx1-a | | | | MatScan | | | pb | | 718 | | | 724 | | 0.95 | | + | | # ATAATTA | | | | |  | | | |
| Dpulex_dsx1-a | | | | MatScan | | | PHDP | | 718 | | | 724 | | 0.97 | | + | | # ATAATTA | | | | |  | | | |
| Dpulex_dsx1-a | | | | MatScan | | | Pph13 | | 718 | | | 724 | | 0.98 | | + | | # ATAATTA | | | | |  | | | |
| Dpulex_dsx1-a | | | | MatScan | | | repo | | 718 | | | 724 | | 0.9 | | + | | # ATAATTA | | | | |  | | | |
| Dpulex_dsx1-a | | | | MatScan | | | ro | | 718 | | | 724 | | 0.92 | | + | | # ATAATTA | | | | |  | | | |
| Dpulex_dsx1-a | | | | MatScan | | | Rx | | 718 | | | 724 | | 0.94 | | + | | # ATAATTA | | | | |  | | | |
| Dpulex_dsx1-a | | | | MatScan | | | Scr | | 718 | | | 724 | | 0.85 | | + | | # ATAATTA | | | | |  | | | |
| Dpulex_dsx1-a | | | | MatScan | | | slou | | 718 | | | 724 | | 0.92 | | + | | # ATAATTA | | | | |  | | | |
| Dpulex_dsx1-a | | | | MatScan | | | unpg | | 718 | | | 724 | | 0.93 | | + | | # ATAATTA | | | | |  | | | |
| Dpulex_dsx1-a | | | | MatScan | | | Vsx1 | | 718 | | | 724 | | 0.92 | | + | | # ATAATTA | | | | |  | | | |
| Dpulex_dsx1-a | | | | MatScan | | | zen | | 718 | | | 724 | | 0.87 | | + | | # ATAATTA | | | | |  | | | |
| Dpulex_dsx1-a | | | | MatScan | | | zen2 | | 718 | | | 724 | | 0.95 | | + | | # ATAATTA | | | | |  | | | |
| Dpulex_dsx1-a | | | | MatScan | | | abd-A | | 719 | | | 725 | | 0.88 | | - | | # ATAATTA | | | | |  | | | |
| Dpulex_dsx1-a | | | | MatScan | | | al | | 719 | | | 725 | | 0.87 | | + | | # TAATTAT | | | | |  | | | |
| Dpulex_dsx1-a | | | | MatScan | | | Antp | | 719 | | | 725 | | 0.85 | | - | | # ATAATTA | | | | |  | | | |
| Dpulex_dsx1-a | | | | MatScan | | | ap | | 719 | | | 725 | | 0.93 | | - | | # ATAATTA | | | | |  | | | |
| Dpulex_dsx1-a | | | | MatScan | | | Awh | | 719 | | | 725 | | 0.92 | | - | | # ATAATTA | | | | |  | | | |
| Dpulex_dsx1-a | | | | MatScan | | | bsh | | 719 | | | 725 | | 0.88 | | - | | # ATAATTA | | | | |  | | | |
| Dpulex_dsx1-a | | | | MatScan | | | btn | | 719 | | | 725 | | 0.89 | | - | | # ATAATTA | | | | |  | | | |
| Dpulex_dsx1-a | | | | MatScan | | | CG11294 | | 719 | | | 725 | | 0.88 | | - | | # ATAATTA | | | | |  | | | |
| Dpulex_dsx1-a | | | | MatScan | | | CG13424 | | 719 | | | 725 | | 0.88 | | - | | # ATAATTA | | | | |  | | | |
| Dpulex_dsx1-a | | | | MatScan | | | CG15696 | | 719 | | | 725 | | 0.88 | | - | | # ATAATTA | | | | |  | | | |
| Dpulex_dsx1-a | | | | MatScan | | | CG18599 | | 719 | | | 725 | | 0.96 | | - | | # ATAATTA | | | | |  | | | |
| Dpulex_dsx1-a | | | | MatScan | | | CG32105 | | 719 | | | 725 | | 0.91 | | - | | # ATAATTA | | | | |  | | | |
| Dpulex_dsx1-a | | | | MatScan | | | CG32532 | | 719 | | | 725 | | 0.92 | | - | | # ATAATTA | | | | |  | | | |
| Dpulex_dsx1-a | | | | MatScan | | | CG4328 | | 719 | | | 725 | | 0.97 | | - | | # ATAATTA | | | | |  | | | |
| Dpulex_dsx1-a | | | | MatScan | | | CG7056 | | 719 | | | 726 | | 0.87 | | - | | # TATAATTA | | | | |  | | | |
| Dpulex_dsx1-a | | | | MatScan | | | CG9876 | | 719 | | | 725 | | 0.94 | | - | | # ATAATTA | | | | |  | | | |
| Dpulex_dsx1-a | | | | MatScan | | | Dll | | 719 | | | 725 | | 0.95 | | + | | # TAATTAT | | | | |  | | | |
| Dpulex_dsx1-a | | | | MatScan | | | E5 | | 719 | | | 725 | | 0.95 | | - | | # ATAATTA | | | | |  | | | |
| Dpulex_dsx1-a | | | | MatScan | | | ems | | 719 | | | 725 | | 0.93 | | - | | # ATAATTA | | | | |  | | | |
| Dpulex_dsx1-a | | | | MatScan | | | en | | 719 | | | 725 | | 0.93 | | - | | # ATAATTA | | | | |  | | | |
| Dpulex_dsx1-a | | | | MatScan | | | eve | | 719 | | | 725 | | 0.93 | | - | | # ATAATTA | | | | |  | | | |
| Dpulex_dsx1-a | | | | MatScan | | | exex | | 719 | | | 725 | | 0.93 | | - | | # ATAATTA | | | | |  | | | |
| Dpulex_dsx1-a | | | | MatScan | | | exex | | 719 | | | 725 | | 0.93 | | - | | # ATAATTA | | | | |  | | | |
| Dpulex_dsx1-a | | | | MatScan | | | ftz | | 719 | | | 725 | | 0.87 | | - | | # ATAATTA | | | | |  | | | |
| Dpulex_dsx1-a | | | | MatScan | | | H2.0 | | 719 | | | 725 | | 0.92 | | - | | # ATAATTA | | | | |  | | | |
| Dpulex_dsx1-a | | | | MatScan | | | hbn | | 719 | | | 725 | | 0.91 | | - | | # ATAATTA | | | | |  | | | |
| Dpulex_dsx1-a | | | | MatScan | | | HGTX | | 719 | | | 725 | | 0.94 | | - | | # ATAATTA | | | | |  | | | |
| Dpulex_dsx1-a | | | | MatScan | | | ind | | 719 | | | 725 | | 0.91 | | - | | # ATAATTA | | | | |  | | | |
| Dpulex_dsx1-a | | | | MatScan | | | inv | | 719 | | | 726 | | 0.93 | | - | | # TATAATTA | | | | |  | | | |
| Dpulex_dsx1-a | | | | MatScan | | | lab | | 719 | | | 725 | | 0.89 | | - | | # ATAATTA | | | | |  | | | |
| Dpulex_dsx1-a | | | | MatScan | | | lbe | | 719 | | | 724 | | 0.99 | | - | | # TAATTA | | | | |  | | | |
| Dpulex_dsx1-a | | | | MatScan | | | lbe | | 719 | | | 724 | | 0.99 | | + | | # TAATTA | | | | |  | | | |
| Dpulex_dsx1-a | | | | MatScan | | | lbl | | 719 | | | 724 | | 1 | | - | | # TAATTA | | | | |  | | | |
| Dpulex_dsx1-a | | | | MatScan | | | lbl | | 719 | | | 724 | | 1 | | + | | # TAATTA | | | | |  | | | |
| Dpulex_dsx1-a | | | | MatScan | | | Lim1 | | 719 | | | 725 | | 0.87 | | - | | # ATAATTA | | | | |  | | | |
| Dpulex_dsx1-a | | | | MatScan | | | Lim3 | | 719 | | | 725 | | 0.95 | | - | | # ATAATTA | | | | |  | | | |
| Dpulex_dsx1-a | | | | MatScan | | | NK7.1 | | 719 | | | 725 | | 0.86 | | - | | # ATAATTA | | | | |  | | | |
| Dpulex_dsx1-a | | | | MatScan | | | Oct | | 719 | | | 726 | | 0.98 | | - | | # TATAATTA | | | | |  | | | |
| Dpulex_dsx1-a | | | | MatScan | | | OdsH | | 719 | | | 725 | | 0.92 | | - | | # ATAATTA | | | | |  | | | |
| Dpulex_dsx1-a | | | | MatScan | | | OdsH | | 719 | | | 725 | | 0.92 | | - | | # ATAATTA | | | | |  | | | |
| Dpulex_dsx1-a | | | | MatScan | | | otp | | 719 | | | 725 | | 0.92 | | - | | # ATAATTA | | | | |  | | | |
| Dpulex_dsx1-a | | | | MatScan | | | pb | | 719 | | | 725 | | 0.95 | | - | | # ATAATTA | | | | |  | | | |
| Dpulex_dsx1-a | | | | MatScan | | | PHDP | | 719 | | | 725 | | 0.97 | | - | | # ATAATTA | | | | |  | | | |
| Dpulex_dsx1-a | | | | MatScan | | | Pph13 | | 719 | | | 725 | | 0.98 | | - | | # ATAATTA | | | | |  | | | |
| Dpulex_dsx1-a | | | | MatScan | | | repo | | 719 | | | 725 | | 0.9 | | - | | # ATAATTA | | | | |  | | | |
| Dpulex_dsx1-a | | | | MatScan | | | ro | | 719 | | | 725 | | 0.92 | | - | | # ATAATTA | | | | |  | | | |
| Dpulex_dsx1-a | | | | MatScan | | | Rx | | 719 | | | 725 | | 0.94 | | - | | # ATAATTA | | | | |  | | | |
| Dpulex_dsx1-a | | | | MatScan | | | Scr | | 719 | | | 725 | | 0.85 | | - | | # ATAATTA | | | | |  | | | |
| Dpulex_dsx1-a | | | | MatScan | | | slou | | 719 | | | 725 | | 0.92 | | - | | # ATAATTA | | | | |  | | | |
| Dpulex_dsx1-a | | | | MatScan | | | unpg | | 719 | | | 725 | | 0.93 | | - | | # ATAATTA | | | | |  | | | |
| Dpulex_dsx1-a | | | | MatScan | | | Vsx1 | | 719 | | | 725 | | 0.92 | | - | | # ATAATTA | | | | |  | | | |
| Dpulex_dsx1-a | | | | MatScan | | | zen | | 719 | | | 725 | | 0.87 | | - | | # ATAATTA | | | | |  | | | |
| Dpulex_dsx1-a | | | | MatScan | | | zen2 | | 719 | | | 725 | | 0.95 | | - | | # ATAATTA | | | | |  | | | |
| Dpulex_dsx1-a | | | | MatScan | | | Zeste | | 722 | | | 737 | | 0.87 | | + | | # TTATATGAGTGTGTGT | | | | |  | | | |
| Dpulex_dsx1-a | | | | MatScan | | | z | | 726 | | | 735 | | 0.9 | | + | | # ATGAGTGTGT | | | | |  | | | |
| Dpulex_dsx1-a | | | | MatScan | | | ara | | 735 | | | 739 | | 0.91 | | - | | # ATACA | | | | |  | | | |
| Dpulex_dsx1-a | | | | MatScan | | | mirr | | 735 | | | 739 | | 0.89 | | - | | # ATACA | | | | |  | | | |
| Dpulex_dsx1-a | | | | MatScan | | | vvl | | 735 | | | 740 | | 0.88 | | - | | # TATACA | | | | |  | | | |
| Dpulex_dsx1-a | | | | MatScan | | | vvl | | 739 | | | 744 | | 0.98 | | + | | # TATTCA | | | | |  | | | |
| Dpulex_dsx1-a | | | | MatScan | | | odd | | 772 | | | 782 | | 0.85 | | - | | # AAAAGTAGCAT | | | | |  | | | |
| Dpulex_dsx1-a | | | | MatScan | | | hb | | 785 | | | 794 | | 0.88 | | - | | # TAACAAAAAA | | | | |  | | | |
| Dpulex_dsx1-a | | | | MatScan | | | pan | | 787 | | | 794 | | 0.85 | | + | | # TTTTGTTA | | | | |  | | | |
| Dpulex_dsx1-a | | | | MatScan | | | BR-C | | 788 | | | 803 | | 0.87 | | + | | # TTTGTTACTATTTTCT | | | | |  | | | |
| Dpulex_dsx1-a | | | | MatScan | | | lbe | | 789 | | | 794 | | 0.94 | | - | | # TAACAA | | | | |  | | | |
| Dpulex_dsx1-a | | | | MatScan | | | ara | | 790 | | | 794 | | 1 | | - | | # TAACA | | | | |  | | | |
| Dpulex_dsx1-a | | | | MatScan | | | caup | | 790 | | | 794 | | 1 | | - | | # TAACA | | | | |  | | | |
| Dpulex_dsx1-a | | | | MatScan | | | mirr | | 790 | | | 794 | | 0.99 | | - | | # TAACA | | | | |  | | | |
| Dpulex_dsx1-a | | | | MatScan | | | br_Z2 | | 793 | | | 800 | | 1 | | + | | # TACTATTT | | | | |  | | | |
| Dpulex_dsx1-a | | | | MatScan | | | Lag1 | | 810 | | | 816 | | 0.87 | | + | | # CTACTAT | | | | |  | | | |
| Dpulex_dsx1-a | | | | MatScan | | | ttk | | 812 | | | 820 | | 0.87 | | - | | # AAGGATAGT | | | | |  | | | |
| Dpulex_dsx1-a | | | | MatScan | | | Eip74EF | | 818 | | | 824 | | 1 | | - | | # CCGGAAG | | | | |  | | | |
| Dpulex_dsx1-a | | | | MatScan | | | Lag1 | | 830 | | | 836 | | 0.86 | | - | | # CTCCCAA | | | | |  | | | |
| Dpulex_dsx1-a | | | | MatScan | | | Abd-B | | 839 | | | 845 | | 0.93 | | - | | # TTTATGG | | | | |  | | | |
| Dpulex_dsx1-a | | | | MatScan | | | cad | | 839 | | | 845 | | 0.92 | | - | | # TTTATGG | | | | |  | | | |
| Dpulex_dsx1-a | | | | MatScan | | | CG42234 | | 839 | | | 845 | | 0.87 | | - | | # TTTATGG | | | | |  | | | |
| Dpulex_dsx1-a | | | | MatScan | | | CG4328 | | 839 | | | 845 | | 0.88 | | - | | # TTTATGG | | | | |  | | | |
| Dpulex_dsx1-a | | | | MatScan | | | Deaf1 | | 848 | | | 853 | | 0.98 | | - | | # TTCGTC | | | | |  | | | |
| Dpulex_dsx1-a | | | | MatScan | | | onecut | | 852 | | | 858 | | 0.89 | | - | | # CTGATTT | | | | |  | | | |
| Dpulex_dsx1-a | | | | MatScan | | | slp1 | | 860 | | | 870 | | 0.91 | | - | | # ATGTTTATACT | | | | |  | | | |
| Dpulex_dsx1-a | | | | MatScan | | | TATA | | 861 | | | 875 | | 0.86 | | + | | # GTATAAACATGGTGG | | | | |  | | | |
| Dpulex_dsx1-a | | | | MatScan | | | lbe | | 864 | | | 869 | | 0.86 | | + | | # TAAACA | | | | |  | | | |
| Dpulex_dsx1-a | | | | MatScan | | | ara | | 865 | | | 869 | | 0.99 | | + | | # AAACA | | | | |  | | | |
| Dpulex_dsx1-a | | | | MatScan | | | caup | | 865 | | | 869 | | 0.9 | | + | | # AAACA | | | | |  | | | |
| Dpulex_dsx1-a | | | | MatScan | | | mirr | | 865 | | | 869 | | 1 | | + | | # AAACA | | | | |  | | | |
| Dpulex_dsx1-a | | | | MatScan | | | Lag1 | | 869 | | | 875 | | 0.91 | | - | | # CCACCAT | | | | |  | | | |
| Dpulex_dsx1-a | | | | MatScan | | | Optix | | 885 | | | 889 | | 1 | | + | | # TGATA | | | | |  | | | |
| Dpulex_dsx1-a | | | | MatScan | | | Six4 | | 885 | | | 890 | | 1 | | + | | # TGATAC | | | | |  | | | |
| Dpulex_dsx1-a | | | | MatScan | | | so | | 885 | | | 890 | | 1 | | + | | # TGATAC | | | | |  | | | |
| Dpulex_dsx1-a | | | | MatScan | | | br_Z2 | | 888 | | | 895 | | 0.91 | | + | | # TACTATTC | | | | |  | | | |
| Dpulex_dsx1-a | | | | MatScan | | | Deaf1 | | 893 | | | 898 | | 0.87 | | + | | # TTCGCC | | | | |  | | | |
| Dpulex_dsx1-a | | | | MatScan | | | Deaf1 | | 896 | | | 901 | | 0.89 | | - | | # CTCGGC | | | | |  | | | |
| Dpulex_dsx1-a | | | | MatScan | | | Deaf1 | | 907 | | | 912 | | 0.87 | | - | | # TTCGAC | | | | |  | | | |
| Dpulex_dsx1-a | | | | MatScan | | | dl_1 | | 914 | | | 925 | | 0.86 | | + | | # CGGCTTTTTCCT | | | | |  | | | |
| Dpulex_dsx1-a | | | | MatScan | | | dl | | 915 | | | 925 | | 0.86 | | + | | # GGCTTTTTCCT | | | | |  | | | |
| Dpulex_dsx1-a | | | | MatScan | | | ara | | 965 | | | 969 | | 0.89 | | - | | # CAACA | | | | |  | | | |
| Dpulex_dsx1-a | | | | MatScan | | | caup | | 965 | | | 969 | | 0.87 | | - | | # CAACA | | | | |  | | | |
| Dpulex_dsx1-a | | | | MatScan | | | mirr | | 965 | | | 969 | | 0.89 | | - | | # CAACA | | | | |  | | | |
| Dpulex_dsx1-a | | | | MatScan | | | ara | | 979 | | | 983 | | 0.99 | | - | | # AAACA | | | | |  | | | |
| Dpulex_dsx1-a | | | | MatScan | | | caup | | 979 | | | 983 | | 0.9 | | - | | # AAACA | | | | |  | | | |
| Dpulex_dsx1-a | | | | MatScan | | | mirr | | 979 | | | 983 | | 1 | | - | | # AAACA | | | | |  | | | |
| Dpulex_dsx1-a | | | | MatScan | | | brk | | 984 | | | 991 | | 0.86 | | - | | # GTGGCACC | | | | |  | | | |
