# Supplementary material for: Molecular cloning of doublesex genes of four cladocera (water flea) species
Source: BMC Genomics. 2013 Apr 10;14:239. doi: 10.1186/1471-2164-14-239 (PMC3637828; doi:10.1186/1471-2164-14-239)
Supplement: Additional file 18 — DapmaDsx1-β TF-map. [file 1471-2164-14-239-S18.doc]

Supplemental Material 18. *DapmaDsx1-β* TF-map

| Column Descriptions | Promoter region ID - Species, dsx paralog number, and dsx transcript identifier  Name of program that generated results  Name of transcription factor identified  Start of transcription factor binding site (TFBS)  End of transcription factor binding site (TFBS)  Match score between known TFBS (from TFBS database) and identified Daphnia dsx promoter sequence motif  Strand on which TFBS was identified in sequence  Reading frame for CDS feature types (not used)  Sequence of transcription factor binding motif (from TFBS database) | | | | | | |
| --- | --- | --- | --- | --- | --- | --- | --- |
| Sequence ID |
| Source |
| Type (TF) |
| Start |
| End |
| Score |
| Strand |
| Phase |
| TF Binding Motif |
|  |  |  |  |  |  |  |  |
| **Sequence ID** | **Source** | **Type (TF)** | **Start** | **End** | **Score** | **Strand** | **TF Binding Motif** |
| Dmagna_dsx1-b | MatScan | exd | 14 | 21 | 0.88 | + | # CTTTGAAA |
| Dmagna_dsx1-b | MatScan | pan | 14 | 21 | 0.9 | + | # CTTTGAAA |
| Dmagna_dsx1-b | MatScan | Six4 | 17 | 22 | 0.92 | + | # TGAAAC |
| Dmagna_dsx1-b | MatScan | ara | 19 | 23 | 0.99 | + | # AAACA |
| Dmagna_dsx1-b | MatScan | caup | 19 | 23 | 0.9 | + | # AAACA |
| Dmagna_dsx1-b | MatScan | mirr | 19 | 23 | 1 | + | # AAACA |
| Dmagna_dsx1-b | MatScan | Deaf1 | 41 | 46 | 0.96 | + | # TTCGTT |
| Dmagna_dsx1-b | MatScan | Deaf1 | 45 | 50 | 1 | + | # TTCGTG |
| Dmagna_dsx1-b | MatScan | ara | 49 | 53 | 0.91 | - | # TTACA |
| Dmagna_dsx1-b | MatScan | caup | 49 | 53 | 0.87 | - | # TTACA |
| Dmagna_dsx1-b | MatScan | mirr | 49 | 53 | 0.88 | - | # TTACA |
| Dmagna_dsx1-b | MatScan | exd | 55 | 62 | 0.99 | + | # ATTTGACA |
| Dmagna_dsx1-b | MatScan | CG11617 | 57 | 63 | 0.92 | + | # TTGACAA |
| Dmagna_dsx1-b | MatScan | achi | 58 | 63 | 0.93 | + | # TGACAA |
| Dmagna_dsx1-b | MatScan | caup | 58 | 62 | 0.85 | + | # TGACA |
| Dmagna_dsx1-b | MatScan | hth | 58 | 63 | 0.91 | + | # TGACAA |
| Dmagna_dsx1-b | MatScan | vis | 58 | 63 | 0.95 | + | # TGACAA |
| Dmagna_dsx1-b | MatScan | ara | 68 | 72 | 0.91 | + | # ATACA |
| Dmagna_dsx1-b | MatScan | mirr | 68 | 72 | 0.89 | + | # ATACA |
| Dmagna_dsx1-b | MatScan | Ttk | 74 | 81 | 0.9 | - | # GATCCTGC |
| Dmagna_dsx1-b | MatScan | ara | 92 | 96 | 0.93 | - | # GAACA |
| Dmagna_dsx1-b | MatScan | caup | 92 | 96 | 0.88 | - | # GAACA |
| Dmagna_dsx1-b | MatScan | mirr | 92 | 96 | 0.89 | - | # GAACA |
| Dmagna_dsx1-b | MatScan | Eip74EF | 93 | 99 | 0.91 | - | # CCGGAAC |
| Dmagna_dsx1-b | MatScan | lbe | 102 | 107 | 0.87 | - | # TAAGCA |
| Dmagna_dsx1-b | MatScan | mtTFA | 102 | 112 | 0.86 | + | # TGCTTATGATC |
| Dmagna_dsx1-b | MatScan | vvl | 102 | 107 | 0.9 | - | # TAAGCA |
| Dmagna_dsx1-b | MatScan | H2.0 | 104 | 110 | 0.85 | + | # CTTATGA |
| Dmagna_dsx1-b | MatScan | twi | 115 | 126 | 0.88 | + | # CAGCATGTGTTC |
| Dmagna_dsx1-b | MatScan | ara | 122 | 126 | 0.93 | - | # GAACA |
| Dmagna_dsx1-b | MatScan | caup | 122 | 126 | 0.88 | - | # GAACA |
| Dmagna_dsx1-b | MatScan | mirr | 122 | 126 | 0.89 | - | # GAACA |
| Dmagna_dsx1-b | MatScan | Deaf1 | 124 | 129 | 0.87 | + | # TTCGAC |
| Dmagna_dsx1-b | MatScan | CG4328 | 129 | 135 | 0.89 | - | # AATATTG |
| Dmagna_dsx1-b | MatScan | ara | 136 | 140 | 0.89 | - | # CAACA |
| Dmagna_dsx1-b | MatScan | caup | 136 | 140 | 0.87 | - | # CAACA |
| Dmagna_dsx1-b | MatScan | mirr | 136 | 140 | 0.89 | - | # CAACA |
| Dmagna_dsx1-b | MatScan | Deaf1 | 169 | 174 | 0.98 | + | # TTCGTC |
| Dmagna_dsx1-b | MatScan | Deaf1 | 176 | 181 | 0.98 | - | # TTCGGC |
| Dmagna_dsx1-b | MatScan | Abd-B | 185 | 191 | 0.87 | + | # TTGATGA |
| Dmagna_dsx1-b | MatScan | CG42234 | 185 | 191 | 0.91 | + | # TTGATGA |
| Dmagna_dsx1-b | MatScan | H2.0 | 185 | 191 | 0.87 | + | # TTGATGA |
| Dmagna_dsx1-b | MatScan | brk | 191 | 198 | 0.9 | - | # TTGGCGCT |
| Dmagna_dsx1-b | MatScan | slbo | 209 | 216 | 0.88 | - | # GTTGCAAA |
| Dmagna_dsx1-b | MatScan | ems | 230 | 236 | 0.87 | - | # TAAATGA |
| Dmagna_dsx1-b | MatScan | ftz | 230 | 236 | 0.85 | - | # TAAATGA |
| Dmagna_dsx1-b | MatScan | B-H1 | 231 | 237 | 0.96 | - | # TTAAATG |
| Dmagna_dsx1-b | MatScan | B-H2 | 231 | 237 | 0.91 | - | # TTAAATG |
| Dmagna_dsx1-b | MatScan | C15 | 231 | 237 | 0.94 | - | # TTAAATG |
| Dmagna_dsx1-b | MatScan | CG11085 | 231 | 237 | 0.86 | - | # TTAAATG |
| Dmagna_dsx1-b | MatScan | CG34031 | 231 | 237 | 0.87 | - | # TTAAATG |
| Dmagna_dsx1-b | MatScan | Hmx | 231 | 237 | 0.86 | - | # TTAAATG |
| Dmagna_dsx1-b | MatScan | NK7.1 | 231 | 237 | 0.89 | - | # TTAAATG |
| Dmagna_dsx1-b | MatScan | B-H1 | 234 | 240 | 0.86 | + | # TTAAAAG |
| Dmagna_dsx1-b | MatScan | exd | 239 | 246 | 0.88 | + | # AGTTGACA |
| Dmagna_dsx1-b | MatScan | CG11617 | 241 | 247 | 0.92 | + | # TTGACAC |
| Dmagna_dsx1-b | MatScan | achi | 242 | 247 | 0.9 | + | # TGACAC |
| Dmagna_dsx1-b | MatScan | caup | 242 | 246 | 0.85 | + | # TGACA |
| Dmagna_dsx1-b | MatScan | hth | 242 | 247 | 0.91 | + | # TGACAC |
| Dmagna_dsx1-b | MatScan | Six4 | 242 | 247 | 0.98 | + | # TGACAC |
| Dmagna_dsx1-b | MatScan | vis | 242 | 247 | 0.97 | + | # TGACAC |
| Dmagna_dsx1-b | MatScan | lbe | 251 | 256 | 0.86 | - | # TAAGAA |
| Dmagna_dsx1-b | MatScan | CG11617 | 254 | 260 | 0.99 | + | # TTAACAA |
| Dmagna_dsx1-b | MatScan | ara | 255 | 259 | 1 | + | # TAACA |
| Dmagna_dsx1-b | MatScan | caup | 255 | 259 | 1 | + | # TAACA |
| Dmagna_dsx1-b | MatScan | lbe | 255 | 260 | 0.94 | + | # TAACAA |
| Dmagna_dsx1-b | MatScan | mirr | 255 | 259 | 0.99 | + | # TAACA |
| Dmagna_dsx1-b | MatScan | vvl | 261 | 266 | 0.87 | - | # CATGCA |
| Dmagna_dsx1-b | MatScan | Dfd | 267 | 282 | 0.87 | - | # CTTTTAATTAACACAC |
| Dmagna_dsx1-b | MatScan | CG11617 | 269 | 275 | 0.99 | - | # TTAACAC |
| Dmagna_dsx1-b | MatScan | Dfd | 269 | 284 | 0.86 | + | # GTGTTAATTAAAAGGT |
| Dmagna_dsx1-b | MatScan | ara | 270 | 274 | 1 | - | # TAACA |
| Dmagna_dsx1-b | MatScan | caup | 270 | 274 | 1 | - | # TAACA |
| Dmagna_dsx1-b | MatScan | dri | 270 | 279 | 0.91 | - | # TTAATTAACA |
| Dmagna_dsx1-b | MatScan | mirr | 270 | 274 | 0.99 | - | # TAACA |
| Dmagna_dsx1-b | MatScan | CG7056 | 271 | 278 | 0.92 | + | # GTTAATTA |
| Dmagna_dsx1-b | MatScan | inv | 271 | 278 | 0.92 | + | # GTTAATTA |
| Dmagna_dsx1-b | MatScan | Oct | 271 | 278 | 0.98 | + | # GTTAATTA |
| Dmagna_dsx1-b | MatScan | Ubx | 271 | 278 | 0.95 | + | # GTTAATTA |
| Dmagna_dsx1-b | MatScan | Vsx2 | 271 | 279 | 0.9 | + | # GTTAATTAA |
| Dmagna_dsx1-b | MatScan | abd-A | 272 | 278 | 1 | + | # TTAATTA |
| Dmagna_dsx1-b | MatScan | Abd-B | 272 | 278 | 0.88 | + | # TTAATTA |
| Dmagna_dsx1-b | MatScan | al | 272 | 278 | 1 | - | # TAATTAA |
| Dmagna_dsx1-b | MatScan | Antp | 272 | 278 | 0.98 | + | # TTAATTA |
| Dmagna_dsx1-b | MatScan | ap | 272 | 278 | 0.97 | + | # TTAATTA |
| Dmagna_dsx1-b | MatScan | Awh | 272 | 278 | 1 | + | # TTAATTA |
| Dmagna_dsx1-b | MatScan | bsh | 272 | 278 | 0.98 | + | # TTAATTA |
| Dmagna_dsx1-b | MatScan | btn | 272 | 278 | 0.95 | + | # TTAATTA |
| Dmagna_dsx1-b | MatScan | C15 | 272 | 278 | 1 | + | # TTAATTA |
| Dmagna_dsx1-b | MatScan | cad | 272 | 278 | 0.9 | + | # TTAATTA |
| Dmagna_dsx1-b | MatScan | CG11085 | 272 | 278 | 0.86 | + | # TTAATTA |
| Dmagna_dsx1-b | MatScan | CG11294 | 272 | 278 | 1 | + | # TTAATTA |
| Dmagna_dsx1-b | MatScan | CG13424 | 272 | 278 | 0.96 | + | # TTAATTA |
| Dmagna_dsx1-b | MatScan | CG15696 | 272 | 278 | 1 | + | # TTAATTA |
| Dmagna_dsx1-b | MatScan | CG18599 | 272 | 278 | 1 | + | # TTAATTA |
| Dmagna_dsx1-b | MatScan | CG32105 | 272 | 278 | 1 | + | # TTAATTA |
| Dmagna_dsx1-b | MatScan | CG32532 | 272 | 278 | 1 | + | # TTAATTA |
| Dmagna_dsx1-b | MatScan | CG34031 | 272 | 278 | 0.88 | + | # TTAATTA |
| Dmagna_dsx1-b | MatScan | CG42234 | 272 | 278 | 0.98 | + | # TTAATTA |
| Dmagna_dsx1-b | MatScan | CG4328 | 272 | 278 | 0.97 | + | # TTAATTA |
| Dmagna_dsx1-b | MatScan | CG9876 | 272 | 278 | 0.97 | + | # TTAATTA |
| Dmagna_dsx1-b | MatScan | Dfd | 272 | 278 | 0.9 | + | # TTAATTA |
| Dmagna_dsx1-b | MatScan | Dll | 272 | 278 | 0.91 | - | # TAATTAA |
| Dmagna_dsx1-b | MatScan | dri | 272 | 281 | 0.93 | + | # TTAATTAAAA |
| Dmagna_dsx1-b | MatScan | E5 | 272 | 278 | 1 | + | # TTAATTA |
| Dmagna_dsx1-b | MatScan | ems | 272 | 278 | 0.99 | + | # TTAATTA |
| Dmagna_dsx1-b | MatScan | en | 272 | 278 | 1 | + | # TTAATTA |
| Dmagna_dsx1-b | MatScan | eve | 272 | 278 | 0.98 | + | # TTAATTA |
| Dmagna_dsx1-b | MatScan | exex | 272 | 278 | 0.97 | + | # TTAATTA |
| Dmagna_dsx1-b | MatScan | exex | 272 | 278 | 0.97 | + | # TTAATTA |
| Dmagna_dsx1-b | MatScan | ftz | 272 | 278 | 1 | + | # TTAATTA |
| Dmagna_dsx1-b | MatScan | H2.0 | 272 | 278 | 1 | + | # TTAATTA |
| Dmagna_dsx1-b | MatScan | hbn | 272 | 278 | 1 | + | # TTAATTA |
| Dmagna_dsx1-b | MatScan | HGTX | 272 | 278 | 1 | + | # TTAATTA |
| Dmagna_dsx1-b | MatScan | Hmx | 272 | 278 | 0.91 | + | # TTAATTA |
| Dmagna_dsx1-b | MatScan | ind | 272 | 278 | 0.98 | + | # TTAATTA |
| Dmagna_dsx1-b | MatScan | lab | 272 | 278 | 1 | + | # TTAATTA |
| Dmagna_dsx1-b | MatScan | Lim1 | 272 | 278 | 1 | + | # TTAATTA |
| Dmagna_dsx1-b | MatScan | Lim3 | 272 | 278 | 1 | + | # TTAATTA |
| Dmagna_dsx1-b | MatScan | NK7.1 | 272 | 278 | 0.95 | + | # TTAATTA |
| Dmagna_dsx1-b | MatScan | OdsH | 272 | 278 | 0.97 | + | # TTAATTA |
| Dmagna_dsx1-b | MatScan | OdsH | 272 | 278 | 0.97 | + | # TTAATTA |
| Dmagna_dsx1-b | MatScan | otp | 272 | 278 | 1 | + | # TTAATTA |
| Dmagna_dsx1-b | MatScan | pb | 272 | 278 | 1 | + | # TTAATTA |
| Dmagna_dsx1-b | MatScan | PHDP | 272 | 278 | 1 | + | # TTAATTA |
| Dmagna_dsx1-b | MatScan | Pph13 | 272 | 278 | 0.97 | + | # TTAATTA |
| Dmagna_dsx1-b | MatScan | repo | 272 | 278 | 1 | + | # TTAATTA |
| Dmagna_dsx1-b | MatScan | ro | 272 | 278 | 0.96 | + | # TTAATTA |
| Dmagna_dsx1-b | MatScan | Rx | 272 | 278 | 0.99 | + | # TTAATTA |
| Dmagna_dsx1-b | MatScan | Scr | 272 | 278 | 0.93 | + | # TTAATTA |
| Dmagna_dsx1-b | MatScan | slou | 272 | 278 | 1 | + | # TTAATTA |
| Dmagna_dsx1-b | MatScan | tup | 272 | 278 | 0.88 | + | # TTAATTA |
| Dmagna_dsx1-b | MatScan | unc-4 | 272 | 278 | 0.94 | + | # TTAATTA |
| Dmagna_dsx1-b | MatScan | unpg | 272 | 278 | 1 | + | # TTAATTA |
| Dmagna_dsx1-b | MatScan | Vsx1 | 272 | 278 | 1 | + | # TTAATTA |
| Dmagna_dsx1-b | MatScan | Vsx2 | 272 | 280 | 0.91 | - | # TTTAATTAA |
| Dmagna_dsx1-b | MatScan | zen | 272 | 278 | 0.9 | + | # TTAATTA |
| Dmagna_dsx1-b | MatScan | zen2 | 272 | 278 | 1 | + | # TTAATTA |
| Dmagna_dsx1-b | MatScan | abd-A | 273 | 279 | 1 | - | # TTAATTA |
| Dmagna_dsx1-b | MatScan | Abd-B | 273 | 279 | 0.88 | - | # TTAATTA |
| Dmagna_dsx1-b | MatScan | al | 273 | 279 | 1 | + | # TAATTAA |
| Dmagna_dsx1-b | MatScan | Antp | 273 | 279 | 0.98 | - | # TTAATTA |
| Dmagna_dsx1-b | MatScan | ap | 273 | 279 | 0.97 | - | # TTAATTA |
| Dmagna_dsx1-b | MatScan | Awh | 273 | 279 | 1 | - | # TTAATTA |
| Dmagna_dsx1-b | MatScan | bsh | 273 | 279 | 0.98 | - | # TTAATTA |
| Dmagna_dsx1-b | MatScan | btn | 273 | 279 | 0.95 | - | # TTAATTA |
| Dmagna_dsx1-b | MatScan | C15 | 273 | 279 | 1 | - | # TTAATTA |
| Dmagna_dsx1-b | MatScan | cad | 273 | 279 | 0.9 | - | # TTAATTA |
| Dmagna_dsx1-b | MatScan | CG11085 | 273 | 279 | 0.86 | - | # TTAATTA |
| Dmagna_dsx1-b | MatScan | CG11294 | 273 | 279 | 1 | - | # TTAATTA |
| Dmagna_dsx1-b | MatScan | CG13424 | 273 | 279 | 0.96 | - | # TTAATTA |
| Dmagna_dsx1-b | MatScan | CG15696 | 273 | 279 | 1 | - | # TTAATTA |
| Dmagna_dsx1-b | MatScan | CG18599 | 273 | 279 | 1 | - | # TTAATTA |
| Dmagna_dsx1-b | MatScan | CG32105 | 273 | 279 | 1 | - | # TTAATTA |
| Dmagna_dsx1-b | MatScan | CG32532 | 273 | 279 | 1 | - | # TTAATTA |
| Dmagna_dsx1-b | MatScan | CG34031 | 273 | 279 | 0.88 | - | # TTAATTA |
| Dmagna_dsx1-b | MatScan | CG42234 | 273 | 279 | 0.98 | - | # TTAATTA |
| Dmagna_dsx1-b | MatScan | CG4328 | 273 | 279 | 0.97 | - | # TTAATTA |
| Dmagna_dsx1-b | MatScan | CG7056 | 273 | 280 | 1 | - | # TTTAATTA |
| Dmagna_dsx1-b | MatScan | CG9876 | 273 | 279 | 0.97 | - | # TTAATTA |
| Dmagna_dsx1-b | MatScan | Dfd | 273 | 279 | 0.9 | - | # TTAATTA |
| Dmagna_dsx1-b | MatScan | Dll | 273 | 279 | 0.91 | + | # TAATTAA |
| Dmagna_dsx1-b | MatScan | E5 | 273 | 279 | 1 | - | # TTAATTA |
| Dmagna_dsx1-b | MatScan | ems | 273 | 279 | 0.99 | - | # TTAATTA |
| Dmagna_dsx1-b | MatScan | en | 273 | 279 | 1 | - | # TTAATTA |
| Dmagna_dsx1-b | MatScan | eve | 273 | 279 | 0.98 | - | # TTAATTA |
| Dmagna_dsx1-b | MatScan | exex | 273 | 279 | 0.97 | - | # TTAATTA |
| Dmagna_dsx1-b | MatScan | exex | 273 | 279 | 0.97 | - | # TTAATTA |
| Dmagna_dsx1-b | MatScan | ftz | 273 | 279 | 1 | - | # TTAATTA |
| Dmagna_dsx1-b | MatScan | H2.0 | 273 | 279 | 1 | - | # TTAATTA |
| Dmagna_dsx1-b | MatScan | hbn | 273 | 279 | 1 | - | # TTAATTA |
| Dmagna_dsx1-b | MatScan | HGTX | 273 | 279 | 1 | - | # TTAATTA |
| Dmagna_dsx1-b | MatScan | Hmx | 273 | 279 | 0.91 | - | # TTAATTA |
| Dmagna_dsx1-b | MatScan | ind | 273 | 279 | 0.98 | - | # TTAATTA |
| Dmagna_dsx1-b | MatScan | inv | 273 | 280 | 0.97 | - | # TTTAATTA |
| Dmagna_dsx1-b | MatScan | lab | 273 | 279 | 1 | - | # TTAATTA |
| Dmagna_dsx1-b | MatScan | lbe | 273 | 278 | 0.99 | - | # TAATTA |
| Dmagna_dsx1-b | MatScan | lbe | 273 | 278 | 0.99 | + | # TAATTA |
| Dmagna_dsx1-b | MatScan | lbl | 273 | 278 | 1 | - | # TAATTA |
| Dmagna_dsx1-b | MatScan | lbl | 273 | 278 | 1 | + | # TAATTA |
| Dmagna_dsx1-b | MatScan | Lim1 | 273 | 279 | 1 | - | # TTAATTA |
| Dmagna_dsx1-b | MatScan | Lim3 | 273 | 279 | 1 | - | # TTAATTA |
| Dmagna_dsx1-b | MatScan | NK7.1 | 273 | 279 | 0.95 | - | # TTAATTA |
| Dmagna_dsx1-b | MatScan | Oct | 273 | 280 | 1 | - | # TTTAATTA |
| Dmagna_dsx1-b | MatScan | OdsH | 273 | 279 | 0.97 | - | # TTAATTA |
| Dmagna_dsx1-b | MatScan | OdsH | 273 | 279 | 0.97 | - | # TTAATTA |
| Dmagna_dsx1-b | MatScan | otp | 273 | 279 | 1 | - | # TTAATTA |
| Dmagna_dsx1-b | MatScan | pb | 273 | 279 | 1 | - | # TTAATTA |
| Dmagna_dsx1-b | MatScan | PHDP | 273 | 279 | 1 | - | # TTAATTA |
| Dmagna_dsx1-b | MatScan | Pph13 | 273 | 279 | 0.97 | - | # TTAATTA |
| Dmagna_dsx1-b | MatScan | repo | 273 | 279 | 1 | - | # TTAATTA |
| Dmagna_dsx1-b | MatScan | ro | 273 | 279 | 0.96 | - | # TTAATTA |
| Dmagna_dsx1-b | MatScan | Rx | 273 | 279 | 0.99 | - | # TTAATTA |
| Dmagna_dsx1-b | MatScan | Scr | 273 | 279 | 0.93 | - | # TTAATTA |
| Dmagna_dsx1-b | MatScan | slou | 273 | 279 | 1 | - | # TTAATTA |
| Dmagna_dsx1-b | MatScan | tup | 273 | 279 | 0.88 | - | # TTAATTA |
| Dmagna_dsx1-b | MatScan | Ubx | 273 | 280 | 1 | - | # TTTAATTA |
| Dmagna_dsx1-b | MatScan | unc-4 | 273 | 279 | 0.94 | - | # TTAATTA |
| Dmagna_dsx1-b | MatScan | unpg | 273 | 279 | 1 | - | # TTAATTA |
| Dmagna_dsx1-b | MatScan | Vsx1 | 273 | 279 | 1 | - | # TTAATTA |
| Dmagna_dsx1-b | MatScan | zen | 273 | 279 | 0.9 | - | # TTAATTA |
| Dmagna_dsx1-b | MatScan | zen2 | 273 | 279 | 1 | - | # TTAATTA |
| Dmagna_dsx1-b | MatScan | B-H1 | 276 | 282 | 0.86 | + | # TTAAAAG |
| Dmagna_dsx1-b | MatScan | achi | 303 | 308 | 1 | - | # TGACAG |
| Dmagna_dsx1-b | MatScan | CG11617 | 303 | 309 | 0.92 | - | # TTGACAG |
| Dmagna_dsx1-b | MatScan | hth | 303 | 308 | 1 | - | # TGACAG |
| Dmagna_dsx1-b | MatScan | vis | 303 | 308 | 1 | - | # TGACAG |
| Dmagna_dsx1-b | MatScan | caup | 304 | 308 | 0.85 | - | # TGACA |
| Dmagna_dsx1-b | MatScan | exd | 304 | 311 | 0.87 | - | # GATTGACA |
| Dmagna_dsx1-b | MatScan | ara | 313 | 317 | 0.91 | - | # ATACA |
| Dmagna_dsx1-b | MatScan | mirr | 313 | 317 | 0.89 | - | # ATACA |
| Dmagna_dsx1-b | MatScan | ct | 325 | 330 | 0.98 | - | # TTAAAC |
| Dmagna_dsx1-b | MatScan | CG7056 | 326 | 333 | 0.89 | + | # TTTAAGTA |
| Dmagna_dsx1-b | MatScan | al | 327 | 333 | 0.85 | - | # TACTTAA |
| Dmagna_dsx1-b | MatScan | bap | 327 | 333 | 0.94 | + | # TTAAGTA |
| Dmagna_dsx1-b | MatScan | C15 | 327 | 333 | 0.89 | + | # TTAAGTA |
| Dmagna_dsx1-b | MatScan | CG32105 | 327 | 333 | 0.85 | + | # TTAAGTA |
| Dmagna_dsx1-b | MatScan | lab | 327 | 333 | 0.85 | + | # TTAAGTA |
| Dmagna_dsx1-b | MatScan | Lim1 | 327 | 333 | 0.85 | + | # TTAAGTA |
| Dmagna_dsx1-b | MatScan | Ovo | 327 | 341 | 0.87 | + | # TTAAGTAACTGAAAC |
| Dmagna_dsx1-b | MatScan | al | 328 | 334 | 0.85 | + | # TAAGTAA |
| Dmagna_dsx1-b | MatScan | lbe | 328 | 333 | 0.92 | + | # TAAGTA |
| Dmagna_dsx1-b | MatScan | Lim1 | 328 | 334 | 0.85 | - | # TTACTTA |
| Dmagna_dsx1-b | MatScan | ovo | 330 | 338 | 0.95 | + | # AGTAACTGA |
| Dmagna_dsx1-b | MatScan | prd | 330 | 338 | 0.95 | + | # AGTAACTGA |
| Dmagna_dsx1-b | MatScan | ovo | 336 | 344 | 0.87 | + | # TGAAACAGA |
| Dmagna_dsx1-b | MatScan | prd | 336 | 344 | 0.87 | + | # TGAAACAGA |
| Dmagna_dsx1-b | MatScan | Six4 | 336 | 341 | 0.92 | + | # TGAAAC |
| Dmagna_dsx1-b | MatScan | ara | 338 | 342 | 0.99 | + | # AAACA |
| Dmagna_dsx1-b | MatScan | caup | 338 | 342 | 0.9 | + | # AAACA |
| Dmagna_dsx1-b | MatScan | mirr | 338 | 342 | 1 | + | # AAACA |
| Dmagna_dsx1-b | MatScan | Gsc | 342 | 347 | 0.94 | - | # TAATCT |
| Dmagna_dsx1-b | MatScan | oc | 342 | 347 | 0.85 | - | # TAATCT |
| Dmagna_dsx1-b | MatScan | CG4328 | 344 | 350 | 0.87 | + | # ATTATGA |
| Dmagna_dsx1-b | MatScan | H2.0 | 344 | 350 | 0.87 | + | # ATTATGA |
| Dmagna_dsx1-b | MatScan | H2.0 | 345 | 351 | 0.86 | - | # TTCATAA |
| Dmagna_dsx1-b | MatScan | vvl | 346 | 351 | 0.9 | + | # TATGAA |
| Dmagna_dsx1-b | MatScan | ct | 347 | 352 | 0.86 | + | # ATGAAC |
| Dmagna_dsx1-b | MatScan | ara | 349 | 353 | 0.93 | + | # GAACA |
| Dmagna_dsx1-b | MatScan | caup | 349 | 353 | 0.88 | + | # GAACA |
| Dmagna_dsx1-b | MatScan | mirr | 349 | 353 | 0.89 | + | # GAACA |
| Dmagna_dsx1-b | MatScan | Dfd | 356 | 371 | 0.94 | + | # TTAGCAATTACCTAGG |
| Dmagna_dsx1-b | MatScan | slbo | 356 | 363 | 0.91 | - | # ATTGCTAA |
| Dmagna_dsx1-b | MatScan | Dll | 359 | 365 | 0.99 | - | # TAATTGC |
| Dmagna_dsx1-b | MatScan | Dr | 359 | 365 | 0.95 | + | # GCAATTA |
| Dmagna_dsx1-b | MatScan | B-H1 | 360 | 366 | 0.89 | - | # GTAATTG |
| Dmagna_dsx1-b | MatScan | B-H2 | 360 | 366 | 0.91 | - | # GTAATTG |
| Dmagna_dsx1-b | MatScan | bsh | 360 | 366 | 0.91 | - | # GTAATTG |
| Dmagna_dsx1-b | MatScan | CG11085 | 360 | 366 | 0.88 | - | # GTAATTG |
| Dmagna_dsx1-b | MatScan | CG13424 | 360 | 366 | 0.92 | - | # GTAATTG |
| Dmagna_dsx1-b | MatScan | CG15696 | 360 | 366 | 0.88 | - | # GTAATTG |
| Dmagna_dsx1-b | MatScan | CG32532 | 360 | 366 | 0.9 | - | # GTAATTG |
| Dmagna_dsx1-b | MatScan | CG34031 | 360 | 366 | 0.86 | - | # GTAATTG |
| Dmagna_dsx1-b | MatScan | CG4328 | 360 | 366 | 0.91 | - | # GTAATTG |
| Dmagna_dsx1-b | MatScan | CG9876 | 360 | 366 | 0.88 | - | # GTAATTG |
| Dmagna_dsx1-b | MatScan | en | 360 | 366 | 0.91 | - | # GTAATTG |
| Dmagna_dsx1-b | MatScan | exex | 360 | 366 | 0.88 | - | # GTAATTG |
| Dmagna_dsx1-b | MatScan | exex | 360 | 366 | 0.88 | - | # GTAATTG |
| Dmagna_dsx1-b | MatScan | hbn | 360 | 366 | 0.89 | - | # GTAATTG |
| Dmagna_dsx1-b | MatScan | Hmx | 360 | 366 | 0.88 | - | # GTAATTG |
| Dmagna_dsx1-b | MatScan | lbl | 360 | 365 | 0.9 | - | # TAATTG |
| Dmagna_dsx1-b | MatScan | NK7.1 | 360 | 366 | 0.88 | - | # GTAATTG |
| Dmagna_dsx1-b | MatScan | OdsH | 360 | 366 | 0.91 | - | # GTAATTG |
| Dmagna_dsx1-b | MatScan | OdsH | 360 | 366 | 0.91 | - | # GTAATTG |
| Dmagna_dsx1-b | MatScan | PHDP | 360 | 366 | 0.89 | - | # GTAATTG |
| Dmagna_dsx1-b | MatScan | Pph13 | 360 | 366 | 0.9 | - | # GTAATTG |
| Dmagna_dsx1-b | MatScan | Rx | 360 | 366 | 0.88 | - | # GTAATTG |
| Dmagna_dsx1-b | MatScan | slou | 360 | 366 | 0.9 | - | # GTAATTG |
| Dmagna_dsx1-b | MatScan | tup | 360 | 366 | 0.91 | - | # GTAATTG |
| Dmagna_dsx1-b | MatScan | unc-4 | 360 | 366 | 0.92 | - | # GTAATTG |
| Dmagna_dsx1-b | MatScan | unpg | 360 | 366 | 0.9 | - | # GTAATTG |
| Dmagna_dsx1-b | MatScan | CG11617 | 393 | 399 | 0.89 | + | # TTCACAT |
| Dmagna_dsx1-b | MatScan | ovo | 420 | 428 | 0.86 | + | # AGGAACTGC |
| Dmagna_dsx1-b | MatScan | prd | 420 | 428 | 0.86 | + | # AGGAACTGC |
| Dmagna_dsx1-b | MatScan | Dfd | 425 | 440 | 0.86 | - | # CAAAGAATTATCGCAG |
| Dmagna_dsx1-b | MatScan | mtTFA | 426 | 436 | 0.88 | - | # GAATTATCGCA |
| Dmagna_dsx1-b | MatScan | Optix | 428 | 432 | 0.87 | + | # CGATA |
| Dmagna_dsx1-b | MatScan | Oct | 429 | 436 | 0.85 | + | # GATAATTC |
| Dmagna_dsx1-b | MatScan | CG4328 | 430 | 436 | 0.87 | + | # ATAATTC |
| Dmagna_dsx1-b | MatScan | PHDP | 430 | 436 | 0.9 | + | # ATAATTC |
| Dmagna_dsx1-b | MatScan | Pph13 | 430 | 436 | 0.88 | + | # ATAATTC |
| Dmagna_dsx1-b | MatScan | Dll | 431 | 437 | 0.88 | + | # TAATTCT |
| Dmagna_dsx1-b | MatScan | lbl | 431 | 436 | 0.85 | + | # TAATTC |
| Dmagna_dsx1-b | MatScan | pan | 436 | 443 | 0.95 | + | # CTTTGATT |
| Dmagna_dsx1-b | MatScan | onecut | 438 | 444 | 1 | + | # TTGATTT |
| Dmagna_dsx1-b | MatScan | hth | 445 | 450 | 0.88 | - | # TGACGG |
| Dmagna_dsx1-b | MatScan | Deaf1 | 452 | 457 | 0.87 | + | # TTCGAC |
| Dmagna_dsx1-b | MatScan | ara | 460 | 464 | 0.89 | + | # CAACA |
| Dmagna_dsx1-b | MatScan | caup | 460 | 464 | 0.87 | + | # CAACA |
| Dmagna_dsx1-b | MatScan | mirr | 460 | 464 | 0.89 | + | # CAACA |
| Dmagna_dsx1-b | MatScan | cad | 463 | 469 | 0.87 | - | # GTTATTG |
| Dmagna_dsx1-b | MatScan | CG4328 | 463 | 469 | 0.93 | - | # GTTATTG |
| Dmagna_dsx1-b | MatScan | lbe | 466 | 471 | 0.95 | + | # TAACCA |
| Dmagna_dsx1-b | MatScan | Six4 | 493 | 498 | 0.92 | - | # TGAAAC |
| Dmagna_dsx1-b | MatScan | exd | 494 | 501 | 0.88 | - | # GTTTGAAA |
| Dmagna_dsx1-b | MatScan | pan | 494 | 501 | 0.85 | - | # GTTTGAAA |
| Dmagna_dsx1-b | MatScan | kni | 498 | 509 | 0.86 | + | # AAACTATAGCAG |
| Dmagna_dsx1-b | MatScan | onecut | 533 | 539 | 1 | - | # TTGATTT |
| Dmagna_dsx1-b | MatScan | pan | 534 | 541 | 0.93 | - | # TTTTGATT |
| Dmagna_dsx1-b | MatScan | Croc | 539 | 554 | 0.91 | + | # AAAAATAAATACTAGA |
| Dmagna_dsx1-b | MatScan | Abd-B | 541 | 547 | 0.88 | - | # TTTATTT |
| Dmagna_dsx1-b | MatScan | cad | 541 | 547 | 0.91 | - | # TTTATTT |
| Dmagna_dsx1-b | MatScan | CG42234 | 541 | 547 | 0.85 | - | # TTTATTT |
| Dmagna_dsx1-b | MatScan | CG4328 | 541 | 547 | 0.9 | - | # TTTATTT |
| Dmagna_dsx1-b | MatScan | lbe | 544 | 549 | 0.91 | + | # TAAATA |
| Dmagna_dsx1-b | MatScan | B-H1 | 569 | 575 | 0.86 | - | # TTAAAAG |
| Dmagna_dsx1-b | MatScan | B-H1 | 572 | 578 | 0.86 | + | # TTAAAAG |
| Dmagna_dsx1-b | MatScan | ara | 593 | 597 | 0.89 | + | # CAACA |
| Dmagna_dsx1-b | MatScan | caup | 593 | 597 | 0.87 | + | # CAACA |
| Dmagna_dsx1-b | MatScan | mirr | 593 | 597 | 0.89 | + | # CAACA |
| Dmagna_dsx1-b | MatScan | dl | 605 | 615 | 0.86 | - | # TTAGAAAAGCA |
| Dmagna_dsx1-b | MatScan | run::Bgb | 614 | 622 | 0.86 | + | # AAAACGCAA |
| Dmagna_dsx1-b | MatScan | tll | 627 | 636 | 0.86 | + | # AAAAATCAAC |
| Dmagna_dsx1-b | MatScan | onecut | 629 | 635 | 1 | - | # TTGATTT |
| Dmagna_dsx1-b | MatScan | br_Z2 | 634 | 641 | 0.86 | - | # TCCTAGTT |
| Dmagna_dsx1-b | MatScan | ara | 645 | 649 | 0.93 | - | # GAACA |
| Dmagna_dsx1-b | MatScan | caup | 645 | 649 | 0.88 | - | # GAACA |
| Dmagna_dsx1-b | MatScan | mirr | 645 | 649 | 0.89 | - | # GAACA |
| Dmagna_dsx1-b | MatScan | ct | 646 | 651 | 1 | - | # TTGAAC |
| Dmagna_dsx1-b | MatScan | pan | 653 | 660 | 0.91 | - | # CTTTGGTC |
| Dmagna_dsx1-b | MatScan | Abd-B | 664 | 670 | 0.88 | - | # TTTATTT |
| Dmagna_dsx1-b | MatScan | br_Z2 | 664 | 671 | 0.86 | - | # TTTTATTT |
| Dmagna_dsx1-b | MatScan | BR-C | 664 | 681 | 0.88 | + | # AAATAAAAGGCAAATTTA |
| Dmagna_dsx1-b | MatScan | cad | 664 | 670 | 0.91 | - | # TTTATTT |
| Dmagna_dsx1-b | MatScan | CG42234 | 664 | 670 | 0.85 | - | # TTTATTT |
| Dmagna_dsx1-b | MatScan | CG4328 | 664 | 670 | 0.9 | - | # TTTATTT |
| Dmagna_dsx1-b | MatScan | br_Z1 | 666 | 679 | 0.86 | + | # ATAAAAGGCAAATT |
| Dmagna_dsx1-b | MatScan | tll | 668 | 677 | 0.89 | + | # AAAAGGCAAA |
| Dmagna_dsx1-b | MatScan | C15 | 676 | 682 | 0.85 | - | # TTAAATT |
| Dmagna_dsx1-b | MatScan | Dfd | 677 | 692 | 0.92 | + | # ATTTAAATTACTATTA |
| Dmagna_dsx1-b | MatScan | C15 | 679 | 685 | 0.85 | + | # TTAAATT |
| Dmagna_dsx1-b | MatScan | CG7056 | 679 | 686 | 0.91 | + | # TTAAATTA |
| Dmagna_dsx1-b | MatScan | al | 680 | 686 | 0.87 | - | # TAATTTA |
| Dmagna_dsx1-b | MatScan | CG11294 | 680 | 686 | 0.87 | + | # TAAATTA |
| Dmagna_dsx1-b | MatScan | CG15696 | 680 | 686 | 0.86 | + | # TAAATTA |
| Dmagna_dsx1-b | MatScan | CG32105 | 680 | 686 | 0.87 | + | # TAAATTA |
| Dmagna_dsx1-b | MatScan | CG4328 | 680 | 686 | 0.87 | + | # TAAATTA |
| Dmagna_dsx1-b | MatScan | Dll | 680 | 686 | 0.87 | - | # TAATTTA |
| Dmagna_dsx1-b | MatScan | dri | 680 | 689 | 0.88 | + | # TAAATTACTA |
| Dmagna_dsx1-b | MatScan | ems | 680 | 686 | 0.86 | + | # TAAATTA |
| Dmagna_dsx1-b | MatScan | ftz | 680 | 686 | 0.85 | + | # TAAATTA |
| Dmagna_dsx1-b | MatScan | Lim1 | 680 | 686 | 0.85 | + | # TAAATTA |
| Dmagna_dsx1-b | MatScan | Lim3 | 680 | 686 | 0.89 | + | # TAAATTA |
| Dmagna_dsx1-b | MatScan | repo | 680 | 686 | 0.85 | + | # TAAATTA |
| Dmagna_dsx1-b | MatScan | exex | 681 | 687 | 0.86 | - | # GTAATTT |
| Dmagna_dsx1-b | MatScan | exex | 681 | 687 | 0.86 | - | # GTAATTT |
| Dmagna_dsx1-b | MatScan | lbl | 681 | 686 | 0.85 | - | # TAATTT |
| Dmagna_dsx1-b | MatScan | OdsH | 681 | 687 | 0.86 | - | # GTAATTT |
| Dmagna_dsx1-b | MatScan | OdsH | 681 | 687 | 0.86 | - | # GTAATTT |
| Dmagna_dsx1-b | MatScan | PHDP | 681 | 687 | 0.89 | - | # GTAATTT |
| Dmagna_dsx1-b | MatScan | Pph13 | 681 | 687 | 0.86 | - | # GTAATTT |
| Dmagna_dsx1-b | MatScan | br_Z2 | 685 | 692 | 0.89 | + | # TACTATTA |
| Dmagna_dsx1-b | MatScan | CG4328 | 686 | 692 | 0.85 | + | # ACTATTA |
| Dmagna_dsx1-b | MatScan | B-H1 | 687 | 693 | 0.9 | - | # TTAATAG |
| Dmagna_dsx1-b | MatScan | B-H2 | 687 | 693 | 0.9 | - | # TTAATAG |
| Dmagna_dsx1-b | MatScan | bsh | 687 | 693 | 0.92 | - | # TTAATAG |
| Dmagna_dsx1-b | MatScan | C15 | 687 | 693 | 0.89 | - | # TTAATAG |
| Dmagna_dsx1-b | MatScan | CG11085 | 687 | 693 | 0.93 | - | # TTAATAG |
| Dmagna_dsx1-b | MatScan | CG13424 | 687 | 693 | 0.91 | - | # TTAATAG |
| Dmagna_dsx1-b | MatScan | CG34031 | 687 | 693 | 0.94 | - | # TTAATAG |
| Dmagna_dsx1-b | MatScan | H2.0 | 687 | 693 | 0.85 | - | # TTAATAG |
| Dmagna_dsx1-b | MatScan | Hmx | 687 | 693 | 0.87 | - | # TTAATAG |
| Dmagna_dsx1-b | MatScan | NK7.1 | 687 | 693 | 0.92 | - | # TTAATAG |
| Dmagna_dsx1-b | MatScan | slou | 687 | 693 | 0.9 | - | # TTAATAG |
| Dmagna_dsx1-b | MatScan | tup | 687 | 693 | 0.92 | - | # TTAATAG |
| Dmagna_dsx1-b | MatScan | unc-4 | 687 | 693 | 0.85 | - | # TTAATAG |
| Dmagna_dsx1-b | MatScan | vvl | 688 | 693 | 0.88 | + | # TATTAA |
| Dmagna_dsx1-b | MatScan | bap | 690 | 696 | 0.86 | + | # TTAAGCG |
| Dmagna_dsx1-b | MatScan | bap | 701 | 707 | 0.86 | - | # TTAAGAG |
| Dmagna_dsx1-b | MatScan | B-H1 | 704 | 710 | 0.96 | + | # TTAAATG |
| Dmagna_dsx1-b | MatScan | B-H2 | 704 | 710 | 0.91 | + | # TTAAATG |
| Dmagna_dsx1-b | MatScan | C15 | 704 | 710 | 0.94 | + | # TTAAATG |
| Dmagna_dsx1-b | MatScan | CG11085 | 704 | 710 | 0.86 | + | # TTAAATG |
| Dmagna_dsx1-b | MatScan | CG34031 | 704 | 710 | 0.87 | + | # TTAAATG |
| Dmagna_dsx1-b | MatScan | Hmx | 704 | 710 | 0.86 | + | # TTAAATG |
| Dmagna_dsx1-b | MatScan | NK7.1 | 704 | 710 | 0.89 | + | # TTAAATG |
| Dmagna_dsx1-b | MatScan | lbe | 709 | 714 | 0.95 | - | # TAACCA |
| Dmagna_dsx1-b | MatScan | mtTFA | 709 | 719 | 0.92 | + | # TGGTTATCAGT |
| Dmagna_dsx1-b | MatScan | so | 712 | 717 | 0.93 | - | # TGATAA |
| Dmagna_dsx1-b | MatScan | Optix | 713 | 717 | 1 | - | # TGATA |
| Dmagna_dsx1-b | MatScan | Optix | 719 | 723 | 0.87 | - | # CGATA |
| Dmagna_dsx1-b | MatScan | ct | 724 | 729 | 0.86 | + | # ATGAAC |
| Dmagna_dsx1-b | MatScan | ara | 726 | 730 | 0.93 | + | # GAACA |
| Dmagna_dsx1-b | MatScan | caup | 726 | 730 | 0.88 | + | # GAACA |
| Dmagna_dsx1-b | MatScan | mirr | 726 | 730 | 0.89 | + | # GAACA |
| Dmagna_dsx1-b | MatScan | vvl | 734 | 739 | 1 | + | # TATGCA |
| Dmagna_dsx1-b | MatScan | Deaf1 | 751 | 756 | 0.98 | + | # TTCGTC |
| Dmagna_dsx1-b | MatScan | Dfd | 755 | 770 | 0.87 | - | # TCGTAAATTAACAAGA |
| Dmagna_dsx1-b | MatScan | CG11617 | 757 | 763 | 0.99 | - | # TTAACAA |
| Dmagna_dsx1-b | MatScan | lbe | 757 | 762 | 0.94 | - | # TAACAA |
| Dmagna_dsx1-b | MatScan | ara | 758 | 762 | 1 | - | # TAACA |
| Dmagna_dsx1-b | MatScan | caup | 758 | 762 | 1 | - | # TAACA |
| Dmagna_dsx1-b | MatScan | dri | 758 | 767 | 0.91 | - | # TAAATTAACA |
| Dmagna_dsx1-b | MatScan | mirr | 758 | 762 | 0.99 | - | # TAACA |
| Dmagna_dsx1-b | MatScan | Oct | 759 | 766 | 0.91 | + | # GTTAATTT |
| Dmagna_dsx1-b | MatScan | abd-A | 760 | 766 | 0.86 | + | # TTAATTT |
| Dmagna_dsx1-b | MatScan | al | 760 | 766 | 0.85 | - | # AAATTAA |
| Dmagna_dsx1-b | MatScan | Awh | 760 | 766 | 0.87 | + | # TTAATTT |
| Dmagna_dsx1-b | MatScan | bsh | 760 | 766 | 0.9 | + | # TTAATTT |
| Dmagna_dsx1-b | MatScan | C15 | 760 | 766 | 0.9 | + | # TTAATTT |
| Dmagna_dsx1-b | MatScan | CG13424 | 760 | 766 | 0.89 | + | # TTAATTT |
| Dmagna_dsx1-b | MatScan | CG15696 | 760 | 766 | 0.91 | + | # TTAATTT |
| Dmagna_dsx1-b | MatScan | CG32105 | 760 | 766 | 0.87 | + | # TTAATTT |
| Dmagna_dsx1-b | MatScan | CG32532 | 760 | 766 | 0.92 | + | # TTAATTT |
| Dmagna_dsx1-b | MatScan | CG34031 | 760 | 766 | 0.86 | + | # TTAATTT |
| Dmagna_dsx1-b | MatScan | CG4328 | 760 | 766 | 0.87 | + | # TTAATTT |
| Dmagna_dsx1-b | MatScan | CG9876 | 760 | 766 | 0.86 | + | # TTAATTT |
| Dmagna_dsx1-b | MatScan | E5 | 760 | 766 | 0.85 | + | # TTAATTT |
| Dmagna_dsx1-b | MatScan | ems | 760 | 766 | 0.86 | + | # TTAATTT |
| Dmagna_dsx1-b | MatScan | en | 760 | 766 | 0.9 | + | # TTAATTT |
| Dmagna_dsx1-b | MatScan | ftz | 760 | 766 | 0.87 | + | # TTAATTT |
| Dmagna_dsx1-b | MatScan | H2.0 | 760 | 766 | 0.86 | + | # TTAATTT |
| Dmagna_dsx1-b | MatScan | hbn | 760 | 766 | 0.92 | + | # TTAATTT |
| Dmagna_dsx1-b | MatScan | HGTX | 760 | 766 | 0.87 | + | # TTAATTT |
| Dmagna_dsx1-b | MatScan | Hmx | 760 | 766 | 0.88 | + | # TTAATTT |
| Dmagna_dsx1-b | MatScan | Lim1 | 760 | 766 | 0.86 | + | # TTAATTT |
| Dmagna_dsx1-b | MatScan | Lim3 | 760 | 766 | 0.85 | + | # TTAATTT |
| Dmagna_dsx1-b | MatScan | NK7.1 | 760 | 766 | 0.89 | + | # TTAATTT |
| Dmagna_dsx1-b | MatScan | OdsH | 760 | 766 | 0.88 | + | # TTAATTT |
| Dmagna_dsx1-b | MatScan | OdsH | 760 | 766 | 0.88 | + | # TTAATTT |
| Dmagna_dsx1-b | MatScan | otp | 760 | 766 | 0.89 | + | # TTAATTT |
| Dmagna_dsx1-b | MatScan | PHDP | 760 | 766 | 0.96 | + | # TTAATTT |
| Dmagna_dsx1-b | MatScan | Pph13 | 760 | 766 | 0.87 | + | # TTAATTT |
| Dmagna_dsx1-b | MatScan | repo | 760 | 766 | 0.88 | + | # TTAATTT |
| Dmagna_dsx1-b | MatScan | Rx | 760 | 766 | 0.88 | + | # TTAATTT |
| Dmagna_dsx1-b | MatScan | slou | 760 | 766 | 0.91 | + | # TTAATTT |
| Dmagna_dsx1-b | MatScan | tup | 760 | 766 | 0.87 | + | # TTAATTT |
| Dmagna_dsx1-b | MatScan | unc-4 | 760 | 766 | 0.89 | + | # TTAATTT |
| Dmagna_dsx1-b | MatScan | unpg | 760 | 766 | 0.89 | + | # TTAATTT |
| Dmagna_dsx1-b | MatScan | Vsx1 | 760 | 766 | 0.89 | + | # TTAATTT |
| Dmagna_dsx1-b | MatScan | zen2 | 760 | 766 | 0.85 | + | # TTAATTT |
| Dmagna_dsx1-b | MatScan | al | 761 | 767 | 0.87 | + | # TAATTTA |
| Dmagna_dsx1-b | MatScan | CG11294 | 761 | 767 | 0.87 | - | # TAAATTA |
| Dmagna_dsx1-b | MatScan | CG15696 | 761 | 767 | 0.86 | - | # TAAATTA |
| Dmagna_dsx1-b | MatScan | CG32105 | 761 | 767 | 0.87 | - | # TAAATTA |
| Dmagna_dsx1-b | MatScan | CG4328 | 761 | 767 | 0.87 | - | # TAAATTA |
| Dmagna_dsx1-b | MatScan | Dll | 761 | 767 | 0.87 | + | # TAATTTA |
| Dmagna_dsx1-b | MatScan | ems | 761 | 767 | 0.86 | - | # TAAATTA |
| Dmagna_dsx1-b | MatScan | ftz | 761 | 767 | 0.85 | - | # TAAATTA |
| Dmagna_dsx1-b | MatScan | lbl | 761 | 766 | 0.85 | + | # TAATTT |
| Dmagna_dsx1-b | MatScan | Lim1 | 761 | 767 | 0.85 | - | # TAAATTA |
| Dmagna_dsx1-b | MatScan | Lim3 | 761 | 767 | 0.89 | - | # TAAATTA |
| Dmagna_dsx1-b | MatScan | repo | 761 | 767 | 0.85 | - | # TAAATTA |
| Dmagna_dsx1-b | MatScan | Abd-B | 764 | 770 | 0.88 | + | # TTTACGA |
| Dmagna_dsx1-b | MatScan | ara | 777 | 781 | 0.89 | - | # CAACA |
| Dmagna_dsx1-b | MatScan | caup | 777 | 781 | 0.87 | - | # CAACA |
| Dmagna_dsx1-b | MatScan | mirr | 777 | 781 | 0.89 | - | # CAACA |
| Dmagna_dsx1-b | MatScan | odd | 779 | 789 | 0.87 | - | # CACACTAGCAA |
| Dmagna_dsx1-b | MatScan | CG11617 | 785 | 791 | 0.88 | - | # TTCACAC |
| Dmagna_dsx1-b | MatScan | Deaf1 | 808 | 813 | 0.91 | - | # CTCGTG |
| Dmagna_dsx1-b | MatScan | Dfd | 818 | 833 | 0.92 | + | # TTGCTGATTAATTCAA |
| Dmagna_dsx1-b | MatScan | Dfd | 820 | 835 | 0.91 | - | # TTTTGAATTAATCAGC |
| Dmagna_dsx1-b | MatScan | AP-1 | 821 | 829 | 0.85 | - | # ATTAATCAG |
| Dmagna_dsx1-b | MatScan | Awh | 821 | 827 | 0.87 | + | # CTGATTA |
| Dmagna_dsx1-b | MatScan | Bcd | 821 | 828 | 0.87 | + | # CTGATTAA |
| Dmagna_dsx1-b | MatScan | dri | 821 | 830 | 0.92 | + | # CTGATTAATT |
| Dmagna_dsx1-b | MatScan | Lim3 | 821 | 827 | 0.87 | + | # CTGATTA |
| Dmagna_dsx1-b | MatScan | onecut | 821 | 827 | 0.88 | + | # CTGATTA |
| Dmagna_dsx1-b | MatScan | abd-A | 822 | 828 | 0.89 | - | # TTAATCA |
| Dmagna_dsx1-b | MatScan | al | 822 | 828 | 0.85 | + | # TGATTAA |
| Dmagna_dsx1-b | MatScan | Antp | 822 | 828 | 0.91 | - | # TTAATCA |
| Dmagna_dsx1-b | MatScan | ap | 822 | 828 | 0.86 | - | # TTAATCA |
| Dmagna_dsx1-b | MatScan | Awh | 822 | 828 | 0.87 | - | # TTAATCA |
| Dmagna_dsx1-b | MatScan | bsh | 822 | 828 | 0.93 | - | # TTAATCA |
| Dmagna_dsx1-b | MatScan | btn | 822 | 828 | 0.89 | - | # TTAATCA |
| Dmagna_dsx1-b | MatScan | C15 | 822 | 828 | 0.93 | - | # TTAATCA |
| Dmagna_dsx1-b | MatScan | CG18599 | 822 | 828 | 0.9 | - | # TTAATCA |
| Dmagna_dsx1-b | MatScan | CG42234 | 822 | 828 | 0.91 | - | # TTAATCA |
| Dmagna_dsx1-b | MatScan | Dfd | 822 | 828 | 0.87 | - | # TTAATCA |
| Dmagna_dsx1-b | MatScan | E5 | 822 | 828 | 0.91 | - | # TTAATCA |
| Dmagna_dsx1-b | MatScan | ems | 822 | 828 | 0.92 | - | # TTAATCA |
| Dmagna_dsx1-b | MatScan | eve | 822 | 828 | 0.92 | - | # TTAATCA |
| Dmagna_dsx1-b | MatScan | ftz | 822 | 828 | 0.92 | - | # TTAATCA |
| Dmagna_dsx1-b | MatScan | Gsc | 822 | 827 | 0.89 | - | # TAATCA |
| Dmagna_dsx1-b | MatScan | H2.0 | 822 | 828 | 0.91 | - | # TTAATCA |
| Dmagna_dsx1-b | MatScan | HGTX | 822 | 828 | 0.89 | - | # TTAATCA |
| Dmagna_dsx1-b | MatScan | ind | 822 | 828 | 0.87 | - | # TTAATCA |
| Dmagna_dsx1-b | MatScan | lab | 822 | 828 | 0.9 | - | # TTAATCA |
| Dmagna_dsx1-b | MatScan | lbe | 822 | 827 | 0.94 | - | # TAATCA |
| Dmagna_dsx1-b | MatScan | lbl | 822 | 827 | 0.92 | - | # TAATCA |
| Dmagna_dsx1-b | MatScan | Lim1 | 822 | 828 | 0.85 | - | # TTAATCA |
| Dmagna_dsx1-b | MatScan | Lim3 | 822 | 828 | 0.89 | - | # TTAATCA |
| Dmagna_dsx1-b | MatScan | Oct | 822 | 829 | 0.88 | - | # ATTAATCA |
| Dmagna_dsx1-b | MatScan | otp | 822 | 828 | 0.88 | - | # TTAATCA |
| Dmagna_dsx1-b | MatScan | pb | 822 | 828 | 0.91 | - | # TTAATCA |
| Dmagna_dsx1-b | MatScan | Ptx1 | 822 | 828 | 0.86 | - | # TTAATCA |
| Dmagna_dsx1-b | MatScan | Scr | 822 | 828 | 0.88 | - | # TTAATCA |
| Dmagna_dsx1-b | MatScan | slou | 822 | 828 | 0.89 | - | # TTAATCA |
| Dmagna_dsx1-b | MatScan | Vsx1 | 822 | 828 | 0.87 | - | # TTAATCA |
| Dmagna_dsx1-b | MatScan | vvl | 822 | 827 | 0.88 | - | # TAATCA |
| Dmagna_dsx1-b | MatScan | zen2 | 822 | 828 | 0.89 | - | # TTAATCA |
| Dmagna_dsx1-b | MatScan | dri | 823 | 832 | 0.96 | - | # TGAATTAATC |
| Dmagna_dsx1-b | MatScan | abd-A | 825 | 831 | 0.87 | + | # TTAATTC |
| Dmagna_dsx1-b | MatScan | al | 825 | 831 | 0.85 | - | # GAATTAA |
| Dmagna_dsx1-b | MatScan | Awh | 825 | 831 | 0.86 | + | # TTAATTC |
| Dmagna_dsx1-b | MatScan | bsh | 825 | 831 | 0.9 | + | # TTAATTC |
| Dmagna_dsx1-b | MatScan | C15 | 825 | 831 | 0.9 | + | # TTAATTC |
| Dmagna_dsx1-b | MatScan | CG13424 | 825 | 831 | 0.89 | + | # TTAATTC |
| Dmagna_dsx1-b | MatScan | CG15696 | 825 | 831 | 0.91 | + | # TTAATTC |
| Dmagna_dsx1-b | MatScan | CG32105 | 825 | 831 | 0.87 | + | # TTAATTC |
| Dmagna_dsx1-b | MatScan | CG32532 | 825 | 831 | 0.91 | + | # TTAATTC |
| Dmagna_dsx1-b | MatScan | CG34031 | 825 | 831 | 0.86 | + | # TTAATTC |
| Dmagna_dsx1-b | MatScan | CG4328 | 825 | 831 | 0.87 | + | # TTAATTC |
| Dmagna_dsx1-b | MatScan | CG9876 | 825 | 831 | 0.86 | + | # TTAATTC |
| Dmagna_dsx1-b | MatScan | E5 | 825 | 831 | 0.85 | + | # TTAATTC |
| Dmagna_dsx1-b | MatScan | ems | 825 | 831 | 0.86 | + | # TTAATTC |
| Dmagna_dsx1-b | MatScan | en | 825 | 831 | 0.9 | + | # TTAATTC |
| Dmagna_dsx1-b | MatScan | ftz | 825 | 831 | 0.87 | + | # TTAATTC |
| Dmagna_dsx1-b | MatScan | H2.0 | 825 | 831 | 0.85 | + | # TTAATTC |
| Dmagna_dsx1-b | MatScan | hbn | 825 | 831 | 0.91 | + | # TTAATTC |
| Dmagna_dsx1-b | MatScan | HGTX | 825 | 831 | 0.87 | + | # TTAATTC |
| Dmagna_dsx1-b | MatScan | Hmx | 825 | 831 | 0.88 | + | # TTAATTC |
| Dmagna_dsx1-b | MatScan | Lim1 | 825 | 831 | 0.86 | + | # TTAATTC |
| Dmagna_dsx1-b | MatScan | Lim3 | 825 | 831 | 0.85 | + | # TTAATTC |
| Dmagna_dsx1-b | MatScan | NK7.1 | 825 | 831 | 0.89 | + | # TTAATTC |
| Dmagna_dsx1-b | MatScan | OdsH | 825 | 831 | 0.87 | + | # TTAATTC |
| Dmagna_dsx1-b | MatScan | OdsH | 825 | 831 | 0.87 | + | # TTAATTC |
| Dmagna_dsx1-b | MatScan | otp | 825 | 831 | 0.87 | + | # TTAATTC |
| Dmagna_dsx1-b | MatScan | PHDP | 825 | 831 | 0.93 | + | # TTAATTC |
| Dmagna_dsx1-b | MatScan | Pph13 | 825 | 831 | 0.87 | + | # TTAATTC |
| Dmagna_dsx1-b | MatScan | repo | 825 | 831 | 0.88 | + | # TTAATTC |
| Dmagna_dsx1-b | MatScan | Rx | 825 | 831 | 0.88 | + | # TTAATTC |
| Dmagna_dsx1-b | MatScan | slou | 825 | 831 | 0.91 | + | # TTAATTC |
| Dmagna_dsx1-b | MatScan | tup | 825 | 831 | 0.86 | + | # TTAATTC |
| Dmagna_dsx1-b | MatScan | unc-4 | 825 | 831 | 0.9 | + | # TTAATTC |
| Dmagna_dsx1-b | MatScan | unpg | 825 | 831 | 0.89 | + | # TTAATTC |
| Dmagna_dsx1-b | MatScan | Vsx1 | 825 | 831 | 0.89 | + | # TTAATTC |
| Dmagna_dsx1-b | MatScan | zen2 | 825 | 831 | 0.85 | + | # TTAATTC |
| Dmagna_dsx1-b | MatScan | al | 826 | 832 | 0.87 | + | # TAATTCA |
| Dmagna_dsx1-b | MatScan | CG11294 | 826 | 832 | 0.87 | - | # TGAATTA |
| Dmagna_dsx1-b | MatScan | CG32105 | 826 | 832 | 0.85 | - | # TGAATTA |
| Dmagna_dsx1-b | MatScan | CG7056 | 826 | 833 | 0.96 | - | # TTGAATTA |
| Dmagna_dsx1-b | MatScan | lbl | 826 | 831 | 0.85 | + | # TAATTC |
| Dmagna_dsx1-b | MatScan | Lim1 | 826 | 832 | 0.85 | - | # TGAATTA |
| Dmagna_dsx1-b | MatScan | Lim3 | 826 | 832 | 0.85 | - | # TGAATTA |
| Dmagna_dsx1-b | MatScan | pan | 828 | 835 | 0.87 | - | # TTTTGAAT |
| Dmagna_dsx1-b | MatScan | Kr | 841 | 851 | 0.86 | - | # CAGAAGGGTTG |
| Dmagna_dsx1-b | MatScan | gt | 854 | 863 | 0.85 | - | # ATTACGCAAA |
| Dmagna_dsx1-b | MatScan | gt | 854 | 863 | 0.85 | + | # TTTGCGTAAT |
| Dmagna_dsx1-b | MatScan | slbo | 856 | 863 | 0.85 | - | # ATTACGCA |
| Dmagna_dsx1-b | MatScan | vvl | 856 | 861 | 0.9 | - | # TACGCA |
| Dmagna_dsx1-b | MatScan | exex | 859 | 865 | 0.86 | + | # GTAATTT |
| Dmagna_dsx1-b | MatScan | exex | 859 | 865 | 0.86 | + | # GTAATTT |
| Dmagna_dsx1-b | MatScan | OdsH | 859 | 865 | 0.86 | + | # GTAATTT |
| Dmagna_dsx1-b | MatScan | OdsH | 859 | 865 | 0.86 | + | # GTAATTT |
| Dmagna_dsx1-b | MatScan | PHDP | 859 | 865 | 0.89 | + | # GTAATTT |
| Dmagna_dsx1-b | MatScan | Pph13 | 859 | 865 | 0.86 | + | # GTAATTT |
| Dmagna_dsx1-b | MatScan | CG4328 | 860 | 866 | 0.87 | - | # AAAATTA |
| Dmagna_dsx1-b | MatScan | Dll | 860 | 866 | 0.91 | + | # TAATTTT |
| Dmagna_dsx1-b | MatScan | lbl | 860 | 865 | 0.85 | + | # TAATTT |
| Dmagna_dsx1-b | MatScan | pan | 863 | 870 | 0.93 | + | # TTTTGATT |
| Dmagna_dsx1-b | MatScan | onecut | 865 | 871 | 1 | + | # TTGATTT |
| Dmagna_dsx1-b | MatScan | CG7056 | 870 | 877 | 0.87 | + | # TTTAAATA |
| Dmagna_dsx1-b | MatScan | Dfd | 870 | 885 | 0.85 | - | # GTTATCATTATTTAAA |
| Dmagna_dsx1-b | MatScan | abd-A | 871 | 877 | 0.85 | + | # TTAAATA |
| Dmagna_dsx1-b | MatScan | al | 871 | 877 | 0.85 | - | # TATTTAA |
| Dmagna_dsx1-b | MatScan | C15 | 871 | 877 | 0.95 | + | # TTAAATA |
| Dmagna_dsx1-b | MatScan | lab | 871 | 877 | 0.86 | + | # TTAAATA |
| Dmagna_dsx1-b | MatScan | Lim1 | 871 | 877 | 0.85 | + | # TTAAATA |
| Dmagna_dsx1-b | MatScan | al | 872 | 878 | 0.85 | + | # TAAATAA |
| Dmagna_dsx1-b | MatScan | lbe | 872 | 877 | 0.91 | + | # TAAATA |
| Dmagna_dsx1-b | MatScan | Lim1 | 872 | 878 | 0.85 | - | # TTATTTA |
| Dmagna_dsx1-b | MatScan | CG4328 | 873 | 879 | 0.89 | - | # ATTATTT |
| Dmagna_dsx1-b | MatScan | Oct | 874 | 881 | 0.88 | + | # AATAATGA |
| Dmagna_dsx1-b | MatScan | Antp | 875 | 881 | 0.87 | + | # ATAATGA |
| Dmagna_dsx1-b | MatScan | ap | 875 | 881 | 0.87 | + | # ATAATGA |
| Dmagna_dsx1-b | MatScan | bsh | 875 | 881 | 0.86 | + | # ATAATGA |
| Dmagna_dsx1-b | MatScan | btn | 875 | 881 | 0.94 | + | # ATAATGA |
| Dmagna_dsx1-b | MatScan | CG18599 | 875 | 881 | 0.91 | + | # ATAATGA |
| Dmagna_dsx1-b | MatScan | Dfd | 875 | 881 | 0.9 | + | # ATAATGA |
| Dmagna_dsx1-b | MatScan | E5 | 875 | 881 | 0.92 | + | # ATAATGA |
| Dmagna_dsx1-b | MatScan | ems | 875 | 881 | 0.94 | + | # ATAATGA |
| Dmagna_dsx1-b | MatScan | eve | 875 | 881 | 0.94 | + | # ATAATGA |
| Dmagna_dsx1-b | MatScan | ftz | 875 | 881 | 0.87 | + | # ATAATGA |
| Dmagna_dsx1-b | MatScan | H2.0 | 875 | 881 | 0.88 | + | # ATAATGA |
| Dmagna_dsx1-b | MatScan | HGTX | 875 | 881 | 0.88 | + | # ATAATGA |
| Dmagna_dsx1-b | MatScan | ind | 875 | 881 | 0.87 | + | # ATAATGA |
| Dmagna_dsx1-b | MatScan | lab | 875 | 881 | 0.85 | + | # ATAATGA |
| Dmagna_dsx1-b | MatScan | Lim3 | 875 | 881 | 0.86 | + | # ATAATGA |
| Dmagna_dsx1-b | MatScan | pb | 875 | 881 | 0.94 | + | # ATAATGA |
| Dmagna_dsx1-b | MatScan | Scr | 875 | 881 | 0.92 | + | # ATAATGA |
| Dmagna_dsx1-b | MatScan | zen | 875 | 881 | 0.93 | + | # ATAATGA |
| Dmagna_dsx1-b | MatScan | zen2 | 875 | 881 | 0.91 | + | # ATAATGA |
| Dmagna_dsx1-b | MatScan | CG4328 | 876 | 882 | 0.88 | - | # ATCATTA |
| Dmagna_dsx1-b | MatScan | lbe | 876 | 881 | 0.92 | + | # TAATGA |
| Dmagna_dsx1-b | MatScan | lbl | 876 | 881 | 0.96 | + | # TAATGA |
| Dmagna_dsx1-b | MatScan | Optix | 879 | 883 | 1 | + | # TGATA |
| Dmagna_dsx1-b | MatScan | so | 879 | 884 | 0.93 | + | # TGATAA |
| Dmagna_dsx1-b | MatScan | ara | 882 | 886 | 1 | + | # TAACA |
| Dmagna_dsx1-b | MatScan | caup | 882 | 886 | 1 | + | # TAACA |
| Dmagna_dsx1-b | MatScan | mirr | 882 | 886 | 0.99 | + | # TAACA |
| Dmagna_dsx1-b | MatScan | ara | 895 | 899 | 0.91 | + | # TTACA |
| Dmagna_dsx1-b | MatScan | caup | 895 | 899 | 0.87 | + | # TTACA |
| Dmagna_dsx1-b | MatScan | mirr | 895 | 899 | 0.88 | + | # TTACA |
| Dmagna_dsx1-b | MatScan | ara | 902 | 906 | 0.89 | - | # CAACA |
| Dmagna_dsx1-b | MatScan | caup | 902 | 906 | 0.87 | - | # CAACA |
| Dmagna_dsx1-b | MatScan | mirr | 902 | 906 | 0.89 | - | # CAACA |
| Dmagna_dsx1-b | MatScan | ara | 905 | 909 | 0.89 | - | # CAACA |
| Dmagna_dsx1-b | MatScan | caup | 905 | 909 | 0.87 | - | # CAACA |
| Dmagna_dsx1-b | MatScan | mirr | 905 | 909 | 0.89 | - | # CAACA |
| Dmagna_dsx1-b | MatScan | Ovo | 905 | 919 | 0.85 | + | # TGTTGTAACAGAAAT |
| Dmagna_dsx1-b | MatScan | ara | 908 | 912 | 0.91 | - | # TTACA |
| Dmagna_dsx1-b | MatScan | caup | 908 | 912 | 0.87 | - | # TTACA |
| Dmagna_dsx1-b | MatScan | mirr | 908 | 912 | 0.88 | - | # TTACA |
| Dmagna_dsx1-b | MatScan | ovo | 908 | 916 | 0.94 | + | # TGTAACAGA |
| Dmagna_dsx1-b | MatScan | prd | 908 | 916 | 0.94 | + | # TGTAACAGA |
| Dmagna_dsx1-b | MatScan | ara | 910 | 914 | 1 | + | # TAACA |
| Dmagna_dsx1-b | MatScan | caup | 910 | 914 | 1 | + | # TAACA |
| Dmagna_dsx1-b | MatScan | mirr | 910 | 914 | 0.99 | + | # TAACA |
| Dmagna_dsx1-b | MatScan | sd | 915 | 926 | 0.91 | + | # GAAATTCGTCAA |
| Dmagna_dsx1-b | MatScan | Deaf1 | 919 | 924 | 0.98 | + | # TTCGTC |
| Dmagna_dsx1-b | MatScan | ara | 924 | 928 | 0.89 | + | # CAACA |
| Dmagna_dsx1-b | MatScan | caup | 924 | 928 | 0.87 | + | # CAACA |
| Dmagna_dsx1-b | MatScan | mirr | 924 | 928 | 0.89 | + | # CAACA |
| Dmagna_dsx1-b | MatScan | ara | 929 | 933 | 0.99 | + | # AAACA |
| Dmagna_dsx1-b | MatScan | caup | 929 | 933 | 0.9 | + | # AAACA |
| Dmagna_dsx1-b | MatScan | mirr | 929 | 933 | 1 | + | # AAACA |
| Dmagna_dsx1-b | MatScan | achi | 956 | 961 | 0.9 | - | # TGACAC |
| Dmagna_dsx1-b | MatScan | hth | 956 | 961 | 0.91 | - | # TGACAC |
| Dmagna_dsx1-b | MatScan | Six4 | 956 | 961 | 0.98 | - | # TGACAC |
| Dmagna_dsx1-b | MatScan | vis | 956 | 961 | 0.97 | - | # TGACAC |
| Dmagna_dsx1-b | MatScan | caup | 957 | 961 | 0.85 | - | # TGACA |
| Dmagna_dsx1-b | MatScan | bap | 964 | 970 | 0.86 | + | # GTAAGTG |
