# Supplementary material for: Molecular cloning of doublesex genes of four cladocera (water flea) species
Source: BMC Genomics. 2013 Apr 10;14:239. doi: 10.1186/1471-2164-14-239 (PMC3637828; doi:10.1186/1471-2164-14-239)
Supplement: Additional file 19 — DappuDsx1-β TF-map. [file 1471-2164-14-239-S19.doc]

Supplemental Material 19. *DappuDsx1-β* TF-map

| Column Descriptions | Promoter region ID - Species, dsx paralog number, and dsx transcript identifier  Name of program that generated results  Name of transcription factor identified  Start of transcription factor binding site (TFBS)  End of transcription factor binding site (TFBS)  Match score between known TFBS (from TFBS database) and identified Daphnia dsx promoter sequence motif  Strand on which TFBS was identified in sequence  Reading frame for CDS feature types (not used)  Sequence of transcription factor binding motif (from TFBS database) | | | | | | |
| --- | --- | --- | --- | --- | --- | --- | --- |
| Sequence ID |
| Source |
| Type (TF) |
| Start |
| End |
| Score |
| Strand |
| Phase |
| TF Binding Motif |
|  |  |  |  |  |  |  |  |
| **Sequence ID** | **Source** | **Type (TF)** | **Start** | **End** | **Score** | **Strand** | **TF Binding Motif** |
| Dpulex_dsx1-b | MatScan | slbo | 6 | 13 | 0.85 | - | # ATTCCAGA |
| Dpulex_dsx1-b | MatScan | exd | 46 | 53 | 0.99 | + | # GTTTGACA |
| Dpulex_dsx1-b | MatScan | CG11617 | 48 | 54 | 0.92 | + | # TTGACAA |
| Dpulex_dsx1-b | MatScan | achi | 49 | 54 | 0.93 | + | # TGACAA |
| Dpulex_dsx1-b | MatScan | caup | 49 | 53 | 0.85 | + | # TGACA |
| Dpulex_dsx1-b | MatScan | hth | 49 | 54 | 0.91 | + | # TGACAA |
| Dpulex_dsx1-b | MatScan | vis | 49 | 54 | 0.95 | + | # TGACAA |
| Dpulex_dsx1-b | MatScan | ara | 52 | 56 | 0.89 | + | # CAACA |
| Dpulex_dsx1-b | MatScan | caup | 52 | 56 | 0.87 | + | # CAACA |
| Dpulex_dsx1-b | MatScan | mirr | 52 | 56 | 0.89 | + | # CAACA |
| Dpulex_dsx1-b | MatScan | vvl | 58 | 63 | 0.87 | + | # CATGCA |
| Dpulex_dsx1-b | MatScan | Deaf1 | 79 | 84 | 1 | + | # TTCGTG |
| Dpulex_dsx1-b | MatScan | ara | 83 | 87 | 0.93 | - | # GAACA |
| Dpulex_dsx1-b | MatScan | caup | 83 | 87 | 0.88 | - | # GAACA |
| Dpulex_dsx1-b | MatScan | mirr | 83 | 87 | 0.89 | - | # GAACA |
| Dpulex_dsx1-b | MatScan | ara | 102 | 106 | 0.93 | - | # GAACA |
| Dpulex_dsx1-b | MatScan | caup | 102 | 106 | 0.88 | - | # GAACA |
| Dpulex_dsx1-b | MatScan | mirr | 102 | 106 | 0.89 | - | # GAACA |
| Dpulex_dsx1-b | MatScan | ct | 103 | 108 | 1 | - | # TTGAAC |
| Dpulex_dsx1-b | MatScan | ct | 104 | 109 | 0.89 | + | # TTCAAC |
| Dpulex_dsx1-b | MatScan | ara | 106 | 110 | 0.89 | + | # CAACA |
| Dpulex_dsx1-b | MatScan | caup | 106 | 110 | 0.87 | + | # CAACA |
| Dpulex_dsx1-b | MatScan | mirr | 106 | 110 | 0.89 | + | # CAACA |
| Dpulex_dsx1-b | MatScan | slp1 | 110 | 120 | 0.86 | + | # ATGTTTTCGAT |
| Dpulex_dsx1-b | MatScan | ara | 111 | 115 | 0.99 | - | # AAACA |
| Dpulex_dsx1-b | MatScan | caup | 111 | 115 | 0.9 | - | # AAACA |
| Dpulex_dsx1-b | MatScan | mirr | 111 | 115 | 1 | - | # AAACA |
| Dpulex_dsx1-b | MatScan | Optix | 117 | 121 | 0.87 | + | # CGATA |
| Dpulex_dsx1-b | MatScan | Oct | 118 | 125 | 0.88 | + | # GATAATTT |
| Dpulex_dsx1-b | MatScan | CG4328 | 119 | 125 | 0.87 | + | # ATAATTT |
| Dpulex_dsx1-b | MatScan | PHDP | 119 | 125 | 0.93 | + | # ATAATTT |
| Dpulex_dsx1-b | MatScan | Pph13 | 119 | 125 | 0.88 | + | # ATAATTT |
| Dpulex_dsx1-b | MatScan | Dll | 120 | 126 | 0.95 | + | # TAATTTC |
| Dpulex_dsx1-b | MatScan | lbl | 120 | 125 | 0.85 | + | # TAATTT |
| Dpulex_dsx1-b | MatScan | ovo | 125 | 133 | 0.87 | - | # AGGAACTGA |
| Dpulex_dsx1-b | MatScan | prd | 125 | 133 | 0.87 | - | # AGGAACTGA |
| Dpulex_dsx1-b | MatScan | ara | 136 | 140 | 0.91 | - | # TTACA |
| Dpulex_dsx1-b | MatScan | caup | 136 | 140 | 0.87 | - | # TTACA |
| Dpulex_dsx1-b | MatScan | mirr | 136 | 140 | 0.88 | - | # TTACA |
| Dpulex_dsx1-b | MatScan | lbe | 138 | 143 | 1 | + | # TAACTA |
| Dpulex_dsx1-b | MatScan | lbl | 138 | 143 | 0.89 | + | # TAACTA |
| Dpulex_dsx1-b | MatScan | exd | 151 | 158 | 0.94 | - | # ATTTGACG |
| Dpulex_dsx1-b | MatScan | Abd-B | 176 | 182 | 0.87 | + | # TTGATGA |
| Dpulex_dsx1-b | MatScan | CG42234 | 176 | 182 | 0.91 | + | # TTGATGA |
| Dpulex_dsx1-b | MatScan | H2.0 | 176 | 182 | 0.87 | + | # TTGATGA |
| Dpulex_dsx1-b | MatScan | ct | 186 | 191 | 0.9 | + | # CTAAAC |
| Dpulex_dsx1-b | MatScan | br_Z3 | 187 | 197 | 0.88 | + | # TAAACTAGCAT |
| Dpulex_dsx1-b | MatScan | ara | 204 | 208 | 0.89 | + | # CAACA |
| Dpulex_dsx1-b | MatScan | caup | 204 | 208 | 0.87 | + | # CAACA |
| Dpulex_dsx1-b | MatScan | mirr | 204 | 208 | 0.89 | + | # CAACA |
| Dpulex_dsx1-b | MatScan | Dfd | 212 | 227 | 0.88 | - | # TGAATGATTATCAGAA |
| Dpulex_dsx1-b | MatScan | mtTFA | 213 | 223 | 0.93 | - | # TGATTATCAGA |
| Dpulex_dsx1-b | MatScan | Optix | 215 | 219 | 1 | + | # TGATA |
| Dpulex_dsx1-b | MatScan | so | 215 | 220 | 0.93 | + | # TGATAA |
| Dpulex_dsx1-b | MatScan | Oct | 216 | 223 | 0.9 | + | # GATAATCA |
| Dpulex_dsx1-b | MatScan | CG18599 | 217 | 223 | 0.86 | + | # ATAATCA |
| Dpulex_dsx1-b | MatScan | E5 | 217 | 223 | 0.85 | + | # ATAATCA |
| Dpulex_dsx1-b | MatScan | ems | 217 | 223 | 0.86 | + | # ATAATCA |
| Dpulex_dsx1-b | MatScan | eve | 217 | 223 | 0.87 | + | # ATAATCA |
| Dpulex_dsx1-b | MatScan | pb | 217 | 223 | 0.86 | + | # ATAATCA |
| Dpulex_dsx1-b | MatScan | CG4328 | 218 | 224 | 0.88 | - | # ATGATTA |
| Dpulex_dsx1-b | MatScan | Gsc | 218 | 223 | 0.89 | + | # TAATCA |
| Dpulex_dsx1-b | MatScan | lbe | 218 | 223 | 0.94 | + | # TAATCA |
| Dpulex_dsx1-b | MatScan | lbl | 218 | 223 | 0.92 | + | # TAATCA |
| Dpulex_dsx1-b | MatScan | onecut | 218 | 224 | 0.87 | - | # ATGATTA |
| Dpulex_dsx1-b | MatScan | vvl | 218 | 223 | 0.88 | + | # TAATCA |
| Dpulex_dsx1-b | MatScan | fkh | 230 | 240 | 0.87 | + | # AGTTTACATAA |
| Dpulex_dsx1-b | MatScan | CG11617 | 232 | 238 | 0.94 | + | # TTTACAT |
| Dpulex_dsx1-b | MatScan | ara | 233 | 237 | 0.91 | + | # TTACA |
| Dpulex_dsx1-b | MatScan | caup | 233 | 237 | 0.87 | + | # TTACA |
| Dpulex_dsx1-b | MatScan | mirr | 233 | 237 | 0.88 | + | # TTACA |
| Dpulex_dsx1-b | MatScan | Abd-B | 235 | 241 | 0.92 | - | # TTTATGT |
| Dpulex_dsx1-b | MatScan | exd | 243 | 250 | 1 | + | # TTTTGACA |
| Dpulex_dsx1-b | MatScan | CG11617 | 245 | 251 | 0.92 | + | # TTGACAA |
| Dpulex_dsx1-b | MatScan | achi | 246 | 251 | 0.93 | + | # TGACAA |
| Dpulex_dsx1-b | MatScan | caup | 246 | 250 | 0.85 | + | # TGACA |
| Dpulex_dsx1-b | MatScan | hth | 246 | 251 | 0.91 | + | # TGACAA |
| Dpulex_dsx1-b | MatScan | vis | 246 | 251 | 0.95 | + | # TGACAA |
| Dpulex_dsx1-b | MatScan | ara | 249 | 253 | 0.89 | + | # CAACA |
| Dpulex_dsx1-b | MatScan | caup | 249 | 253 | 0.87 | + | # CAACA |
| Dpulex_dsx1-b | MatScan | mirr | 249 | 253 | 0.89 | + | # CAACA |
| Dpulex_dsx1-b | MatScan | Dfd | 258 | 273 | 0.88 | - | # ATGTTGATTAACACAC |
| Dpulex_dsx1-b | MatScan | CG11617 | 260 | 266 | 0.99 | - | # TTAACAC |
| Dpulex_dsx1-b | MatScan | ara | 261 | 265 | 1 | - | # TAACA |
| Dpulex_dsx1-b | MatScan | caup | 261 | 265 | 1 | - | # TAACA |
| Dpulex_dsx1-b | MatScan | dri | 261 | 270 | 0.91 | - | # TTGATTAACA |
| Dpulex_dsx1-b | MatScan | mirr | 261 | 265 | 0.99 | - | # TAACA |
| Dpulex_dsx1-b | MatScan | Oct | 262 | 269 | 0.92 | + | # GTTAATCA |
| Dpulex_dsx1-b | MatScan | abd-A | 263 | 269 | 0.89 | + | # TTAATCA |
| Dpulex_dsx1-b | MatScan | al | 263 | 269 | 0.85 | - | # TGATTAA |
| Dpulex_dsx1-b | MatScan | Antp | 263 | 269 | 0.91 | + | # TTAATCA |
| Dpulex_dsx1-b | MatScan | ap | 263 | 269 | 0.86 | + | # TTAATCA |
| Dpulex_dsx1-b | MatScan | Awh | 263 | 269 | 0.87 | + | # TTAATCA |
| Dpulex_dsx1-b | MatScan | bsh | 263 | 269 | 0.93 | + | # TTAATCA |
| Dpulex_dsx1-b | MatScan | btn | 263 | 269 | 0.89 | + | # TTAATCA |
| Dpulex_dsx1-b | MatScan | C15 | 263 | 269 | 0.93 | + | # TTAATCA |
| Dpulex_dsx1-b | MatScan | CG18599 | 263 | 269 | 0.9 | + | # TTAATCA |
| Dpulex_dsx1-b | MatScan | CG42234 | 263 | 269 | 0.91 | + | # TTAATCA |
| Dpulex_dsx1-b | MatScan | Dfd | 263 | 269 | 0.87 | + | # TTAATCA |
| Dpulex_dsx1-b | MatScan | E5 | 263 | 269 | 0.91 | + | # TTAATCA |
| Dpulex_dsx1-b | MatScan | ems | 263 | 269 | 0.92 | + | # TTAATCA |
| Dpulex_dsx1-b | MatScan | eve | 263 | 269 | 0.92 | + | # TTAATCA |
| Dpulex_dsx1-b | MatScan | ftz | 263 | 269 | 0.92 | + | # TTAATCA |
| Dpulex_dsx1-b | MatScan | H2.0 | 263 | 269 | 0.91 | + | # TTAATCA |
| Dpulex_dsx1-b | MatScan | HGTX | 263 | 269 | 0.89 | + | # TTAATCA |
| Dpulex_dsx1-b | MatScan | ind | 263 | 269 | 0.87 | + | # TTAATCA |
| Dpulex_dsx1-b | MatScan | lab | 263 | 269 | 0.9 | + | # TTAATCA |
| Dpulex_dsx1-b | MatScan | Lim1 | 263 | 269 | 0.85 | + | # TTAATCA |
| Dpulex_dsx1-b | MatScan | Lim3 | 263 | 269 | 0.89 | + | # TTAATCA |
| Dpulex_dsx1-b | MatScan | otp | 263 | 269 | 0.88 | + | # TTAATCA |
| Dpulex_dsx1-b | MatScan | pb | 263 | 269 | 0.91 | + | # TTAATCA |
| Dpulex_dsx1-b | MatScan | Ptx1 | 263 | 269 | 0.86 | + | # TTAATCA |
| Dpulex_dsx1-b | MatScan | Scr | 263 | 269 | 0.88 | + | # TTAATCA |
| Dpulex_dsx1-b | MatScan | slou | 263 | 269 | 0.89 | + | # TTAATCA |
| Dpulex_dsx1-b | MatScan | Vsx1 | 263 | 269 | 0.87 | + | # TTAATCA |
| Dpulex_dsx1-b | MatScan | zen2 | 263 | 269 | 0.89 | + | # TTAATCA |
| Dpulex_dsx1-b | MatScan | abd-A | 264 | 270 | 0.85 | - | # TTGATTA |
| Dpulex_dsx1-b | MatScan | al | 264 | 270 | 0.85 | + | # TAATCAA |
| Dpulex_dsx1-b | MatScan | Awh | 264 | 270 | 0.89 | - | # TTGATTA |
| Dpulex_dsx1-b | MatScan | cad | 264 | 270 | 0.87 | - | # TTGATTA |
| Dpulex_dsx1-b | MatScan | CG15696 | 264 | 270 | 0.92 | - | # TTGATTA |
| Dpulex_dsx1-b | MatScan | CG32105 | 264 | 270 | 0.85 | - | # TTGATTA |
| Dpulex_dsx1-b | MatScan | CG42234 | 264 | 270 | 0.93 | - | # TTGATTA |
| Dpulex_dsx1-b | MatScan | CG4328 | 264 | 270 | 0.89 | - | # TTGATTA |
| Dpulex_dsx1-b | MatScan | Gsc | 264 | 269 | 0.89 | + | # TAATCA |
| Dpulex_dsx1-b | MatScan | H2.0 | 264 | 270 | 0.91 | - | # TTGATTA |
| Dpulex_dsx1-b | MatScan | lbe | 264 | 269 | 0.94 | + | # TAATCA |
| Dpulex_dsx1-b | MatScan | lbl | 264 | 269 | 0.92 | + | # TAATCA |
| Dpulex_dsx1-b | MatScan | Lim1 | 264 | 270 | 0.85 | - | # TTGATTA |
| Dpulex_dsx1-b | MatScan | Lim3 | 264 | 270 | 0.88 | - | # TTGATTA |
| Dpulex_dsx1-b | MatScan | onecut | 264 | 270 | 0.99 | - | # TTGATTA |
| Dpulex_dsx1-b | MatScan | vvl | 264 | 269 | 0.88 | + | # TAATCA |
| Dpulex_dsx1-b | MatScan | ara | 268 | 272 | 0.89 | + | # CAACA |
| Dpulex_dsx1-b | MatScan | caup | 268 | 272 | 0.87 | + | # CAACA |
| Dpulex_dsx1-b | MatScan | mirr | 268 | 272 | 0.89 | + | # CAACA |
| Dpulex_dsx1-b | MatScan | ovo | 287 | 295 | 0.87 | - | # AGGAACAGC |
| Dpulex_dsx1-b | MatScan | prd | 287 | 295 | 0.87 | - | # AGGAACAGC |
| Dpulex_dsx1-b | MatScan | ara | 289 | 293 | 0.93 | - | # GAACA |
| Dpulex_dsx1-b | MatScan | caup | 289 | 293 | 0.88 | - | # GAACA |
| Dpulex_dsx1-b | MatScan | mirr | 289 | 293 | 0.89 | - | # GAACA |
| Dpulex_dsx1-b | MatScan | achi | 294 | 299 | 1 | - | # TGACAG |
| Dpulex_dsx1-b | MatScan | CG11617 | 294 | 300 | 0.92 | - | # TTGACAG |
| Dpulex_dsx1-b | MatScan | hth | 294 | 299 | 1 | - | # TGACAG |
| Dpulex_dsx1-b | MatScan | vis | 294 | 299 | 1 | - | # TGACAG |
| Dpulex_dsx1-b | MatScan | caup | 295 | 299 | 0.85 | - | # TGACA |
| Dpulex_dsx1-b | MatScan | exd | 295 | 302 | 0.87 | - | # GATTGACA |
| Dpulex_dsx1-b | MatScan | C15 | 301 | 307 | 0.85 | - | # TTAAGGA |
| Dpulex_dsx1-b | MatScan | lbe | 301 | 306 | 0.85 | - | # TAAGGA |
| Dpulex_dsx1-b | MatScan | Oct | 303 | 310 | 0.92 | + | # CTTAATGA |
| Dpulex_dsx1-b | MatScan | Ubx | 303 | 310 | 0.91 | + | # CTTAATGA |
| Dpulex_dsx1-b | MatScan | abd-A | 304 | 310 | 0.95 | + | # TTAATGA |
| Dpulex_dsx1-b | MatScan | Abd-B | 304 | 310 | 0.91 | + | # TTAATGA |
| Dpulex_dsx1-b | MatScan | al | 304 | 310 | 0.85 | - | # TCATTAA |
| Dpulex_dsx1-b | MatScan | Antp | 304 | 310 | 1 | + | # TTAATGA |
| Dpulex_dsx1-b | MatScan | ap | 304 | 310 | 0.91 | + | # TTAATGA |
| Dpulex_dsx1-b | MatScan | Awh | 304 | 310 | 0.9 | + | # TTAATGA |
| Dpulex_dsx1-b | MatScan | bsh | 304 | 310 | 0.97 | + | # TTAATGA |
| Dpulex_dsx1-b | MatScan | btn | 304 | 310 | 1 | + | # TTAATGA |
| Dpulex_dsx1-b | MatScan | C15 | 304 | 310 | 0.96 | + | # TTAATGA |
| Dpulex_dsx1-b | MatScan | CG13424 | 304 | 310 | 0.88 | + | # TTAATGA |
| Dpulex_dsx1-b | MatScan | CG18599 | 304 | 310 | 0.95 | + | # TTAATGA |
| Dpulex_dsx1-b | MatScan | CG32105 | 304 | 310 | 0.85 | + | # TTAATGA |
| Dpulex_dsx1-b | MatScan | CG32532 | 304 | 310 | 0.86 | + | # TTAATGA |
| Dpulex_dsx1-b | MatScan | CG42234 | 304 | 310 | 0.96 | + | # TTAATGA |
| Dpulex_dsx1-b | MatScan | CG4328 | 304 | 310 | 0.85 | + | # TTAATGA |
| Dpulex_dsx1-b | MatScan | Dfd | 304 | 310 | 1 | + | # TTAATGA |
| Dpulex_dsx1-b | MatScan | E5 | 304 | 310 | 0.97 | + | # TTAATGA |
| Dpulex_dsx1-b | MatScan | ems | 304 | 310 | 1 | + | # TTAATGA |
| Dpulex_dsx1-b | MatScan | en | 304 | 310 | 0.86 | + | # TTAATGA |
| Dpulex_dsx1-b | MatScan | eve | 304 | 310 | 0.99 | + | # TTAATGA |
| Dpulex_dsx1-b | MatScan | ftz | 304 | 310 | 1 | + | # TTAATGA |
| Dpulex_dsx1-b | MatScan | H2.0 | 304 | 310 | 0.96 | + | # TTAATGA |
| Dpulex_dsx1-b | MatScan | HGTX | 304 | 310 | 0.94 | + | # TTAATGA |
| Dpulex_dsx1-b | MatScan | ind | 304 | 310 | 0.94 | + | # TTAATGA |
| Dpulex_dsx1-b | MatScan | lab | 304 | 310 | 0.96 | + | # TTAATGA |
| Dpulex_dsx1-b | MatScan | Lim1 | 304 | 310 | 0.85 | + | # TTAATGA |
| Dpulex_dsx1-b | MatScan | Lim3 | 304 | 310 | 0.91 | + | # TTAATGA |
| Dpulex_dsx1-b | MatScan | NK7.1 | 304 | 310 | 0.88 | + | # TTAATGA |
| Dpulex_dsx1-b | MatScan | otp | 304 | 310 | 0.91 | + | # TTAATGA |
| Dpulex_dsx1-b | MatScan | pb | 304 | 310 | 0.99 | + | # TTAATGA |
| Dpulex_dsx1-b | MatScan | PHDP | 304 | 310 | 0.85 | + | # TTAATGA |
| Dpulex_dsx1-b | MatScan | Scr | 304 | 310 | 1 | + | # TTAATGA |
| Dpulex_dsx1-b | MatScan | slou | 304 | 310 | 0.93 | + | # TTAATGA |
| Dpulex_dsx1-b | MatScan | tup | 304 | 310 | 0.87 | + | # TTAATGA |
| Dpulex_dsx1-b | MatScan | unpg | 304 | 310 | 0.86 | + | # TTAATGA |
| Dpulex_dsx1-b | MatScan | Vsx1 | 304 | 310 | 0.89 | + | # TTAATGA |
| Dpulex_dsx1-b | MatScan | zen | 304 | 310 | 0.96 | + | # TTAATGA |
| Dpulex_dsx1-b | MatScan | zen2 | 304 | 310 | 0.96 | + | # TTAATGA |
| Dpulex_dsx1-b | MatScan | CG4328 | 305 | 311 | 0.88 | - | # ATCATTA |
| Dpulex_dsx1-b | MatScan | lbe | 305 | 310 | 0.92 | + | # TAATGA |
| Dpulex_dsx1-b | MatScan | lbl | 305 | 310 | 0.96 | + | # TAATGA |
| Dpulex_dsx1-b | MatScan | Optix | 308 | 312 | 1 | + | # TGATA |
| Dpulex_dsx1-b | MatScan | Six4 | 308 | 313 | 1 | + | # TGATAC |
| Dpulex_dsx1-b | MatScan | so | 308 | 313 | 1 | + | # TGATAC |
| Dpulex_dsx1-b | MatScan | ara | 310 | 314 | 0.91 | + | # ATACA |
| Dpulex_dsx1-b | MatScan | mirr | 310 | 314 | 0.89 | + | # ATACA |
| Dpulex_dsx1-b | MatScan | vvl | 313 | 318 | 0.87 | + | # CATGCA |
| Dpulex_dsx1-b | MatScan | vvl | 315 | 320 | 1 | - | # TATGCA |
| Dpulex_dsx1-b | MatScan | Abd-B | 316 | 322 | 0.89 | - | # TTTATGC |
| Dpulex_dsx1-b | MatScan | hb | 316 | 325 | 0.88 | + | # GCATAAAACA |
| Dpulex_dsx1-b | MatScan | ara | 321 | 325 | 0.99 | + | # AAACA |
| Dpulex_dsx1-b | MatScan | caup | 321 | 325 | 0.9 | + | # AAACA |
| Dpulex_dsx1-b | MatScan | mirr | 321 | 325 | 1 | + | # AAACA |
| Dpulex_dsx1-b | MatScan | ara | 324 | 328 | 0.89 | + | # CAACA |
| Dpulex_dsx1-b | MatScan | caup | 324 | 328 | 0.87 | + | # CAACA |
| Dpulex_dsx1-b | MatScan | mirr | 324 | 328 | 0.89 | + | # CAACA |
| Dpulex_dsx1-b | MatScan | Abd-B | 328 | 334 | 0.88 | - | # TTTATTT |
| Dpulex_dsx1-b | MatScan | cad | 328 | 334 | 0.91 | - | # TTTATTT |
| Dpulex_dsx1-b | MatScan | CG42234 | 328 | 334 | 0.85 | - | # TTTATTT |
| Dpulex_dsx1-b | MatScan | CG4328 | 328 | 334 | 0.9 | - | # TTTATTT |
| Dpulex_dsx1-b | MatScan | lbe | 331 | 336 | 0.91 | + | # TAAATA |
| Dpulex_dsx1-b | MatScan | Dfd | 334 | 349 | 0.88 | + | # ATACTTATTAATACTA |
| Dpulex_dsx1-b | MatScan | lbe | 335 | 340 | 0.92 | - | # TAAGTA |
| Dpulex_dsx1-b | MatScan | Dfd | 336 | 351 | 0.89 | - | # TGTAGTATTAATAAGT |
| Dpulex_dsx1-b | MatScan | cad | 337 | 343 | 0.86 | + | # CTTATTA |
| Dpulex_dsx1-b | MatScan | CG4328 | 337 | 343 | 0.93 | + | # CTTATTA |
| Dpulex_dsx1-b | MatScan | CG9876 | 337 | 343 | 0.86 | + | # CTTATTA |
| Dpulex_dsx1-b | MatScan | dri | 337 | 346 | 0.85 | + | # CTTATTAATA |
| Dpulex_dsx1-b | MatScan | H2.0 | 337 | 343 | 0.89 | + | # CTTATTA |
| Dpulex_dsx1-b | MatScan | Lim3 | 337 | 343 | 0.85 | + | # CTTATTA |
| Dpulex_dsx1-b | MatScan | abd-A | 338 | 344 | 0.89 | - | # TTAATAA |
| Dpulex_dsx1-b | MatScan | al | 338 | 344 | 0.85 | + | # TTATTAA |
| Dpulex_dsx1-b | MatScan | Antp | 338 | 344 | 0.91 | - | # TTAATAA |
| Dpulex_dsx1-b | MatScan | ap | 338 | 344 | 0.86 | - | # TTAATAA |
| Dpulex_dsx1-b | MatScan | Awh | 338 | 344 | 0.87 | - | # TTAATAA |
| Dpulex_dsx1-b | MatScan | bsh | 338 | 344 | 0.9 | - | # TTAATAA |
| Dpulex_dsx1-b | MatScan | btn | 338 | 344 | 0.89 | - | # TTAATAA |
| Dpulex_dsx1-b | MatScan | C15 | 338 | 344 | 0.9 | - | # TTAATAA |
| Dpulex_dsx1-b | MatScan | CG13424 | 338 | 344 | 0.87 | - | # TTAATAA |
| Dpulex_dsx1-b | MatScan | CG18599 | 338 | 344 | 0.89 | - | # TTAATAA |
| Dpulex_dsx1-b | MatScan | CG42234 | 338 | 344 | 0.89 | - | # TTAATAA |
| Dpulex_dsx1-b | MatScan | CG7056 | 338 | 345 | 0.88 | - | # ATTAATAA |
| Dpulex_dsx1-b | MatScan | Dfd | 338 | 344 | 0.88 | - | # TTAATAA |
| Dpulex_dsx1-b | MatScan | E5 | 338 | 344 | 0.93 | - | # TTAATAA |
| Dpulex_dsx1-b | MatScan | ems | 338 | 344 | 0.91 | - | # TTAATAA |
| Dpulex_dsx1-b | MatScan | eve | 338 | 344 | 0.9 | - | # TTAATAA |
| Dpulex_dsx1-b | MatScan | ftz | 338 | 344 | 0.92 | - | # TTAATAA |
| Dpulex_dsx1-b | MatScan | H2.0 | 338 | 344 | 0.96 | - | # TTAATAA |
| Dpulex_dsx1-b | MatScan | HGTX | 338 | 344 | 0.9 | - | # TTAATAA |
| Dpulex_dsx1-b | MatScan | ind | 338 | 344 | 0.87 | - | # TTAATAA |
| Dpulex_dsx1-b | MatScan | lab | 338 | 344 | 0.9 | - | # TTAATAA |
| Dpulex_dsx1-b | MatScan | lbe | 338 | 343 | 0.93 | - | # TAATAA |
| Dpulex_dsx1-b | MatScan | lbl | 338 | 343 | 0.9 | - | # TAATAA |
| Dpulex_dsx1-b | MatScan | Lim1 | 338 | 344 | 0.85 | - | # TTAATAA |
| Dpulex_dsx1-b | MatScan | Lim3 | 338 | 344 | 0.87 | - | # TTAATAA |
| Dpulex_dsx1-b | MatScan | NK7.1 | 338 | 344 | 0.87 | - | # TTAATAA |
| Dpulex_dsx1-b | MatScan | Oct | 338 | 345 | 0.88 | - | # ATTAATAA |
| Dpulex_dsx1-b | MatScan | otp | 338 | 344 | 0.87 | - | # TTAATAA |
| Dpulex_dsx1-b | MatScan | pb | 338 | 344 | 0.91 | - | # TTAATAA |
| Dpulex_dsx1-b | MatScan | PHDP | 338 | 344 | 0.85 | - | # TTAATAA |
| Dpulex_dsx1-b | MatScan | Scr | 338 | 344 | 0.88 | - | # TTAATAA |
| Dpulex_dsx1-b | MatScan | slou | 338 | 344 | 0.9 | - | # TTAATAA |
| Dpulex_dsx1-b | MatScan | Vsx1 | 338 | 344 | 0.89 | - | # TTAATAA |
| Dpulex_dsx1-b | MatScan | zen2 | 338 | 344 | 0.89 | - | # TTAATAA |
| Dpulex_dsx1-b | MatScan | vvl | 339 | 344 | 0.88 | + | # TATTAA |
| Dpulex_dsx1-b | MatScan | vvl | 341 | 346 | 0.88 | - | # TATTAA |
| Dpulex_dsx1-b | MatScan | ara | 353 | 357 | 0.99 | - | # AAACA |
| Dpulex_dsx1-b | MatScan | caup | 353 | 357 | 0.9 | - | # AAACA |
| Dpulex_dsx1-b | MatScan | fkh | 353 | 363 | 0.92 | + | # TGTTTGGATAA |
| Dpulex_dsx1-b | MatScan | mirr | 353 | 357 | 1 | - | # AAACA |
| Dpulex_dsx1-b | MatScan | fkh | 356 | 366 | 0.86 | - | # TATTTATCCAA |
| Dpulex_dsx1-b | MatScan | vvl | 357 | 362 | 0.88 | - | # TATCCA |
| Dpulex_dsx1-b | MatScan | bcd | 358 | 363 | 0.86 | - | # TTATCC |
| Dpulex_dsx1-b | MatScan | lbe | 361 | 366 | 0.91 | + | # TAAATA |
| Dpulex_dsx1-b | MatScan | ara | 376 | 380 | 0.89 | - | # CAACA |
| Dpulex_dsx1-b | MatScan | caup | 376 | 380 | 0.87 | - | # CAACA |
| Dpulex_dsx1-b | MatScan | mirr | 376 | 380 | 0.89 | - | # CAACA |
| Dpulex_dsx1-b | MatScan | ct | 377 | 382 | 0.89 | - | # TTCAAC |
| Dpulex_dsx1-b | MatScan | CG11617 | 394 | 400 | 0.94 | + | # TTTACAT |
| Dpulex_dsx1-b | MatScan | ara | 395 | 399 | 0.91 | + | # TTACA |
| Dpulex_dsx1-b | MatScan | caup | 395 | 399 | 0.87 | + | # TTACA |
| Dpulex_dsx1-b | MatScan | mirr | 395 | 399 | 0.88 | + | # TTACA |
| Dpulex_dsx1-b | MatScan | lbe | 419 | 424 | 0.86 | + | # TAAGAA |
| Dpulex_dsx1-b | MatScan | Dfd | 426 | 441 | 0.87 | - | # CGAAGAATTATCTCAA |
| Dpulex_dsx1-b | MatScan | mtTFA | 427 | 437 | 0.86 | - | # GAATTATCTCA |
| Dpulex_dsx1-b | MatScan | Oct | 430 | 437 | 0.85 | + | # GATAATTC |
| Dpulex_dsx1-b | MatScan | CG4328 | 431 | 437 | 0.87 | + | # ATAATTC |
| Dpulex_dsx1-b | MatScan | PHDP | 431 | 437 | 0.9 | + | # ATAATTC |
| Dpulex_dsx1-b | MatScan | Pph13 | 431 | 437 | 0.88 | + | # ATAATTC |
| Dpulex_dsx1-b | MatScan | Dll | 432 | 438 | 0.88 | + | # TAATTCT |
| Dpulex_dsx1-b | MatScan | lbl | 432 | 437 | 0.85 | + | # TAATTC |
| Dpulex_dsx1-b | MatScan | sd | 448 | 459 | 0.89 | + | # GACATTTTACAT |
| Dpulex_dsx1-b | MatScan | CG11617 | 453 | 459 | 0.94 | + | # TTTACAT |
| Dpulex_dsx1-b | MatScan | ara | 454 | 458 | 0.91 | + | # TTACA |
| Dpulex_dsx1-b | MatScan | caup | 454 | 458 | 0.87 | + | # TTACA |
| Dpulex_dsx1-b | MatScan | mirr | 454 | 458 | 0.88 | + | # TTACA |
| Dpulex_dsx1-b | MatScan | ara | 460 | 464 | 0.89 | + | # CAACA |
| Dpulex_dsx1-b | MatScan | caup | 460 | 464 | 0.87 | + | # CAACA |
| Dpulex_dsx1-b | MatScan | mirr | 460 | 464 | 0.89 | + | # CAACA |
| Dpulex_dsx1-b | MatScan | vvl | 471 | 476 | 0.9 | + | # TATGAA |
| Dpulex_dsx1-b | MatScan | Six4 | 473 | 478 | 0.92 | + | # TGAAAC |
| Dpulex_dsx1-b | MatScan | ara | 475 | 479 | 0.99 | + | # AAACA |
| Dpulex_dsx1-b | MatScan | caup | 475 | 479 | 0.9 | + | # AAACA |
| Dpulex_dsx1-b | MatScan | mirr | 475 | 479 | 1 | + | # AAACA |
| Dpulex_dsx1-b | MatScan | Six4 | 495 | 500 | 0.92 | - | # TGAAAC |
| Dpulex_dsx1-b | MatScan | exd | 496 | 503 | 0.88 | - | # GTTTGAAA |
| Dpulex_dsx1-b | MatScan | pan | 496 | 503 | 0.85 | - | # GTTTGAAA |
| Dpulex_dsx1-b | MatScan | ara | 500 | 504 | 0.99 | + | # AAACA |
| Dpulex_dsx1-b | MatScan | caup | 500 | 504 | 0.9 | + | # AAACA |
| Dpulex_dsx1-b | MatScan | mirr | 500 | 504 | 1 | + | # AAACA |
| Dpulex_dsx1-b | MatScan | ara | 503 | 507 | 0.89 | + | # CAACA |
| Dpulex_dsx1-b | MatScan | caup | 503 | 507 | 0.87 | + | # CAACA |
| Dpulex_dsx1-b | MatScan | mirr | 503 | 507 | 0.89 | + | # CAACA |
| Dpulex_dsx1-b | MatScan | br_Z2 | 516 | 523 | 0.88 | + | # TTCTATAT |
| Dpulex_dsx1-b | MatScan | Cf2_II | 516 | 525 | 0.85 | - | # GTATATAGAA |
| Dpulex_dsx1-b | MatScan | CF2-II | 517 | 525 | 0.88 | - | # GTATATAGA |
| Dpulex_dsx1-b | MatScan | CF2-II | 517 | 525 | 0.88 | - | # GTATATAGA |
| Dpulex_dsx1-b | MatScan | onecut | 526 | 532 | 0.87 | + | # GTGATTT |
| Dpulex_dsx1-b | MatScan | slbo | 529 | 536 | 0.9 | + | # ATTTCAAA |
| Dpulex_dsx1-b | MatScan | exd | 530 | 537 | 0.88 | - | # ATTTGAAA |
| Dpulex_dsx1-b | MatScan | pan | 530 | 537 | 0.86 | - | # ATTTGAAA |
| Dpulex_dsx1-b | MatScan | achi | 539 | 544 | 0.92 | + | # TGACAT |
| Dpulex_dsx1-b | MatScan | caup | 539 | 543 | 0.85 | + | # TGACA |
| Dpulex_dsx1-b | MatScan | hth | 539 | 544 | 0.96 | + | # TGACAT |
| Dpulex_dsx1-b | MatScan | vis | 539 | 544 | 0.95 | + | # TGACAT |
| Dpulex_dsx1-b | MatScan | Dfd | 556 | 571 | 0.92 | + | # AGGCGCATTACTTATC |
| Dpulex_dsx1-b | MatScan | al | 563 | 569 | 0.85 | - | # TAAGTAA |
| Dpulex_dsx1-b | MatScan | Lim1 | 563 | 569 | 0.85 | + | # TTACTTA |
| Dpulex_dsx1-b | MatScan | lbe | 564 | 569 | 0.92 | - | # TAAGTA |
| Dpulex_dsx1-b | MatScan | mtTFA | 564 | 574 | 0.96 | + | # TACTTATCATT |
| Dpulex_dsx1-b | MatScan | so | 567 | 572 | 0.93 | - | # TGATAA |
| Dpulex_dsx1-b | MatScan | Optix | 568 | 572 | 1 | - | # TGATA |
| Dpulex_dsx1-b | MatScan | br_Z3 | 573 | 583 | 0.91 | - | # AAAACTAAAAA |
| Dpulex_dsx1-b | MatScan | lbe | 573 | 578 | 0.85 | - | # TAAAAA |
| Dpulex_dsx1-b | MatScan | exd | 580 | 587 | 0.9 | + | # TTTTGACT |
| Dpulex_dsx1-b | MatScan | achi | 593 | 598 | 0.9 | - | # TGACAC |
| Dpulex_dsx1-b | MatScan | hth | 593 | 598 | 0.91 | - | # TGACAC |
| Dpulex_dsx1-b | MatScan | Six4 | 593 | 598 | 0.98 | - | # TGACAC |
| Dpulex_dsx1-b | MatScan | vis | 593 | 598 | 0.97 | - | # TGACAC |
| Dpulex_dsx1-b | MatScan | caup | 594 | 598 | 0.85 | - | # TGACA |
| Dpulex_dsx1-b | MatScan | ct | 599 | 604 | 0.86 | + | # GTGAAC |
| Dpulex_dsx1-b | MatScan | Lag1 | 606 | 612 | 0.91 | - | # CAACCAA |
| Dpulex_dsx1-b | MatScan | ct | 609 | 614 | 0.89 | - | # TTCAAC |
| Dpulex_dsx1-b | MatScan | ara | 622 | 626 | 0.99 | - | # AAACA |
| Dpulex_dsx1-b | MatScan | caup | 622 | 626 | 0.9 | - | # AAACA |
| Dpulex_dsx1-b | MatScan | mirr | 622 | 626 | 1 | - | # AAACA |
| Dpulex_dsx1-b | MatScan | Six4 | 623 | 628 | 0.92 | - | # TGAAAC |
| Dpulex_dsx1-b | MatScan | exd | 624 | 631 | 0.89 | - | # TTTTGAAA |
| Dpulex_dsx1-b | MatScan | pan | 624 | 631 | 0.89 | - | # TTTTGAAA |
| Dpulex_dsx1-b | MatScan | CG11617 | 631 | 637 | 0.94 | - | # TTTACAT |
| Dpulex_dsx1-b | MatScan | ara | 632 | 636 | 0.91 | - | # TTACA |
| Dpulex_dsx1-b | MatScan | caup | 632 | 636 | 0.87 | - | # TTACA |
| Dpulex_dsx1-b | MatScan | mirr | 632 | 636 | 0.88 | - | # TTACA |
| Dpulex_dsx1-b | MatScan | br_Z3 | 635 | 645 | 0.85 | + | # AAAACAAAATT |
| Dpulex_dsx1-b | MatScan | ara | 636 | 640 | 0.99 | + | # AAACA |
| Dpulex_dsx1-b | MatScan | caup | 636 | 640 | 0.9 | + | # AAACA |
| Dpulex_dsx1-b | MatScan | mirr | 636 | 640 | 1 | + | # AAACA |
| Dpulex_dsx1-b | MatScan | CG4328 | 640 | 646 | 0.87 | + | # AAAATTG |
| Dpulex_dsx1-b | MatScan | slbo | 643 | 650 | 0.93 | + | # ATTGCGAA |
| Dpulex_dsx1-b | MatScan | dl | 645 | 655 | 0.96 | + | # TGCGAAAAACC |
| Dpulex_dsx1-b | MatScan | dl | 646 | 656 | 0.89 | - | # GGGTTTTTCGC |
| Dpulex_dsx1-b | MatScan | dl_1 | 646 | 657 | 0.89 | - | # TGGGTTTTTCGC |
| Dpulex_dsx1-b | MatScan | pan | 660 | 667 | 0.9 | - | # TTTTGGTC |
| Dpulex_dsx1-b | MatScan | br_Z2 | 679 | 686 | 0.88 | - | # AGCTATTT |
| Dpulex_dsx1-b | MatScan | Zeste | 684 | 699 | 0.89 | + | # GCTATTGAGTGACCCC |
| Dpulex_dsx1-b | MatScan | z | 688 | 697 | 0.91 | + | # TTGAGTGACC |
| Dpulex_dsx1-b | MatScan | usp | 690 | 699 | 0.92 | - | # GGGGTCACTC |
| Dpulex_dsx1-b | MatScan | CF1 | 691 | 699 | 0.94 | - | # GGGGTCACT |
| Dpulex_dsx1-b | MatScan | CF1 | 691 | 699 | 0.94 | - | # GGGGTCACT |
| Dpulex_dsx1-b | MatScan | lbe | 702 | 707 | 0.86 | - | # TAAGAA |
| Dpulex_dsx1-b | MatScan | B-H1 | 705 | 711 | 0.96 | + | # TTAAATG |
| Dpulex_dsx1-b | MatScan | B-H2 | 705 | 711 | 0.91 | + | # TTAAATG |
| Dpulex_dsx1-b | MatScan | C15 | 705 | 711 | 0.94 | + | # TTAAATG |
| Dpulex_dsx1-b | MatScan | CG11085 | 705 | 711 | 0.86 | + | # TTAAATG |
| Dpulex_dsx1-b | MatScan | CG34031 | 705 | 711 | 0.87 | + | # TTAAATG |
| Dpulex_dsx1-b | MatScan | Hmx | 705 | 711 | 0.86 | + | # TTAAATG |
| Dpulex_dsx1-b | MatScan | NK7.1 | 705 | 711 | 0.89 | + | # TTAAATG |
| Dpulex_dsx1-b | MatScan | Deaf1 | 715 | 720 | 0.96 | + | # TTCGTT |
| Dpulex_dsx1-b | MatScan | ct | 718 | 723 | 0.89 | - | # TTCAAC |
| Dpulex_dsx1-b | MatScan | ct | 719 | 724 | 1 | + | # TTGAAC |
| Dpulex_dsx1-b | MatScan | ara | 721 | 725 | 0.93 | + | # GAACA |
| Dpulex_dsx1-b | MatScan | caup | 721 | 725 | 0.88 | + | # GAACA |
| Dpulex_dsx1-b | MatScan | mirr | 721 | 725 | 0.89 | + | # GAACA |
| Dpulex_dsx1-b | MatScan | dl | 733 | 743 | 0.92 | - | # CGAGAAAAACT |
| Dpulex_dsx1-b | MatScan | Abd-B | 741 | 747 | 0.88 | - | # TTTACGA |
| Dpulex_dsx1-b | MatScan | Abd-B | 748 | 754 | 0.88 | + | # TTTACGA |
| Dpulex_dsx1-b | MatScan | Optix | 752 | 756 | 0.87 | + | # CGATA |
| Dpulex_dsx1-b | MatScan | vvl | 757 | 762 | 0.98 | + | # TATTCA |
| Dpulex_dsx1-b | MatScan | Six4 | 773 | 778 | 0.92 | + | # TGAAAC |
| Dpulex_dsx1-b | MatScan | Croc | 785 | 800 | 0.86 | + | # CAATGTAAATATAAGT |
| Dpulex_dsx1-b | MatScan | BR-C | 786 | 798 | 0.89 | + | # AATGTAAATATAA |
| Dpulex_dsx1-b | MatScan | slp1 | 786 | 796 | 0.86 | - | # ATATTTACATT |
| Dpulex_dsx1-b | MatScan | CG11617 | 787 | 793 | 0.94 | - | # TTTACAT |
| Dpulex_dsx1-b | MatScan | ara | 788 | 792 | 0.91 | - | # TTACA |
| Dpulex_dsx1-b | MatScan | caup | 788 | 792 | 0.87 | - | # TTACA |
| Dpulex_dsx1-b | MatScan | mirr | 788 | 792 | 0.88 | - | # TTACA |
| Dpulex_dsx1-b | MatScan | Cf2_II | 789 | 798 | 0.85 | + | # GTAAATATAA |
| Dpulex_dsx1-b | MatScan | Cf2_II | 789 | 798 | 0.87 | - | # TTATATTTAC |
| Dpulex_dsx1-b | MatScan | CF2-II | 789 | 797 | 0.87 | + | # GTAAATATA |
| Dpulex_dsx1-b | MatScan | CF2-II | 789 | 797 | 0.88 | + | # GTAAATATA |
| Dpulex_dsx1-b | MatScan | CF2-II | 790 | 798 | 0.86 | - | # TTATATTTA |
| Dpulex_dsx1-b | MatScan | lbe | 790 | 795 | 0.91 | + | # TAAATA |
| Dpulex_dsx1-b | MatScan | bap | 795 | 801 | 0.86 | + | # ATAAGTG |
| Dpulex_dsx1-b | MatScan | ara | 800 | 804 | 0.99 | - | # AAACA |
| Dpulex_dsx1-b | MatScan | caup | 800 | 804 | 0.9 | - | # AAACA |
| Dpulex_dsx1-b | MatScan | mirr | 800 | 804 | 1 | - | # AAACA |
| Dpulex_dsx1-b | MatScan | Deaf1 | 813 | 818 | 0.96 | + | # TTCGTT |
| Dpulex_dsx1-b | MatScan | vvl | 821 | 826 | 0.9 | + | # TATGAA |
| Dpulex_dsx1-b | MatScan | Six4 | 823 | 828 | 0.92 | + | # TGAAAC |
| Dpulex_dsx1-b | MatScan | ovo | 837 | 845 | 0.89 | - | # AGAAACAGA |
| Dpulex_dsx1-b | MatScan | prd | 837 | 845 | 0.89 | - | # AGAAACAGA |
| Dpulex_dsx1-b | MatScan | ara | 839 | 843 | 0.99 | - | # AAACA |
| Dpulex_dsx1-b | MatScan | caup | 839 | 843 | 0.9 | - | # AAACA |
| Dpulex_dsx1-b | MatScan | mirr | 839 | 843 | 1 | - | # AAACA |
| Dpulex_dsx1-b | MatScan | slbo | 857 | 864 | 1 | + | # ATTGCAAA |
| Dpulex_dsx1-b | MatScan | ttk | 863 | 871 | 0.88 | + | # AAGGATGAT |
| Dpulex_dsx1-b | MatScan | mtTFA | 866 | 876 | 0.96 | - | # TTCTTATCATC |
| Dpulex_dsx1-b | MatScan | Optix | 868 | 872 | 1 | + | # TGATA |
| Dpulex_dsx1-b | MatScan | so | 868 | 873 | 0.93 | + | # TGATAA |
| Dpulex_dsx1-b | MatScan | lbe | 871 | 876 | 0.86 | + | # TAAGAA |
| Dpulex_dsx1-b | MatScan | ara | 878 | 882 | 0.91 | + | # TTACA |
| Dpulex_dsx1-b | MatScan | caup | 878 | 882 | 0.87 | + | # TTACA |
| Dpulex_dsx1-b | MatScan | mirr | 878 | 882 | 0.88 | + | # TTACA |
| Dpulex_dsx1-b | MatScan | ara | 888 | 892 | 0.89 | - | # CAACA |
| Dpulex_dsx1-b | MatScan | caup | 888 | 892 | 0.87 | - | # CAACA |
| Dpulex_dsx1-b | MatScan | mirr | 888 | 892 | 0.89 | - | # CAACA |
| Dpulex_dsx1-b | MatScan | ara | 891 | 895 | 0.91 | - | # TTACA |
| Dpulex_dsx1-b | MatScan | caup | 891 | 895 | 0.87 | - | # TTACA |
| Dpulex_dsx1-b | MatScan | mirr | 891 | 895 | 0.88 | - | # TTACA |
| Dpulex_dsx1-b | MatScan | lbe | 893 | 898 | 0.86 | + | # TAAGAA |
| Dpulex_dsx1-b | MatScan | fkh | 903 | 913 | 0.86 | - | # TGTTTGTTGAT |
| Dpulex_dsx1-b | MatScan | ara | 905 | 909 | 0.89 | + | # CAACA |
| Dpulex_dsx1-b | MatScan | caup | 905 | 909 | 0.87 | + | # CAACA |
| Dpulex_dsx1-b | MatScan | mirr | 905 | 909 | 0.89 | + | # CAACA |
| Dpulex_dsx1-b | MatScan | ara | 909 | 913 | 0.99 | + | # AAACA |
| Dpulex_dsx1-b | MatScan | caup | 909 | 913 | 0.9 | + | # AAACA |
| Dpulex_dsx1-b | MatScan | mirr | 909 | 913 | 1 | + | # AAACA |
| Dpulex_dsx1-b | MatScan | Eip74EF | 912 | 918 | 0.88 | + | # CAGGAAA |
| Dpulex_dsx1-b | MatScan | dl_2 | 914 | 923 | 0.85 | - | # GAGGGTTTCC |
| Dpulex_dsx1-b | MatScan | ara | 943 | 947 | 0.91 | - | # ATACA |
| Dpulex_dsx1-b | MatScan | mirr | 943 | 947 | 0.89 | - | # ATACA |
| Dpulex_dsx1-b | MatScan | Six4 | 944 | 949 | 1 | - | # TGATAC |
| Dpulex_dsx1-b | MatScan | so | 944 | 949 | 1 | - | # TGATAC |
| Dpulex_dsx1-b | MatScan | Optix | 945 | 949 | 1 | - | # TGATA |
| Dpulex_dsx1-b | MatScan | Abd-B | 946 | 959 | 0.86 | - | # ACTTTTATGGTGAT |
| Dpulex_dsx1-b | MatScan | Abd-B | 950 | 956 | 0.93 | - | # TTTATGG |
| Dpulex_dsx1-b | MatScan | cad | 950 | 956 | 0.92 | - | # TTTATGG |
| Dpulex_dsx1-b | MatScan | CG42234 | 950 | 956 | 0.87 | - | # TTTATGG |
| Dpulex_dsx1-b | MatScan | CG4328 | 950 | 956 | 0.88 | - | # TTTATGG |
| Dpulex_dsx1-b | MatScan | lbe | 959 | 964 | 0.95 | + | # TAACCA |
| Dpulex_dsx1-b | MatScan | slbo | 970 | 977 | 1 | - | # ATTGCAAA |
