# Supplementary material for: Molecular cloning of doublesex genes of four cladocera (water flea) species
Source: BMC Genomics. 2013 Apr 10;14:239. doi: 10.1186/1471-2164-14-239 (PMC3637828; doi:10.1186/1471-2164-14-239)
Supplement: Additional file 20 — DapmaDsx2 TF-map. [file 1471-2164-14-239-S20.doc]

Supplemental Material 20. *DapmaDsx2* TF-map

| Column Descriptions | Promoter region ID - Species, dsx paralog number, and dsx transcript identifier  Name of program that generated results  Name of transcription factor identified  Start of transcription factor binding site (TFBS)  End of transcription factor binding site (TFBS)  Match score between known TFBS (from TFBS database) and identified Daphnia dsx promoter sequence motif  Strand on which TFBS was identified in sequence  Reading frame for CDS feature types (not used)  Sequence of transcription factor binding motif (from TFBS database) | | | | | | |
| --- | --- | --- | --- | --- | --- | --- | --- |
| Sequence ID |
| Source |
| Type (TF) |
| Start |
| End |
| Score |
| Strand |
| Phase |
| TF Binding Motif |
|  |  |  |  |  |  |  |  |
| **Sequence ID** | **Source** | **Type (TF)** | **Start** | **End** | **Score** | **Strand** | **TF Binding Motif** |
| Dmagna_dsx2 | MatScan | hb | 3 | 12 | 0.88 | - | # AAAAAAAAAA |
| Dmagna_dsx2 | MatScan | hb | 4 | 13 | 0.92 | - | # CAAAAAAAAA |
| Dmagna_dsx2 | MatScan | hb | 5 | 14 | 0.9 | - | # ACAAAAAAAA |
| Dmagna_dsx2 | MatScan | br_Z3 | 7 | 17 | 0.86 | - | # AAAACAAAAAA |
| Dmagna_dsx2 | MatScan | hb | 7 | 16 | 0.88 | - | # AAACAAAAAA |
| Dmagna_dsx2 | MatScan | slp1 | 11 | 21 | 0.9 | + | # TTGTTTTTGTT |
| Dmagna_dsx2 | MatScan | ara | 12 | 16 | 0.99 | - | # AAACA |
| Dmagna_dsx2 | MatScan | BR-C | 12 | 26 | 0.87 | - | # AACAAAACAAAAACA |
| Dmagna_dsx2 | MatScan | caup | 12 | 16 | 0.9 | - | # AAACA |
| Dmagna_dsx2 | MatScan | mirr | 12 | 16 | 1 | - | # AAACA |
| Dmagna_dsx2 | MatScan | br_Z3 | 13 | 23 | 0.88 | - | # AAAACAAAAAC |
| Dmagna_dsx2 | MatScan | ara | 18 | 22 | 0.99 | - | # AAACA |
| Dmagna_dsx2 | MatScan | caup | 18 | 22 | 0.9 | - | # AAACA |
| Dmagna_dsx2 | MatScan | mirr | 18 | 22 | 1 | - | # AAACA |
| Dmagna_dsx2 | MatScan | ara | 23 | 27 | 0.89 | - | # CAACA |
| Dmagna_dsx2 | MatScan | caup | 23 | 27 | 0.87 | - | # CAACA |
| Dmagna_dsx2 | MatScan | mirr | 23 | 27 | 0.89 | - | # CAACA |
| Dmagna_dsx2 | MatScan | achi | 64 | 69 | 0.93 | - | # TGACAA |
| Dmagna_dsx2 | MatScan | hth | 64 | 69 | 0.91 | - | # TGACAA |
| Dmagna_dsx2 | MatScan | vis | 64 | 69 | 0.95 | - | # TGACAA |
| Dmagna_dsx2 | MatScan | caup | 65 | 69 | 0.85 | - | # TGACA |
| Dmagna_dsx2 | MatScan | achi | 83 | 88 | 0.93 | - | # TGACAA |
| Dmagna_dsx2 | MatScan | CG11617 | 83 | 89 | 0.92 | - | # TTGACAA |
| Dmagna_dsx2 | MatScan | hth | 83 | 88 | 0.91 | - | # TGACAA |
| Dmagna_dsx2 | MatScan | vis | 83 | 88 | 0.95 | - | # TGACAA |
| Dmagna_dsx2 | MatScan | caup | 84 | 88 | 0.85 | - | # TGACA |
| Dmagna_dsx2 | MatScan | exd | 84 | 91 | 0.86 | - | # ACTTGACA |
| Dmagna_dsx2 | MatScan | vnd | 84 | 92 | 0.87 | + | # TGTCAAGTC |
| Dmagna_dsx2 | MatScan | Six4 | 101 | 106 | 0.95 | - | # TGAGAC |
| Dmagna_dsx2 | MatScan | ems | 104 | 110 | 0.85 | - | # CAAATGA |
| Dmagna_dsx2 | MatScan | Deaf1 | 121 | 126 | 0.93 | - | # TTCGTA |
| Dmagna_dsx2 | MatScan | ara | 134 | 138 | 0.91 | - | # TTACA |
| Dmagna_dsx2 | MatScan | caup | 134 | 138 | 0.87 | - | # TTACA |
| Dmagna_dsx2 | MatScan | mirr | 134 | 138 | 0.88 | - | # TTACA |
| Dmagna_dsx2 | MatScan | exd | 150 | 157 | 0.95 | + | # TTTTGACG |
| Dmagna_dsx2 | MatScan | B-H1 | 165 | 171 | 0.86 | - | # TTAAAAG |
| Dmagna_dsx2 | MatScan | B-H1 | 168 | 174 | 0.91 | + | # TTAAACG |
| Dmagna_dsx2 | MatScan | C15 | 168 | 174 | 0.87 | + | # TTAAACG |
| Dmagna_dsx2 | MatScan | ct | 168 | 173 | 0.98 | + | # TTAAAC |
| Dmagna_dsx2 | MatScan | lbe | 194 | 199 | 0.85 | - | # TAAAAA |
| Dmagna_dsx2 | MatScan | br_Z3 | 210 | 220 | 0.86 | - | # AAAACAAAAAT |
| Dmagna_dsx2 | MatScan | BR-C | 212 | 224 | 0.87 | - | # AATGAAAACAAAA |
| Dmagna_dsx2 | MatScan | slp1 | 214 | 224 | 0.95 | + | # TTGTTTTCATT |
| Dmagna_dsx2 | MatScan | ara | 215 | 219 | 0.99 | - | # AAACA |
| Dmagna_dsx2 | MatScan | caup | 215 | 219 | 0.9 | - | # AAACA |
| Dmagna_dsx2 | MatScan | mirr | 215 | 219 | 1 | - | # AAACA |
| Dmagna_dsx2 | MatScan | br_Z2 | 223 | 230 | 0.98 | + | # TTCTATTT |
| Dmagna_dsx2 | MatScan | inv | 224 | 231 | 0.85 | + | # TCTATTTA |
| Dmagna_dsx2 | MatScan | Vsx2 | 225 | 233 | 0.87 | - | # TTTAAATAG |
| Dmagna_dsx2 | MatScan | abd-A | 226 | 232 | 0.85 | - | # TTAAATA |
| Dmagna_dsx2 | MatScan | al | 226 | 232 | 0.85 | + | # TATTTAA |
| Dmagna_dsx2 | MatScan | C15 | 226 | 232 | 0.95 | - | # TTAAATA |
| Dmagna_dsx2 | MatScan | CG7056 | 226 | 233 | 0.87 | - | # TTTAAATA |
| Dmagna_dsx2 | MatScan | lab | 226 | 232 | 0.86 | - | # TTAAATA |
| Dmagna_dsx2 | MatScan | lbe | 226 | 231 | 0.91 | - | # TAAATA |
| Dmagna_dsx2 | MatScan | Lim1 | 226 | 232 | 0.85 | - | # TTAAATA |
| Dmagna_dsx2 | MatScan | C15 | 229 | 235 | 0.85 | + | # TTAAATT |
| Dmagna_dsx2 | MatScan | lbe | 234 | 239 | 0.85 | - | # TAAAAA |
| Dmagna_dsx2 | MatScan | sd | 236 | 247 | 0.86 | - | # ATCATTCCTAAA |
| Dmagna_dsx2 | MatScan | onecut | 246 | 252 | 0.88 | + | # ATGATTT |
| Dmagna_dsx2 | MatScan | C15 | 248 | 254 | 0.85 | - | # TTAAATC |
| Dmagna_dsx2 | MatScan | dri | 249 | 258 | 0.89 | - | # CCAATTAAAT |
| Dmagna_dsx2 | MatScan | CG7056 | 250 | 257 | 0.88 | + | # TTTAATTG |
| Dmagna_dsx2 | MatScan | inv | 250 | 257 | 0.92 | + | # TTTAATTG |
| Dmagna_dsx2 | MatScan | Oct | 250 | 257 | 0.89 | + | # TTTAATTG |
| Dmagna_dsx2 | MatScan | Ubx | 250 | 257 | 0.94 | + | # TTTAATTG |
| Dmagna_dsx2 | MatScan | Vsx2 | 250 | 258 | 0.93 | + | # TTTAATTGG |
| Dmagna_dsx2 | MatScan | abd-A | 251 | 257 | 0.88 | + | # TTAATTG |
| Dmagna_dsx2 | MatScan | al | 251 | 257 | 0.85 | - | # CAATTAA |
| Dmagna_dsx2 | MatScan | ap | 251 | 257 | 0.86 | + | # TTAATTG |
| Dmagna_dsx2 | MatScan | Awh | 251 | 257 | 0.87 | + | # TTAATTG |
| Dmagna_dsx2 | MatScan | B-H1 | 251 | 257 | 1 | + | # TTAATTG |
| Dmagna_dsx2 | MatScan | B-H2 | 251 | 257 | 1 | + | # TTAATTG |
| Dmagna_dsx2 | MatScan | bsh | 251 | 257 | 1 | + | # TTAATTG |
| Dmagna_dsx2 | MatScan | C15 | 251 | 257 | 0.99 | + | # TTAATTG |
| Dmagna_dsx2 | MatScan | cad | 251 | 257 | 0.9 | + | # TTAATTG |
| Dmagna_dsx2 | MatScan | CG11085 | 251 | 257 | 1 | + | # TTAATTG |
| Dmagna_dsx2 | MatScan | CG13424 | 251 | 257 | 1 | + | # TTAATTG |
| Dmagna_dsx2 | MatScan | CG15696 | 251 | 257 | 1 | + | # TTAATTG |
| Dmagna_dsx2 | MatScan | CG18599 | 251 | 257 | 0.86 | + | # TTAATTG |
| Dmagna_dsx2 | MatScan | CG32105 | 251 | 257 | 0.91 | + | # TTAATTG |
| Dmagna_dsx2 | MatScan | CG32532 | 251 | 257 | 0.99 | + | # TTAATTG |
| Dmagna_dsx2 | MatScan | CG34031 | 251 | 257 | 1 | + | # TTAATTG |
| Dmagna_dsx2 | MatScan | CG42234 | 251 | 257 | 0.87 | + | # TTAATTG |
| Dmagna_dsx2 | MatScan | CG4328 | 251 | 257 | 0.97 | + | # TTAATTG |
| Dmagna_dsx2 | MatScan | CG9876 | 251 | 257 | 0.91 | + | # TTAATTG |
| Dmagna_dsx2 | MatScan | E5 | 251 | 257 | 0.89 | + | # TTAATTG |
| Dmagna_dsx2 | MatScan | ems | 251 | 257 | 0.92 | + | # TTAATTG |
| Dmagna_dsx2 | MatScan | en | 251 | 257 | 0.98 | + | # TTAATTG |
| Dmagna_dsx2 | MatScan | eve | 251 | 257 | 0.87 | + | # TTAATTG |
| Dmagna_dsx2 | MatScan | exex | 251 | 257 | 0.85 | + | # TTAATTG |
| Dmagna_dsx2 | MatScan | exex | 251 | 257 | 0.85 | + | # TTAATTG |
| Dmagna_dsx2 | MatScan | ftz | 251 | 257 | 0.91 | + | # TTAATTG |
| Dmagna_dsx2 | MatScan | H2.0 | 251 | 257 | 0.9 | + | # TTAATTG |
| Dmagna_dsx2 | MatScan | hbn | 251 | 257 | 0.98 | + | # TTAATTG |
| Dmagna_dsx2 | MatScan | HGTX | 251 | 257 | 0.91 | + | # TTAATTG |
| Dmagna_dsx2 | MatScan | Hmx | 251 | 257 | 1 | + | # TTAATTG |
| Dmagna_dsx2 | MatScan | Lim1 | 251 | 257 | 0.87 | + | # TTAATTG |
| Dmagna_dsx2 | MatScan | Lim3 | 251 | 257 | 0.89 | + | # TTAATTG |
| Dmagna_dsx2 | MatScan | NK7.1 | 251 | 257 | 1 | + | # TTAATTG |
| Dmagna_dsx2 | MatScan | OdsH | 251 | 257 | 0.93 | + | # TTAATTG |
| Dmagna_dsx2 | MatScan | OdsH | 251 | 257 | 0.93 | + | # TTAATTG |
| Dmagna_dsx2 | MatScan | otp | 251 | 257 | 0.9 | + | # TTAATTG |
| Dmagna_dsx2 | MatScan | PHDP | 251 | 257 | 0.96 | + | # TTAATTG |
| Dmagna_dsx2 | MatScan | Pph13 | 251 | 257 | 0.91 | + | # TTAATTG |
| Dmagna_dsx2 | MatScan | repo | 251 | 257 | 0.92 | + | # TTAATTG |
| Dmagna_dsx2 | MatScan | ro | 251 | 257 | 0.86 | + | # TTAATTG |
| Dmagna_dsx2 | MatScan | Rx | 251 | 257 | 0.95 | + | # TTAATTG |
| Dmagna_dsx2 | MatScan | slou | 251 | 257 | 1 | + | # TTAATTG |
| Dmagna_dsx2 | MatScan | tup | 251 | 257 | 1 | + | # TTAATTG |
| Dmagna_dsx2 | MatScan | unc-4 | 251 | 257 | 1 | + | # TTAATTG |
| Dmagna_dsx2 | MatScan | unpg | 251 | 257 | 0.94 | + | # TTAATTG |
| Dmagna_dsx2 | MatScan | Vsx1 | 251 | 257 | 0.93 | + | # TTAATTG |
| Dmagna_dsx2 | MatScan | zen2 | 251 | 257 | 0.88 | + | # TTAATTG |
| Dmagna_dsx2 | MatScan | CG7056 | 252 | 259 | 0.87 | - | # TCCAATTA |
| Dmagna_dsx2 | MatScan | Dll | 252 | 258 | 0.93 | + | # TAATTGG |
| Dmagna_dsx2 | MatScan | Dr | 252 | 258 | 1 | - | # CCAATTA |
| Dmagna_dsx2 | MatScan | inv | 252 | 259 | 0.85 | - | # TCCAATTA |
| Dmagna_dsx2 | MatScan | lbl | 252 | 257 | 0.9 | + | # TAATTG |
| Dmagna_dsx2 | MatScan | dl_2 | 264 | 273 | 0.85 | + | # TTGGATTTCC |
| Dmagna_dsx2 | MatScan | exd | 278 | 285 | 0.86 | + | # CCTTGACA |
| Dmagna_dsx2 | MatScan | CG11617 | 280 | 286 | 0.92 | + | # TTGACAA |
| Dmagna_dsx2 | MatScan | achi | 281 | 286 | 0.93 | + | # TGACAA |
| Dmagna_dsx2 | MatScan | caup | 281 | 285 | 0.85 | + | # TGACA |
| Dmagna_dsx2 | MatScan | hth | 281 | 286 | 0.91 | + | # TGACAA |
| Dmagna_dsx2 | MatScan | vis | 281 | 286 | 0.95 | + | # TGACAA |
| Dmagna_dsx2 | MatScan | D | 283 | 293 | 0.86 | - | # TCCACTGTTGT |
| Dmagna_dsx2 | MatScan | ara | 284 | 288 | 0.89 | + | # CAACA |
| Dmagna_dsx2 | MatScan | caup | 284 | 288 | 0.87 | + | # CAACA |
| Dmagna_dsx2 | MatScan | mirr | 284 | 288 | 0.89 | + | # CAACA |
| Dmagna_dsx2 | MatScan | Deaf1 | 322 | 327 | 0.98 | - | # TTCGTC |
| Dmagna_dsx2 | MatScan | Abd-B | 333 | 339 | 0.91 | + | # TTTATCA |
| Dmagna_dsx2 | MatScan | cad | 333 | 339 | 0.87 | + | # TTTATCA |
| Dmagna_dsx2 | MatScan | CG42234 | 333 | 339 | 0.93 | + | # TTTATCA |
| Dmagna_dsx2 | MatScan | H2.0 | 333 | 339 | 0.9 | + | # TTTATCA |
| Dmagna_dsx2 | MatScan | so | 334 | 339 | 0.93 | - | # TGATAA |
| Dmagna_dsx2 | MatScan | Optix | 335 | 339 | 1 | - | # TGATA |
| Dmagna_dsx2 | MatScan | Optix | 346 | 350 | 0.87 | - | # CGATA |
| Dmagna_dsx2 | MatScan | ct | 356 | 361 | 0.86 | - | # GTGAAC |
| Dmagna_dsx2 | MatScan | onecut | 387 | 393 | 1 | + | # TTGATTT |
| Dmagna_dsx2 | MatScan | exd | 391 | 398 | 1 | + | # TTTTGACA |
| Dmagna_dsx2 | MatScan | CG11617 | 393 | 399 | 0.93 | + | # TTGACAT |
| Dmagna_dsx2 | MatScan | achi | 394 | 399 | 0.92 | + | # TGACAT |
| Dmagna_dsx2 | MatScan | caup | 394 | 398 | 0.85 | + | # TGACA |
| Dmagna_dsx2 | MatScan | hth | 394 | 399 | 0.96 | + | # TGACAT |
| Dmagna_dsx2 | MatScan | vis | 394 | 399 | 0.95 | + | # TGACAT |
| Dmagna_dsx2 | MatScan | achi | 399 | 404 | 0.9 | + | # TGACAC |
| Dmagna_dsx2 | MatScan | caup | 399 | 403 | 0.85 | + | # TGACA |
| Dmagna_dsx2 | MatScan | hth | 399 | 404 | 0.91 | + | # TGACAC |
| Dmagna_dsx2 | MatScan | Six4 | 399 | 404 | 0.98 | + | # TGACAC |
| Dmagna_dsx2 | MatScan | vis | 399 | 404 | 0.97 | + | # TGACAC |
| Dmagna_dsx2 | MatScan | Abd-B | 404 | 410 | 0.87 | - | # TTTATAG |
| Dmagna_dsx2 | MatScan | cad | 404 | 410 | 0.89 | - | # TTTATAG |
| Dmagna_dsx2 | MatScan | TATA | 404 | 418 | 0.91 | + | # CTATAAAAACTGCTA |
| Dmagna_dsx2 | MatScan | lbe | 407 | 412 | 0.85 | + | # TAAAAA |
| Dmagna_dsx2 | MatScan | Dfd | 411 | 426 | 0.87 | - | # TACGAGATTAGCAGTT |
| Dmagna_dsx2 | MatScan | Bcd | 416 | 423 | 0.87 | - | # GAGATTAG |
| Dmagna_dsx2 | MatScan | Gsc | 417 | 422 | 0.94 | + | # TAATCT |
| Dmagna_dsx2 | MatScan | oc | 417 | 422 | 0.85 | + | # TAATCT |
| Dmagna_dsx2 | MatScan | Deaf1 | 463 | 468 | 0.98 | + | # TTCGTC |
| Dmagna_dsx2 | MatScan | hb | 470 | 479 | 0.95 | - | # GAAAAAAAAA |
| Dmagna_dsx2 | MatScan | hb | 471 | 480 | 0.88 | - | # CGAAAAAAAA |
| Dmagna_dsx2 | MatScan | hb | 472 | 481 | 0.92 | - | # GCGAAAAAAA |
| Dmagna_dsx2 | MatScan | Deaf1 | 477 | 482 | 0.87 | + | # TTCGCC |
| Dmagna_dsx2 | MatScan | Deaf1 | 480 | 485 | 0.89 | - | # CTCGGC |
| Dmagna_dsx2 | MatScan | pan | 493 | 500 | 0.95 | - | # GTTTGATC |
| Dmagna_dsx2 | MatScan | B-H1 | 503 | 509 | 0.89 | - | # CTAAATG |
| Dmagna_dsx2 | MatScan | Trl | 513 | 522 | 0.89 | - | # CTTCTCTCCC |
| Dmagna_dsx2 | MatScan | CG13424 | 531 | 537 | 0.88 | + | # CTAATAG |
| Dmagna_dsx2 | MatScan | Dll | 532 | 538 | 0.88 | + | # TAATAGC |
| Dmagna_dsx2 | MatScan | ct | 550 | 555 | 0.86 | + | # GTGAAC |
| Dmagna_dsx2 | MatScan | ara | 552 | 556 | 0.93 | + | # GAACA |
| Dmagna_dsx2 | MatScan | caup | 552 | 556 | 0.88 | + | # GAACA |
| Dmagna_dsx2 | MatScan | mirr | 552 | 556 | 0.89 | + | # GAACA |
| Dmagna_dsx2 | MatScan | pan | 552 | 559 | 0.85 | - | # ATTTGTTC |
| Dmagna_dsx2 | MatScan | br_Z2 | 556 | 563 | 0.86 | - | # CCCTATTT |
| Dmagna_dsx2 | MatScan | Deaf1 | 562 | 567 | 0.87 | - | # TTCGCC |
| Dmagna_dsx2 | MatScan | Deaf1 | 590 | 595 | 0.98 | + | # TTCGTC |
| Dmagna_dsx2 | MatScan | onecut | 599 | 605 | 0.87 | + | # CTGATTG |
| Dmagna_dsx2 | MatScan | slbo | 604 | 611 | 0.85 | - | # ATCGCACA |
| Dmagna_dsx2 | MatScan | Trl | 627 | 636 | 0.85 | - | # TCTCTCTTTC |
| Dmagna_dsx2 | MatScan | Trl | 629 | 638 | 0.95 | - | # TTTCTCTCTT |
| Dmagna_dsx2 | MatScan | ovo | 634 | 642 | 0.93 | + | # AGAAACAGT |
| Dmagna_dsx2 | MatScan | prd | 634 | 642 | 0.93 | + | # AGAAACAGT |
| Dmagna_dsx2 | MatScan | ara | 636 | 640 | 0.99 | + | # AAACA |
| Dmagna_dsx2 | MatScan | caup | 636 | 640 | 0.9 | + | # AAACA |
| Dmagna_dsx2 | MatScan | mirr | 636 | 640 | 1 | + | # AAACA |
| Dmagna_dsx2 | MatScan | Deaf1 | 652 | 657 | 0.96 | + | # TTCGTT |
| Dmagna_dsx2 | MatScan | pan | 666 | 673 | 0.87 | + | # TTTTGAAT |
| Dmagna_dsx2 | MatScan | CG7056 | 668 | 675 | 0.89 | + | # TTGAATAA |
| Dmagna_dsx2 | MatScan | vvl | 669 | 674 | 0.98 | - | # TATTCA |
| Dmagna_dsx2 | MatScan | Abd-B | 670 | 676 | 0.86 | - | # TTTATTC |
| Dmagna_dsx2 | MatScan | cad | 670 | 676 | 0.91 | - | # TTTATTC |
| Dmagna_dsx2 | MatScan | CG42234 | 670 | 676 | 0.85 | - | # TTTATTC |
| Dmagna_dsx2 | MatScan | CG4328 | 670 | 676 | 0.9 | - | # TTTATTC |
| Dmagna_dsx2 | MatScan | CG15696 | 673 | 679 | 0.86 | + | # TAAATTG |
| Dmagna_dsx2 | MatScan | CG4328 | 673 | 679 | 0.87 | + | # TAAATTG |
| Dmagna_dsx2 | MatScan | ovo | 680 | 688 | 0.9 | + | # TCTAACAGT |
| Dmagna_dsx2 | MatScan | prd | 680 | 688 | 0.9 | + | # TCTAACAGT |
| Dmagna_dsx2 | MatScan | ara | 682 | 686 | 1 | + | # TAACA |
| Dmagna_dsx2 | MatScan | caup | 682 | 686 | 1 | + | # TAACA |
| Dmagna_dsx2 | MatScan | mirr | 682 | 686 | 0.99 | + | # TAACA |
| Dmagna_dsx2 | MatScan | hb | 690 | 699 | 0.9 | - | # GATTAAAAAA |
| Dmagna_dsx2 | MatScan | C15 | 691 | 697 | 0.85 | - | # TTAAAAA |
| Dmagna_dsx2 | MatScan | lbe | 691 | 696 | 0.85 | - | # TAAAAA |
| Dmagna_dsx2 | MatScan | dri | 692 | 701 | 0.93 | - | # TTGATTAAAA |
| Dmagna_dsx2 | MatScan | CG7056 | 693 | 700 | 0.88 | + | # TTTAATCA |
| Dmagna_dsx2 | MatScan | Oct | 693 | 700 | 0.94 | + | # TTTAATCA |
| Dmagna_dsx2 | MatScan | Ubx | 693 | 700 | 0.89 | + | # TTTAATCA |
| Dmagna_dsx2 | MatScan | abd-A | 694 | 700 | 0.89 | + | # TTAATCA |
| Dmagna_dsx2 | MatScan | al | 694 | 700 | 0.85 | - | # TGATTAA |
| Dmagna_dsx2 | MatScan | Antp | 694 | 700 | 0.91 | + | # TTAATCA |
| Dmagna_dsx2 | MatScan | ap | 694 | 700 | 0.86 | + | # TTAATCA |
| Dmagna_dsx2 | MatScan | Awh | 694 | 700 | 0.87 | + | # TTAATCA |
| Dmagna_dsx2 | MatScan | bsh | 694 | 700 | 0.93 | + | # TTAATCA |
| Dmagna_dsx2 | MatScan | btn | 694 | 700 | 0.89 | + | # TTAATCA |
| Dmagna_dsx2 | MatScan | C15 | 694 | 700 | 0.93 | + | # TTAATCA |
| Dmagna_dsx2 | MatScan | CG18599 | 694 | 700 | 0.9 | + | # TTAATCA |
| Dmagna_dsx2 | MatScan | CG42234 | 694 | 700 | 0.91 | + | # TTAATCA |
| Dmagna_dsx2 | MatScan | Dfd | 694 | 700 | 0.87 | + | # TTAATCA |
| Dmagna_dsx2 | MatScan | E5 | 694 | 700 | 0.91 | + | # TTAATCA |
| Dmagna_dsx2 | MatScan | ems | 694 | 700 | 0.92 | + | # TTAATCA |
| Dmagna_dsx2 | MatScan | eve | 694 | 700 | 0.92 | + | # TTAATCA |
| Dmagna_dsx2 | MatScan | ftz | 694 | 700 | 0.92 | + | # TTAATCA |
| Dmagna_dsx2 | MatScan | H2.0 | 694 | 700 | 0.91 | + | # TTAATCA |
| Dmagna_dsx2 | MatScan | HGTX | 694 | 700 | 0.89 | + | # TTAATCA |
| Dmagna_dsx2 | MatScan | ind | 694 | 700 | 0.87 | + | # TTAATCA |
| Dmagna_dsx2 | MatScan | lab | 694 | 700 | 0.9 | + | # TTAATCA |
| Dmagna_dsx2 | MatScan | Lim1 | 694 | 700 | 0.85 | + | # TTAATCA |
| Dmagna_dsx2 | MatScan | Lim3 | 694 | 700 | 0.89 | + | # TTAATCA |
| Dmagna_dsx2 | MatScan | otp | 694 | 700 | 0.88 | + | # TTAATCA |
| Dmagna_dsx2 | MatScan | pb | 694 | 700 | 0.91 | + | # TTAATCA |
| Dmagna_dsx2 | MatScan | Ptx1 | 694 | 700 | 0.86 | + | # TTAATCA |
| Dmagna_dsx2 | MatScan | Scr | 694 | 700 | 0.88 | + | # TTAATCA |
| Dmagna_dsx2 | MatScan | slou | 694 | 700 | 0.89 | + | # TTAATCA |
| Dmagna_dsx2 | MatScan | Vsx1 | 694 | 700 | 0.87 | + | # TTAATCA |
| Dmagna_dsx2 | MatScan | zen2 | 694 | 700 | 0.89 | + | # TTAATCA |
| Dmagna_dsx2 | MatScan | abd-A | 695 | 701 | 0.85 | - | # TTGATTA |
| Dmagna_dsx2 | MatScan | al | 695 | 701 | 0.85 | + | # TAATCAA |
| Dmagna_dsx2 | MatScan | Awh | 695 | 701 | 0.89 | - | # TTGATTA |
| Dmagna_dsx2 | MatScan | cad | 695 | 701 | 0.87 | - | # TTGATTA |
| Dmagna_dsx2 | MatScan | CG15696 | 695 | 701 | 0.92 | - | # TTGATTA |
| Dmagna_dsx2 | MatScan | CG32105 | 695 | 701 | 0.85 | - | # TTGATTA |
| Dmagna_dsx2 | MatScan | CG42234 | 695 | 701 | 0.93 | - | # TTGATTA |
| Dmagna_dsx2 | MatScan | CG4328 | 695 | 701 | 0.89 | - | # TTGATTA |
| Dmagna_dsx2 | MatScan | Gsc | 695 | 700 | 0.89 | + | # TAATCA |
| Dmagna_dsx2 | MatScan | H2.0 | 695 | 701 | 0.91 | - | # TTGATTA |
| Dmagna_dsx2 | MatScan | lbe | 695 | 700 | 0.94 | + | # TAATCA |
| Dmagna_dsx2 | MatScan | lbl | 695 | 700 | 0.92 | + | # TAATCA |
| Dmagna_dsx2 | MatScan | Lim1 | 695 | 701 | 0.85 | - | # TTGATTA |
| Dmagna_dsx2 | MatScan | Lim3 | 695 | 701 | 0.88 | - | # TTGATTA |
| Dmagna_dsx2 | MatScan | onecut | 695 | 701 | 0.99 | - | # TTGATTA |
| Dmagna_dsx2 | MatScan | vvl | 695 | 700 | 0.88 | + | # TAATCA |
| Dmagna_dsx2 | MatScan | ara | 699 | 703 | 0.89 | + | # CAACA |
| Dmagna_dsx2 | MatScan | caup | 699 | 703 | 0.87 | + | # CAACA |
| Dmagna_dsx2 | MatScan | mirr | 699 | 703 | 0.89 | + | # CAACA |
| Dmagna_dsx2 | MatScan | ara | 712 | 716 | 0.99 | - | # AAACA |
| Dmagna_dsx2 | MatScan | caup | 712 | 716 | 0.9 | - | # AAACA |
| Dmagna_dsx2 | MatScan | mirr | 712 | 716 | 1 | - | # AAACA |
| Dmagna_dsx2 | MatScan | D | 717 | 727 | 0.88 | + | # TCCATTGATCT |
| Dmagna_dsx2 | MatScan | dTCF | 718 | 728 | 0.91 | + | # CCATTGATCTT |
| Dmagna_dsx2 | MatScan | pan | 719 | 726 | 0.87 | + | # CATTGATC |
| Dmagna_dsx2 | MatScan | ct | 735 | 740 | 0.92 | - | # CTGAAC |
| Dmagna_dsx2 | MatScan | hb | 741 | 750 | 0.88 | - | # GCCTAAAAAG |
| Dmagna_dsx2 | MatScan | lbe | 742 | 747 | 0.85 | - | # TAAAAA |
| Dmagna_dsx2 | MatScan | achi | 766 | 771 | 0.92 | - | # TGACAT |
| Dmagna_dsx2 | MatScan | CG11617 | 766 | 772 | 0.93 | - | # TTGACAT |
| Dmagna_dsx2 | MatScan | hth | 766 | 771 | 0.96 | - | # TGACAT |
| Dmagna_dsx2 | MatScan | vis | 766 | 771 | 0.95 | - | # TGACAT |
| Dmagna_dsx2 | MatScan | caup | 767 | 771 | 0.85 | - | # TGACA |
| Dmagna_dsx2 | MatScan | exd | 767 | 774 | 0.88 | - | # TATTGACA |
| Dmagna_dsx2 | MatScan | cad | 770 | 776 | 0.87 | - | # CTTATTG |
| Dmagna_dsx2 | MatScan | CG4328 | 770 | 776 | 0.93 | - | # CTTATTG |
| Dmagna_dsx2 | MatScan | sd | 780 | 791 | 0.86 | - | # AACATTTCTGAA |
| Dmagna_dsx2 | MatScan | Dfd | 785 | 800 | 0.85 | - | # CCAACTATTAACATTT |
| Dmagna_dsx2 | MatScan | CG11617 | 787 | 793 | 1 | - | # TTAACAT |
| Dmagna_dsx2 | MatScan | ara | 788 | 792 | 1 | - | # TAACA |
| Dmagna_dsx2 | MatScan | caup | 788 | 792 | 1 | - | # TAACA |
| Dmagna_dsx2 | MatScan | mirr | 788 | 792 | 0.99 | - | # TAACA |
| Dmagna_dsx2 | MatScan | B-H1 | 790 | 796 | 0.9 | + | # TTAATAG |
| Dmagna_dsx2 | MatScan | B-H2 | 790 | 796 | 0.9 | + | # TTAATAG |
| Dmagna_dsx2 | MatScan | bsh | 790 | 796 | 0.92 | + | # TTAATAG |
| Dmagna_dsx2 | MatScan | C15 | 790 | 796 | 0.89 | + | # TTAATAG |
| Dmagna_dsx2 | MatScan | CG11085 | 790 | 796 | 0.93 | + | # TTAATAG |
| Dmagna_dsx2 | MatScan | CG13424 | 790 | 796 | 0.91 | + | # TTAATAG |
| Dmagna_dsx2 | MatScan | CG34031 | 790 | 796 | 0.94 | + | # TTAATAG |
| Dmagna_dsx2 | MatScan | H2.0 | 790 | 796 | 0.85 | + | # TTAATAG |
| Dmagna_dsx2 | MatScan | Hmx | 790 | 796 | 0.87 | + | # TTAATAG |
| Dmagna_dsx2 | MatScan | NK7.1 | 790 | 796 | 0.92 | + | # TTAATAG |
| Dmagna_dsx2 | MatScan | slou | 790 | 796 | 0.9 | + | # TTAATAG |
| Dmagna_dsx2 | MatScan | tup | 790 | 796 | 0.92 | + | # TTAATAG |
| Dmagna_dsx2 | MatScan | unc-4 | 790 | 796 | 0.85 | + | # TTAATAG |
| Dmagna_dsx2 | MatScan | vvl | 790 | 795 | 0.88 | - | # TATTAA |
| Dmagna_dsx2 | MatScan | CG4328 | 791 | 797 | 0.85 | - | # ACTATTA |
| Dmagna_dsx2 | MatScan | Deaf1 | 805 | 810 | 1 | - | # TTCGTG |
| Dmagna_dsx2 | MatScan | dl_2 | 822 | 831 | 0.88 | + | # GTGGGTTTCC |
| Dmagna_dsx2 | MatScan | dl | 823 | 833 | 0.87 | - | # TGGGAAACCCA |
| Dmagna_dsx2 | MatScan | br_Z2 | 838 | 845 | 0.89 | + | # TACTACTT |
| Dmagna_dsx2 | MatScan | ct | 844 | 849 | 0.89 | + | # TTCAAC |
| Dmagna_dsx2 | MatScan | Deaf1 | 848 | 853 | 0.96 | - | # TTCGGT |
| Dmagna_dsx2 | MatScan | Zeste | 851 | 866 | 0.88 | + | # GAATCTGAGTGGCTTG |
| Dmagna_dsx2 | MatScan | z | 855 | 864 | 0.86 | + | # CTGAGTGGCT |
| Dmagna_dsx2 | MatScan | ara | 913 | 917 | 0.99 | + | # AAACA |
| Dmagna_dsx2 | MatScan | caup | 913 | 917 | 0.9 | + | # AAACA |
| Dmagna_dsx2 | MatScan | mirr | 913 | 917 | 1 | + | # AAACA |
| Dmagna_dsx2 | MatScan | Bcd | 926 | 933 | 0.95 | - | # GGGATTAG |
| Dmagna_dsx2 | MatScan | Ptx1 | 926 | 932 | 0.93 | + | # CTAATCC |
| Dmagna_dsx2 | MatScan | bcd | 927 | 932 | 1 | + | # TAATCC |
| Dmagna_dsx2 | MatScan | Gsc | 927 | 932 | 1 | + | # TAATCC |
| Dmagna_dsx2 | MatScan | oc | 927 | 932 | 1 | + | # TAATCC |
| Dmagna_dsx2 | MatScan | Deaf1 | 942 | 947 | 0.96 | + | # TTCGTT |
| Dmagna_dsx2 | MatScan | Lag1 | 957 | 963 | 0.88 | - | # CCACCAC |
| Dmagna_dsx2 | MatScan | Six4 | 981 | 986 | 0.95 | - | # TGAGAC |
| Dmagna_dsx2 | MatScan | ara | 992 | 996 | 0.89 | + | # CAACA |
| Dmagna_dsx2 | MatScan | caup | 992 | 996 | 0.87 | + | # CAACA |
| Dmagna_dsx2 | MatScan | mirr | 992 | 996 | 0.89 | + | # CAACA |
