# Supplementary material for: Molecular cloning of doublesex genes of four cladocera (water flea) species
Source: BMC Genomics. 2013 Apr 10;14:239. doi: 10.1186/1471-2164-14-239 (PMC3637828; doi:10.1186/1471-2164-14-239)
Supplement: Additional file 21 — DappuDsx2 TF-map. [file 1471-2164-14-239-S21.doc]

Supplemental Material 21. *DappuDsx2* TF-map

| Column Descriptions | Promoter region ID - Species, dsx paralog number, and dsx transcript identifier  Name of program that generated results  Name of transcription factor identified  Start of transcription factor binding site (TFBS)  End of transcription factor binding site (TFBS)  Match score between known TFBS (from TFBS database) and identified Daphnia dsx promoter sequence motif  Strand on which TFBS was identified in sequence  Reading frame for CDS feature types (not used)  Sequence of transcription factor binding motif (from TFBS database) | | | | | | |
| --- | --- | --- | --- | --- | --- | --- | --- |
| Sequence ID |
| Source |
| Type (TF) |
| Start |
| End |
| Score |
| Strand |
| Phase |
| TF Binding Motif |
|  |  |  |  |  |  |  |  |
| **Sequence ID** | **Source** | **Type (TF)** | **Start** | **End** | **Score** | **Strand** | **TF Binding Motif** |
| Dpulex_dsx2 | MatScan | achi | 3 | 8 | 0.93 | - | # TGACAA |
| Dpulex_dsx2 | MatScan | hth | 3 | 8 | 0.91 | - | # TGACAA |
| Dpulex_dsx2 | MatScan | vis | 3 | 8 | 0.95 | - | # TGACAA |
| Dpulex_dsx2 | MatScan | caup | 4 | 8 | 0.85 | - | # TGACA |
| Dpulex_dsx2 | MatScan | achi | 22 | 27 | 0.93 | - | # TGACAA |
| Dpulex_dsx2 | MatScan | CG11617 | 22 | 28 | 0.92 | - | # TTGACAA |
| Dpulex_dsx2 | MatScan | hth | 22 | 27 | 0.91 | - | # TGACAA |
| Dpulex_dsx2 | MatScan | vis | 22 | 27 | 0.95 | - | # TGACAA |
| Dpulex_dsx2 | MatScan | caup | 23 | 27 | 0.85 | - | # TGACA |
| Dpulex_dsx2 | MatScan | exd | 23 | 30 | 0.86 | - | # ACTTGACA |
| Dpulex_dsx2 | MatScan | vnd | 23 | 31 | 0.87 | + | # TGTCAAGTC |
| Dpulex_dsx2 | MatScan | Six4 | 40 | 45 | 0.95 | - | # TGAGAC |
| Dpulex_dsx2 | MatScan | ems | 43 | 49 | 0.85 | - | # CAAATGA |
| Dpulex_dsx2 | MatScan | Deaf1 | 60 | 65 | 0.93 | - | # TTCGTA |
| Dpulex_dsx2 | MatScan | ara | 72 | 76 | 0.91 | - | # TTACA |
| Dpulex_dsx2 | MatScan | caup | 72 | 76 | 0.87 | - | # TTACA |
| Dpulex_dsx2 | MatScan | mirr | 72 | 76 | 0.88 | - | # TTACA |
| Dpulex_dsx2 | MatScan | exd | 88 | 95 | 0.95 | + | # TTTTGACG |
| Dpulex_dsx2 | MatScan | B-H1 | 102 | 108 | 0.85 | - | # TTAAAGG |
| Dpulex_dsx2 | MatScan | C15 | 102 | 108 | 0.9 | - | # TTAAAGG |
| Dpulex_dsx2 | MatScan | sd | 102 | 113 | 0.85 | - | # AACATTTAAAGG |
| Dpulex_dsx2 | MatScan | B-H1 | 105 | 111 | 0.96 | + | # TTAAATG |
| Dpulex_dsx2 | MatScan | B-H2 | 105 | 111 | 0.91 | + | # TTAAATG |
| Dpulex_dsx2 | MatScan | C15 | 105 | 111 | 0.94 | + | # TTAAATG |
| Dpulex_dsx2 | MatScan | CG11085 | 105 | 111 | 0.86 | + | # TTAAATG |
| Dpulex_dsx2 | MatScan | CG34031 | 105 | 111 | 0.87 | + | # TTAAATG |
| Dpulex_dsx2 | MatScan | Hmx | 105 | 111 | 0.86 | + | # TTAAATG |
| Dpulex_dsx2 | MatScan | NK7.1 | 105 | 111 | 0.89 | + | # TTAAATG |
| Dpulex_dsx2 | MatScan | ara | 110 | 114 | 0.89 | - | # CAACA |
| Dpulex_dsx2 | MatScan | caup | 110 | 114 | 0.87 | - | # CAACA |
| Dpulex_dsx2 | MatScan | mirr | 110 | 114 | 0.89 | - | # CAACA |
| Dpulex_dsx2 | MatScan | ct | 111 | 116 | 0.89 | - | # TTCAAC |
| Dpulex_dsx2 | MatScan | hb | 120 | 129 | 0.87 | + | # GGGAAAAAAA |
| Dpulex_dsx2 | MatScan | hb | 121 | 130 | 0.86 | + | # GGAAAAAAAT |
| Dpulex_dsx2 | MatScan | ara | 130 | 134 | 0.99 | - | # AAACA |
| Dpulex_dsx2 | MatScan | caup | 130 | 134 | 0.9 | - | # AAACA |
| Dpulex_dsx2 | MatScan | mirr | 130 | 134 | 1 | - | # AAACA |
| Dpulex_dsx2 | MatScan | hb | 140 | 149 | 0.95 | - | # GAAAAAAAAA |
| Dpulex_dsx2 | MatScan | hb | 142 | 151 | 0.9 | - | # GAGAAAAAAA |
| Dpulex_dsx2 | MatScan | hb | 143 | 152 | 0.88 | - | # GGAGAAAAAA |
| Dpulex_dsx2 | MatScan | lbe | 154 | 159 | 0.86 | - | # TAAGAA |
| Dpulex_dsx2 | MatScan | C15 | 166 | 172 | 0.85 | - | # TTAAATT |
| Dpulex_dsx2 | MatScan | C15 | 169 | 175 | 0.85 | + | # TTAAATT |
| Dpulex_dsx2 | MatScan | sd | 175 | 186 | 0.87 | - | # TTCATTCCTCAA |
| Dpulex_dsx2 | MatScan | C15 | 187 | 193 | 0.85 | - | # TTAAATC |
| Dpulex_dsx2 | MatScan | dri | 188 | 197 | 0.87 | - | # AAAATTAAAT |
| Dpulex_dsx2 | MatScan | CG7056 | 189 | 196 | 0.85 | + | # TTTAATTT |
| Dpulex_dsx2 | MatScan | inv | 189 | 196 | 0.87 | + | # TTTAATTT |
| Dpulex_dsx2 | MatScan | Oct | 189 | 196 | 0.92 | + | # TTTAATTT |
| Dpulex_dsx2 | MatScan | Ubx | 189 | 196 | 0.89 | + | # TTTAATTT |
| Dpulex_dsx2 | MatScan | abd-A | 190 | 196 | 0.86 | + | # TTAATTT |
| Dpulex_dsx2 | MatScan | al | 190 | 196 | 0.85 | - | # AAATTAA |
| Dpulex_dsx2 | MatScan | Awh | 190 | 196 | 0.87 | + | # TTAATTT |
| Dpulex_dsx2 | MatScan | bsh | 190 | 196 | 0.9 | + | # TTAATTT |
| Dpulex_dsx2 | MatScan | C15 | 190 | 196 | 0.9 | + | # TTAATTT |
| Dpulex_dsx2 | MatScan | CG13424 | 190 | 196 | 0.89 | + | # TTAATTT |
| Dpulex_dsx2 | MatScan | CG15696 | 190 | 196 | 0.91 | + | # TTAATTT |
| Dpulex_dsx2 | MatScan | CG32105 | 190 | 196 | 0.87 | + | # TTAATTT |
| Dpulex_dsx2 | MatScan | CG32532 | 190 | 196 | 0.92 | + | # TTAATTT |
| Dpulex_dsx2 | MatScan | CG34031 | 190 | 196 | 0.86 | + | # TTAATTT |
| Dpulex_dsx2 | MatScan | CG4328 | 190 | 196 | 0.87 | + | # TTAATTT |
| Dpulex_dsx2 | MatScan | CG9876 | 190 | 196 | 0.86 | + | # TTAATTT |
| Dpulex_dsx2 | MatScan | E5 | 190 | 196 | 0.85 | + | # TTAATTT |
| Dpulex_dsx2 | MatScan | ems | 190 | 196 | 0.86 | + | # TTAATTT |
| Dpulex_dsx2 | MatScan | en | 190 | 196 | 0.9 | + | # TTAATTT |
| Dpulex_dsx2 | MatScan | ftz | 190 | 196 | 0.87 | + | # TTAATTT |
| Dpulex_dsx2 | MatScan | H2.0 | 190 | 196 | 0.86 | + | # TTAATTT |
| Dpulex_dsx2 | MatScan | hbn | 190 | 196 | 0.92 | + | # TTAATTT |
| Dpulex_dsx2 | MatScan | HGTX | 190 | 196 | 0.87 | + | # TTAATTT |
| Dpulex_dsx2 | MatScan | Hmx | 190 | 196 | 0.88 | + | # TTAATTT |
| Dpulex_dsx2 | MatScan | Lim1 | 190 | 196 | 0.86 | + | # TTAATTT |
| Dpulex_dsx2 | MatScan | Lim3 | 190 | 196 | 0.85 | + | # TTAATTT |
| Dpulex_dsx2 | MatScan | NK7.1 | 190 | 196 | 0.89 | + | # TTAATTT |
| Dpulex_dsx2 | MatScan | OdsH | 190 | 196 | 0.88 | + | # TTAATTT |
| Dpulex_dsx2 | MatScan | OdsH | 190 | 196 | 0.88 | + | # TTAATTT |
| Dpulex_dsx2 | MatScan | otp | 190 | 196 | 0.89 | + | # TTAATTT |
| Dpulex_dsx2 | MatScan | PHDP | 190 | 196 | 0.96 | + | # TTAATTT |
| Dpulex_dsx2 | MatScan | Pph13 | 190 | 196 | 0.87 | + | # TTAATTT |
| Dpulex_dsx2 | MatScan | repo | 190 | 196 | 0.88 | + | # TTAATTT |
| Dpulex_dsx2 | MatScan | Rx | 190 | 196 | 0.88 | + | # TTAATTT |
| Dpulex_dsx2 | MatScan | slou | 190 | 196 | 0.91 | + | # TTAATTT |
| Dpulex_dsx2 | MatScan | tup | 190 | 196 | 0.87 | + | # TTAATTT |
| Dpulex_dsx2 | MatScan | unc-4 | 190 | 196 | 0.89 | + | # TTAATTT |
| Dpulex_dsx2 | MatScan | unpg | 190 | 196 | 0.89 | + | # TTAATTT |
| Dpulex_dsx2 | MatScan | Vsx1 | 190 | 196 | 0.89 | + | # TTAATTT |
| Dpulex_dsx2 | MatScan | zen2 | 190 | 196 | 0.85 | + | # TTAATTT |
| Dpulex_dsx2 | MatScan | CG4328 | 191 | 197 | 0.87 | - | # AAAATTA |
| Dpulex_dsx2 | MatScan | Dll | 191 | 197 | 0.91 | + | # TAATTTT |
| Dpulex_dsx2 | MatScan | lbl | 191 | 196 | 0.85 | + | # TAATTT |
| Dpulex_dsx2 | MatScan | Lag1 | 196 | 202 | 0.95 | - | # CTACTAA |
| Dpulex_dsx2 | MatScan | ara | 219 | 223 | 0.89 | + | # CAACA |
| Dpulex_dsx2 | MatScan | caup | 219 | 223 | 0.87 | + | # CAACA |
| Dpulex_dsx2 | MatScan | mirr | 219 | 223 | 0.89 | + | # CAACA |
| Dpulex_dsx2 | MatScan | eve | 247 | 253 | 0.85 | + | # CTAAGGA |
| Dpulex_dsx2 | MatScan | lbe | 248 | 253 | 0.85 | + | # TAAGGA |
| Dpulex_dsx2 | MatScan | sd | 256 | 267 | 0.93 | + | # GACATTTATCAA |
| Dpulex_dsx2 | MatScan | B-H1 | 258 | 264 | 0.89 | - | # ATAAATG |
| Dpulex_dsx2 | MatScan | B-H2 | 258 | 264 | 0.86 | - | # ATAAATG |
| Dpulex_dsx2 | MatScan | Abd-B | 260 | 266 | 0.91 | + | # TTTATCA |
| Dpulex_dsx2 | MatScan | cad | 260 | 266 | 0.87 | + | # TTTATCA |
| Dpulex_dsx2 | MatScan | CG42234 | 260 | 266 | 0.93 | + | # TTTATCA |
| Dpulex_dsx2 | MatScan | H2.0 | 260 | 266 | 0.9 | + | # TTTATCA |
| Dpulex_dsx2 | MatScan | H2.0 | 261 | 267 | 0.86 | - | # TTGATAA |
| Dpulex_dsx2 | MatScan | so | 261 | 266 | 0.93 | - | # TGATAA |
| Dpulex_dsx2 | MatScan | exd | 262 | 269 | 0.93 | - | # TTTTGATA |
| Dpulex_dsx2 | MatScan | Optix | 262 | 266 | 1 | - | # TGATA |
| Dpulex_dsx2 | MatScan | pan | 262 | 269 | 0.96 | - | # TTTTGATA |
| Dpulex_dsx2 | MatScan | ara | 274 | 278 | 0.91 | + | # ATACA |
| Dpulex_dsx2 | MatScan | mirr | 274 | 278 | 0.89 | + | # ATACA |
| Dpulex_dsx2 | MatScan | Optix | 279 | 283 | 0.87 | - | # CGATA |
| Dpulex_dsx2 | MatScan | ct | 289 | 294 | 0.86 | - | # GTGAAC |
| Dpulex_dsx2 | MatScan | onecut | 318 | 324 | 1 | + | # TTGATTT |
| Dpulex_dsx2 | MatScan | exd | 322 | 329 | 1 | + | # TTTTGACA |
| Dpulex_dsx2 | MatScan | CG11617 | 324 | 330 | 0.93 | + | # TTGACAT |
| Dpulex_dsx2 | MatScan | achi | 325 | 330 | 0.92 | + | # TGACAT |
| Dpulex_dsx2 | MatScan | caup | 325 | 329 | 0.85 | + | # TGACA |
| Dpulex_dsx2 | MatScan | hth | 325 | 330 | 0.96 | + | # TGACAT |
| Dpulex_dsx2 | MatScan | vis | 325 | 330 | 0.95 | + | # TGACAT |
| Dpulex_dsx2 | MatScan | achi | 330 | 335 | 0.9 | + | # TGACAC |
| Dpulex_dsx2 | MatScan | caup | 330 | 334 | 0.85 | + | # TGACA |
| Dpulex_dsx2 | MatScan | hth | 330 | 335 | 0.91 | + | # TGACAC |
| Dpulex_dsx2 | MatScan | Six4 | 330 | 335 | 0.98 | + | # TGACAC |
| Dpulex_dsx2 | MatScan | vis | 330 | 335 | 0.97 | + | # TGACAC |
| Dpulex_dsx2 | MatScan | vvl | 336 | 341 | 0.9 | + | # TATGAA |
| Dpulex_dsx2 | MatScan | ovo | 338 | 346 | 0.85 | + | # TGAAACTGC |
| Dpulex_dsx2 | MatScan | prd | 338 | 346 | 0.85 | + | # TGAAACTGC |
| Dpulex_dsx2 | MatScan | Six4 | 338 | 343 | 0.92 | + | # TGAAAC |
| Dpulex_dsx2 | MatScan | ara | 349 | 353 | 0.91 | - | # TTACA |
| Dpulex_dsx2 | MatScan | caup | 349 | 353 | 0.87 | - | # TTACA |
| Dpulex_dsx2 | MatScan | mirr | 349 | 353 | 0.88 | - | # TTACA |
| Dpulex_dsx2 | MatScan | bap | 350 | 356 | 0.86 | + | # GTAAGTG |
| Dpulex_dsx2 | MatScan | ara | 355 | 359 | 0.91 | - | # TTACA |
| Dpulex_dsx2 | MatScan | caup | 355 | 359 | 0.87 | - | # TTACA |
| Dpulex_dsx2 | MatScan | mirr | 355 | 359 | 0.88 | - | # TTACA |
| Dpulex_dsx2 | MatScan | Gsc | 357 | 362 | 0.94 | + | # TAATCT |
| Dpulex_dsx2 | MatScan | oc | 357 | 362 | 0.85 | + | # TAATCT |
| Dpulex_dsx2 | MatScan | achi | 375 | 380 | 1 | + | # TGACAG |
| Dpulex_dsx2 | MatScan | caup | 375 | 379 | 0.85 | + | # TGACA |
| Dpulex_dsx2 | MatScan | hth | 375 | 380 | 1 | + | # TGACAG |
| Dpulex_dsx2 | MatScan | vis | 375 | 380 | 1 | + | # TGACAG |
| Dpulex_dsx2 | MatScan | brk | 379 | 386 | 0.89 | - | # CGGGCGCT |
| Dpulex_dsx2 | MatScan | B-H1 | 416 | 422 | 0.86 | - | # TTAAAAG |
| Dpulex_dsx2 | MatScan | oc | 420 | 425 | 0.86 | + | # TAAGCC |
| Dpulex_dsx2 | MatScan | Deaf1 | 429 | 434 | 0.96 | + | # TTCGTT |
| Dpulex_dsx2 | MatScan | eve | 430 | 436 | 0.86 | - | # CTAACGA |
| Dpulex_dsx2 | MatScan | lbe | 430 | 435 | 0.93 | - | # TAACGA |
| Dpulex_dsx2 | MatScan | BR-C | 437 | 452 | 0.86 | + | # GTTTTTTTTATTTATT |
| Dpulex_dsx2 | MatScan | BR-C | 438 | 450 | 0.92 | - | # TAAATAAAAAAAA |
| Dpulex_dsx2 | MatScan | Croc | 440 | 455 | 0.87 | - | # ATAAATAAATAAAAAA |
| Dpulex_dsx2 | MatScan | hb | 440 | 449 | 0.92 | - | # AAATAAAAAA |
| Dpulex_dsx2 | MatScan | lbe | 441 | 446 | 0.85 | - | # TAAAAA |
| Dpulex_dsx2 | MatScan | br_Z2 | 442 | 449 | 0.86 | + | # TTTTATTT |
| Dpulex_dsx2 | MatScan | BR-C | 442 | 454 | 0.94 | - | # TAAATAAATAAAA |
| Dpulex_dsx2 | MatScan | Abd-B | 443 | 449 | 0.88 | + | # TTTATTT |
| Dpulex_dsx2 | MatScan | cad | 443 | 449 | 0.91 | + | # TTTATTT |
| Dpulex_dsx2 | MatScan | CG42234 | 443 | 449 | 0.85 | + | # TTTATTT |
| Dpulex_dsx2 | MatScan | CG4328 | 443 | 449 | 0.9 | + | # TTTATTT |
| Dpulex_dsx2 | MatScan | al | 444 | 450 | 0.85 | - | # TAAATAA |
| Dpulex_dsx2 | MatScan | Croc | 444 | 459 | 0.89 | - | # AAAAATAAATAAATAA |
| Dpulex_dsx2 | MatScan | Lim1 | 444 | 450 | 0.85 | + | # TTATTTA |
| Dpulex_dsx2 | MatScan | lbe | 445 | 450 | 0.91 | - | # TAAATA |
| Dpulex_dsx2 | MatScan | BR-C | 446 | 458 | 0.91 | - | # AAAATAAATAAAT |
| Dpulex_dsx2 | MatScan | Abd-B | 447 | 453 | 0.88 | + | # TTTATTT |
| Dpulex_dsx2 | MatScan | cad | 447 | 453 | 0.91 | + | # TTTATTT |
| Dpulex_dsx2 | MatScan | CG42234 | 447 | 453 | 0.85 | + | # TTTATTT |
| Dpulex_dsx2 | MatScan | CG4328 | 447 | 453 | 0.9 | + | # TTTATTT |
| Dpulex_dsx2 | MatScan | al | 448 | 454 | 0.85 | - | # TAAATAA |
| Dpulex_dsx2 | MatScan | Lim1 | 448 | 454 | 0.85 | + | # TTATTTA |
| Dpulex_dsx2 | MatScan | lbe | 449 | 454 | 0.91 | - | # TAAATA |
| Dpulex_dsx2 | MatScan | Abd-B | 451 | 457 | 0.88 | + | # TTTATTT |
| Dpulex_dsx2 | MatScan | cad | 451 | 457 | 0.91 | + | # TTTATTT |
| Dpulex_dsx2 | MatScan | CG42234 | 451 | 457 | 0.85 | + | # TTTATTT |
| Dpulex_dsx2 | MatScan | CG4328 | 451 | 457 | 0.9 | + | # TTTATTT |
| Dpulex_dsx2 | MatScan | BR-C | 452 | 464 | 0.92 | - | # ATAATAAAAATAA |
| Dpulex_dsx2 | MatScan | hb | 454 | 463 | 0.88 | - | # TAATAAAAAT |
| Dpulex_dsx2 | MatScan | lbe | 455 | 460 | 0.85 | - | # TAAAAA |
| Dpulex_dsx2 | MatScan | CG7056 | 456 | 463 | 0.88 | + | # TTTTATTA |
| Dpulex_dsx2 | MatScan | Ubx | 456 | 463 | 0.89 | + | # TTTTATTA |
| Dpulex_dsx2 | MatScan | abd-A | 457 | 463 | 0.87 | + | # TTTATTA |
| Dpulex_dsx2 | MatScan | Abd-B | 457 | 463 | 0.97 | + | # TTTATTA |
| Dpulex_dsx2 | MatScan | al | 457 | 463 | 0.85 | - | # TAATAAA |
| Dpulex_dsx2 | MatScan | Awh | 457 | 463 | 0.86 | + | # TTTATTA |
| Dpulex_dsx2 | MatScan | C15 | 457 | 463 | 0.85 | + | # TTTATTA |
| Dpulex_dsx2 | MatScan | cad | 457 | 463 | 1 | + | # TTTATTA |
| Dpulex_dsx2 | MatScan | CG15696 | 457 | 463 | 0.91 | + | # TTTATTA |
| Dpulex_dsx2 | MatScan | CG32105 | 457 | 463 | 0.87 | + | # TTTATTA |
| Dpulex_dsx2 | MatScan | CG42234 | 457 | 463 | 1 | + | # TTTATTA |
| Dpulex_dsx2 | MatScan | CG4328 | 457 | 463 | 1 | + | # TTTATTA |
| Dpulex_dsx2 | MatScan | Dfd | 457 | 472 | 0.86 | + | # TTTATTATTATTAGAT |
| Dpulex_dsx2 | MatScan | H2.0 | 457 | 463 | 0.99 | + | # TTTATTA |
| Dpulex_dsx2 | MatScan | Lim1 | 457 | 463 | 0.85 | + | # TTTATTA |
| Dpulex_dsx2 | MatScan | Lim3 | 457 | 463 | 0.86 | + | # TTTATTA |
| Dpulex_dsx2 | MatScan | repo | 457 | 463 | 0.85 | + | # TTTATTA |
| Dpulex_dsx2 | MatScan | CG18599 | 458 | 464 | 0.85 | - | # ATAATAA |
| Dpulex_dsx2 | MatScan | E5 | 458 | 464 | 0.88 | - | # ATAATAA |
| Dpulex_dsx2 | MatScan | ems | 458 | 464 | 0.85 | - | # ATAATAA |
| Dpulex_dsx2 | MatScan | eve | 458 | 464 | 0.85 | - | # ATAATAA |
| Dpulex_dsx2 | MatScan | H2.0 | 458 | 464 | 0.88 | - | # ATAATAA |
| Dpulex_dsx2 | MatScan | lbe | 458 | 463 | 0.93 | - | # TAATAA |
| Dpulex_dsx2 | MatScan | lbl | 458 | 463 | 0.9 | - | # TAATAA |
| Dpulex_dsx2 | MatScan | Oct | 458 | 465 | 0.85 | - | # AATAATAA |
| Dpulex_dsx2 | MatScan | pb | 458 | 464 | 0.86 | - | # ATAATAA |
| Dpulex_dsx2 | MatScan | cad | 460 | 466 | 0.87 | + | # ATTATTA |
| Dpulex_dsx2 | MatScan | CG42234 | 460 | 466 | 0.87 | + | # ATTATTA |
| Dpulex_dsx2 | MatScan | CG4328 | 460 | 466 | 0.99 | + | # ATTATTA |
| Dpulex_dsx2 | MatScan | H2.0 | 460 | 466 | 0.91 | + | # ATTATTA |
| Dpulex_dsx2 | MatScan | CG18599 | 461 | 467 | 0.85 | - | # ATAATAA |
| Dpulex_dsx2 | MatScan | E5 | 461 | 467 | 0.88 | - | # ATAATAA |
| Dpulex_dsx2 | MatScan | ems | 461 | 467 | 0.85 | - | # ATAATAA |
| Dpulex_dsx2 | MatScan | eve | 461 | 467 | 0.85 | - | # ATAATAA |
| Dpulex_dsx2 | MatScan | H2.0 | 461 | 467 | 0.88 | - | # ATAATAA |
| Dpulex_dsx2 | MatScan | lbe | 461 | 466 | 0.93 | - | # TAATAA |
| Dpulex_dsx2 | MatScan | lbl | 461 | 466 | 0.9 | - | # TAATAA |
| Dpulex_dsx2 | MatScan | Oct | 461 | 468 | 0.85 | - | # AATAATAA |
| Dpulex_dsx2 | MatScan | pb | 461 | 467 | 0.86 | - | # ATAATAA |
| Dpulex_dsx2 | MatScan | cad | 463 | 469 | 0.87 | + | # ATTATTA |
| Dpulex_dsx2 | MatScan | CG42234 | 463 | 469 | 0.87 | + | # ATTATTA |
| Dpulex_dsx2 | MatScan | CG4328 | 463 | 469 | 0.99 | + | # ATTATTA |
| Dpulex_dsx2 | MatScan | H2.0 | 463 | 469 | 0.91 | + | # ATTATTA |
| Dpulex_dsx2 | MatScan | ap | 464 | 470 | 0.89 | - | # CTAATAA |
| Dpulex_dsx2 | MatScan | CG18599 | 464 | 470 | 0.89 | - | # CTAATAA |
| Dpulex_dsx2 | MatScan | E5 | 464 | 470 | 0.92 | - | # CTAATAA |
| Dpulex_dsx2 | MatScan | ems | 464 | 470 | 0.89 | - | # CTAATAA |
| Dpulex_dsx2 | MatScan | eve | 464 | 470 | 0.91 | - | # CTAATAA |
| Dpulex_dsx2 | MatScan | H2.0 | 464 | 470 | 0.86 | - | # CTAATAA |
| Dpulex_dsx2 | MatScan | ind | 464 | 470 | 0.9 | - | # CTAATAA |
| Dpulex_dsx2 | MatScan | inv | 464 | 471 | 0.85 | - | # TCTAATAA |
| Dpulex_dsx2 | MatScan | lbe | 464 | 469 | 0.93 | - | # TAATAA |
| Dpulex_dsx2 | MatScan | lbl | 464 | 469 | 0.9 | - | # TAATAA |
| Dpulex_dsx2 | MatScan | Lim3 | 464 | 470 | 0.86 | - | # CTAATAA |
| Dpulex_dsx2 | MatScan | Oct | 464 | 471 | 0.87 | - | # TCTAATAA |
| Dpulex_dsx2 | MatScan | pb | 464 | 470 | 0.9 | - | # CTAATAA |
| Dpulex_dsx2 | MatScan | ro | 464 | 470 | 0.86 | - | # CTAATAA |
| Dpulex_dsx2 | MatScan | Vsx1 | 464 | 470 | 0.85 | - | # CTAATAA |
| Dpulex_dsx2 | MatScan | zen | 464 | 470 | 0.89 | - | # CTAATAA |
| Dpulex_dsx2 | MatScan | zen2 | 464 | 470 | 0.86 | - | # CTAATAA |
| Dpulex_dsx2 | MatScan | pan | 478 | 485 | 0.95 | - | # GTTTGATC |
| Dpulex_dsx2 | MatScan | B-H1 | 488 | 494 | 0.89 | - | # CTAAATG |
| Dpulex_dsx2 | MatScan | slbo | 491 | 498 | 0.91 | - | # ATTGCTAA |
| Dpulex_dsx2 | MatScan | Six4 | 513 | 518 | 0.92 | + | # TGAAAC |
| Dpulex_dsx2 | MatScan | slbo | 520 | 527 | 0.85 | - | # ATTGGAAA |
| Dpulex_dsx2 | MatScan | ara | 533 | 537 | 0.89 | - | # CAACA |
| Dpulex_dsx2 | MatScan | caup | 533 | 537 | 0.87 | - | # CAACA |
| Dpulex_dsx2 | MatScan | mirr | 533 | 537 | 0.89 | - | # CAACA |
| Dpulex_dsx2 | MatScan | ct | 543 | 548 | 0.92 | + | # CTGAAC |
| Dpulex_dsx2 | MatScan | fkh | 543 | 553 | 0.91 | - | # TGTTTGTTCAG |
| Dpulex_dsx2 | MatScan | ara | 545 | 549 | 0.93 | + | # GAACA |
| Dpulex_dsx2 | MatScan | caup | 545 | 549 | 0.88 | + | # GAACA |
| Dpulex_dsx2 | MatScan | mirr | 545 | 549 | 0.89 | + | # GAACA |
| Dpulex_dsx2 | MatScan | ara | 549 | 553 | 0.99 | + | # AAACA |
| Dpulex_dsx2 | MatScan | caup | 549 | 553 | 0.9 | + | # AAACA |
| Dpulex_dsx2 | MatScan | mirr | 549 | 553 | 1 | + | # AAACA |
| Dpulex_dsx2 | MatScan | Deaf1 | 555 | 560 | 0.87 | - | # TTCGCC |
| Dpulex_dsx2 | MatScan | pan | 576 | 583 | 0.86 | + | # CTTTGGTT |
| Dpulex_dsx2 | MatScan | Deaf1 | 582 | 587 | 0.98 | + | # TTCGTC |
| Dpulex_dsx2 | MatScan | onecut | 591 | 597 | 0.87 | + | # CTGATTG |
| Dpulex_dsx2 | MatScan | ara | 619 | 623 | 0.93 | - | # GAACA |
| Dpulex_dsx2 | MatScan | caup | 619 | 623 | 0.88 | - | # GAACA |
| Dpulex_dsx2 | MatScan | mirr | 619 | 623 | 0.89 | - | # GAACA |
| Dpulex_dsx2 | MatScan | ct | 620 | 625 | 0.86 | - | # GTGAAC |
| Dpulex_dsx2 | MatScan | CG11617 | 621 | 627 | 0.89 | + | # TTCACAT |
| Dpulex_dsx2 | MatScan | pan | 663 | 670 | 0.87 | + | # TTTTGAAT |
| Dpulex_dsx2 | MatScan | vvl | 666 | 671 | 0.98 | - | # TATTCA |
| Dpulex_dsx2 | MatScan | CG4328 | 670 | 676 | 0.9 | + | # TATATTG |
| Dpulex_dsx2 | MatScan | BR-C | 676 | 691 | 0.95 | + | # GTCTGTACTATTTTTT |
| Dpulex_dsx2 | MatScan | br_Z2 | 681 | 688 | 1 | + | # TACTATTT |
| Dpulex_dsx2 | MatScan | hb | 687 | 696 | 0.87 | - | # GGTTAAAAAA |
| Dpulex_dsx2 | MatScan | C15 | 688 | 694 | 0.85 | - | # TTAAAAA |
| Dpulex_dsx2 | MatScan | lbe | 688 | 693 | 0.85 | - | # TAAAAA |
| Dpulex_dsx2 | MatScan | ct | 690 | 695 | 0.89 | + | # TTTAAC |
| Dpulex_dsx2 | MatScan | lbe | 692 | 697 | 0.95 | + | # TAACCA |
| Dpulex_dsx2 | MatScan | ara | 708 | 712 | 0.91 | - | # ATACA |
| Dpulex_dsx2 | MatScan | mirr | 708 | 712 | 0.89 | - | # ATACA |
| Dpulex_dsx2 | MatScan | vvl | 708 | 713 | 0.88 | - | # TATACA |
| Dpulex_dsx2 | MatScan | Kr | 711 | 720 | 0.87 | - | # AATGGGATAT |
| Dpulex_dsx2 | MatScan | D | 715 | 725 | 0.85 | + | # CCCATTGATCT |
| Dpulex_dsx2 | MatScan | dTCF | 716 | 726 | 0.91 | + | # CCATTGATCTT |
| Dpulex_dsx2 | MatScan | pan | 717 | 724 | 0.87 | + | # CATTGATC |
| Dpulex_dsx2 | MatScan | Ttk | 732 | 739 | 0.87 | + | # GGTCCAGC |
| Dpulex_dsx2 | MatScan | ara | 740 | 744 | 0.99 | - | # AAACA |
| Dpulex_dsx2 | MatScan | caup | 740 | 744 | 0.9 | - | # AAACA |
| Dpulex_dsx2 | MatScan | lbe | 740 | 745 | 0.86 | - | # TAAACA |
| Dpulex_dsx2 | MatScan | mirr | 740 | 744 | 1 | - | # AAACA |
| Dpulex_dsx2 | MatScan | ct | 741 | 746 | 0.9 | - | # CTAAAC |
| Dpulex_dsx2 | MatScan | Deaf1 | 750 | 755 | 0.89 | - | # CTCGGC |
| Dpulex_dsx2 | MatScan | achi | 761 | 766 | 0.92 | - | # TGACAT |
| Dpulex_dsx2 | MatScan | CG11617 | 761 | 767 | 0.93 | - | # TTGACAT |
| Dpulex_dsx2 | MatScan | hth | 761 | 766 | 0.96 | - | # TGACAT |
| Dpulex_dsx2 | MatScan | vis | 761 | 766 | 0.95 | - | # TGACAT |
| Dpulex_dsx2 | MatScan | caup | 762 | 766 | 0.85 | - | # TGACA |
| Dpulex_dsx2 | MatScan | exd | 762 | 769 | 0.88 | - | # TATTGACA |
| Dpulex_dsx2 | MatScan | cad | 765 | 771 | 0.87 | - | # CTTATTG |
| Dpulex_dsx2 | MatScan | CG4328 | 765 | 771 | 0.93 | - | # CTTATTG |
| Dpulex_dsx2 | MatScan | lbe | 785 | 790 | 0.91 | + | # TAAATA |
| Dpulex_dsx2 | MatScan | br_Z2 | 786 | 793 | 0.94 | - | # AACTATTT |
| Dpulex_dsx2 | MatScan | z | 792 | 801 | 0.91 | + | # TTGAGAGACT |
| Dpulex_dsx2 | MatScan | dl | 832 | 842 | 0.87 | - | # TGGGAAACCCA |
| Dpulex_dsx2 | MatScan | br_Z2 | 842 | 849 | 0.92 | + | # ATCTATTT |
| Dpulex_dsx2 | MatScan | br_Z2 | 847 | 854 | 0.86 | + | # TTTTATTT |
| Dpulex_dsx2 | MatScan | Abd-B | 848 | 854 | 0.88 | + | # TTTATTT |
| Dpulex_dsx2 | MatScan | cad | 848 | 854 | 0.91 | + | # TTTATTT |
| Dpulex_dsx2 | MatScan | CG42234 | 848 | 854 | 0.85 | + | # TTTATTT |
| Dpulex_dsx2 | MatScan | CG4328 | 848 | 854 | 0.9 | + | # TTTATTT |
| Dpulex_dsx2 | MatScan | ct | 853 | 858 | 0.89 | + | # TTCAAC |
| Dpulex_dsx2 | MatScan | Lag1 | 855 | 861 | 0.91 | + | # CAACCAA |
| Dpulex_dsx2 | MatScan | pan | 856 | 863 | 0.86 | - | # CTTTGGTT |
| Dpulex_dsx2 | MatScan | ara | 875 | 879 | 0.89 | - | # CAACA |
| Dpulex_dsx2 | MatScan | caup | 875 | 879 | 0.87 | - | # CAACA |
| Dpulex_dsx2 | MatScan | mirr | 875 | 879 | 0.89 | - | # CAACA |
| Dpulex_dsx2 | MatScan | Deaf1 | 895 | 900 | 0.87 | - | # TTCGCC |
| Dpulex_dsx2 | MatScan | pan | 910 | 917 | 0.85 | - | # TTTTCATC |
| Dpulex_dsx2 | MatScan | slp1 | 910 | 920 | 0.87 | - | # ATGTTTTCATC |
| Dpulex_dsx2 | MatScan | ara | 915 | 919 | 0.99 | + | # AAACA |
| Dpulex_dsx2 | MatScan | caup | 915 | 919 | 0.9 | + | # AAACA |
| Dpulex_dsx2 | MatScan | mirr | 915 | 919 | 1 | + | # AAACA |
| Dpulex_dsx2 | MatScan | Deaf1 | 925 | 930 | 0.98 | - | # TTCGGC |
| Dpulex_dsx2 | MatScan | Deaf1 | 948 | 953 | 0.98 | + | # TTCGTC |
| Dpulex_dsx2 | MatScan | Lag1 | 956 | 962 | 0.86 | - | # CCACTAT |
| Dpulex_dsx2 | MatScan | Lag1 | 959 | 965 | 0.88 | - | # CCACCAC |
| Dpulex_dsx2 | MatScan | CF2-II | 965 | 973 | 0.86 | + | # GTATGTGTA |
| Dpulex_dsx2 | MatScan | Six4 | 981 | 986 | 0.95 | - | # TGAGAC |
| Dpulex_dsx2 | MatScan | ara | 992 | 996 | 1 | + | # TAACA |
| Dpulex_dsx2 | MatScan | caup | 992 | 996 | 1 | + | # TAACA |
| Dpulex_dsx2 | MatScan | mirr | 992 | 996 | 0.99 | + | # TAACA |
